# Clinical research framework proposal for ketogenic metabolic therapy in glioblastoma

**DOI:** 10.1186/s12916-024-03775-4

**Published:** 2024-12-05

**Authors:** Tomás Duraj, Miriam Kalamian, Giulio Zuccoli, Joseph C. Maroon, Dominic P. D’Agostino, Adrienne C. Scheck, Angela Poff, Sebastian F. Winter, Jethro Hu, Rainer J. Klement, Alicia Hickson, Derek C. Lee, Isabella Cooper, Barbara Kofler, Kenneth A. Schwartz, Matthew C. L. Phillips, Colin E. Champ, Beth Zupec-Kania, Jocelyn Tan-Shalaby, Fabiano M. Serfaty, Egiroh Omene, Gabriel Arismendi-Morillo, Michael Kiebish, Richard Cheng, Ahmed M. El-Sakka, Axel Pflueger, Edward H. Mathews, Donese Worden, Hanping Shi, Raffaele Ivan Cincione, Jean Pierre Spinosa, Abdul Kadir Slocum, Mehmet Salih Iyikesici, Atsuo Yanagisawa, Geoffrey J. Pilkington, Anthony Chaffee, Wafaa Abdel-Hadi, Amr K. Elsamman, Pavel Klein, Keisuke Hagihara, Zsófia Clemens, George W. Yu, Athanasios E. Evangeliou, Janak K. Nathan, Kris Smith, David Fortin, Jorg Dietrich, Purna Mukherjee, Thomas N. Seyfried

**Affiliations:** 1 Biology Department, Boston College, Chestnut Hill, MA 02467 USA; 2 Dietary Therapies LLC, Hamilton, MT 59840 USA; 3 Neuroradiology, Private Practice, Philadelphia, PA 19103 USA; 4 Department of Neurological Surgery, University of Pittsburgh Medical Center, Pittsburgh, PA 15213 USA; 5 Department of Molecular Pharmacology and Physiology, University of South Florida Morsani College of Medicine, Tampa, FL 33612 USA; 6 Department of Child Health, University of Arizona College of Medicine, Phoenix, Phoenix, AZ 85004 USA; 7 Department of Neurology, Division of Neuro-Oncology, Massachusetts General Hospital Cancer Center, Harvard Medical School, Boston, MA 02114 USA; 8 Cedars-Sinai Cancer, Cedars-Sinai Medical Center, Los Angeles, CA 90048 USA; 9 Department of Radiotherapy and Radiation Oncology, Leopoldina Hospital Schweinfurt, 97422 Schweinfurt, Germany; 10 Rayma Health, Maple Grove, MN 55311 USA; 11 Ageing Biology and Age-Related Diseases Group, School of Life Sciences, University of Westminster, London, W1W 6UW UK; 12 Research Program for Receptor Biochemistry and Tumor Metabolism, Department of Pediatrics, University Hospital of the Paracelsus Medical University, Müllner Hauptstr. 48, 5020 Salzburg, Austria; 13 Department of Medicine, Michigan State University, East Lansing, MI 48824 USA; 14 Department of Neurology, Waikato Hospital, Hamilton, 3204 New Zealand; 15 Department of Medicine, University of Auckland, Auckland, 1142 New Zealand; 16 Exercise Oncology & Resiliency Center and Department of Radiation Oncology, Allegheny Health Network, Pittsburgh, PA 15212 USA; 17 Ketogenic Therapies LLC, Elm Grove, WI 53122 USA; 18 School of Medicine, University of Pittsburgh, Veteran Affairs Pittsburgh Healthcare System, Pittsburgh, PA 15240 USA; 19 Department of Clinical Medicine, State University of Rio de Janeiro (UERJ), Rio de Janeiro, RJ 20550-170 Brazil; 20 Serfaty Clínicas, Rio de Janeiro, RJ 22440-040 Brazil; 21 Department of Oncology, Cross Cancer Institute, Edmonton, AB T6G 1Z2 Canada; 22 Department of Medicine, Faculty of Health Sciences, University of Deusto, 48007 Bilbao (Bizkaia), Spain; 23 Facultad de Medicina, Instituto de Investigaciones Biológicas, Universidad del Zulia, Maracaibo, 4005 Venezuela; 24 BPGbio Inc, Framingham, MA 01701 USA; 25 Cheng Integrative Health Center, Columbia, SC 29212 USA; 26 Metabolic Terrain Institute of Health, East Congress Street, Tucson, AZ 85701 USA; 27 Pflueger Medical Nephrologyand , Internal Medicine Services P.L.L.C, 6 Nelson Road, Monsey, NY 10952 USA; 28 Department of Physiology, Faculty of Health Sciences, University of Pretoria, Pretoria, 0002 South Africa; 29 Arizona State University, Tempe, AZ 85281 USA; 30 Department of Gastrointestinal Surgery and Department of Clinical Nutrition, Beijing Shijitan Hospital, Capital Medical University, Beijing, 100038 China; 31 Department of Clinical and Experimental Medicine, University of Foggia, 71122 Foggia, Puglia, Italy; 32 Integrative Oncology, Breast and Gynecologic Oncology Surgery, Private Practice, Rue Des Terreaux 2, 1002 Lausanne, Switzerland; 33 Medical Oncology, ChemoThermia Oncology Center, Istanbul, 34365 Turkey; 34 Department of Medical Oncology, Altınbaş University Bahçelievler Medical Park Hospital, Istanbul, 34180 Turkey; 35 The Japanese College of Intravenous Therapy, Tokyo, 150-0013 Japan; 36 University of Portsmouth, Portsmouth, PO1 2UP UK; 37 Department of Neurosurgery, Sir Charles Gairdner Hospital, Perth, 6009 Australia; 38 Clinical Oncology Department, Cairo University, Giza, 12613 Egypt; 39 Neurosurgery Department, Cairo University, Giza, 12613 Egypt; 40 Mid-Atlantic Epilepsy and Sleep Center, 6410 Rockledge Drive, Suite 610, Bethesda, MD 20817 USA; 41 Department of Advanced Hybrid Medicine, Graduate School of Medicine, Osaka University, Osaka, 565-0871 Japan; 42 International Center for Medical Nutritional Intervention, Budapest, 1137 Hungary; 43 George W, Yu Foundation For Nutrition & Health and Aegis Medical & Research Associates, Annapolis, MD 21401 USA; 44 Department of Pediatrics, Medical School, Aristotle University of Thessaloniki, Papageorgiou Hospital, Efkarpia, 56403 Thessaloniki, Greece; 45 Dr. DY Patil Medical College, Hospital and Research Centre, Pune, Maharashtra 411018 India; 46 Barrow Neurological Institute, Dignity Health St. Joseph’s Hospital and Medical Center, Phoenix, AZ 85013 USA; 47 Université de Sherbrooke, Sherbrooke, QC J1K 2R1 Canada

**Keywords:** Cancer, Glioblastoma, Metabolism, Research design, Warburg Effect, Glutaminolysis, Precision medicine

## Abstract

Glioblastoma (GBM) is the most aggressive primary brain tumor in adults, with a universally lethal prognosis despite maximal standard therapies. Here, we present a consensus treatment protocol based on the metabolic requirements of GBM cells for the two major fermentable fuels: glucose and glutamine. Glucose is a source of carbon and ATP synthesis for tumor growth through glycolysis, while glutamine provides nitrogen, carbon, and ATP synthesis through glutaminolysis. As no tumor can grow without anabolic substrates or energy, the simultaneous targeting of glycolysis and glutaminolysis is expected to reduce the proliferation of most if not all GBM cells. Ketogenic metabolic therapy (KMT) leverages diet-drug combinations that inhibit glycolysis, glutaminolysis, and growth signaling while shifting energy metabolism to therapeutic ketosis. The glucose-ketone index (GKI) is a standardized biomarker for assessing biological compliance, ideally via real-time monitoring. KMT aims to increase substrate competition and normalize the tumor microenvironment through GKI-adjusted ketogenic diets, calorie restriction, and fasting, while also targeting glycolytic and glutaminolytic flux using specific metabolic inhibitors. Non-fermentable fuels, such as ketone bodies, fatty acids, or lactate, are comparatively less efficient in supporting the long-term bioenergetic and biosynthetic demands of cancer cell proliferation. The proposed strategy may be implemented as a synergistic metabolic priming baseline in GBM as well as other tumors driven by glycolysis and glutaminolysis, regardless of their residual mitochondrial function. Suggested best practices are provided to guide future KMT research in metabolic oncology, offering a shared, evidence-driven framework for observational and interventional studies.

**Supplementary Information:**

The online version contains supplementary material available at 10.1186/s12916-024-03775-4.

## Background

### Standard of care for brain cancer management

Glioblastoma (GBM), the most common and aggressive primary brain tumor in adults, has one of the highest mortality rates of all cancers. Despite the advent of multimodality in neuro-oncology and emergence of novel therapies, long-term survival remains poor for most high-grade brain tumors [1–4]. In fact, median overall survival (mOS) for GBM is only marginally better today than it was in 1926: 14–21 months versus 8–14 months, respectively [5, 6]. More importantly, incremental improvements in mOS or progression-free survival (PFS) should not be confused with long-term survival, which remains less than 0.8% at 10 years from diagnosis [7, 8]. None of the current cytotoxic, molecularly targeted, or immune-based therapies have translated into robust improvements in long-term survival at the population level [9–11]. When deciding on palliative care, oncologists and patients may have a different understanding of therapeutic goals, and patients may not understand that the proposed treatments are “unlikely to be curative”, leading to inaccurate expectations [12, 13]. If therapeutic success is defined as long-term survival, it becomes clear that no major advancements have been made in GBM therapy despite a century of cancer research [14].

The current standard of care (SOC) involves maximal safe surgical resection, radiotherapy, and temozolomide chemotherapy, with an average mOS across clinical trials of 15.6 months (compared to 10.1 with surgery alone in historical cohorts), reaching a 5-year relative survival rate of less than 10% [15, 16]. A small improvement in mOS is observed in younger patients and high-grade gliomas with specific isocitrate dehydrogenase (*IDH*) mutations [17, 18]. The degree of surgical debulking is considered one of the most important prognostic factors, which could explain the survival differences between SOC (which includes debulking) and biopsy alone (without debulking) in best supportive care [19–21]. Elective treatments such as FDA-approved Tumor-Treating Fields (TTF) or novel immune-based therapies are occasionally offered after SOC for a modest increase in PFS and mOS [22, 23]. Unfortunately, despite providing desirable benefits in the form of transient tumor control and short-term survival, SOC does not yield meaningful improvements in *long-term survival* in comparison with post-surgical “best supportive care,” defined as symptom management (edema, nausea, pain, and malnutrition) [24, 25]. For recurrent GBM, consensus guidelines such as the NCCN encourage participation in clinical trials due to dissatisfactory treatment outcomes [26, 27]; unfortunately, clinical trials with various therapies, alone or in combination, have not yet achieved a significant extension of survival [28]. Therefore, patients should be informed of the expected benefits and adverse effects of existing therapeutic approaches to assist with informed consent and shared decision-making [9, 29]. Considering the dismal prognosis despite maximal SOC, novel clinical research frameworks are urgently needed to drive improvements in quality of life and long-term survival.

### Cancer as a mitochondrial metabolic disease: an emerging therapeutic paradigm

To address these challenges, we propose research guidelines for the management of GBM based on the understanding of cancer as a mitochondrial metabolic disease [30, 31].

Two major biochemical processes exist to generate energy in eukaryotic animal cells: substrate-level phosphorylation (SLP), also known as fermentation, and mitochondrial oxidative phosphorylation (OXPHOS), via electron transport chain-induced chemiosmosis. Non-tumoral cells are metabolically flexible: in the presence of oxygen, OXPHOS is sufficient to supply most of the energy requirements in a highly efficient and regulated system, relying on SLP only under certain physiological conditions [32]. Conversely, SLP can produce energy in the cytosol (e.g., Embden-Meyerhof-Parnas glycolytic pathway) and in the mitochondria (e.g., succinate-CoA ligase reaction in the TCA cycle), independent of OXPHOS [33, 34]. Cancer cells, including GBM, are largely dependent on increased SLP flux of glucose and glutamine through the glycolysis and glutaminolysis pathways, regardless of the presence of oxygen [33, 35–38]. In this protocol, we favor a functional definition of SLP dependency as the comparatively limited capacity of malignant cells to sustain long-term *proliferation* when forced to use OXPHOS-exclusive metabolism (e.g., deprivation of glucose and glutamine, the two primary SLP fuels, at the substrate, transport, or utilization level). Insufficient or “dysfunctional” OXPHOS in cancer cells, as compared to normal cells, is hypothesized to arise from the well-documented and universal abnormalities in the number, structure, dynamics, and collective functional efficiency of the mitochondrial population [39–45].

To our knowledge, there are no models of cancer that retain aggressive and limitless replicative capacity in the simultaneous absence of glycolysis and glutaminolysis, despite substitution with non-fermentable OXPHOS fuels (e.g., ketone bodies, fatty acids, pyruvate, lactate), as recapitulated by essential nutrient constraints in cell culture [46–48]. Similarly, neither basic nor clinical research to date supports the notion that tumors with certain mutations (e.g., BRAF V600E) can effectively metabolize fatty acids or ketone bodies to maintain constant growth after effective dual targeting of glucose and glutamine, even if they may do so over short experimental endpoints as long as SLP flux is maintained [49–52]. While it is possible that insights from in vitro mechanistic studies do not fully translate to the in vivo condition [53–55], we hypothesize that the minimal bioenergetic requirements for cell viability (ATP sufficiency) may be applicable across model systems, even if heterogeneity in fuel utilization may arise once energy constraints have been met. Therefore, historical controversies regarding the role of OXPHOS in cancer may have originated from imprecise definitions; as stated by Otto Warburg himself, “we have here a perfect example of a dispute about words” [56, 57].

**Fig. 1:**
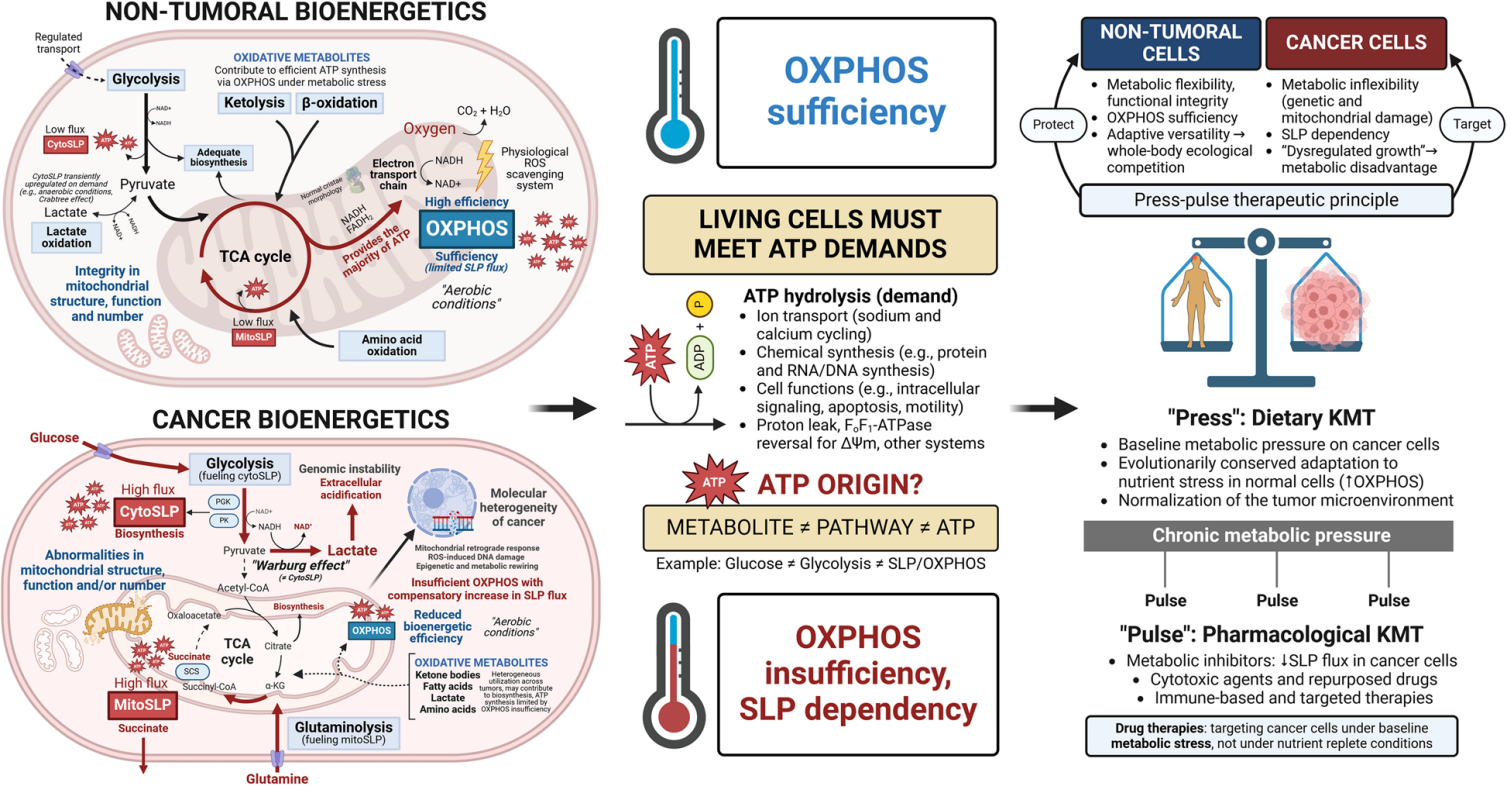
Simplified diagram of normal and cancer cell metabolism, with special emphasis on ATP synthesis (SLP and OXPHOS). All living cells must meet their ATP demands. Normal cells, including growth-regulated proliferating cells, generate the majority of ATP through the multi-step, ultrastructure-dependent process of OXPHOS. Cancer cells exhibit abnormalities in mitochondrial structure, function and/or number, as well as increased biosynthetic and redox demands, leading to a comparatively reduced efficiency of OXPHOS and compensatory upregulation of cytosolic and mitochondrial SLP. Cytosolic SLP is driven by glycolytic flux but is not synonymous with the Warburg effect (aerobic lactic acid fermentation). Oxidative metabolites can feed into the TCA cycle through catabolic pathways (glycolysis, glutaminolysis, lactate oxidation, β-oxidation, ketolysis), contributing to both SLP and OXPHOS; the total ATP yield is determined by nutrient availability and transport, as well as pathway flux, integrity, and efficiency. Cell division can be constrained by biosynthesis in the excess (assuming sufficient ATP), but energy is limiting for survival under nutrient depletion. The goal of KMT is to synergize with other therapies by targeting SLP flux in cancer cells and upregulating OXPHOS in normal cells, increasing metabolic stress and whole-body ecological competition

To avoid these issues, we identify “respiratory insufficiency” or “insufficient OXPHOS” as the therapeutically exploitable fact that cancer cells, unlike normal cells, appear unable to proliferate exclusively via OXPHOS when SLP is absent, not by the relative degree of mitochondrial function they may still retain [58–60]. Residual OXPHOS is a quantifiable category but, from a purely utilitarian point of view, it may not be able to support long-term proliferation in the absence of sufficient SLP flux, representing a targetable difference between non-tumoral and tumoral cells [61]. The proposed metabolic dependencies are summarized in Fig. 1. In this model, oxidative fuel utilization becomes functionally constrained by baseline SLP requirements and absolute OXPHOS efficiency, not substrate uptake or labeling, accounting for the relative metabolic heterogeneity across tumors (for example, in ketolytic activity) [62–65]. From a translational perspective, attaining a sufficient level of nutrient stress in vivo will likely require whole-body physiological adaptations (recapitulating fasting metabolism) as well as pharmacological interventions (metabolic inhibitors), reducing the effective ATP/biosynthetic output of the glycolytic and glutaminolytic pathways even if the input metabolites are still present in the tumor microenvironment. In preclinical models, dietary interventions that induce or “mimic” fasting have been tested to protect normal cells and potentiate the anti-tumoral effects of such metabolic inhibitors [66–69], but most clinical trials to date involved differential stress sensitization to conventional chemoradiotherapy rather than diet-drug combinations directed exclusively at cancer metabolism [70, 71].

Regrettably, standard GBM therapeutics are not designed to take advantage of the metabolic vulnerabilities of cancer cells; instead, they focus on DNA repair mechanisms. In fact, as an unintended consequence of non-specific cell damage, radiotherapy has been shown to induce detrimental metabolic changes and inflammation in the tumor microenvironment, impacting the phenotype of recurrence, which should be weighed against the desirable short-term cytotoxic or immune-potentiation effects [72–75]. In a similar fashion, temozolomide may increase systemic inflammation and tumor-driver mutations [76, 77]. Both brain-directed radiotherapy and systemic antineoplastic therapy can result in neurological complications (including brain tissue necrosis, brain atrophy, and neurocognitive impairment), which should be prevented if long-term survival is expected [78]. Furthermore, as part of supportive therapy, patients with brain cancer often receive corticosteroids (e.g., dexamethasone) to reduce vasogenic edema [27, 79]. The injudicious use of corticosteroids has been questioned due to correlations with reduced survival via dysregulated glucose metabolism, increased insulin signaling and immune suppression [80–88]. Current recommendations specify that “the lowest dose of steroids should be used for the shortest time possible,” in contrast with the “traditional, often uncritical use of steroids” [80], but this advice has yet to be widely adopted [89–91]. Finally, bevacizumab, a second-line anti-angiogenic therapy, may harbor unwanted adverse effects by facilitating distal tumor invasion through the neural parenchyma and perivascular network, without offering improvements to long-term survival [92–94].

While conventional chemoradiotherapies in GBM are well-intentioned, not addressing the unique characteristics of cancer metabolism may hinder their long-term effectiveness. Given the emphasis on patient autonomy in contemporary medical ethics, we advocate for well-informed patients to actively participate in their disease management, fostering supportive follow-up care to explore suitable clinical trials and complementary therapies [95–98]. Therefore, to reach a broader patient population, novel evidence-based treatments must be developed, tested, and accepted into standard clinical guidelines. In pursuit of this goal, accumulating evidence suggests that targeting glycolysis and glutaminolysis while transitioning the patient’s whole-body physiology into *therapeutic* *ketosis* could be an effective and translationally viable antineoplastic strategy [35]. Winter and colleagues coined the term “Ketogenic Metabolic Therapy” (KMT) to describe the systemic metabolic changes induced by very low carbohydrate (ketogenic) diets, calorie restriction, and/or fasting [99].

In the current framework, KMT is redefined and expanded as an “umbrella” term that includes long-term dietary, physical activity, and lifestyle modifications (requiring objective, measurable biological outcomes), combined with pharmacological targeting of glycolysis, glutaminolysis, and the tumor microenvironment. KMT is increasingly recognized as an emerging therapeutic approach for a broad range of cancers, while also improving quality of life [99–114].

Very low-carbohydrate, moderate-protein, high-fat ketogenic diets (KDs) induce a metabolic state of increased glycolytic substrate competition for cancer cells while also elevating non-fermentable ketone bodies to serve as an alternative energy source in normal cells [63, 99, 115–117]. In this context, KDs, calorie restriction, and fasting are anti-angiogenic, anti-inflammatory, and anti-invasive and can facilitate cancer cell death through multiple mechanisms [118–126]. Additionally, ketone body metabolism will enhance the ΔG′ATP hydrolysis in normal cells, thus awarding normal cells a bioenergetic advantage over tumor cells [127, 128]. A reduction in the rate of SLP flux will also lower the acidity in the tumor microenvironment, subsequently reducing inflammation and potentially limiting distant metastases [129]. Activities associated with cancer cell proliferation, such as biomass synthesis, are also inhibited by restricting the rate of glucose and glutamine fermentation [130, 131].

Dietary KMT has been found to interact synergistically with other drugs, procedures, and specific molecular tumor characteristics such as the *IDH1-R132H* mutation [132–134]. Gain-of-function *IDH* mutations can induce the production of 2-hydroxyglutarate (2-HG), an “oncometabolite” with aberrant epigenetic and immunosuppressive effects [135]. At the same time, accumulation of 2-HG may inhibit SLP flux, limiting the biomass and energy synthesis required for tumor growth [136–139]. From a metabolic perspective, in the specific case of high-grade glioma, *IDH1*-*R132H* could be viewed as a “therapeutic” mutation. In light of the inconsistent clinical outcomes with IDH inhibitors in high-grade gliomas so far [140, 141], we and others have proposed that “instead of shutting down mutant IDH enzymes, exploiting the selective vulnerabilities caused by them might be another attractive and promising strategy” [142].

It is important to mention, however, that dietary changes alone are unlikely to control tumor progression in most patients. While rigorous calorically restricted KDs and fasting may be effective in targeting glycolysis, insulin, and growth signaling, they do not adequately inhibit glutaminolysis [143–147]. Consequently, it will be essential to design and test KMT protocols with drugs that also inhibit glutaminolysis at the substrate, enzyme, and/or transport level. Current perspectives on leveraging cancer metabolism are mixed and often contradictory, although most agree on the need for combinatorial approaches [70, 148]. We propose that the best possibility of effective metabolic therapies will involve the simultaneous targeting of glucose and glutamine (specifically, SLP flux) after whole-body adaptation to therapeutic ketosis, leading to a normalization of the tumor microenvironment and enhancement of OXPHOS function and adaptive capacity in normal cells [129, 132, 143].

It should be noted that most early clinical trials explored additivity with SOC of either dietary modification alone (e.g., KDs, caloric restriction, amino acid depletion, fasting-mimicking protocols) [149], or single pathway metabolic inhibition (e.g., systemic glucose or insulin regulation via metformin or SGLT2 inhibitors; glycolysis inhibitors such as 2-Deoxy-D-glucose; glutaminolysis inhibitors such as CB-839 or DON prodrugs) [70].

In preclinical models, KDs in monotherapy induce predominantly favorable survival-prolonging effects across syngeneic and xenogeneic models, with variability in outcomes attributable to methodological differences (timing of intervention, tumor localization, diet composition, and degree of caloric restriction) [150]. Experimental factors such as failure to consistently reduce glycemia/insulin (despite increases in ketonemia), diet initiation (before or after tumor implantation), composition (ketogenic ratio), and palatability, as well as ad libitum or restricted feeding, could account for diverging results even when using identical tumor models [151, 152].

For high-grade glioma therapy, a cumulative total of 187 patients have been treated in more than 13 clinical studies thus far [153], demonstrating feasibility, safety, and tolerability, as well as improvements in quality of life and self-management [154, 155]. Additionally, more than 60 ongoing clinical trials are testing KDs in combination with standard, immune-based, and other targeted approaches (such as PI3K inhibitors), in GBM and other solid malignancies [156]. Unfortunately, there are no established “therapeutic targets” for clinical implementation beyond achieving a minimal state of ketosis (usually at a very modest ≥ 0.3 mM capillary βHB) and, if possible, sporadic but not sustained improvements in glycemia or insulin signaling; these have *not* been considered primary endpoints in any published study so far. If we conceptualize the KD as a bona-fide systemic “drug” intervention to reduce glycolytic flux, we lack data describing the area under the curve (AUC) of different ranges of glycemia and the anti-tumor effects across time. We suggest that future clinical trials should be designed to reach surrogate biomarkers of biological efficacy (such as real-time monitoring and stratification based on glycemia and ketonemia ranges, or chronic insulin suppression), rather than relying on self-reported dietary adherence. Conversely, there has been extensive preclinical development of pharmacological inhibitors aimed at nearly all metabolic pathways identified as upregulated or aberrant in cancer, subsequently added to various SOC regimens upon reaching clinical testing (without dietary intervention) [157]. Canonical pathways include glycolysis and glutaminolysis, but also other amino acids (methionine, arginine, tyrosine), the electron transport chain, fatty acid oxidation, lactate transport, mutant IDH enzymes, the kynurenine pathway, and even ketolysis. We have limited our proposal to mechanisms related to ATP synthesis, with the intention of establishing a clear therapeutic prioritization (SLP > OXPHOS). The goal of this framework is to formalize and build upon previous studies by constructing rational combinatory diet-drug approaches.

We acknowledge that the pleiotropic effects of dietary KMT may be equally mediated through decreases in growth signaling (insulin/IGF-1, AMPK, PI3K/AKT/mTOR axis), immune responses, post-translational epigenetic modification, gut microbiome, and/or regulation of the systemic hormonal milieu, rather than direct suppression of ATP-generating pathways [158, 159]. It is also possible that cancer cells exhibit increased sensitivity to SLP targeting due to biosynthetic or redox requirements (NAD^+^/NADH, NADP^+^/NADPH) [130, 160]. However, we argue that bioenergetics are interconnected with all the above, with major relevance for cell viability under metabolic stress, while intra/extracellular growth factors and biosynthesis may be determining of maximal proliferation (assuming baseline viability, and thus ATP sufficiency). Accordingly, it can be expected that healthy cell populations will display unique vulnerability thresholds to combined diet-drug metabolic pressure, carrying a risk of toxicity (e.g., rapidly proliferating immune and epithelial cells are more sensitive to pharmacological inhibition of glutamine) [161]. While we hypothesize that neoplastic cells are comparatively more susceptible to metabolic stress due to SLP dependency, mutational burden, and dysregulated growth itself, we aim to minimize off-target effects by following the press-pulse therapeutic principle, where drugs with a narrow therapeutic index (such as cytotoxic agents or metabolic inhibitors) are carefully dose-escalated and applied intermittently on a “metabolic priming” dietary KMT baseline [132].

## Purpose and rationale

Building upon this knowledge, we offer a framework for future research on KMT with additional pharmacological targeting of glycolysis and glutaminolysis as a minimally toxic therapeutic strategy for GBM management. The resulting shift to fat-derived ketone body metabolism allows for the relative reduction of glucose and glutamine-driven SLP flux while maintaining normal cell function by upregulating oxidative metabolism and increasing competitive evolutionary pressure [105, 145, 162]. The proposed drugs and strategies are intended to further restrict biosynthetic and bioenergetic pathways in tumor tissues. We have constructed this proposal by synthetizing the expert opinion of researchers and clinicians involved in previous preclinical and translational KMT research. Importantly, while this approach was developed primarily for GBM, the mechanistic basis should be applicable to all malignant cancers exhibiting SLP dependency on glucose and glutamine, as defined above [35, 115]. In this case, GBM was selected due to poor SOC outcomes and ethical considerations, as well as the potential benefit of therapeutic ketosis to seizure management and intracranial edema, rather than intrinsic bioenergetic characteristics [163].

It is important to acknowledge that forthcoming clinical research on cancer metabolism will likely involve combined testing with standard chemoradiotherapeutics as well as novel targeted and immune-based treatments, as the natural consequence of the incremental “one drug-one target” model [164, 165]. Under this research paradigm, current SOC serves as the gold standard, while KMT is tested as a secondary, adjuvant therapy. In this scenario, the utility of KMT is being demonstrated to enhance the anti-tumor effects of radiotherapy, chemotherapy, and targeted approaches (e.g., VEGF and immune checkpoint inhibitors) across different cancer models, via reductions in tumor nutrient utilization, hypoxia, inflammation, invasion, and angiogenesis, as well as regulation of pathways mediating tumor growth such as mTOR, insulin-PI3K, AMPK-PGC-1α, autophagy, epigenetic signaling, immune recognition, and multiple other pleiotropic mechanisms [68, 126, 158, 166–171]. In this way, changes in metabolism are being shown to mimic or potentiate the action of pharmaceutical agents, often without additional toxicity.

In the proposed framework, KMT is positioned as an evolutionarily advantageous prerequisite “metabolic priming” baseline upon which other cytotoxic therapies are introduced to assess potential synergy, additivity, or antagonism, rationalizing research priorities. It is an implicit assumption that clinical studies exploring precision nutrition or single metabolic inhibitors as adjuncts with SOC will be carried out in parallel, particularly for tumors where SOC offers a well-established track record of survival benefit; in cases where SOC may be deemed insufficient (as determined by the patient), a conceptual reframing of KMT at the foundational level may provide an ethical opportunity to explore the effectiveness of standalone diet-drug metabolic targeting.

A growing body of evidence suggests that well-formulated KDs can slow tumor progression, but most published reports to date have lacked a robust, modifiable protocol for clinical implementation and data collection. There is a lack of consensus for optimal KD therapy in cancer, leading to a heterogeneity of methodological approaches and lapses in effective monitoring [153]. Poor standardization has led to difficulties with inter-study comparability, as not all protocols described as “ketogenic” will offer therapeutic benefits in cancer-specific settings [172, 173]. A general, isocaloric/eucaloric, ad libitum KD is not synonymous with dietary KMT. The application of KDs in cancer should be nuanced and must fulfill a set of measurable biological criteria, with each patient exhibiting an individualized response over time. It is therefore essential to record data systematically (ideally, in real time), correlating cumulative physiological changes with anti-tumor effects. As such, the glucose-ketone index (GKI) was developed as a unifying biomarker for assessing “biological” compliance and outcomes in brain cancer [174]. Rather than relying on self-reported dietary compliance, any evaluation of clinical efficacy should be correlated with measurements of blood glucose and blood ketones (which can then be used to derive the GKI), as well other objective biological measurements (e.g., insulin, metabolic imaging, metabolomics), allowing for inter-study comparisons and external validity under different methodologies [102, 134, 145, 175].

It should be noted that a single dietary intervention is unlikely to affect all patients equally despite standardization efforts, with population-level genetic variability across endocrine and metabolic phenotypes [176, 177]. We recognize that real-world, large-scale clinical implementation of KMT will carry inherent heterogeneity that cannot (and perhaps should not) be avoided, granting patients and clinicians the freedom to adapt to specific and changing needs. However, it is necessary to develop initial best practices for KMT to serve as an evidence-based reference point without sacrificing therapeutic efficacy, addressing challenges raised in previous reports and being mindful of resource constraints for clinical research in smaller, financially constrained institutions.

### Ketone body metabolism in cancer: why therapeutic ketosis?

Russell Wilder at the Mayo Clinic formally developed the KD as a treatment for pediatric epilepsy in the 1920s, although various forms of very low carbohydrate diets and fasting have been used empirically for seizure control, diabetes, obesity, and other diseases since antiquity [178]. Prescription of KDs for epilepsy declined with the advent of new anticonvulsants but continues to be a cornerstone in the treatment of drug-resistant epilepsy as well as inborn errors of carbohydrate metabolism [179–181]. Recently, KDs experienced a major resurgence in clinical applications, particularly for insulin resistance, obesity, and neuroprogressive disorders [158, 182, 183], while ketogenically compensated glucose modulation as a cancer therapy has been described more than 80 years ago in a case series by Brünings [184].

Achieving stable therapeutic ketosis requires adjustments to the macronutrient composition of the diet. The KD is defined as a dietary pattern that is very low in carbohydrates (typically less than 20 g/day, which depletes liver glycogen and initiates ketogenesis), adequate in high-quality protein (sufficient for muscle maintenance, without excessive contribution to endogenous glucose production), and variable in fat, depending on whether it is intended to be hypocaloric (loss of adipose tissue), eucaloric (weight maintenance), or hypercaloric (recovery of adipose tissue). Restricted consumption of carbohydrates elicits a physiological metabolic adaptation favoring fat-derived fuels over glucose, resulting in the endogenous production of water-soluble metabolites collectively known as ketone bodies: acetoacetate, beta-hydroxybutyrate (βHB), and acetone [128, 185].

Acetoacetate and βHB are synthesized predominantly in the liver and exported into the bloodstream, serving as a “glucose substitute” for energy and biosynthesis in mitochondrially healthy cells [186]. Acetone is a breakdown product of acetoacetate that is released in breath and urine [187]. Acetoacetate and βHB are readily oxidized by all major organs, except for the liver, which relies on fatty acid oxidation and gluconeogenic substrates under glycogen depletion [188, 189]. After ketogenic adaptation, ketone bodies can supply more than 50% of the energy requirements of the human body, and over 70% of the brain’s energy needs [190–192]. From an endocrine perspective, dietary carbohydrate restriction reduces plasma glucose excursions, bolus insulin spikes, and basal insulin levels, removing insulin’s suppression of key enzymes controlling ketogenesis [193, 194]. Moreover, glucagon secretion decreases over time, further reducing basal hepatic glucose output (glycogenolysis and gluconeogenesis), glucose availability, and basal insulin [195]. A more in-depth discussion regarding physiological requirements for exogenous carbohydrates and endogenous glucose production, as well as metabolic acidosis, is offered in Additional File 1: Appendix 1.

## Standardizing KDs for biological efficacy

Different versions of the KD have been described in both scientific and lay texts, often including conflicting advice, especially for cancer management. This has led to widespread confusion in the public sphere and obstacles for clinical implementation. In the following sections, we summarize ketogenic procedures that have been tested for GBM. These practical definitions may help in choosing the intervention that best suits a particular need, with most seeking as much flexibility as possible without compromising therapeutic efficacy. It is important to remember that efforts to improve diet adherence, which are vital for patient accrual, are still bound by the GKI or other objective metrics of metabolic and tumor responses.

The GKI is the ratio of glucose to βHB, the two metabolites of interest in dietary KMT [174]. Glucose and ketones are assessed by capillary blood sampling using specialized handheld glucometers or extrapolated from interstitial fluid measurements using real-time wearable monitors. Steady-state GKI levels are used to estimate the degree of therapeutic ketosis and other biological processes, such as insulin signaling, growth-promoting pathways, and systemic inflammation, which are not readily accessible for repeated or real-time sampling, and are generally correlated with persistent decreases in glycemia and increases in ketonemia (resulting in a decreased GKI) [102]. In future clinical trials, it will be essential to capture the AUC and variability of glycemia and ketonemia over extended study intervals to establish statistical correlations with therapeutic efficacy, as short-term metabolic changes are not expected to induce sufficient competitive metabolic pressure. To facilitate longitudinal tracking, an updated version of the GKI tracking tool is provided in Additional File 2: GKI tracking spreadsheet.

**Fig. 2:**
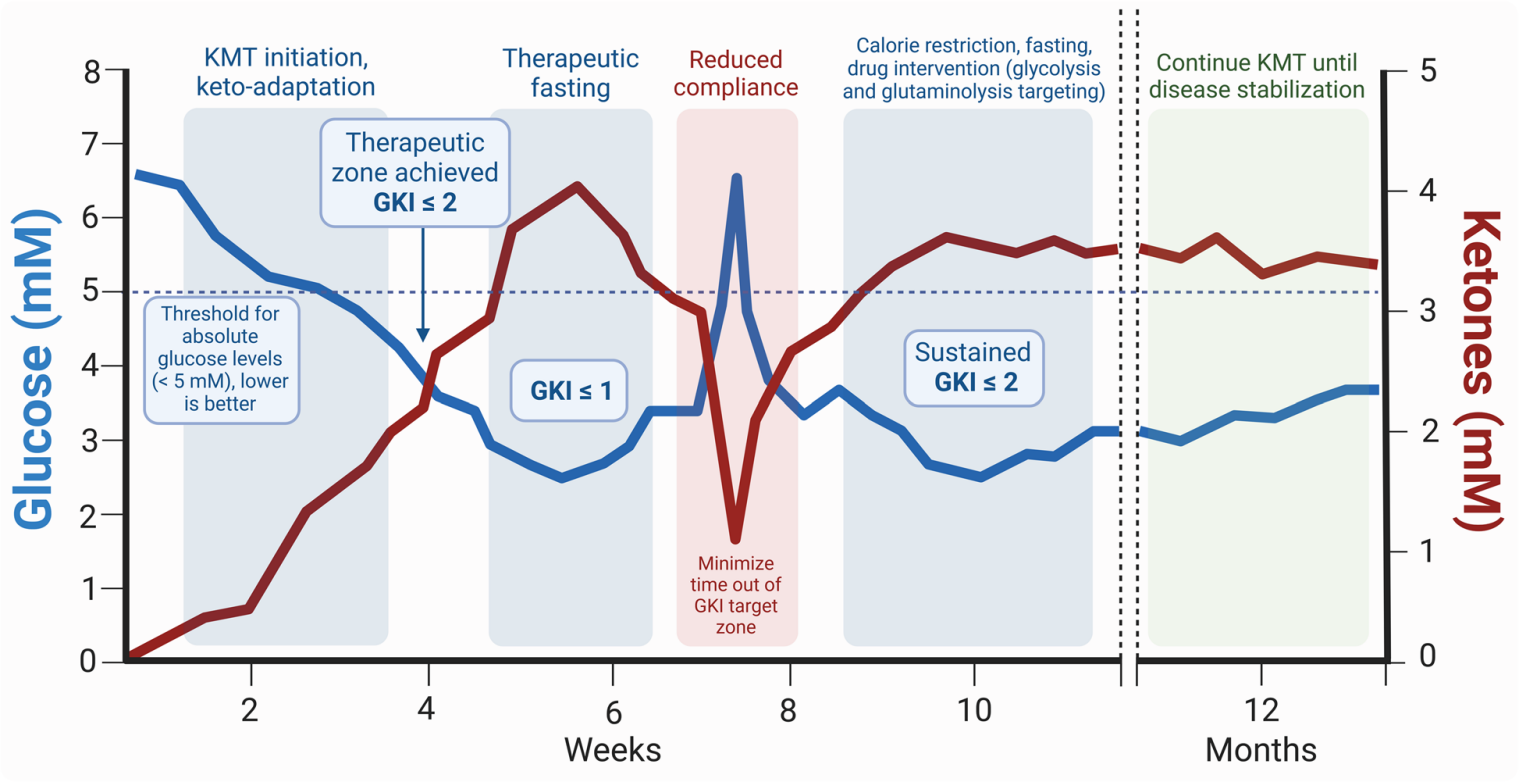
Illustrative diagram of blood glucose, βHB, and GKI during different phases of dietary KMT. Note that the suggested glucose and ketone levels are representative of inter-individual and intra-individual variability, not prescriptive. In this example, after initiating a GKI-adjusted KD, glycemia is maintained below 5 mM and ketonemia above 1–2 mM. The proposed therapeutic zone has been achieved once glucose levels are less than two-fold ketone levels (e.g., 5 mM glucose, 2.5 mM βHB, GKI ≤ 2), and optimal when glucose levels are equal or lower than ketone levels (e.g., 4 mM glucose, 4 mM βHB, GKI ≤ 1). Absolute glucose levels should be at their physiological minimum. Dietary, stress, or therapy-induced excursions (e.g., corticosteroids) should be minimized. Exercise-induced gluconeogenesis is expected and offset via skeletal muscle demand. As a long-term therapeutic strategy, dietary KMT may continue as long as there is evidence of persistent disease or risk of recurrence. Real-time GKI tracking is recommended in research settings to avoid ambiguity regarding biological outcomes

The baseline dietary strategy is to follow a macronutrient distribution that facilitates ketogenic adaptation, preserves lean body mass (LBM), and maintains an adequate micronutrient balance, while keeping sustained daily GKI values below 2.0, ideally near 1.0 or below (Fig. 2). In clinical studies, averaged weekly, monthly, and yearly values should be collected for a data-driven appraisal of efficacy [134]. Continuous, uninterrupted maintenance of therapeutic GKI ranges may be preferable to occasional, short-term, or cyclical strategies [145]. It is important to note that dietary KMT is defined by a gradual, sustained, whole-body metabolic and endocrine adaptation in fuel partitioning. Absolute blood glucose levels should be consistently below 90 mg/dl or 5 mM; this is an arbitrary, statistically derived cut-off that has been associated with improved survival but does not define a known biological constraint [85, 102, 196, 197]. Preclinical and clinical evidence suggests that patients should aim for the lowest, physiologically safe and sustainable glucose and insulin levels [198–202], where the proxy indicating effective insulin suppression is via elevated blood ketone levels throughout the day, especially during the evening pre-prandial time [203, 204]. Patients with cancer can present with normal to low glycemia (and consequently low fasting insulin) due to tumor hypermetabolism, concealing metabolic dysregulation; it is therefore valuable to measure glycemia, ketonemia, and insulin secretion during the feeding period (e.g., before dinner). A morning fasted reading can be misleading, as healthy populations (and even type 2 diabetics) can present with low levels of nutritional ketosis after the overnight fast [205, 206].

### Classic KD: ketogenic ratios, macronutrients, diet adherence

The classic KD developed by Wilder is still prescribed in the epilepsy field. In adults, the patient’s energy needs are initially calculated using standard formulas in a 3:1 to 4:1 ratio of fat grams to combined carbohydrate-plus-protein grams. The main benefit of this approach is that both carbohydrate and protein are kept very low (together, < 10% of total calories), making it easier to reach higher levels of ketosis. There is consensus on how to maintain this diet, several medical foods are available, contraindications are clearly defined, and potential side effects can be proactively monitored and addressed [207–209]. The classic KD may be too rigid for broad clinical application and adherence across heterogeneous cancer populations, but it serves as a well-documented reference template from which to extrapolate introductory practical guidance (e.g., ketogenic recipes and cookbooks), long-term patient monitoring and diet troubleshooting [208].

Recently, macronutrient distributions have been adapted for classic KDs, as they are more intuitive than diet ratios. The macronutrient distribution (% energy) of the classic KD is commonly defined as 88–90% fat, 6–8% protein, and 4% carbohydrate. It should be noted that the daily energy intake will determine the absolute quantity of macronutrients (grams), making flexible distributions less suitable for higher caloric expenditures. For example, a KD consisting of 10% carbohydrate for a total caloric intake of 2500 kcal/day equals to approximately 60 g/day, which may be incompatible with therapeutic ketosis for most patients. At the physiological level, reaching sufficient liver ketogenesis typically involves a carbohydrate intake below 20–50 g/day, depending on the metabolic fitness of the individual [210]. Consequently, the maximum threshold of carbohydrate intake that still allows for the desired degree of ketosis and glycemic control will need to be individually titrated, followed by protein for muscle maintenance, and fat for the desired caloric density. An automated calculator based on the Mifflin-St Jeor equation is provided in Additional File 2: GKI tracking spreadsheet; it is important to note that predictive equations can underestimate the energy requirements of patients with cancer, which are ultimately dictated by the desired weight evolution over time [211, 212].

Diet adherence to classic high-ratio KDs can be perceived as challenging in free-living adults [213]. However, highly motivated patients with an adequate understanding of the scientific rationale have been able to maintain strict compliance over prolonged periods [214, 215]. Patients with cancer may require more protein to preserve LBM, especially if the diet is calorically restricted [216, 217]. In this regard, KDs with adequate protein and micronutrient content (such as paleolithic KDs), which induce a lower than baseline, stable GKI, could improve feasibility and long-term compliance [134, 203, 218, 219].

### Calorically restricted KD (KD-R): reaching GKI targets while preserving muscle mass

The classic KD was originally intended to be eucaloric or unrestricted (“ad libitum”) to allow for the appropriate maturation of pediatric patients with epilepsy, and while therapeutic benefits have been reported in preclinical cancer models in both unrestricted and calorically restricted amounts [150, 220], most clinical studies focused on eucaloric feeding to promote weight maintenance [114, 155, 221]. A failure to reduce proliferation could be a consequence of persistently elevated glucose availability, endocrine, or growth-promoting signaling due to energy surplus, despite shifting to a ketogenic state [222, 223].

In contrast, KDs consumed in calorically restricted amounts, resulting in a gradual, deliberate reduction of fat mass (with preserved muscle mass), could produce better cumulative, steady-state GKI values, in tandem with the underlying metabolic and signaling effects, such as insulin suppression [102, 134, 224, 225]. Calorie restriction (independent of macronutrient composition) increases metabolic pressure on tumor cells by modulating nutrient-sensing pathways [226–228]. Similarly, reduced energy intake makes it easier to adapt to the higher overall fat intake despite enhanced satiety [229].

The KD-R protocol should be personalized in duration, periodicity, and degree, while being monitored to ensure mild calorie restriction does not increase the risk of malnutrition. After setting a carbohydrate limit to induce ketogenesis and calculating protein needs to preserve muscle mass, the energy density of the diet will be adjusted by total fat intake. It may be necessary to exclude calorie restriction in malnourished or underweight patients (as a rule, BMI < 18). In practice, patients with lower body fat percentages can follow KD-R in a cyclical fashion, introducing a return to previous isocaloric conditions or a slight caloric surplus when weight recovery is required; these intervals should still be GKI-adjusted, that is, adhering to ketogenic ratios and aiming for the lowest possible GKI.

In all cases, excessive LBM loss should be avoided. A classic KD-R with a high ketogenic ratio is typically too low in protein for long-term muscle maintenance in adults. Emerging evidence suggests that a well-formulated, protein sufficient KD may exert global anti-cachexic effects by decreasing pro-inflammatory cytokines and metabolites (inducing a protein-sparing metabolic shift) [230–234], with further anti-catabolic effects mediated by ketone bodies [235–238]. Therefore, protein intake should be modified for sufficiency, monitoring the impact on GKI, glucose variability, and ketogenesis. Adequate protein intake has either neutral or minor effects on ketogenesis and insulin signaling, as well as hepatic/renal gluconeogenesis [239–241]. Total protein intake can be started at the minimum recommended daily intake of 0.8 g/kg of body weight (for a sedentary individual in isocaloric conditions), and then increased progressively based on factors related to protein needs, such as age, physical activity, or health status [242–244].

It is important to emphasize that dietary amino acids cannot be restricted for clinically relevant glutamine depletion, as glutamine levels remain relatively stable through de novo synthesis regardless of diet composition [245, 246]. Physical activity coupled with a low-carbohydrate diet as well as prolonged fasting are potential non-pharmacological strategies to achieve transient or chronic reduction in plasma glutamine, respectively [247–250].

### Supplementation of medium-chain triglycerides (MCTs) and exogenous ketone bodies

MCTs (particularly C8 caprylic acid) are a type of dietary fat that can be supplemented to potentiate liver ketogenesis [251]. MCTs bypass normal fat digestion and diffuse across the intestinal membrane into the hepatic capillary bed, where they are readily converted into ketones. Mild gastrointestinal side effects may arise during the initial weeks of supplementation, with tolerance improving through gradual dose escalation [252–254]. KDs with supplemental MCTs typically pre-specify a set daily intake (e.g., 2–8 tbsp, or 10–30% of total daily calories in the form of MCTs), which is intended to improve ketonemia but also to lower other sources of fat, simplifying trial design and improving adherence [222, 255–257]. A possible drawback of a diet enriched in purified MCTs, as opposed to naturally occurring high-fat foods, is that they are comparatively devoid of micronutrients, particularly liposoluble vitamins. For this reason, overall food choices should emphasize micronutrient density [258–261], especially if the baseline KD is composed exclusively of medical foods that may be missing essential nutrients, or if significant amounts of dietary supplements such as MCTs are needed to achieve specific biological outcomes (e.g., GKI stability or cachexia prevention).

Analogously, exogenous ketone bodies (e.g., ketone esters or ketone salts) are a novel dietary formulation that can be taken orally to temporarily enhance circulating βHB levels [262]. Beyond their bioenergetic role, ketone bodies act as pleiotropic signaling molecules with potential antineoplastic benefits on their own [100, 263–268]. It is unclear, however, whether short-term decreases in the GKI value via supplemental MCTs or exogenous ketones, without a global metabolic transition to therapeutic ketosis by chronic KDs and/or fasting (increased oxidative efficiency of fat-derived metabolites), would retain protective effects against SLP inhibition in normal cells [269]. Elevated blood glucose and ketone levels are typically not found during the natural physiology of calorie restriction or fasting [270]. In the context of dietary KMT, exogenous ketones can also have a measurable impact on glycemic regulation [271–275]. Therefore, supplementation of ketone bodies could be considered to further enhance the therapeutic efficacy of KD/KD-R, particularly under circumstances of reduced compliance, or to reach the higher levels hypothesized to mitigate cancer cachexia [230, 235, 236].

In conclusion, supplementation of MCTs and exogenous ketones can be viewed as a valuable tool to empower patients to modulate their ketonemia, ketogenesis, and gluconeogenesis, without being an absolute requirement. Boosting ketone levels while following a GKI-adjusted KD may be especially useful during the initial adaptation to fasting, lower limits of euglycemia, radiotherapy, hyperbaric medicine, and conventional and adjuvant drug therapies [103, 276–280].

### Flexible protocols and quantifiable criteria of compliance

Beyond the classic KD, several dietary regimens to achieve various degrees of ketosis have been described in clinical studies, including the modified Atkins diet (MAD) (60–65% fat, 25–35% protein, 5–10% carbohydrate) [281]; high-protein KDs (60% fat, 35% protein, 5% carbohydrates) [282]; paleolithic KDs (based on animal fat, meat, and offal with a 2:1 fat:protein ratio) [218, 283]; Mediterranean KDs (< 15% carbohydrates, based on green vegetables, olive oil, fish, and meat) [284, 285]; general, non-otherwise specified KDs (70–80% fat, 10–20% protein, < 10% carbohydrate) [286]; plant-based, low carbohydrate diets (generally not sufficiently ketogenic) [287]; as well as other targeted and cyclical variations, with or without calorie restriction [288]. Intermittent or prolonged water-only fasting can be included regardless of diet composition [145, 289, 290]. The primary differences are in the maximum limit of carbohydrate and protein, the timing of feeding, and intermittent calorie restriction, as well as the underlying food selection to accommodate personal dietary preferences. Nevertheless, diet flexibility should be contingent upon the patient’s individual physiological response. Biological, measurable, and quantifiable effects (not subjective biases or beliefs) will ultimately determine the suitability of the chosen foods. If glycemia/ketonemia, and, by extension, the sustained GKI values are not in the prespecified target zone, the selected diet may not be appropriate for the patient.

Given the flexibility in implementation and interpersonal variability, any prospective KD protocol for cancer therapy should favor unbiased compliance biomarkers (e.g., longitudinal, steady-state GKI), as well as periodic blood markers and surrogate endpoints (e.g., comprehensive metabolic panel, tumor biomarkers, anatomic-metabolic imaging, metabolomics). Critical benchmarks, laboratory tests, and troubleshooting for dietary KMT are presented in Table 1. Any KD protocol, whatever the practical *food* selection may be, should fulfill the following criteria:

**Table 1:** Key criteria for GKI-adjusted KD/KD-R implementation and troubleshooting

Implementation criteria	Troubleshooting
GKI ≤ 2.0, ideally ≤ 1.0, with absolute glucose levels < 90 mg/dl (5 mM)	A knowledgeable dietitian should be available to assist patients in optimizing biological changes (e.g., maintaining deep ketosis), beyond ensuring diet compliance. Reduce total carbohydrate intake to less than 5% of total calories, replace carbohydrate with fat sources. Review food tracking to detect hidden carbohydrate sources or ingredients that may impact glycemia even if present in small amounts that only minimally change the food nutrition label. Pay attention to hidden carbohydrates in dietary supplements and excipients, as well as cephalic insulin secretagogues due to sweet taste stimulus, such as “keto-friendly” zero-calorie sweeteners (e.g., xylitol, erythritol, allulose, stevia). If necessary and feasible, reduce total caloric intake (KD-R), e.g., reduce calories by 5% increments each week until GKI goals are met. Implement intermittent fasting (e.g., 16:8 or 20:4), alternate-day fasting (ADF), fasting-mimicking diet (FMD) or prolonged water-only fasting. High fiber KDs can hinder therapeutic GKI targets. An excess of dietary fiber can reduce stomach acidity, thereby decreasing cholecystokinin stimulated bile synthesis and excretion into the small intestine, which could lead to reduced essential fatty acid and fat-soluble vitamin uptake [291]. Real-time monitoring via CGM/CKM coupled with event logging can detect sources of variability, such as cortisol, sleep disturbances or pharmacological interference (e.g., corticosteroids). Medium chain triglycerides (MCTs) are more ketogenic than long-chain fatty acids and can be incorporated into meals to increase ketosis. Ketone supplements can provide a temporary boost in ketonemia but are not a substitute for nutritional ketosis. Consider the impact of physical activity, stress management and circadian rhythms. To facilitate GKI targets, evaluate the suitability of gluconeogenesis and glycolysis-targeting drugs (*Additional File 5: Table S2*)
Preserve LBM, recover fat stores if gradually depleted. Weight (fat) loss during KMT should be controlled and therapeutic, improving general and metabolic health	Optimize protein quality and quantity, especially during chronic calorie restriction or higher levels of physical activity. Protein intake in patients with cancer and normal kidney function is typically set above 1 g/kg/day and, if possible, over 1.5 g/kg/day [216]. Impact on GKI of higher protein targets should be monitored. It is important to account for the protein content in natural food sources; for instance, fattier cuts of meat tend to have slightly lower protein compared to lean cuts. Total fat intake will be variable to meet satiety and/or protocol (e.g., caloric restriction). Cyclical caloric surplus (from fat and protein, not carbohydrate), while maintaining ketosis, can mitigate unintentional weight loss. Advanced disease driving tumor-host pro-catabolic effects and intensive treatment can aggravate pathological weight loss (muscle wasting) that must be differentiated from dietary KMT
Adequate micronutrient intake, avoid initial symptoms of keto-adaptation (“keto flu”) and potential long-term side effects. Baseline assessment, blood panel analysis, and monitoring of health status	Metabolic adaptation can be facilitated by a gradual decrease in carbohydrate intake (e.g., change meal composition and frequency in stages until all meals conform to ketogenic ratios) [292, 293]. Assess micronutrient status if food choices are not sufficient to reach the recommended levels of essential nutrients: supplement as needed. Assess electrolyte balance: supplement as needed. Ensure adequate hydration. Consider supplementation to prevent both common and rare side effects, such as constipation (fiber and non-digestible carbohydrates, when necessary), hypocarnitinemia (L-carnitine) or nephrolithiasis (e.g., potassium citrate) [294–296]. Ensure adequate protein intake, as long-term side effects can develop due to excessive protein restriction. Monitoring should be adjusted to the demands of SOC and KMT interventions. Suggested laboratory testing (not prescriptive nor exhaustive): hepatic/renal function (including cystatin C), hemogram, glucose and insulin homeostasis (e.g., HbA1c, fructosamine, HOMA-IR, IGF-1 and binding proteins, c-peptide), hormone testing (e.g., vitamin D, thyroid, glucagon, osteocalcin), inflammation markers and lipid panel, including triglycerides, LDL particle size, apoB100/apoA-I ratio and lipoprotein (a) (traditional cholesterol surrogate markers might not be applicable for patients following KDs) [297–300]. Oral glucose tolerance tests (OGTT) with glucose, insulin and c-peptide sampling can be informative to define the insulin-response phenotype in healthy individuals but may not be advisable in the context of sustained dietary KMT and active cancer [195, 301]

Allow for a sustained GKI of 2.0 or below, ideally 1.0 or below. This involves the lowest physiologically achievable absolute glucose levels (ideally less than 5 mM or 90 mg/dl), minimal glycemic variability (difference between the highest and lowest glucose level), as well as reduced insulin signaling and the related growth-promoting and energy-sensing pathways (e.g., PI3K, mTOR). Glucose and βHB are expected to fluctuate depending on carbohydrate and calorie restriction, as well as fasting duration, protein intake, drug therapies, hormonal balance, emotional stress, circadian rhythms, and nutritional status (vitamin and mineral sufficiency). Reaching the proposed GKI targets implicitly translates into lowering carbohydrate to < 20–50 g/day, regardless of ketogenic ratios or macronutrient percentages, unless concurring with a high level of physical activity [302]. Technologies such as continuous glucose monitoring (CGM) and continuous ketone monitoring (CKM) should be leveraged during the learning phase as the patient explores the impact of different foods on GKI variability [303–305]; it should be noted that CKM sensors are currently available as non-medical devices [306], while clinical testing of dual glucose-ketone monitoring systems is underway [306–308].

1. Allow for a sustained GKI of 2.0 or below, ideally 1.0 or below. This involves the lowest physiologically achievable absolute glucose levels (ideally less than 5 mM or 90 mg/dl), minimal glycemic variability (difference between the highest and lowest glucose level), as well as reduced insulin signaling and the related growth-promoting and energy-sensing pathways (e.g., PI3K, mTOR). Glucose and βHB are expected to fluctuate depending on carbohydrate and calorie restriction, as well as fasting duration, protein intake, drug therapies, hormonal balance, emotional stress, circadian rhythms, and nutritional status (vitamin and mineral sufficiency). Reaching the proposed GKI targets implicitly translates into lowering carbohydrate to < 20–50 g/day, regardless of ketogenic ratios or macronutrient percentages, unless concurring with a high level of physical activity [302]. Technologies such as continuous glucose monitoring (CGM) and continuous ketone monitoring (CKM) should be leveraged during the learning phase as the patient explores the impact of different foods on GKI variability [303–305]; it should be noted that CKM sensors are currently available as non-medical devices [306], while clinical testing of dual glucose-ketone monitoring systems is underway [306–308].
2. Patients are often faced with uncertainty regarding “optimal” GKI targets that would be safe and physiologically attainable, depending on their evolving disease status and concomitant therapies. Two empirical GKI baselines can be determined to serve as idiosyncratic biological reference points. Once completing the initial ketogenic adaptation via dietary modification, a fasting GKI baseline can be measured after at least 72 h of water-only fasting (e.g., days 4 to 7 of a 5–7-day water-only fast), which produces GKI values unaffected by dietary inputs [145]. A zero carbohydrate, paleolithic KD with intermittent fasting (e.g., one meal per day) can provide a second baseline that is representative of the lowest GKI variability during minimal dietary inputs (fat and protein only, in a compressed feeding window) [134, 218, 283, 309]. The influence of preexisting conditions, such as insulin resistance, can be captured with a repeated measures design. All subsequent diet adjustments can be compared to these two benchmarks. During study planning and subsequent data analysis, diet flexibility should not compromise GKI targets: “biological” compliance outweighs self-reported or perceived “dietary” compliance.
3. Adequate protein intake to maintain LBM without disrupting GKI, starting at 0.8 g/kg of body weight and typically settling between 1.2 and 1.5 g/kg for most individuals [310]. Higher initial targets are justified in certain patient demographics (e.g., older age), preexisting comorbidities or anticipated negative impacts of the cancer diagnosis (e.g., loss of appetite during active cancer treatment, or limited physical activity due to cancer fatigue) [311]. Protein quality should be a focus to ensure adequate amino acid ratios without forcing protein overconsumption [312].
4. Changes in LBM should be monitored on a regular basis. Patients at borderline low weight or with insufficient LBM may alternate between KD-R and GKI-adjusted eucaloric/surplus intervals to preserve and rebuild muscle tissue. Although the systemic metabolic alterations induced by tumor-derived factors secreted directly by GBM cells are still under study, functional impairment leading to undernutrition and side effects of treatment may contribute to progressive loss of skeletal muscle [313–316]. Importantly, irreversible or accelerated cachexia has not been reported in clinical trials examining KDs across several cancer subtypes (despite variable reductions in fat mass), but underweight patients were often excluded a priori, and most studies were designed to prevent weight loss by minimizing calorie restriction [221, 231, 232, 317]. It will be important to examine the impact of well-formulated KDs on cancer-related cachexia in the clinic, ensuring adequate nutrition and protein sufficiency while managing its multifactorial origins, such as systemic inflammation and endocrine dysregulation, which may be difficult to capture in preclinical models [236, 318–321]. Off-label and research-phase anti-catabolic agents, anti-inflammatory drugs, and appetite regulators can synergize with exercise and nutrition therapy to prevent muscle wasting [322, 323].
5. Adequate micronutrient and vitamin intake. It is preferable to obtain all dietary elements from nutrient-dense foods (e.g., eggs, beef, oily fish, offal) [324]. If the included foods cannot maintain adequate levels of certain essential nutrients or minerals, specific multivitamin and mineral supplementation is warranted [325]. Monitor for secondary hypocarnitinemia and supplement if needed [326]. Macronutrient and micronutrient tracking can be simplified using diet-tracking software [327, 328].

## Lessons learned from clinical research evaluating KDs for GBM

Large-scale clinical integration of precision nutrition for cancer management still poses a challenge, with no consensus on best practices [290, 329]. Consequently, patients tend to freely choose their dietary plan [330, 331]. KMT is a potential biomarker-driven metabolic therapy to lower glycolytic SLP, insulin, and oncogenic signaling below baseline, while also stabilizing the tumor microenvironment, contingent upon biological compliance and impacts from other therapies [332, 333]. Additional File 3: Table S1 provides relevant examples of realistically achievable glucose and βHB values that have been reported in studies examining KDs in high-grade brain tumors; additional cancer subtypes have been discussed in [290, 334–336].

Concerns have been raised about the feasibility of reaching and sustaining the hypothetical therapeutic window of KMT, suggesting that “dietary-induced hypoglycemia as a treatment for brain tumors may be simplistic” [222]. While a high degree of personal motivation, specific domain knowledge, and (typically) the assistance of a KD-trained professional is indeed critical for strict diet adherence, more research is needed to establish causal links between quantifiable metabolic changes and therapeutic outcomes, as well as synergistic pharmacological interventions to enhance efficacy [337]. Unfortunately, most GBM studies investigating KDs have not consistently tracked glycemia/ketonemia or other biochemical parameters across time (e.g., serial metabolic imaging or metabolomic profiling), thus patient stratification based on total cumulative exposure to different ranges cannot be performed [153]. A minority of studies documented daily glucose and ketone readings at different non-standardized endpoints but did not report raw data. To embody the goals of precision nutrition, future clinical studies will have to measure, report, and analyze biological responses separately for each patient, regardless of outcome, avoiding group averages [338, 339].

## Common pitfalls in clinical research methodology

Clinical studies evaluating KMT may fail due to early oversights in experimental design that can be mitigated with the right knowledge and preparation. Table 2 summarizes recommended and alternative methods for dietary KMT implementation.

- Trials often lack ongoing communication and support to retain participants and reduce non-compliance. Recent technologies such as smartphone monitoring applications, telemedicine, and real-time biofeedback (e.g., CGM/CKM or multi-metabolite sensors) may alleviate this issue. Frequent communication with a dietitian/nutritionist trained in KMT as well as a “research kitchen” may improve adherence (e.g., NCT03451799 and NCT03535701). Tracking and optimization of the desired biological markers should be emphasized over self-reported dietary compliance during nutritional counseling [340].
- It is exceptionally difficult to gain Institutional Review Board (IRB) approval for KMT trials without concurrent chemoradiotherapeutics, even if their contribution to the long-term management of GBM remains limited [7]. Considering the inadvertent consequences on tumor metabolism, it will be important to design GBM trials with at least one KMT intervention arm in which, after surgical debulking, carefully selected components of SOC (e.g., conventional fractionated radiotherapy) will be tentatively delayed for a clinically acceptable period until an interim evaluation of response. Based on predefined outcomes (partial remission or stable disease), SOC would be delayed again until a subsequent evaluation or disease progression. In this paradigm, KMT refers to both dietary and pharmacological targeting of tumor metabolism, as defined in the protocol below, not a generic KD as monotherapy.
- Results from several GBM trials indicate that chemoradiotherapy can be safely delayed for up to 6 weeks after surgery; in some trials, delaying chemoradiotherapy has been paradoxically associated with improved outcomes [341–345]. It is not inconceivable, however, that delaying chemoradiotherapy may have a negative impact on PFS or mOS, despite dubious influence on long-term survival [346–348]. Consequently, well-informed GBM patients should be given the choice to enroll into any prospective group after evaluating the abovementioned survival data (e.g., dietary and pharmacological KMT, or in combination with dose-adjusted temozolomide and/or radiotherapy). Alternatively, patients that are unable or unwilling to undergo some or all aspects of SOC could be offered enrollment in diet-drug KMT trials. In a similar way, the active monitoring period in low-grade gliomas confers an ethical opportunity for the evaluation of non-toxic therapeutic strategies such as dietary KMT, following the recent example of dual inhibitors of mutant IDH1/2 enzymes, which have been tested specifically to “delay the potential long-term toxic effects” of adjuvant chemoradiotherapy [349]. If relative disease stability is achieved despite tumor persistence, repeated surgical debulking could be considered to reduce tumor load [134, 350].
- IRB approval for KMT as monotherapy or KMT with only partial SOC will likely demand frequent metabolic and/or anatomic imaging to ensure safety and ongoing tumor evaluation, with a modifiable treatment plan. Accordingly, in a fixed trial design, no GBM patient would be deprived of the potential benefit of chemoradiotherapy, which would be offered to all patients who request it (see Additional File 4: Figure S1). Clinical evaluation of KMT is ideally suited for adaptive trial designs, such as platform trials with response-adaptive randomization, given that it combines a metabolic priming baseline with additional, elective, synergistic press-pulse therapies that require a flexible implementation, compared to a common control group [351, 352]. A core set of interventions in the form of biomarker-driven dietary and pharmacological KMT could be included in the shared master protocol, but subsequent trial arms would need to be adjusted with experimental or salvage therapies based on pre-defined outcomes during each interim analysis.
- Eligibility and exclusion criteria should consider the functional demands of the interventions to maximize sample size without compromising efficacy. Eligibility considerations include disease status, side effects or sequalae from prior therapies, comorbidities, performance status, organ function, and contraception and pregnancy testing; conversely, exclusion criteria must include the absolute contraindications of KDs, such as rare inborn errors of metabolism [353].
- While well-controlled dietary studies where prepared food is provided to all participants are ideal in terms of diet adherence [209], offering patients to self-select their experimental group and foster self-efficacy may be advantageous in studies where therapeutic outcomes are linked to active participation [354, 355]. Similarly, in a recent KD trial originally planned with two diet arms, patients reported explicit disinterest to participate in the control diet arm (i.e., low-fat treatment) [114]. Therefore, elucidating biological mechanisms and maximal theoretical efficacy of any prospective diet-drug combination may benefit from pilot studies designed in non-randomized, “ideal” scenarios (e.g., self-selected patients with high functional status), proceeding to randomization in the general population after the most promising interventions and biomarker thresholds have been identified.
- Most feasibility and tolerability trials have not aimed for the lowest sustained GKI, instead focusing on diet flexibility to ensure better adherence. Compliance is a major challenge, and the diet needs to be as easy to follow and palatable as possible (e.g., prepared meals, medical foods, enteral feeding), but simplicity should not outweigh biological efficacy even at the conceptual phase. Motivated, well-informed patients should understand that objective biomarkers of compliance (such as cumulative time in specific GKI ranges) may influence therapeutic outcomes [102]. Informed consent should be obtained not due to expected side effects, which are preventable or manageable, but to make patients consciously aware of the importance of active participation. Therefore, patients should be instructed to pursue the lowest possible GKI, beyond the trial’s basic requirements. Moreover, correlations with outcomes should be stratified according to biological readouts rather than dietary compliance.
- Patients are seldom encouraged to reinforce GKI targets after the intervention period, which is generally no longer than 1–3 months due to budgetary constraints. Studies that are limited in time may fail to produce robust results given that achieving stable therapeutic ketosis often encompasses several weeks, and it is unknown whether long-term maintenance impacts the risk of recurrence. If a trend towards improved PFS or mOS is detected, it will be important to weigh the influence of KMT and SOC, which also requires extended follow-up. Ultimately, GKI-adjusted KD/KD-R should be considered a long-term strategy rather than a limited intervention.
- Randomized trials assign patients to the KD intervention while maintaining “usual” (preferred) diet in the control group; a higher demand is consequently placed on the intervention group, especially given the overwhelming physical, emotional, and financial burdens that accompany a cancer diagnosis [356, 357]. Sufficient guidance and understanding of the scientific rationale are therefore essential for patient accrual, compliance, and optimization of therapeutic outcomes. As dietary KMT has been associated with improvements in quality of life and self-efficacy across a broad range of cancers [197, 221, 358–361], it will become important to develop insurance models and healthcare policies that facilitate access and minimize out-of-pocket costs [362].
- KMT is often tested in smaller, single-center, investigator-initiated trials. Given that researchers proposing such trials may feel it would be unethical to exclude any potential participants, patients unable to keep GKI or predefined surrogate markers in specific ranges could be used as internal controls. Contemporaneous external controls (from the post-Stupp era) are also a consistent source of comparative survival data [23, 363, 364]. Understandably, dietary KMT studies of sufficient length cannot be easily double-blinded or placebo-controlled.
- Individual case reports are highly heterogeneous and lack statistical power, even though they may be a more appropriate methodology for personalized medicine [365]. Case reports should be written following systematic reporting guidelines, such as the CARE guidelines [366, 367]. In contrast, larger clinical trials will require a multi-disciplinary team capable of tracking and supporting each patient individually. GKI allows for quantitative comparisons across different cohorts and types of cancer, but the dietary and/or pharmacological interventions to achieve GKI targets should be personalized.

**Table 2:** Recommended and alternative methods for GKI-adjusted KD/KD-R implementation

Recommended methods	Alternative methods
Instruct patients to measure blood glucose and βHB at least twice daily: after the overnight fast (morning), and 1–2 h prior to the last meal (evening). Real-time CGM/CKM is the preferred method for data collection. In future clinical research, it will be essential to track glucose/ketones continuously in order to stratify patients according to time spent in discrete GKI ranges. CGM/CKM can be validated via finger-prick sampling, reducing testing burden. If only CGM is available, it can be coupled with capillary ketone monitoring due to lower general variability in ketonemia. Pre-specified GKI targets should be considered primary trial endpoints	Measure GKI just once daily (ideally in the pre-prandial evening period, after the overnight fast, or prior to the first meal if practicing intermittent fasting, maintaining consistency across measurements). Urinary ketones are not accurate for GKI calculation but can inform sufficient carbohydrate restriction during diet initiation (first 2 weeks) [332]. Testing burden can be reduced after attaining diet stability (i.e., food selection remains unchanged), unless in a clinical trial. For long-term diet maintenance, glucose levels can be inversely correlated with ketosis: if carbohydrates are sufficiently restricted, maintaining a stable lower range of euglycemia (e.g., ≈ 60 mg/dl) is likely accompanied by higher ketonemia
KD/KD-R should be assigned depending on initial weight and estimated fat mass. Patients can incorporate fasting in a cyclical manner, as dictated by their adipose tissue reserves. For example, patients with sufficient fat mass can implement a 3–7-day water-only fast or fasting mimicking diets (FMDs) every 1–2 months [368]. Obese/overweight patients can extend fasting beyond 7 days under medical supervision. An average, temporary weight loss of ≈ 3 kg can be expected after a 3-day fast, increasing to ≈ 8 kg after 20 days [369]. A significant fraction of this weight comprises glycogen-associated water storage and intestinal contents, which are quickly recovered [370, 371]. Under proper implementation, LBM reduction is minimal, and most fat mass is subsequently regained [371, 372]. Gradual adipose tissue recovery is feasible if the caloric density of a low carbohydrate diet is sufficient [373, 374]. Fasting should not be limited or discouraged unless there is a risk of cancer cachexia, but each fasting period should be planned and supervised. Inexpensive and non-invasive methods such as bio-electrical impedance can be used to track approximate changes in fat and LBM over time within the same individual	Underweight patients should not practice prolonged fasting without alternating cycles of weight recovery. Loss of LBM should be avoided. A trained dietitian should advise patients on implementing a small caloric surplus with resistance/strength training to recover muscle and fat mass after fasting or KD-R intervals, while still adhering to GKI targets. If body fat stores are too low, it may be preferable to avoid fasting and focus on GKI while maintaining an isocaloric diet. Ongoing changes in average weight (over several weeks) will dictate whether the diet is effectively calorically restricted, eucaloric or hypercaloric, regardless of self-reported or estimated caloric intake. Safety and contraindications of water-only fasting are discussed in [375]; special attention should be given to electrolyte balance and refeeding. If the patient presents with low fat mass (but normal weight/BMI), a FMD may be considered to avoid protein breakdown while potentiating therapeutic GKI ranges [376]
In treatment-naïve GBM patients, dietary KMT can be initiated as a neoadjuvant strategy with the aim of reducing tumor growth rates. In the absence of life-threatening symptoms, after surgery, select elements of SOC that may be antagonistic could be preemptively scheduled but delayed until completing a standalone KMT period (including both dietary and pharmacological targeting, as defined in the treatment timeline), for a conservative maximum of 6 weeks. If sufficient radiologic responses or disease stability can be confirmed, radiotherapy and/or chemotherapy can be delayed again while intensifying KMT, for no more than 6 weeks, and reevaluated periodically as long as regression or stability are maintained. During SOC delay, it may be essential to perform sequential imaging to corroborate metabolic responses (see Additional File 4: Figure S1)	This “if/then” experimental design would be applicable to histologically and molecularly confirmed GBM (before or after debulking surgery), given that delaying chemoradiotherapy for up to 6 weeks has shown little to no impact on PFS and mOS (when no other treatment was given, representing a window of opportunity to institute KMT) [341–346]. Outside clinical trials, patients are encouraged to discuss survival data regarding SOC initiation with their treating physician [377]. It should be noted that the long-term survival of GBM with current SOC is less than 10% at 5 years, independent of timing or dosing schedule. Follow-up with imaging and bloodwork should be provided to all patients regardless of their desired treatment, preventing patient abandonment [378, 379]
Patients will be asked for informed consent after they receive education as to how dietary KMT will be administered as a therapy, including how non-compliance could have a detrimental impact on the expected benefits. Follow-up should be frequent enough to detect early trends in tumor progression. Researchers should have flexibility in trial design to react to this eventuality, intensifying SOC, metabolic inhibition, or microenvironment targeting	Even though strict adherence to the diet, biomarkers and treatment protocol is necessary, some flexibility should be offered in the timeline of diet-drug implementation. Patients that require second-line salvage therapies not previously defined in the trial design could be reported as individual cases in more heterogeneous cohorts

## Patient education and data collection

After enrollment, each participant should be provided with the following:

- *Description and informed consent for the proposed therapies*. Dietary and pharmacological KMT could include a combination of GKI-adjusted KDs (with or without caloric restriction), fasting, and drug/adjuvant therapies (e.g., metabolic inhibitors, drug repurposing, investigational compounds, hyperbaric medicine, hyperthermia, photo/sonodynamic approaches), with elective and protractible 6-week delay of chemotherapy and/or radiotherapy prior to image-based reevaluation. Dietary KMT implementation requires some level of scientific literacy and active participation. Well-informed patients will be ultimately responsible for dietary compliance, and for requesting support if they are unable to meet biomarker targets. Patient education is key to fostering motivation and biological efficacy. Working with a dietitian/nutritionist knowledgeable in the initiation and maintenance of KMT is extremely helpful.
- *Tools to monitor blood glucose and ketones*. Patients should be counseled on how to use finger-prick glucose/ketone meters, initially measuring at least twice a day to capture data variability. GKI tracking allows patients to actively engage in the treatment process, which could improve compliance. Researchers can expand this testing schedule; for example, 1–2 h post-meal when new foods or changes in portion size are introduced. Patients should also have leeway to reduce the testing burden after the end of the trial, eventually measuring only a few days per week if food selection remains unchanged. Depending on the trial budget, real-time CGM ± CKM is preferred, delivering more robust data collection and biofeedback [304, 305]. Longitudinal tracking should be emphasized to allow for correlations with long-term outcomes. Urine acetone strips (urinary ketones) and breath acetone analyzers are often poorly correlated with blood ketone levels and thus discouraged in research settings; however, they can be useful for outpatient self-tracking, when verified by gold-standard testing methods [204, 380, 381].
- *Preapproved food lists, meal templates, sample meal plans, and recipes to streamline macronutrient tracking*. Patients and caregivers will be expected to keep food records, adhere to templates, or use diet tracking software, especially during the diet transition phase [327]. A photographic diary may help with logging and data sharing, with the added benefit of time-stamping the feeding schedule. In the future, image-based food recognition algorithms could reduce logging efforts [382, 383]. There may be circumstances, however, where short-term adherence to simplified lists of “allowed/excluded” foods could suffice, if GKI and biomarker targets are reached.
- *The healthcare staff should be prepared to answer general questions and help with diet implementation*. Routine follow-up and troubleshooting sessions are recommended, particularly during the adaptation period (e.g., first appointment within 2 weeks to ensure biological endpoints are met). Compliance and motivation can be significantly improved when patients are held accountable via external monitoring, coaching, and remote care [384, 385]. In contrast, compliance may be compromised if disagreements exist between family members or external healthcare providers regarding the suitability of the treatment plan. Reaching consensus is encouraged, with examination of the scientific literature to resolve any questions regarding the rationale and expected outcomes of all proposed therapies, including SOC. General and disease-specific educational resources to implement long-term KD plans are available for both patients and clinicians [208, 311, 334, 386–390]. Given the wide access to low-carbohydrate recipe books for general audiences, it is important to reiterate that the suitability of the chosen plan should be determined by monitoring the lasting induction of the desired biological outcomes (e.g., sustained GKI or biomarker targets, such as insulin suppression), rather than any particular set of *food* recommendations, irrespective of the goal or medical condition they were originally designed for (e.g., weight loss, epilepsy, diabetes mellitus).
- Appropriate psychological and emotional support, with individual or group counseling [391].

## Key steps in the treatment timeline

All prospective participants should undergo a baseline evaluation before they are considered for KMT, including medical history, nutritional and anthropometric assessment, bloodwork, and anatomical/metabolic imaging; this is particularly relevant before pharmacological or systemic interventions, which may not be adequate for all patients. Psychological and neurocognitive health often suffers greatly after a GBM diagnosis, which can impact the ability of patients to follow treatments which require active participation [392]. Therefore, it is also important to assess if a proactive approach based on health ownership reduces morbidity and improves quality of life [393].

**Fig. 3:**
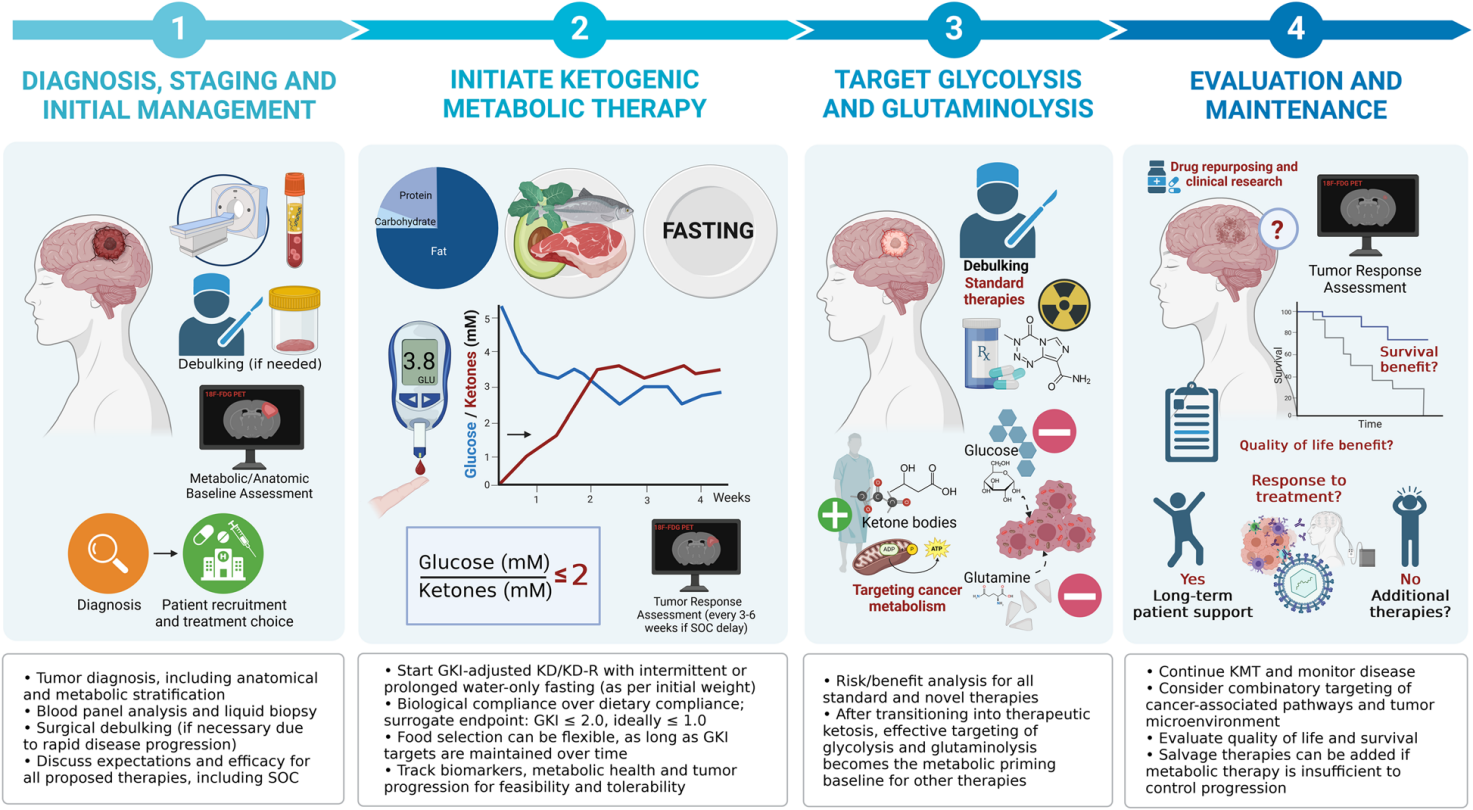
Overview of KMT implementation in high-grade glioma research, including both dietary KMT (GKI-adjusted KD/KD-R and fasting, aimed at increasing chronic metabolic pressure on cancer cells while favoring OXPHOS metabolism in normal tissues), as well as pharmacological KMT (targeting of glycolysis and glutaminolysis in a press-pulse design, in addition to cancer-associated pathways to normalize the tumor microenvironment)

A chronological timeline is crucial for record-keeping and establishing associations between procedures and therapeutic outcomes or side effects. Figure 3 provides an overview of KMT for high-grade glioma and Table 3 summarizes key steps in the suggested clinical implementation of dietary and pharmacological KMT. The steps in this timeline are based on the “press-pulse” therapeutic strategy [132]. GKI-adjusted KD/KD-R and fasting are implemented as a metabolic “press” to restrict fermentable fuels, reduce inflammation, and normalize the tumor microenvironment, while drugs that simultaneously target glycolysis, glutaminolysis, and other cancer-associated pathways are defined as either “press” or “pulse” interventions, depending on pharmacodynamics and safety. Representative diet-drug combinations have been presented in case reports and pilot clinical trials, although most studies thus far emphasized feasibility and additivity with SOC, rather than integrating dietary and pharmacological KMT as a prerequisite, continuous, biomarker-driven “metabolic priming” baseline [103, 107, 109, 134, 279, 394–397].

**Table 3:** Recommended timeline for dietary and pharmacological KMT research in GBM

Key steps in the clinical implementation of KMT	Overview
1. Tumor diagnosis and shared decision-making	Brain tumor pathology is the gold standard for differential diagnosis. The preliminary diagnosis can be made via non-invasive techniques, as applicable (CT, MRI, PET; liquid biopsy can be informative in extra-neural tumors but has not been validated for GBM). If maximal surgical resection is feasible, histopathologic and molecular characterization can be performed after surgical debulking. Initial tissue biopsy in tumors suitable for later subtotal or complete resection is more valuable when it provides actionable therapeutic guidance; otherwise, final histopathological analysis can be obtained from surgical samples. Patients are encouraged to request detailed, written documentation regarding expected SOC outcomes (long-term survival and quality of life) to facilitate informed consent
2. Blood panel analysis	Evaluation of basal health status will determine the best therapeutic approaches, as well as feasibility of KMT and chemoradiotherapy. It is advisable to formulate a well-defined schedule for tumor evaluation. In GBM, radiologic imaging is necessary due to lack of reliable blood-based biomarkers
3. Metabolic stratification	Previously established bioenergetic phenotypes can be used as estimates to guide metabolic therapy. Glucose and glutamine are universally recognized as necessary for tumor growth, regardless of utilization pathway. In a research context, metabolite flux and substrate dependency of tumor samples can be assessed using in vitro/ex vivo bench top and in vivo metabolic imaging techniques. In the case of chemo/radiotherapy delay, clinical trials may require baseline and serial anatomic/metabolic imaging (e.g., every 3–6 weeks) to corroborate favorable responses
4. Surgical debulking	The extent of surgical resection is one of the strongest prognostic factors in GBM. If presenting with life-threatening symptoms, surgery should be performed as soon as possible, ideally with intraoperative fluorescence mapping (e.g., 5-ALA or novel research-phase metabolic markers). In the absence of such symptoms, a short watchful waiting period with dietary KMT initiation (GKI-adjusted KD/fasting) could be considered prior to debulking, potentially facilitating better surgical delineation
5. Initiate dietary KMT	KMT can be initiated before or after surgery. Self-reported dietary compliance and/or food records are not reliable sources of data in research settings. Instead, cumulative GKI ranges, or similar unbiased biological compliance markers, should be predefined as primary or surrogate study endpoints. Patients with good general health (as per clinical history, bloodwork, and stratification of disease) and sufficient body fat mass may initiate KMT with prolonged water-only fasting (≥ 3–5 days), which generally achieves GKI ≤ 2.0 during the fasting interval. Alternatively, it is possible to gradually transition to a GKI-adjusted KD/KD-R, which should be maintained as long as the tumor persists. Patients with sufficient body fat reserves may consider longer fasting periods (> 1–3 weeks), which are safe under medical supervision and will provide longer cumulative exposure to reduced GKI as well as autophagic effects
6. Radiation therapy	For the clinical testing of combined dietary and pharmacological KMT, given the conflicting metabolic consequences of brain-directed radiation, a request to modify the timing of radiotherapy may be proposed by the investigators: late-stage adverse effects should be weighed against short-term anti-tumoral or synergistic benefits [11, 73, 75]. For IRB approval, radiotherapy could be conditionally delayed or used at low-dose regimens until signs of disease progression. During informed consent, patients should inquire about the absolute survival increase provided by radiotherapy along with the possible short- and long-term side effects. If the patient chooses a multimodal approach, dietary KMT may provide radiosensitizing potentiation, although some benefits may be blunted by radiation-induced destabilization of the tumor microenvironment and concomitant steroid administration. Untargeted radiation techniques should be avoided in all cases
7. Drug treatments	Ideally, all drug treatments (especially metabolic inhibitors and chemotherapy) should be administered in a GKI ≤ 2.0 range. Corticosteroids should be used only when unavoidable, at the minimum effective dose, and frequently reassessed with the goal of de-escalation as soon as clinically feasible. After achieving a therapeutic GKI-adjusted baseline, glycolysis and glutaminolysis should be targeted simultaneously; various diet-drug combinations can be evaluated to this effect (e.g., targeting insulin signaling via repurposed drugs such as metformin or SGLT2 inhibitors, or direct metabolic inhibition using research-phase glucose and glutamine antagonists). Further stabilization of the tumor microenvironment or targeting of cancer-associated pathways, including immune-based therapies, can be initiated after the effective restriction of SLP metabolism while increasing whole-body adaptation to nutritional ketosis
8. Physical activity	An individualized dose of physical activity that promotes long-term muscle maintenance and aerobic fitness is recommended. The type of exercise should be based on the patient’s training experience and current exercise capacity. Resistance training preserves muscle mass, increasing a major physiological glucose sink, further enhancing ketogenesis through decreased insulin secretion
9. Stress management	Given that active participation and motivation is required for successful KMT implementation, patients should receive adequate psychological and emotional support to enhance quality of life, reduce non-compliance, and avoid stress-related therapy excursions
10. Evaluation of outcomes and therapy adjustments	Radiologic imaging should be performed within 2–8 weeks of KMT initiation. Current guidelines recommend brain imaging every 2–4 months during the first 3 years from diagnosis [26]. More frequent reevaluation may be necessary in clinical trials of combined diet-drug KMT, especially if proposing changes to the SOC schedule. Treatment can be adjusted in the event of tumor progression. Repeated surgical debulking can be planned for slow growing tumors

**Fig. 4:**
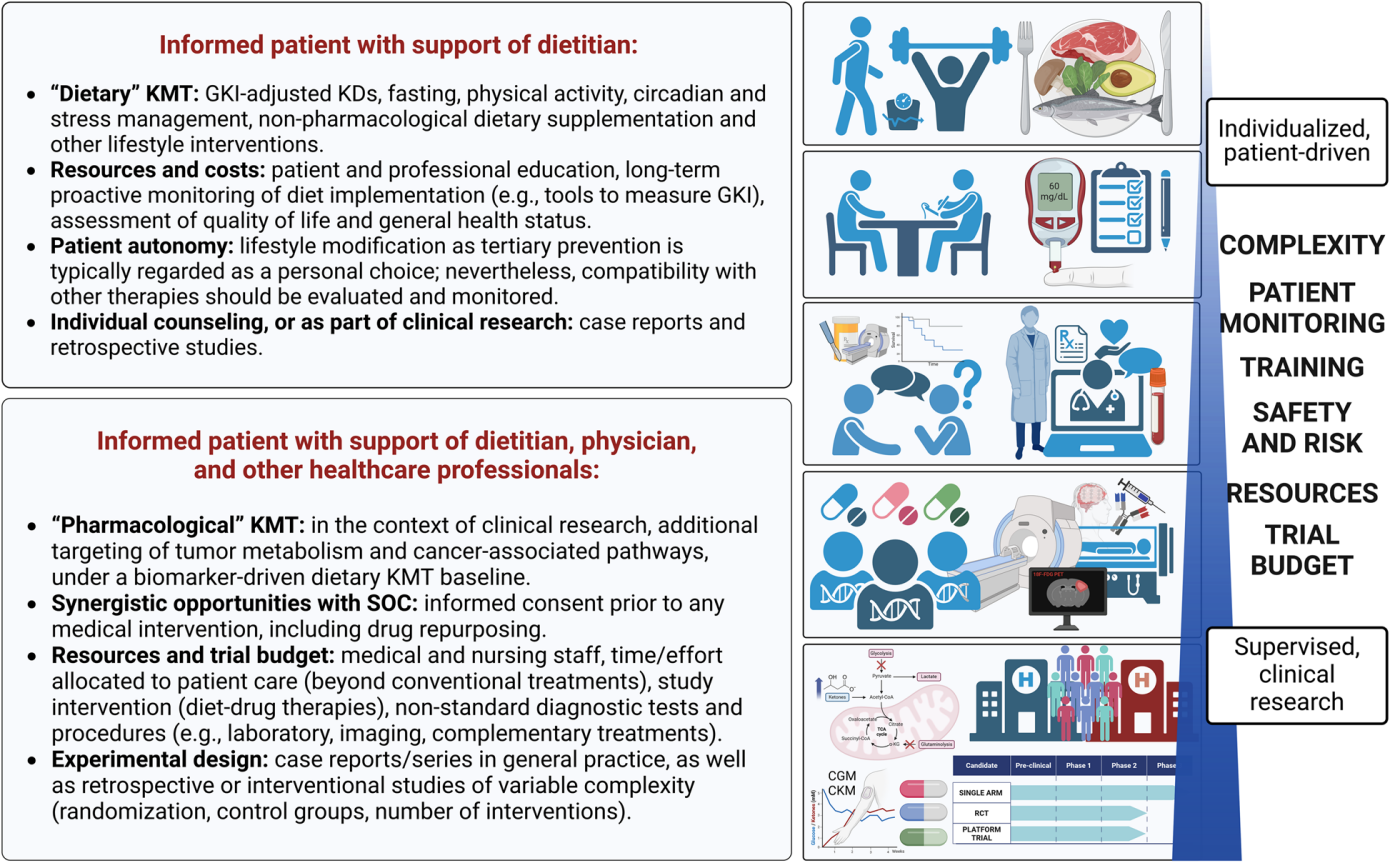
Prerequisites and potential experimental complexity of KMT. Any interested patient can initiate dietary KMT, ideally under the supervision of a trained dietitian. The resources and staff required for pharmacological KMT are dependent on the number of interventions and clinical settings (for example, a GKI-adjusted KD in addition to SOC, or research therapies such as glutaminolysis inhibition)

The suggested KMT framework is constructed in a modular fashion, with intrinsic flexibility during both routine clinical application and research design. It is not expected that all steps of the timeline, such as the testing of diverse diet-drug combinations, will be implemented by a single research institution. Understandably, the potential costs and resources will vary greatly depending on the number and complexity of the proposed interventions (e.g., from patient education and monitoring by a single dietitian, up to a multi-arm, multi-site platform clinical trial). As described in Fig. 4, the basic requirements of KMT can be adjusted to fit various clinical contexts. Even though adjustments to nutrition and over-the-counter supplementation are within the patient’s purview, the use of prescription medication involves the cooperation of a physician trained in metabolic oncology. Lack of familiarity, inertia of prescribing habits and fear of legal vulnerability can be reasons for not pursuing off-label use within standard practice [398–400]. Recently, educational resources and training programs have emerged to address these barriers [386, 401]. In research settings, any therapy beyond SOC must be presented in concordance with local deontological guidelines, with IRB-approval and informed consent. Outside research, dietary KMT (KD/KD-R and fasting) can be implemented freely by the patient, but active drug repurposing or compassionate use (as allowed by local regulations) requires a clear rationale and informed consent [402–404]. In the latter case, the primary goals should be safety, quality of life, and improved therapeutic outcomes, with emphasis on reporting the collected data to applicable regulating bodies (under certain compassionate use programs) as well as the broader scientific community [403, 405–407].

### Tumor diagnosis and shared decision-making

It is preferable, if possible, to make the initial *tentative* diagnosis using non-invasive neuroimaging techniques (CT, MRI, PET) to avoid the risk of exacerbating tumor growth or iatrogenic cell dissemination through inflammatory oncotaxis [408–411]. Liquid biopsy can be informative for diagnosis and disease monitoring in extra-neural cancers but has not been sufficiently validated in GBM [412]. Initial tissue biopsy prior to surgery would be more applicable to cases where it can provide actionable information (that is, when histological, molecular, or metabolic characterization dictates subsequent therapies, beyond simple staging). In tumors suitable for maximal safe resection, histopathological and molecular analysis after maximal debulking would be preferred to fine-needle biopsy, serving as the gold-standard for differential diagnosis [413, 414]. Patients should be informed about the risk/benefit of contrast agents such as gadolinium and iodinated contrast media [415].

There has been increased emphasis on early cancer detection, including direct-to-consumer liquid biopsy and diagnostic imaging, but lacking consensus regarding dubious findings [416–418]. When facing benign or slow-growing tumors with conservative management [419, 420], patients may decide to request information about dietary KMT during active surveillance. It is important to mention that, despite mechanistic rationale and potential normalization of risk factors such as inflammation and metabolic syndrome [421–423], we are not aware of any longitudinal clinical trial exploring whether KDs and/or cyclical fasting could reduce the risk of high-grade transformation. Primary and tertiary cancer prevention have been longstanding targets for dietary modification [424, 425]. However, we recognize that proposing such interventional trials may be challenging due to lengthy follow-up periods, and thus encourage retrospective studies on patients that have chosen to follow long-term ketogenic lifestyles on their own [203].

After the most probable diagnosis has been determined, patients should be offered a follow-up consultation to explore treatment options and analyze expected outcomes. This step is applicable to all types of cancer. In ideal circumstances, an empathetic conversation should take place to fully inform the patient, as part of the shared decision-making process. Unfortunately, despite recent progress in patient-centered care, cancer treatments are still frequently delivered in a paternalistic framework, with poor communication about expected outcomes and reasonable alternative treatments [330, 426, 427]. To aid with informed consent, patients are encouraged to request written documentation about the estimated efficacy of conventional therapies, given that their high degree of protocolization allows for a relatively accurate calibration of expectations [428]. Ambiguous or ill-defined verbal descriptions of expected SOC outcomes are unacceptable. In our view, informed consent is only possible when the necessary time has been devoted to defining the long-term survival rates (not PFS or mOS) from applicable contemporary clinical trials that serve as the basis for SOC guidelines. This poses ethical considerations about the duty to inform and the “right not to know,” but also facilitates reaching consensus and allows the patient to judge the need for emerging/research therapies, off-label, or compassionate use, at different levels of the evidence-based pyramid [403, 404, 429, 430].

### Blood panel analysis

It is important to analyze all relevant blood elements to establish a comparative baseline before KMT and surgery. As described in Table 1, suggested laboratory tests and monitoring frequency should be adjusted to the demands of the proposed interventions (e.g., diet-drug combinations). Biomarkers of general health, including hemogram, electrolytes, hepatic and renal function, inflammation, and vitamin status, should be within or near the normal range before initiating therapeutic measures that carry a risk of adverse effects. Key dietary nutrients to monitor on a carbohydrate-restricted diet include carnitine, thiamine, folate, pantothenic acid, calcium, phosphorus, iron, vitamin D, and trace minerals [431]. KDs, partial (“fasting-mimicking”), and/or water-only fasting can improve preexisting lifestyle-related blood panel abnormalities, particularly hyperinsulinemia, insulin resistance, and chronic inflammation [221, 368, 432].

GBM is generally not amenable for estimation of tumor burden using blood-based biomarkers due to the blood–brain barrier, which is only partially disrupted in most cases [433]. A small number of circulating proteins, extracellular vesicles, tumor cells, and DNA/RNA fragments in blood and CSF have been proposed for diagnosis and follow-up [412, 434–436]. However, until these methods are fully validated, imaging techniques remain the gold-standard for response and recurrence evaluation [437, 438]. In extra-neural cancers, a combination of traditional protein-based tumor markers, circulating tumor cells, DNA/RNA fragments, and imaging modalities (including ultrasound and infrared thermography) can be leveraged to track disease progression [439–441].

A transient elevation of circulating tumor markers during systemic therapy (a phenomenon known as “spiking”) could be misinterpreted as progressive disease [442–447]. It is therefore essential to correlate tumor markers with other clinical and radiological parameters, especially if the patient opts for active monitoring with standalone dietary and/or pharmacological KMT. Recently, liquid biopsy services have come to market (e.g., circulating tumor cells, including glial cells), which are sometimes requested independently without informing the treating physician; however, due to their novelty, therapeutic decisions should be made in conjunction with radiologic responses, clinical criteria, and additional laboratory testing [448, 449].

### Metabolic stratification

During routine clinical practice, metabolic targeting can be informed by previous molecular and mechanistic characterizations of the tumor subtype: high uptake of glucose and glutamine is considered a common feature of high-grade gliomas, correlating with cell density and aggressiveness, especially in grade 3 and 4 tumors [450–452].

We recommend standardized metabolic imaging of glucose uptake (18F-FDG PET coupled with anatomic imaging) for all GBM patients, which may offer improved staging and delineation of surgical margins [453]. A fasting period (≥ 12 h) is advisable prior to the scan to facilitate 18-FDG transport into the tumor and lower insulin-mediated glucose uptake in the surrounding tissue [454, 455]. It is important to consider glycemia and ketonemia for standardized uptake value (SUV) normalization in patients that have already initiated a KD, as both can influence 18F-FDG uptake in normal brain [456, 457]. IRB approval of clinical trials proposing radiotherapy delay may necessitate a combination of anatomic and/or metabolic imaging at baseline and then sequentially every 3–6 weeks to corroborate pre-specified outcomes (e.g., stable disease). Other non-metabolizable glucose analogs and imaging modalities are being developed to circumvent the limitations of repeated PET radiation exposure [458].

The specific metabolic analysis of each patient’s tumor tissue is more relevant to research settings. Given the ongoing debate regarding the absolute degree of intratumoral metabolic heterogeneity, diagnostic tools have been developed to gain a more accurate picture of primary metabolic dependencies [58, 459, 460]. These include bench-top assays for the mapping of glycolytic, glutaminolytic and oxidative pathways, oxygen consumption and extracellular acidification rates (OCR/ECAR), mtDNA sequencing, ultrastructural characterization, novel PET tracers (e.g., glutamine, ketone bodies, oxygen sensors, amino acid metabolism, lipid synthesis, apoptosis), MRS/MRI imaging (e.g., glucose, glutamine, ATP synthesis, TCA cycle, ketone bodies), and NMR and LC/GC–MS metabolomics [61, 461, 462]. Metabolic assays can be performed non-invasively or in fresh tumor preparations and patient-derived organoids [463]. The translational value of metabolic stratification and the associated phenotypes has been further discussed in Additional File 1: Appendix 2.

The fundamental bioenergetic hierarchy of normal cells (OXPHOS > SLP) and cancer cells (OXPHOS < SLP) should not be forgotten when developing mitochondria-targeting drugs, such as electron transport chain inhibitors [63, 464–468]. Recent clinical efforts to target OXPHOS have been halted due to severe and arguably predictable toxicity, suggesting a very narrow therapeutic index [469]. In our view, low-dose mitochondrial targeting would be mechanistically sound only after the effective inhibition of SLP flux, as cancer cells have already adapted to fermentation as a compensatory and/or biosynthetic mechanism. From this perspective, cancer cells lacking SLP dependency may no longer display the primary hallmark of cancer (i.e., dysregulated cell growth). Better mechanistic insights into how restoring OXPHOS sufficiency regulates cell division could be gained from nuclear-cytoplasm transfer experiments and mitochondrial transplantation, rather than OXPHOS inhibition [470–472].

### Surgical debulking

Surgical debulking should be performed promptly after diagnosis, while still prioritizing careful surgical planning to ensure maximal resection. In asymptomatic or slow-growing tumors, active surveillance provides an opportunity to implement dietary KMT as a neuroprotective intervention prior to surgery, which could reduce angiogenesis, inflammation, and edema [126, 221, 473], and thus potentially facilitate better surgical delineation when coupled with metabolic imaging and intraoperative markers [456, 474, 475]. A short active surveillance interval to allow for KMT initiation in suitable non-critical cases has not been explored in earlier GBM trials (Additional File 3: Table S1), likely due to IRB approval policies [112, 476]. The extent of surgical resection is one of the most important predictive factors for GBM survival [477]. To ensure complete resection of all contrast-enhancing areas, intraoperative fluorescence markers such as 5-aminolevulinic acid (5-ALA) or novel pH-sensitive agents can be considered for eligible patients [478–480]. Most recurrences are experienced locally or in proximity to the resection cavity of the first surgery [481, 482]. Patients initiating KMT after recurrence should evaluate the possibility of repeated surgical debulking, unless presenting with diffuse, multifocal, or deep infiltrative tumors; cytoreductive surgery for well-defined recurrent lesions extends survival and may facilitate salvage therapies [350, 483, 484].

### Initiate dietary KMT

GKI-adjusted KD/KD-R and fasting can be administered in a neoadjuvant phase (in the peri-diagnostic period), uninterrupted, or resumed within 24–72 h of surgical debulking, depending on the patient’s condition [485, 486]. Mechanistically, these strategies have been studied to improve wound healing and reduce inflammatory markers [158, 487–492], alleviate pain [493, 494], and stimulate anti-tumor immunity [170, 495], thus inducing a favorable physiological environment for post-surgical recovery. Long-term adherence should be stratified according to cumulative biomarker ranges, such as real-time tracking of GKI, which can be predefined as primary or surrogate endpoints.

If KMT has been initiated prior to surgery during a watchful waiting period, patients with adequate body weight and good functional status may accelerate ketogenic adaptation through water-only fasting or fasting-mimicking diets (FMDs) [145, 368]. However, adjusting to an isocaloric KD or KD-R for 1 to 3 weeks before undergoing zero-calorie or partial fasting enables a more gradual metabolic transition. A well-formulated KD should proactively avoid common side effects, such as electrolyte imbalances or undesired LBM loss, to minimize negative impacts on quality of life. Initial weight loss during the transition to nutritional ketosis and fasting is mostly due to increased diuresis (water loss) and fat loss, not LBM [102, 496]. Adequate hydration and electrolyte supplementation (e.g., sodium, chloride, magnesium, and potassium) is recommended for both KDs and fasting [497, 498]. In clinical trials, successful implementation of KDs is often accompanied by a reduction or discontinuation of medication, particularly for chronic diseases associated with insulin resistance, such as type 2 diabetes, dyslipidemia, NAFLD, and hypertension [384, 499].

Based on changes in the metabolome, water-only fasting for periods over 72 h is likely required to fully transition into the fasted state in humans [371, 500, 501], although more research is needed to determine the appropriate timing for antineoplastic effects [502, 503]. Medically supervised water-only fasting for over 60 days has been shown safe and effective in obesity management [375], and fasting-mimicking protocols for up to 21 days have been implemented in large cohorts with normal baseline weight [369]. While sufficient body fat stores could allow for therapeutic fasting beyond 1–3 weeks in select patients, feasibility studies focused mostly on 5–7 days in GBM [145], as well as short-term and fasting-mimicking protocols in other malignancies [368, 504]. After the fast, the attending dietitian should instruct a slow and methodical refeeding (while still adhering to GKI targets) to prevent overfeeding, electrolyte imbalance, or reactive hyperglycemia [145, 311, 505].

If fasting is contraindicated due to risk of cachexia or preexisting health conditions, an isocaloric GKI-adjusted KD can be initiated instead [375, 506]. It is important to review sodium restriction, as low sodium diets have been shown to deplete magnesium and increase insulin resistance, thus promoting hyperglycemia [507, 508]. Similar to water-only fasting, a strict KD is expected to induce mild diuretic effects and improve glycemic control; accordingly, it is recommended to reevaluate existing prescriptions (e.g., antihypertensives, antidiabetics) and replenish electrolyte levels, especially prior to acute dietary changes. Asymptomatic hyperuricemia may also develop in a small subset of patients and should be monitored, resolving spontaneously in most cases [509–511].

The initial weight loss from adipose tissue is expected to continue slowly and controllably during KD-R. It is important to remember that gradual fat utilization associated with KD-R and fasting is therapeutic, whereas LBM loss associated with cachexia is pathogenic [236, 512]. Isocaloric feeding to maintain muscle mass should be favored over chronic calorie restriction in individuals at borderline low weight (e.g., BMI < 18, or as determined by the dietitian) [513]. Participants that cannot comply with the diet (e.g., impaired swallowing function) could receive nutrients in optimal balance via enteral feeding (as demonstrated in pediatric and adult patients with epilepsy), or, if enteral feeds are not possible, via parenteral ketogenic nutrition [256, 514–516].

### Radiation therapy

In addition to neurosurgery, radiotherapy of growing sophistication has remained the cornerstone of GBM therapy [6, 517, 518]. The current SOC recommends postoperative radiation with target volume delineation, for a total dose of 60 Gy in 30 fractions [26]; temozolomide alone is typically only considered in elderly patients, especially if the tumor is MGMT-methylated [519, 520]. Given the conflicting effects on cancer metabolism described below, in the specific context of future research evaluating diet-drug KMT as the primary treatment modality, a proposal to modify the timing of radiotherapy could be requested by the investigators if biologically justified. Despite short-term cytotoxicity to cancer cells, ionizing radiation induces metabolic reprogramming in the tumor niche, negatively influences the phenotype of recurrence, and triggers secondary inflammatory responses in the peritumoral tissue [521–526], while also damaging normal brain parenchyma and blood vessels [527–529]. Even targeted modalities can cause delayed adverse effects that are seldom factored in the risk/benefit analysis given the poor overall prognosis [78, 530, 531]. Concerns have been raised about the potential off-target brain toxicity caused by conventional radiation protocols [532–536]. Consequently, it will be important to design clinical trials comparing the potential synergistic benefit of radiation-induced cytotoxicity, chemosensitization, and immune modulation, with the residual adverse effects on surviving tumor cells and their microenvironment [196, 537, 538]. To meet IRB requirements, radiotherapy could be conditionally and sequentially delayed for a clinically acceptable period based on interim response analysis or applied at low-dose regimens as a synergistic potentiation strategy (e.g., NCT01466686) [537–540].

In other types of cancer, KDs and fasting have been proposed as feasible and potentially effective radiotherapy adjuncts, acting as radiosensitizers while mitigating adverse effects [541–543]. It is worth reiterating that, in contemporary medical ethics, therapeutic decision-making is ultimately driven by the informed patient [330, 544–546]. Accordingly, brain-sparing modalities of radiation may be offered as auxiliary or salvage therapies if other approaches have failed or if they are actively requested by the participant [532, 533, 547].

#### Drug treatments

It is our view that any drug therapy will be most effective once the patient reaches a stable therapeutic GKI zone (for example, 2.0 or below, ideally 1.0 or below, with special attention to absolute glucose levels and insulin signaling, which should be at their physiological minimum). A combination of nutritionally balanced KDs, calorie restriction, and fasting will promote therapeutic ketosis, after which drug therapies can be initiated.

As corticosteroids decrease immune function and increase glycemia, independently associated with poor GBM survival, they should be used only when unavoidable, at the lowest dosage, for the shortest possible time [80, 81, 548]. Alternatives allowing for dose reduction include combinatory regimens of non-steroidal medications such as COX-2 inhibitors, fingolimod, acetazolamide, angiotensin II receptor antagonists, ACE inhibitors, or glyburide (which impacts insulin signaling) [549–552]; nutraceuticals such as boswellic acids [553]; as well as novel agents such as corticorelin acetate, vaptans and vascular endothelial growth factor (VEGF), or vascular endothelial protein tyrosine phosphatase (VE-PTP) antagonists [554]. The rationale for dexamethasone should be reevaluated upon edema reduction, rather than prescribed as an indefinite treatment [555]. Patients and caregivers are encouraged to inquire periodically about the clinical justification of the ongoing corticosteroid posology.

It is important to recognize that intensive SOC therapy (particularly, high-dose corticosteroids, and radiation) could lead to erratic glycemia or low ketonemia despite strict diet adherence [556, 557]. Based on previous reports, more intensive dietary changes, such as cyclical water-only fasting (≥ 3–5 days) and paleolithic KDs (≈ 0 g carbohydrates/day) with a narrow feeding window (e.g., intermittent fasting with one meal per day), may be required to reach a stable GKI during concomitant steroid and radiation therapy [108, 145, 196, 218, 558]. Such personalized dietary adjustments are compatible with trial planning and can be implemented at the discretion of the attending physician or dietitian (specific biomarker targets can be pursued as primary endpoints, but it may be impractical to predefine all possible methods to achieve them).

Metabolic imaging and previous characterizations of the tumor subtype can suggest a preliminary description of the primary metabolic dependencies [451, 559, 560]. After transitioning to a sustained GKI-adjusted KD/KD-R, pharmacological targeting of glycolysis and glutaminolysis should be implemented gradually, ensuring any off-target SLP inhibition in normal cells is buffered via ketone body metabolism. Baseline ketogenic adaptation is a “*sine qua non*” condition for the safe targeting of SLP fuels. This is not required for modulating other cancer-associated pathways, such as redox balance, immune response, or autophagy, but is recommended for its synergistic anti-proliferative, anti-inflammatory, and anti-angiogenic effects [68, 126, 166–170, 561].

Additional File 5: Table S2 and Additional File 6: Table S3 summarize repurposed drugs and novel research-phase chemicals for the targeting of SLP and tumor-associated pathways. While we provide general recommendations based on the press-pulse therapeutic principle, a multitude of clinically approved drugs have been proposed as candidates for GBM therapy [539, 562, 563]. Combinatory approaches, rather than single-pathway targeting, may be necessary for optimal results [394]. However, in efforts to isolate confounding variables and mitigate financial risk, only a small number of clinical trials have tested multi-drug additions to SOC, seldom with dietary metabolic priming [564–566]. The intellectual property landscape and lack of financial incentives to explore non-patentable combinatorial approaches is a significant challenge for the validation of promising preclinical observations [567, 568]. Improving drug bioavailability, blood–brain barrier transport and local delivery (e.g., intracranial drug reservoirs) are also critical factors [539, 569]. Understandably, even though off-label prescription is permissible in most countries on a case-by-case basis, the general use of any non-standard therapy will need be validated and incorporated into SOC through extensive clinical testing [567, 570]. Regardless of the clinical context, monitoring of adverse events and dose modification schedules must be in place for any tested pharmacological agent.

**Fig. 5:**
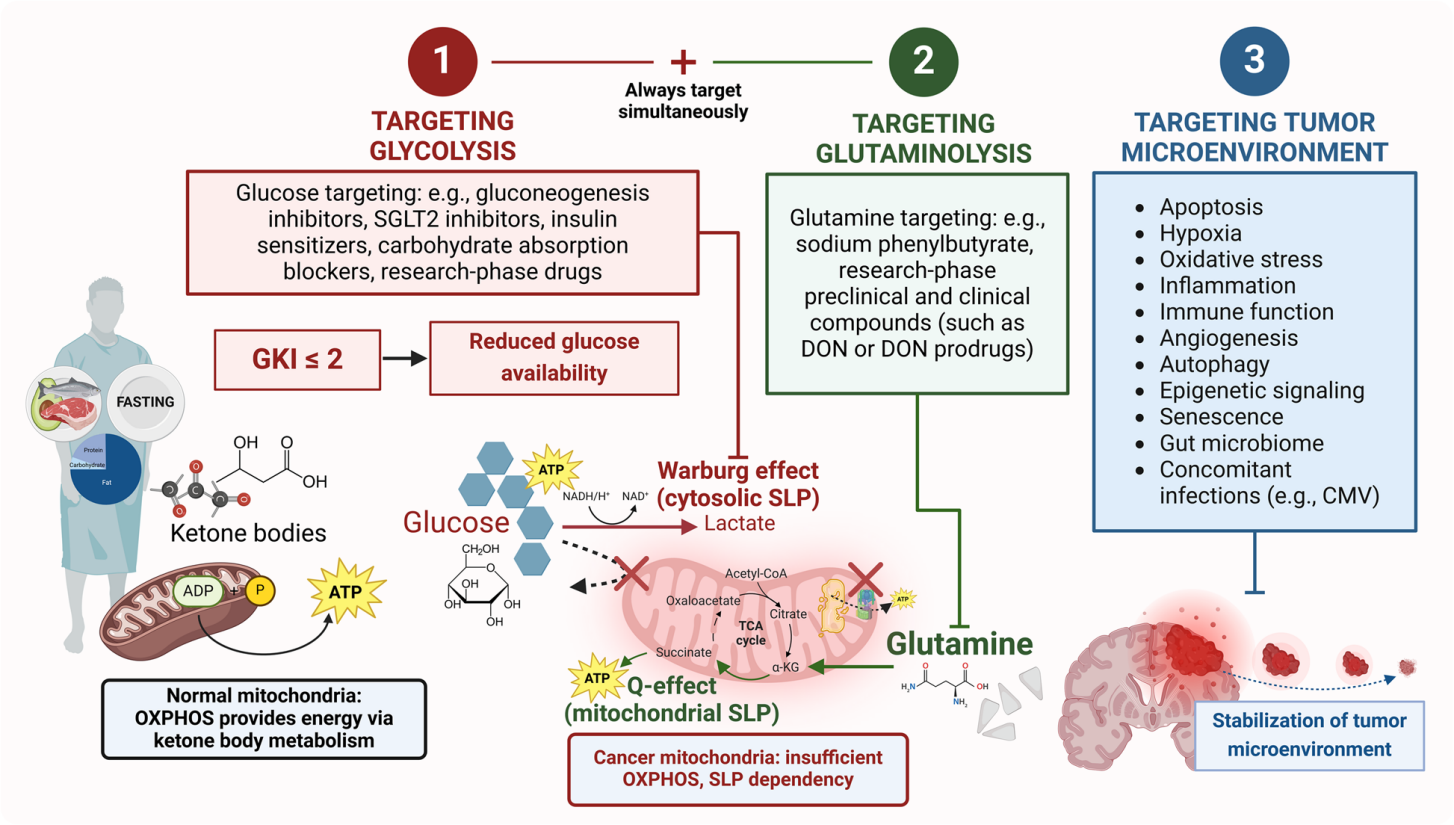
Overview of potential drug treatments as part of KMT research. Strategies are divided into glucose targeting (red), glutamine targeting (green), and tumor microenvironment stabilization (blue). Safe administration of metabolic inhibitors will require physiological adaptation to a GKI-adjusted KD/KD-R, which can be accelerated by water-only fasting. Then, glycolysis targeting can be considered to further improve GKI and slow tumor progression (e.g., antidiabetic agents such as metformin or SGLT2 inhibitors, as well as research-phase glycolytic inhibitors). Glutaminolysis should be targeted at the same time (e.g., sodium phenylbutyrate, DON, or novel glutamine inhibitors). Finally, normalization of the tumor microenvironment can be explored in a modular fashion; for example, cell proliferation (mebendazole), inflammation (NSAIDs), hypoxia (HBOT), redox balance (DCA, intravenous vitamin C), immunotherapy, or combinatory approaches (e.g., CUSP9)

The timeline of drug administration is outlined in Fig. 5 and can be structured as follows:

- Dietary KMT (GKI-adjusted KD/KD-R and fasting) reduces the glycolytic dependency of normal tissues and stimulates compensatory ketone body metabolism.
- If the tumor is shown to be glycolytic (i.e., high 18F-FDG uptake), consider additional systemic targeting of substrate, such as renal glucose reabsorption or gluconeogenesis inhibitors (e.g., SGLT2 inhibitors, metformin). Direct inhibition of glycolysis should be administered only after reaching sustained therapeutic ketosis to improve safety and tolerability (“keto-adaptation”). Therapeutic targets in early clinical trials include hexokinase (2-Deoxy-D-glucose, lonidamine, 3-bromopyruvate), phosphofructokinase (3PO, ACT-PFK-158), and pyruvate kinase (gossypol/AT-101, TLN-232) [571, 572].
- We propose that concurrent targeting of glutaminolysis is essential to avoid therapy resistance. At this time, one of the anti-glutaminolytic drugs considered to work best as part of KMT in preclinical models is the pan-glutaminase inhibitor 6-diazo-5-oxo-L-norleucine (DON) [143]. Any prospective compound that can safely and effectively target glutamine availability and/or utilization may elicit comparable effects, such as DON prodrugs or novel glutaminase inhibitors [573, 574]. Additionally, tumor-specific delivery of DON is being investigated as an enhancer of anti-tumor immunity [575].
- After cancer cells have been rendered vulnerable by press-pulse metabolic pressure, cancer-associated pathways and the tumor microenvironment can be targeted via synergistic drug combinations.

### Pharmacological targeting of glycolysis

Dietary KMT shifts whole-body physiology to an evolutionarily conserved metabolic state of nutrient scarcity that is inhospitable to tumor growth, but facing advanced disease will require multimodal and combinatorial strategies [103, 105, 162, 576, 577]. Further improvements to GKI (substrate availability) and direct targeting of glycolysis can be implemented after ketogenic adaptation via drug repurposing or investigational compounds, depending on the clinical context (Additional File 5: Table S2).

Metformin at standard antidiabetic dosing improves glycemic control via mild liver gluconeogenesis inhibition and increased insulin sensitivity [578]. At realistically achievable in vivo concentrations, direct cytotoxicity via complex I inhibition is unlikely, but positive regulation of the tumor immune microenvironment has been noted [579]. Cancer therapy with metformin is being evaluated in an expanding number of clinical trials due to its good safety profile, mostly as a synergistic addition to SOC [580, 581]. Berberine, an over-the-counter alternative, exhibits similar effects on glycemic control [582]. Other biguanides may be more efficacious in lowering gluconeogenesis but also carry a higher risk of lactic acidosis, which restricts their use to research [583, 584]. At this time, we do not recommend OXPHOS inhibitors with higher potency, such as IACS-010759, due to unacceptable off-target toxicity [585, 586].

SGLT2 inhibitors (e.g., dapagliflozin, empagliflozin, canagliflozin) can be considered to further decrease GKI and insulin signaling [587–589]; dose adjustments and monitoring of ketoacidosis is required during KDs and fasting, especially in patients with a history of type 2 diabetes or prone to ketoacidosis [590–593]. SGLT2 inhibitors experienced a recent resurgence as attractive combinations with PI3K inhibitors via suppression of the insulin feedback loop; however, greater synergy was observed with the KD in certain preclinical models, even if future clinical adoption would be more demanding [68, 594]. Therefore, a dual combination of low-dose SGLT2 inhibitors and KDs warrants further research. In fact, the KD is now being rebranded as “insulin suppressing” and trials with PI3K inhibitors are underway [595]. Renewed interest in the often-overlooked intersection between diet and cancer may lead to a more universal appreciation of “how common clinical practices such as intravenous glucose administration, glucocorticoid use, or providing patients with glucose-laden nutritional supplements may impact therapeutic responses” [68].

Other antidiabetic drugs that do not act through endogenous insulin production, such as thiazolidinediones (“glitazones”), dipeptidyl peptidase-4 (DPP-4) inhibitors (“gliptins”), glucagon-like peptide 1 (GLP-1) agonists or bromocriptine, could be tools to achieve and sustain specific GKI ranges. Exogenous insulin causes surges in growth signaling that may accelerate tumor progression and chemoresistance despite transient glucose disposal, with a controversial role in cancer therapy [596]. The consequences of drug-induced insulin secretion (e.g., sulfonylureas or meglitinides) in the context of therapeutic ketosis, where insulin should be physiologically low, are not fully elucidated. MCTs and exogenous ketones can rapidly boost ketone levels and prevent hypoglycemic events during drug therapies, KDs or fasting, as well as mitigating central nervous system (CNS) oxygen toxicity in adjuvant hyperbaric medicine [277, 597].

Beyond systemic glucose availability, several research-phase chemicals that target the glycolytic pathway at the substrate, transport, or enzyme level have been explored in clinical trials, such as the classical competitive inhibitor 2-Deoxy-D-glucose [598]. However, it is important to note that systemic inhibition of glycolysis without preemptive priming to alternative energy pathways could lead to dose-limiting toxicities [66]. In our view, effective and sustained ketogenic adaptation at the biological level should be a prerequisite for the clinical testing of glycolytic inhibitors. Thus far, direct targeting of glycolysis has been relatively limited due to safety concerns [70], which could be partially offset by adjuvant dietary KMT and dose optimization, given that therapeutic ketosis also reduces glycolytic flux and increases tissue competition. Therapy resistance or prior metabolic stratification could provide a rationale for intensifying glycolysis targeting beyond substrate availability, as suggested by the principles of precision medicine [599].

### Pharmacological targeting of glutaminolysis

In the context of monotherapy inhibition of glycolysis, cancer cell viability could be rescued by the other primary fermentable fuel, glutamine [33]. Even though intra-tumoral heterogeneity and clonal selection creates a potentially unlimited mutational and epigenetic landscape [600, 601], the number of metabolic substrates able to sustain proliferation is unlikely to be unlimited in light of the universal mitochondrial defects and bioenergetic/anabolic dependencies found in GBM [40, 43, 45, 115]. Currently, novel glutamine antagonists and other metabolic inhibitors such as DON prodrugs or CB-839 are being tested as monotherapy additions to SOC (Additional File 5: Table S2). Single-pathway inhibition may not be optimal due to functional redundancy: unless proven otherwise through metabolic stratification, we propose concurrent initial targeting of glucose and glutamine-driven SLP after ketogenic adaptation, given that they are the most robustly consumed for energy, biomass, and redox homeostasis [31].

DON is the prototypical drug for broad-acting glutaminolysis inhibition, targeting multiple isoforms of glutaminases and glutamine-utilizing enzymes [602]. DON is currently not FDA-approved, but has an extensive history of clinical testing, a relatively good safety profile at moderate doses, and could be revisited as a research therapy in its original or prodrug forms [603–605]. Continuous daily parenteral administration produced dose-limiting side effects in previous clinical trials (most notably, oral mucositis, nausea, vomiting, and myelosuppression; premedication with antiemetics can be implemented prophylactically) [606–608]. Instead, congruent with the short half-life (1.2 h), low-dose intermittent administration would be preferable, as suggested by initial dose-escalation studies. Thus, future research may consider parenteral or oral administration in the 0.2 to 1.1 mg/kg/day range, adjusted to individual tolerance [609, 610]. Dosing frequency (continuous or intermittent) will be contingent upon route of administration, anti-tumor response, and safety. In more recent phase IIa studies, DON has been administered at 140 mg/m^2^ (twice weekly) in 15-min infusions, combined with plasma glutamine depletion [611].

While DON prodrugs with improved oral bioavailability are being developed, the original compound demonstrated biological activity at oral doses up to 1.1 mg/kg/day for a duration of two or more weeks [603, 612]. DON has been administered orally as a single daily dose (without resting periods), continuous split doses every 4–6 h (with a higher incidence of side effects, such as oral mucositis), or as single or split doses given intermittently every 2–4 days (lowest incidence of side effects) [609]. Preclinical evidence from our group suggests that the KD-R may increase DON concentrations across the blood–brain barrier and reduce dosing requirements when administered on a per-need basis [143]. Based on previous clinical testing, the recommended starting point would be a lower daily dose taken with a fatty meal vehicle in a single (e.g., 0.4 mg/kg q24h) or split schedule (e.g., 0.1 mg/kg q6h), with a 1–3-day resting period upon side effects, increasing to 1.1 mg/kg (or higher) based on tolerance and pharmacokinetics. Single 1.2–2.5 mg/kg oral doses were necessary to reach serum peak concentrations comparable to 0.6–1.2 mg/kg intravenous infusion; consequently, rather than oral administration, subcutaneous delivery starting in the 0.2 to 0.4 mg/kg range (twice or thrice weekly) may be preferable for improved bioavailability and convenience in outpatient care [609, 613]. Ideally, DON would be administered after confirming stable therapeutic ketosis as a synergistic potentiation strategy. It has been suggested that supplementing DON with adenine (400 mg/day) or 4-amino-5-imidazole carboxamide (800 mg/day), gastric pH-buffering (due to DON acid-labile properties), and hypoxanthine and increased fiber intake, may reduce off-target damage to the oral and intestinal mucosa; nevertheless, the mechanisms underlying these protective effects and their relevance on therapeutic efficacy will need to be confirmed in future studies [609, 614]. The immunomodulatory effects of DON should also be considered in the context of checkpoint inhibition, neoantigen vaccines, and adoptive cell therapy [615, 616].

Sodium phenylbutyrate is a clinically approved orphan drug for urea cycle disorders and neurodegenerative diseases, with potential anti-tumor effects as a single agent or coadjuvant with glutamine antagonism [617, 618]. Phenylbutyrate rapidly metabolizes to phenylacetate, conjugated with phenylacetyl-CoA and glutamine, acting as an ammonia scavenger and inducing durable plasma glutamine depletion [619]. It is also being investigated as a histone deacetylase (HDAC) inhibitor [620]. Clinical trials in solid tumors noted a sustained dose-dependent reduction in plasma glutamine with oral doses between 180 and 360 mg/kg/day, up to a maximally tolerated dose of 27 g/day [617, 621–623]. Phenylbutyrate decreases systemic availability of glutamine, resulting in substrate competition; thus, similar to PEG-glutaminase, phenylbutyrate-induced glutamine depletion may be explored to reduce dosing requirements of DON or other enzyme-level inhibitors of glutaminolysis [624]. Interestingly, the administration of phenylacetate was feasible after prolonged fasting, accompanied by counterregulatory hormonal responses to maintain fuel homeostasis [625].

L-asparaginase, a first-line treatment for a variety of lymphoproliferative disorders, induces acute extracellular glutamine depletion through conversion to glutamate, a mechanism hypothesized to play a significant role in its antineoplastic benefits [626, 627]. L-asparaginase requires parenteral administration and is currently available in three formulations (including generic drugs) [628]. Clinical trials in solid malignancies have focused primarily on single addition to chemotherapy in pancreatic cancer, yielding only marginal improvements in survival [629]. Consequently, it has been proposed that combinations with specific glutaminolysis inhibitors such as DON may further improve therapeutic efficacy [630–632].

Other research-phase glutamine inhibitors include the aforementioned DON prodrugs (e.g., JHU-083 and DRP-104, which contain the same active compound but aim to improve bioavailability and pharmacodynamics; Azo-DON, which is selectively reduced to DON by azo-reductases in hypoxic environments; as well as azotomycin, a tripeptide diazo analog) [605, 613, 617, 633], CB-839 (telaglenastat), IPN60090, BPTES, and compound 968 (glutaminase inhibitors) [574, 634–636], V-9302 (glutamine transport inhibitor) [637], azaserine and acivicin (glutamine mimics) [638], and caudatan A [639], physapubescin K [640], and aspulvinone O [641]. Blood–brain barrier permeability as well as isoform specificity are limiting factors, given that targeting all glutaminases (rather than specific isoforms) may be preferable to avoid therapy resistance. Telaglenastat is an investigational, first-in-clinic, small molecule oral selective inhibitor of GLS1, which has reached up to phase II clinical trials in advanced solid and hematological malignancies, including IDH-mutant astrocytoma [157, 634, 642, 643]. Most active trials are now focusing on combinations with targeted therapies and immunotherapies, but we hypothesize that glycolytic compensation may also play a role in the mixed efficacy reported so far [644, 645]. Likewise, the orphan drug CPI-613 is a lipoic acid analog that targets alpha-ketoglutarate dehydrogenase (α-KGDH), inhibiting both mitochondrial SLP and TCA cycle flux, with a relatively good safety profile but disappointing performance in metastatic pancreatic cancer [646–648]. Despite failure as a single agent, we have observed a promising synergistic interaction when CPI-613 was combined with the KD-R in a pediatric glioma model [649].

Repurposed drugs with potential inhibitory effects on the glutaminolytic pathway include aminooxyacetate, apomorphine, tamoxifen/raloxifene, sulfasalazine, and ceftriaxone [36, 650–652]. Over-the-counter nutraceuticals with direct or indirect effects include EGCG [653], xanthohumol and hesperidin [654], ursolic acid [655, 656], caffeic acid [657], curcumin [658], apigenin [659], berberine [660], and other compounds with only preliminary mechanistic evidence [661]. Achieving effective inhibition of glutamine metabolism through supplementation may be difficult, unless standardized for equivalent biological activity. Patients are therefore encouraged to inquire about ongoing clinical trials or compassionate use of glutaminolysis inhibitors (such as DON, novel DON prodrugs, or CB-839). When enrolling into clinical trials, participants should be offered flexibility to implement dietary KMT with additional glycolysis targeting, given that monotherapy inhibition has only produced modest clinical benefits thus far [71].

### Pharmacological targeting of the tumor microenvironment and cancer-associated pathways

The tumor microenvironment has a profound impact on therapeutic outcomes and is influenced by factors such as hypoxia [662], redox balance [663], immune function [664], inflammation [665], angiogenesis [666], autophagy [667], epigenetic signaling [668], radiation-induced senescence [669], the gut-brain signaling axis [670], tumor-synaptic networks [671], and concomitant infections (e.g., GBM exhibits a high detection rate of cytomegalovirus, which can contribute to increased oncogenic signaling, and has been clinically targeted using antivirals such as valganciclovir or adoptive cell therapy) [672–674]. The patient’s internal “macroenvironment,” that is, whole-body physiology and its exposome, also plays an undeniable but often forgotten role, especially if envisioning cancer as a competing “ecological” process between normal and malignant cells [675–677]. For example, insulin resistance and the accompanying hyperinsulinemia have been correlated with poor prognosis and can substantially reduce the efficacy of certain therapeutic approaches, such as inhibition of the insulin/PI3K axis [678–680]. GKI-adjusted KDs and fasting promote a wide-ranging normalization of the patient’s physiological macroenvironment, as well as the local tumor microenvironment, at all the levels described above [129].

Beyond metabolism, several targeted therapies based on mutational heterogeneity have been evaluated in clinical trials with arguably underwhelming results; these include growth and signaling pathways with known alterations in GBM, as well as multi-kinase inhibitors and immunotherapies [9, 681]. A lack of multi-targeted approaches has been highlighted as one of the possible reasons for this failure [682]. We hypothesize that classical antineoplastics and targeted efforts would be enhanced if applied on a baseline of dietary KMT with effective SLP targeting [102, 170, 221, 683]. For example, tumor neoantigen heterogeneity could be reduced by clonal selection through metabolic pressure, potentially improving immune recognition [684]. Early trials of checkpoint inhibitors in GBM failed to show efficacy due to the relatively immunoprivileged nature of the CNS [685, 686]. Treatment strategies aiming to overcome this site-specific limitation are underway, such as neoantigen-derived peptide and dendritic cell vaccines with coadjuvant immunostimulation [23, 687, 688]. The immunomodulatory effects of dietary and pharmacological KMT could promote and maintain a tumor-suppressive phenotype [495, 604, 689–691]. It should be noted that targeted therapies and KMT are generally compatible, with further studies needed to uncover synergistic opportunities [169, 170, 683, 692–694]. However, since most targeted therapies are only available in research settings, it is also worth exploring off-label indications with putative anti-cancer effects that are more easily accessible during routine clinical practice.

Additional File 6: Table S3 summarizes clinically approved drugs and strategies that have been proposed to modulate the GBM microenvironment. We refer to additional reviews discussing novel compounds and off-label indications with preclinical evidence that may hold promise but require further clinical testing [695–697]. It is important to note that this list is based on preexisting clinical use (“drug repurposing”) and may not involve the most potent or selective compounds in their respective category; rather, the intent is to address health disparities and lower the financial burden of cancer treatments, promoting a democratization of cancer care and off-patent drug development through publicly funded research [406, 698]. Additional File 1: Appendix 3 provides further detail on an illustrative selection of repurposed drugs that have initiated pilot safety and feasibility studies in GBM.

From an experimental perspective, combining multiple therapies will make it difficult to assign causality. It is also possible that certain interventions will increase the risk of toxicity or adverse interactions without a meaningful therapeutic benefit. Successful examples of the feasibility of multi-drug protocols can be found in the CUSP9 [394], CLOVA [699], MEMMAT [700], COMBAT [701], gMDACT [702], renin-angiotensin modulators [703], and COAST (NCT05036226) clinical trials. Cancer metabolism was not the primary target in the aforementioned proof-of-concept studies, and they did not include a “metabolic priming” dietary baseline. During informed consent, the risk/benefit analysis of combining individually safe but collectively undefined off-label drugs should be weighed against the expected efficacy of SOC and the biological rationale, including preclinical evidence. The key highlighted concept in this regard is the targeting of glycolysis and glutaminolysis while under therapeutic ketosis (metabolic press), in synergy with cancer-associated pathways (microenvironment pulse), rather than endorsing any specific drug combination as the most desirable for this purpose. Metabolic and molecular analysis during this process is important to reveal if evolutionary pressure selects for therapy-resistant cells. Future clinical research will be required to establish the optimal dosing, timing, and scheduling of the most effective press-pulse KMT combinations.

A major current limitation of drug repositioning is the lack of molecularly driven stratification and robust biomarkers to guide personalized therapy. Drug selection is often based on rational combinations that have demonstrated synergistic cytotoxicity in preclinical models, rather than specific tumor characteristics [704]. Safety concerns may understandably arise in multi-drug protocols at both the pharmacokinetic and pharmacodynamic level. To isolate the strength of each variable, most clinical trials involve single drug additions to SOC. In combinatory trials, assessing the benefit of each intervention becomes increasingly difficult, even in cross-over and multi-arm designs. Furthermore, patients with cancer are often polymedicated for prior comorbidities, with overlapping antineoplastic treatments making them a particularly vulnerable population. It is therefore important to carefully evaluate participants according to baseline health status and available molecular markers, starting with the safest interventions that demonstrate the highest scientific rationale. If combinatory approaches are proposed, drug-to-drug interactions must be screened preemptively (e.g., CYP system), followed by a slow dose buildup to foster tolerability, as elegantly illustrated in the CUSP9 trial [394]. Despite these challenges, we believe that press-pulse targeting of tumor-associated pathways in synergy with KMT will be developed as an affordable and translationally viable therapeutic strategy.

### Over-the-counter dietary supplementation

It is beyond the scope of these guidelines to detail all possible dietary supplements that may be of interest during multimodal cancer therapy. As a general concept, lifestyle interventions and supplementation are intended to improve the adaptive capacity of the non-tumoral cell mass (the prevailing portion of the patient’s ecology), which will compete with the tumor for bioenergetic and biosynthetic resources [705]. This also improves the likelihood that targeting of glycolysis, glutaminolysis, and the tumor microenvironment will be better tolerated by normal cells, or that synergistic opportunities may arise [706, 707].

Additional File 7: Table S4 includes common over-the-counter nutraceuticals with emerging preclinical and clinical evidence for complementary cancer use, mostly through supporting healthy tissue function. Given that this list is not intended to be exhaustive, many excellent reviews on this topic can be found elsewhere [708–711].

It is exceedingly unlikely that large randomized clinical trials will be performed for non-patentable natural products: consequently, we encourage documenting and sharing individual clinical experiences via systematic case reporting in peer-reviewed, reputable scientific journals [366, 367]. Supplementation should be disclosed to the attending medical team and reported independently for each patient. It is indispensable to review contraindications, adverse reactions, and potential drug interactions, which can be screened using online databases [712]. It is also advisable to establish a clear timeline of intake to avoid putatively antagonistic combinations (e.g., antioxidant effects during pro-oxidant therapies) [713, 714]. It must be clearly stated that supplementation is not intended to resolve advanced cancer, and owing to its namesake, it should be viewed as “supplementary”. However, when implemented judiciously, it is also unlikely to interfere with most conventional antineoplastic drugs or KMT, thus becoming a personal choice of the informed patient [715].

### Physical activity

Moderate daily physical exercise is encouraged and should be tailored to the age and fitness of the patient, including both resistance/strength training for muscle maintenance as well as aerobic/high-intensity training for cardiometabolic health [716, 717]. As a core pillar of KMT, physical activity is anti-cachexic, increases insulin sensitivity, and facilitates physiological glucose and glutamine clearance [249, 718, 719]. Furthermore, low-intensity endurance exercises such as regularly spread-out walking (smaller doses but higher frequency) modulates osteocalcin and glucagon signaling, consequently lowering glucose availability and insulin secretion [720].

Recording of metabolic parameters (such as GKI) should be contextualized, as fuel utilization and transient stress responses may influence measurements in the post-exercise window. In light of the beneficial effects of exercise on reducing cancer mortality and recurrence [721–723], as well as the inverse association between muscle mass and strength with all-cause mortality [724], patients should strive for a daily dose of physical activity that is sufficient to stimulate muscle protein synthesis, adjusted to their training experience and comorbidities; for example, as per current cancer guidelines, aim for at least 150 min per week of aerobic exercise, two or more days a week of resistance training, or ≥ 10 metabolic equivalent of task (MET)-hours per week of overall physical activity [725–727].

### Stress management

Facing a serious cancer diagnosis can be traumatic and emotionally distressful, impacting patients’ mental health and psychosocial wellbeing [728]. It is pivotal that patients receive appropriate mental health support that suits their preferences and beliefs, including multidisciplinary psycho-oncological care with clinical psychotherapy, sleep hygiene, breathing exercises, limbic system retraining, meditation or prayer, not least because a high level of personal motivation is required for KMT implementation [729, 730]. On a physiological level, stress management is important to stabilize the hypothalamus–pituitary–adrenal axis and sympathetic nervous system, maintaining adequate cortisol levels, immune function, and circadian rhythms [731–733].

### Evaluation of outcomes and therapy adjustments

To assess the effectiveness of KMT, we recommend monitoring tumor response using non-invasive anatomical or combined metabolic imaging within the first 1–2 months (e.g., MRI with elective 18F-FDG PET at 8 weeks), and then every 2–4 months during active treatment, in line with standard guidelines [26, 734]. If changes to SOC timing are proposed in a diet-drug KMT trial (such as radiotherapy delay), neuroimaging may need to be more frequent to detect early trends in tumor progression. If the tumor is stable or shows signs of partial response, follow-up can be scheduled every 2 to 4 months for the next 2 to 3 years, and less frequently thereafter. It is important to create a schedule that would enable timely adjustments to the therapeutic plan. In extra-neural cancers, previously positive tumor markers as well as validated liquid biopsies may assist in estimating tumor burden [735–737]. Repeated surgical debulking can prevent bulk effect, especially in slow growing tumors [738]. Active monitoring and GKI-adjusted KD/KD-R should be maintained as long as there is evidence of persistent disease or risk of recurrence.

## Conclusions

### Ethical considerations and future directions

One of the greatest challenges in GBM therapy is the inability of the current SOC to eliminate all microscopic tumor infiltration and cancer stem cells [739, 740]. After the inevitable recurrence, patients are often confronted with salvage therapies of limited clinical utility [741]. These grim prospects make it difficult for physicians to communicate prognosis and for patients to make realistic and informed decisions about their preferred treatment plan [742–744]. It is not uncommon to avoid emerging therapies due to safety concerns (*primum non nocere*), fear of straying too far from the established guidelines (*defensive medicine*), or lack of familiarity. This may be entirely within *lex artis* for early-stage cancers, despite the perceived drawbacks of certain antineoplastics, which could be regarded as justifiable if durable remission is achieved [95, 745]. For terminal, incurable cancers, it is a matter of interpretation of medical ethics as to whom should be the arbiter of therapeutic decisions, especially for interventions where the risk/benefit ratio is not fully established [746–749].

This is an ethical consideration, not a scientific one, to be decided collectively at the societal and policy level. Nevertheless, from a patient advocacy perspective, advancing education about novel therapies at all the levels of the evidence-based pyramid is essential to facilitate shared decision-making. Going forward, a larger collection of clinical trials will be needed to standardize the implementation of GKI-adjusted dietary KMT with concurrent SLP targeting. This is the context where we aim to provide a comprehensive, minimally toxic, and cost-effective GBM treatment plan, with a solid theoretical background, pilot clinical studies, and ample research potential, as it is gradually developed to become part of the standard oncology toolkit. We wish to inspire patients to take a proactive and informed role in the management of their disease, physicians to make evidence-based decisions while still exercising clinical freedom, and researchers to join the quest for discovery of the many promising therapeutic avenues that are yet to come by targeting the fundamental bioenergetic dependencies of cancer cells.

A flexible and modular protocol has been presented to guide translational GBM research, based on the evidence that most of the defining hallmarks of cancer can be explained from a mitochondrial metabolic perspective [30, 35]. As predicted by evolutionary biology, cancer cells suffer from a distinctive lack of adaptive versatility due to both mitochondrial and genomic damage, as well as persistent anabolic demands. GBM cells, like most other cancers, are comparatively more dependent on SLP flux for energy and biosynthesis due to universal defects in mitochondrial number, structure, and function, despite ample downstream mutational heterogeneity, metabolic reprogramming, and single-cell heterogeneity [35, 152, 750].

KMT is conceptualized as a press-pulse therapeutic strategy. This framework can be adjusted for any cancer subtype that is unable to proliferate under the relative restriction of both glycolysis and glutaminolysis (SLP dependency), even when supplied with compensatory oxidative fuels (OXPHOS insufficiency). Given the biochemical underpinnings, it will be important to search for cancer models that retain uncontrolled cell proliferation using primarily OXPHOS after the simultaneous targeting of glycolytic and glutaminolytic SLP flux, as this would pose an exception to the mechanistic rationale. Future research, stemming from collections of case reports and clinical trials, will offer unique insights into the optimal dosing, timing, and scheduling for maximally safe and effective SLP targeting after physiological adaptation to therapeutic ketosis.

## Supplementary Information

Supplementary Material 1.

Supplementary Material 2.

Supplementary Material 3.

Supplementary Material 4.

Supplementary Material 5.

Supplementary Material 6.

Supplementary Material 7.

## Data Availability

No datasets were generated or analysed during the current study.

## Abbreviations

2-HG: 2-Hydroxyglutarate
ADF: Alternate-day fasting
AUC: Area under the curve
BMI: Body mass index
CGM: Continuous glucose monitoring
CKM: Continuous ketone monitoring
CNS: Central nervous system
DCA: Dichloroacetate
DON: 6-Diazo-5-oxo-L-norleucine
ECAR: Extracellular acidification rate
FMD: Fasting-mimicking diet
GBM: Glioblastoma
GKI: Glucose-Ketone Index
HBOT: Hyperbaric oxygen therapy
HDAC: Histone deacetylase
IDH: Isocitrate dehydrogenase
IRB: Institutional Review Board
KD: Ketogenic diet
KD-R: Calorically restricted ketogenic diet
KMT: Ketogenic Metabolic Therapy
LBM: Lean body mass
MAD: Modified Atkins diet
MBZ: Mebendazole
MCT: Medium‐chain triglyceride
MKD: Modified ketogenic diet
mOS: Median overall survival
NSAID: Nonsteroidal anti-inflammatory drug
OCR: Oxygen consumption rate
OGTT: Oral glucose tolerance test
OXPHOS: Oxidative phosphorylation
PFS: Progression-free survival
SLP: Substrate-level phosphorylation
SOC: Standard of care
SUV: Standardized uptake value
TTF: Tumor-Treating Fields
VEGF: Vascular endothelial growth factor
VE-PTP: Vascular endothelial protein tyrosine phosphatase
βHB: Beta-hydroxybutyrate
