# Supplementary material for: Clinical research framework proposal for ketogenic metabolic therapy in glioblastoma
Source: BMC Med. 2024 Dec 5;22:578. doi: 10.1186/s12916-024-03775-4 (PMC11622503; doi:10.1186/s12916-024-03775-4)
Supplement: Supplementary file 1 — Supplementary Material 1. [file 12916_2024_3775_MOESM1_ESM.docx]

### Appendix 1. Confronting myths: relative and absolute requirements of dietary carbohydrates and glucose as metabolic fuels.

It is pertinent to briefly discuss the enduring misconception that glucose itself represents an “absolutely essential”, “universal fuel” in human physiology, which requires nuanced definition and gradation, but has been perpetuated *verbatim* and may have been incorporated into the physiology education of currently practicing healthcare professionals [1-7].

We must first address the distinction between endogenous and exogenous sources of glucose. Clinical trials and epidemiological studies of very low to zero carbohydrate diets support the statement of the US National Academies of Sciences that “the lower limit of dietary carbohydrate compatible with life apparently is zero, provided that adequate amounts of protein and fat are consumed” [8-11]. Even so, despite seemingly safe and increasingly popular, the long-term effects of a truly “zero” carbohydrate diet (without micronutrient supplementation) are difficult to ascertain through controlled experimentation, being only inferred from evolutionary biology, observational studies, and mechanistic data [12-14]. With this caveat, it has now been clearly established in large cohorts of patients, both adult and pediatric, that the oral intake of carbohydrates can be chronically very low (< 5-10% of total daily energy), as long as essential micronutrients are obtained from the underlying food selection and/or supplementation [15-18].

During fasting or in the absence of dietary carbohydrates, a steady-state euglycemia will be maintained in a low but physiological range via hepatic and renal gluconeogenesis from endogenous sources, such as lactate, fatty acids (glycerol), gluconeogenic amino acids and odd chain fatty acids [19-21]. From an evolutionary perspective, a minimal threshold of gluconeogenesis was preserved even after indefinite periods of fasting, questioning whether glucose *itself* is essential [22, 23]. It was not until the seminal work of Cahill *et al.* corroborating the remarkable metabolic flexibility of human physiology that the absolute requirements of glucose under compensatory ketosis could be quantified [24]. Drenick *et al.* demonstrated that, after a 2-month fast in obese subjects, insulin stimulation failed to precipitate hypoglycemic reactions with plasma glucose as low as 9 mg/dl (0.5 mM) [25]. During prolonged fasting, blood glucose levels below 30 mg/dL (1.70 mM) have been sustained continuously for several months without adverse effects [22, 23]. It is apparent that glucose requirements can be significantly displaced by fat-derived fuels, assuming a gradual period of ketogenic adaptation proportional to the degree of glucose depletion [26, 27]. Most human tissues require at least 1 to 4 weeks of strict KD adherence for the effective upregulation of ketone body metabolism, a process that can be accelerated through water-only fasting [28-30]. Without ketogenic adaptation, glucose is indeed the “primary metabolic fuel”, as evidenced by hypoglycemic reactions after accidental secretagogue or insulin overdose in diabetic patients following carbohydrate-rich diets, even under conditions of diabetic ketoacidosis [31-33].

Many clinicians fear *ketosis* due to confusion with diabetic ketoacidosis, defined by the triad of excessive ketogenesis, metabolic acidosis and concomitant hyperglycemia [34]. A low level of ketones (e.g., < 0.5 mM) prior to initiating carbohydrate restriction indicates that the individual is likely not deficient in insulin and therefore not at risk for ketoacidosis [35]. Clinicians may wish to monitor serum bicarbonate during the early stages of ketogenic adaptation. Ketoacidosis does not occur unless ketones coexist with hyperglycemia and decreasing bicarbonate levels, indicating insulin insufficiency (not to be confused with insulin suppression via carbohydrate restriction, which in turn increases insulin sensitivity, as indicated by lower insulin requirements for euglycemia) [36].

In the context of KMT, evolutionary competition for the limited nutrient supply between the tumor and normal tissues may be potentiated [37]. It is important to clarify that the uninterrupted maintenance of very low glucose levels (< 3 mM) is not realistically achievable for most patients following isocaloric KDs and typically requires prolonged fasting or pharmacological interventions. Fortunately, the anti-tumoral benefits of KMT are hypothesized to arise from pleiotropic regulation of energy sensing and growth signaling pathways (PI3K, AKT, AMPK/mTOR, PGC-1α), inflammation, angiogenesis, and autophagy, not solely as the result of reduced glucose availability with compensatory ketosis, which simply serves as a surrogate marker for successful clinical implementation [38-43].

### Appendix 2. Metabolic stratification: SLP/OXPHOS.

In preclinical models, the restriction of glycolysis and glutaminolysis reduces tumor growth regardless of whether uptake is directed towards fermentation, biosynthesis, or oxidation. In flux analysis, a mixed readout from cytosolic SLP (estimated by proton extrusion) and OXPHOS (estimated by oxygen consumption) will be detected in most tumor samples, with additional changes by *in vitro* culturing [44-46]. It is important to note, however, that respirometry and flux analysis (OCR/ECAR), as well as metabolite labeling, may not be translationally meaningful markers of OXPHOS beyond short-term experimental endpoints (e.g., 24-72 h). For example, a significant increase in OCR in response to βHB has been detected *in vitro* in a subtype of breast cancer, but βHB had no effect on long-term proliferation under physiological glucose concentrations [47]. Similarly, the magnitude of transient fatty acid oxidation or ketone body utilization should be weighed against the anti-proliferative effects of SLP targeting (e.g., restriction of glycolytic and glutaminolytic flux) [48, 49]. In the presence of SLP fuels, metabolomic profiling and tracing studies typically show TCA cycle labeling with almost any desired oxidative substrate (e.g., glutamine, lactate, fatty acids, ketone bodies), without offering a comparative definition of the tumor's absolute bioenergetic dependencies under press-pulse metabolic targeting [44, 50-53]. In the context of KMT, it will become important to characterize the long-term proliferative capacity under the relative inhibition of SLP flux, either via dietary interventions and/or metabolic inhibitors, as well as the associated mitochondrial retrograde signaling and resource competition with normal tissues. Thus, if metabolic stratification is performed in patient-derived *in vitro* or *ex vivo* models, or *in vivo* preclinical metabolite tracing studies, cancer cell oxidative capacity (such as ketolysis) should be evaluated after inducing a comparable degree of glycolysis and glutaminolysis targeting, mirroring the intended therapeutic context. From this perspective, uncovering secondary adaptative pathways could guide subsequent metabolic therapies.

It is pertinent to briefly discuss the SLP/OXPHOS dichotomy, as the assumption of fully functional OXPHOS in cancer cells would imply their rescue via oxidative metabolism [54, 55]. The mitochondrial metabolic theory of cancer proposes that all cancer cells are vulnerable to metabolic targeting due to inherent SLP dependency, which arises as a consequence of necessary and sufficient impairment of mitochondrial respiration [56, 57]. In practical terms, most malignant cells appear unable to sustain proliferation after the simultaneous inhibition of the glycolytic and glutaminolytic pathways, regardless of residual OXPHOS [58-63]. Likewise, the biosynthetic and redox requirements of rapid proliferation may favor increased SLP flux, opening an avenue for therapeutic interventions [64]. This observation is not incompatible with recent methodological efforts to quantify ancillary pathways that contribute to tumor growth, as long as SLP flux is uninterrupted, such as TCA cycle turnover via fatty acids, ketone bodies, lactate, and other acetyl-CoA-yielding substrates, catabolism of amino acids, fermentation of non-canonical metabolites (e.g., uridine-derived ribose), the pentose phosphate pathway, fatty acid/cholesterol synthesis, redox homeostasis or autophagy [65-74]. However, none of these pathways have been shown to sustain tumor proliferation in the context of effective SLP targeting, even though they can be “technically” quantified in short endpoint experiments, which has led to widespread confusion in data interpretation [75-80].

Indeed, mitochondrial metabolism can be essential to facilitate proliferation in the presence of sufficient SLP flux (e.g., contributing to biosynthesis, ATP and redox balance), but, unlike in normal cells, it appears insufficient for the long-term maintenance of bioenergetic functions [81-85]. It is exceedingly difficult to ascertain operational parameters of mitochondrial sufficiency over short experimental endpoints [44]. To the extent of our knowledge, no continuously proliferating tumorigenic cells have been described as supported exclusively via OXPHOS when deprived of both glycolysis and glutaminolysis (e.g., compensating for SLP and biosynthetic restriction via ketone, fatty acid, or lactate oxidation). In fact, given the pervasive claims that “oxidative metabolism persists in the tumour and may exceed that in adjacent non-malignant tissue” [86], as well as the existence of a “rapid metabolic shift of tumor cells towards OXPHOS when the Warburg effect is abrogated” [87], it should be relatively common to find hypothetical OXPHOS-driven cancer cells, supporting high proliferation rates by non-fermentable fuels after complete SLP inhibition. While attempts have been made to develop experimental models more reliant on OXPHOS by genetic ablation of glycolytic enzymes, the impact of targeting glycolysis and glutaminolysis and the comparative tumorigenic potential of stable oxidative clones has not been fully explored [88-90]. The fact that this phenotype is elusive could suggest a tangible biological constraint on cell growth that is not captured by prevailing theories of metabolic plasticity, perhaps in the absolute degree of respiratory sufficiency, either by loss of function or increased demand, as originally proposed by Warburg [91].

In contrast with this binary perspective, a recent computational approach identified several distinct transcriptional subtypes in GBM, including a “mitochondrial subtype” that has been repeatedly cited as reliant “exclusively on oxidative phosphorylation for energy production” [92, 93]. However, this study did not address functionally relevant parameters of respiration by means of long-term proliferation of “mitochondrial” GBM clones under escalating SLP targeting. Consequently, short-term changes in OCR following substrate addition or electron transport chain inhibition are frequently interpreted as “normal” OXPHOS [94], which may lead to incorrect assumptions about respiratory sufficiency. Indeed, given the apparent favorable influence on clinical outcomes of such mitochondrial subtypes, complete enrichment in “OXPHOS-driven tumor cells” (lacking glycolytic/plurimetabolic populations, including glutaminolysis) may no longer fit the definition of cancer. For example, slow growing tumors typically exhibit lower SLP flux and higher residual OXPHOS than more aggressive tumors [89, 95, 96]. Even if we assume the prevailing view that "glycolysis in most tumours is upregulated without mitochondrial dysfunction" and "oxidative phosphorylation continues normally" [65], SLP dependency remains the distinctive metabolic feature of cancer.

### Appendix 3. Pharmacological targeting of the tumor microenvironment and cancer-associated pathways.

Mebendazole (MBZ) is an anti-helminthic drug with rekindled interest due to promising antineoplastic effects against GBM and other cancers [97-99]. MBZ acts by inhibiting tubulin polymerization and glucose transport [100]. Combinatory protocols and chronic dosing for cancer typically ranges from 10 to 50 mg/kg/day [101, 102]. Due to its excellent safety profile, dose-escalation and tolerability studies in adults have evaluated up to 200 mg/kg/day without dose‐limiting toxicities, although monitoring of liver function is advisable at higher doses [97, 103]. Ongoing trials in pediatric gliomas are testing doses from 50 to 200 mg/kg/day, in combination with chemoradiotherapy, for up to 70 weeks in low-grade glioma and 48 weeks in high-grade glioma/pontine glioma (NCT01837862). MBZ should be taken with a fatty vehicle for improved bioavailability (e.g., a meal consisting of at least 15-20 g fat), preferably in the context of dietary KMT [104, 105]. Pharmacokinetics can be variable depending on the dosing schedule, likely requiring continued daily treatment to reach optimal serum concentrations, which has sparked interest in different MBZ polymorphs and prodrugs, as well as other benzimidazoles [99, 106-110]. An over-the-counter alternative obtained off-label by cancer patients is fenbendazole [111]. Fenbendazole has been tested in small human studies at variable dosing, with a safety profile comparable to MBZ, but it never received regulatory approval for clinical use [112-114]. Consequently, the quality of commercial brands can be unpredictable as they are not intended for human consumption [115]. Even though the effects of fenbendazole appear similar to MBZ in preclinical models and case reports [116-119], additional clinical research would be needed to establish pharmacokinetic and pharmacodynamic equivalency or superiority for different tumor subtypes [120].

MBZ could be combined with other anti-protozoal/anti-helminthic, anti-fungal and/or antibiotic drugs [121-123]. These categories are receiving increasing attention for drug repurposing in cancer, including hydroxychloroquine [124], nitazoxanide [125], levamisole [126], pyrvinium pamoate [127] and praziquantel [128], as well itraconazole [129] and doxycycline [130]. Clinical trials for several malignancies are underway (**Additional File 6: Table S3**). It is important to note that interference with tumor growth is unlikely to arise from resolving an active or latent infection, but rather via interaction with shared cancer-associated pathways that are either partially known, as with chloroquine/autophagy [131], itraconazole/angiogenesis [132] and doxycycline/stemness [133], or remain largely unknown. Most of these compounds are safe in isolation but their optimal dosing for cancer is unclear, particularly for CNS tumors where blood-brain barrier permeability is a limiting factor [134, 135]. After single agent trials with SOC are completed, if favorable, combinatory protocols could be established based on tolerability, safety, mechanism of action and patient stratification [102, 136]. For example, specific mutations in high-grade gliomas (such as BRAF V600 or BRAF fusion/duplication) may benefit from autophagy inhibition via hydroxychloroquine (NCT04201457), further potentiated by KD-R and fasting [137, 138].

Hyperbaric oxygen therapy (HBOT) is a systemic treatment that involves breathing a high concentration of oxygen (usually 90-100%) inside a pressurized chamber with elevated barometric pressure (1.5 to 3.0 atmospheres absolute; ATA). It has been suggested that HBOT reverses tumor hypoxia and generates ROS to selectively kill cancer cells, synergizing with radiotherapy and certain chemotherapeutics which also act through an oxidative stress mechanism [139-142]. Most clinical studies in GBM administered HBOT at 1.5-2.5 ATA, with 60-120 min sessions, 3-5 days a week [143-145], although mild HBOT is also under evaluation for improved tolerability and availability [146]. Relative and absolute contraindications of HBOT are well characterized from longstanding historical application for decompression sickness and wound healing [147, 148]. One of the very rare but preventable side effects is oxygen-induced excitotoxicity resulting in CNS oxygen toxicity seizures [149]. Ideally, HBOT would be initiated after transitioning into therapeutic ketosis for at least several weeks to benefit from its antiepileptic properties; however, ketone-generating MCTs or exogenous ketones can be supplemented for their acute neuroprotective effects at preventing CNS oxygen toxicity seizures [150, 151]. In this category, we also highlight hyperthermia, ozone, and focused ultrasound as emerging therapies to leverage the reduced adaptive versatility of cancer cells under metabolic pressure [152-154]. Despite the fact that the brain is not an easily accessible anatomical enclave, multiple hyperthermia modalities have been developed for GBM [155, 156], as well as proof-of-concept intratumoral oxygen-ozone reservoirs [157].

Dichloroacetate (DCA), an over-the-counter PDK inhibitor, can further increase mitochondrial oxidative stress in tumor cells [158, 159]. DCA has been tested in several pilot clinical trials that demonstrated safety and *in vivo* activity against solid tumors, including GBM [160-163]. Although DCA is marketed as a non-regulated research chemical, it was originally developed as a pharmacological agent. Therefore, it is crucial to seek evidence-based resources for appropriate clinical use, and source DCA from a reliable supplier [164]. Given the growing off-label use by cancer patients, it is important to inform the treating physician and discuss the relevant scientific literature and potential drug interactions [165-170]. The minimum effective daily dose should be determined gradually (with typical dose escalation from 6.25 to 15-25 mg/kg/day, maintained for 2 weeks on and 1 week off), accompanied with empirical supplementation to mitigate side effects (e.g., thiamine or benfotiamine, R-alpha lipoic acid, and acetyl L-carnitine) [171, 172].

Vitamin C has been studied for its potential anti-cancer benefits since the pioneering work of Linus Pauling in the 1970s and is now recognized as a more conventional coadjuvant therapy for targeting redox metabolism [173-175]. Given that only high concentrations are hypothesized to effectively increase oxidative stress in tumor cells, intravenous administration is preferred: maximum oral dosing results in plasma concentrations of less than 300 µM, whereas infusion increases levels up to 20-30 mM [176]. Most cancer studies have tested dosing schedules up to 1.5 g/kg, 2 or 3 times per week, for several weeks or months, which can be adjusted to desired peak plasma concentrations [177, 178]. Before administering high-dose vitamin C, patients should be screened for risk factors (such as G6PD deficiency), drug interactions, and antineoplastic treatments that may elicit antagonistic effects [179, 180]. Vitamin C may function as a glucose antagonist [181], interfering with the electrochemical methodology for glucose monitoring, including CGM sensors [182, 183].

Finally, it is worth mentioning disulfiram, an ALDH inhibitor and ROS inducer that has been proposed in several drug repurposing protocols for GBM and other cancers [136, 184]. Its cytotoxic effects appear to be copper-dependent, which has motivated parallel copper supplementation in clinical studies (e.g., 2-8 mg elemental copper per day) [185-187]. Disulfiram is contraindicated alongside HBOT due to increased risk of oxygen toxicity [147].

Nonsteroidal anti-inflammatory drugs (NSAIDs), including non-selective (e.g., aspirin) and COX-2 specific inhibitors (e.g., “coxibs”, such as celecoxib, as well as indomethacin, diclofenac, ketoprofen, or ibuprofen), may be beneficial for the short-term management of tumor inflammation [188]. The specific examples listed above have been shown to permeate into the CNS in humans, although other NSAIDs may also exhibit acceptable brain/plasma ratios based on animal studies [189-193]. In the case of GBM, dosing and treatment duration should be directed at reducing brain inflammation and corticosteroid needs [194, 195]. The KD itself exhibits broad anti-inflammatory properties through pleiotropic mechanisms, which may further alleviate neuroinflammation [196-198].

Tumor angiogenesis is another potential target for drug repurposing [132, 199-204]. **Additional File 6: Table S3** includes compounds with secondary anti-angiogenic properties, which could be further augmented by small molecules, endogenous peptides such as endostatin and specific VEGF targeting [205-208]. Bevacizumab is typically applied in a palliative context, and while evidence suggesting improvements in mOS is lacking [209, 210] and a detrimental role in facilitating tumor invasion has been reported [211-213], future research may explore whether low-dose regimens synergize with the antiangiogenic properties of dietary KMT [214-217]. Tetrathiomolybdate, an orphan drug for Wilson's disease, has been suggested as a direct inhibitor of angiogenesis for its ability to deplete systemic copper without affecting other copper-dependent cellular processes [218]. Interestingly, therapeutic efficacy could be further enhanced by relative glucose deprivation [219]. Encouraging outcomes from breast cancer highlight this mechanism as a viable alternative to anti-VEGF antibodies, potentially avoiding the drawbacks of increased distal invasion [213]. However, the narrow therapeutic window demands active monitoring (e.g., serum ceruloplasmin), which could be resource-intensive in outpatient settings [220].

In conclusion, an extensive array of clinically available drugs has been proposed for GBM therapy, either alone or in combination, including captopril, sertraline, artesunate, ritonavir/nelfinavir, aprepitant and atorvastatin [102, 136], quinacrine [221], pimozide, risperidone and paliperidone [222], proscillaridin A [223], AM404 (a paracetamol metabolite) [224], thioridazine [225], flupenthixol [226], cyclosporin A [227], methadone [228], donepezil [229], nicardipine [230], mibefradil [231], prazosin [232], apomorphine [233], nimodipine [234], chloramphenicol [235], idebenone [236], as well as other off-label and emerging indications at variable stages of clinical development [237-240]. This list could be further expanded to include drug repurposing and supplementation for extra-neural cancers [237, 241, 242].

It is important to reiterate that these compounds should be considered on a case-by-case basis within a dietary KMT framework with simultaneous targeting of glycolysis and glutaminolysis. Each potential combination requires a risk/benefit assessment and a strong mechanistic rationale for preferential action on cancer cells; for example, inhibition of the mevalonate pathway using statins may exert anti-tumoral effects but has been noted to alter normal cell function, and thus may need to be regarded as a “pulse” rather than a “press” [243-245]. Analogous to standard cytotoxic agents and immunotherapies, it is our view that inhibiting cancer-associated pathways will be more effective once applied to an already metabolically-weakened population of cancer cells, making side effects and resistance to therapy less likely.

**References:**

1. Nakrani MN, Wineland RH, Anjum F: **Physiology, Glucose Metabolism**. *StatPearls* 2022.

2. Mathew P, Thoppil D: **Hypoglycemia**. In: *StatPearls [Internet].* edn.: StatPearls Publishing; 2022.

3. Ritter S: **Monitoring and maintenance of brain glucose supply: importance of hindbrain catecholamine neurons in this multifaceted task**. *Appetite Food Intake* 2017:177-204.

4. Mergenthaler P, Lindauer U, Dienel GA, Meisel A: **Sugar for the brain: the role of glucose in physiological and pathological brain function**. *Trends Neurosci* 2013, **36**(10):587-597.

5. Luz MR, de Oliveira GA, de Sousa CR, Da Poian AT: **Glucose as the sole metabolic fuel: The possible influence of formal teaching on the establishment of a misconception about energy‐yielding metabolism among students from Rio de Janeiro, Brazil**. *Biochemistry molecular biology education* 2008, **36**(6):407-416.

6. Ghosh A, Cheung YY, Mansfield BC, Chou JY: **Brain contains a functional glucose-6-phosphatase complex capable of endogenous glucose production**. *J Biol Chem* 2005, **280**(12):11114-11119.

7. Brosnan JT: **Comments on metabolic needs for glucose and the role of gluconeogenesis**. *Eur J Clin Nutr* 1999, **53 Suppl 1**(1):S107-111.

8. Lupton JR, Brooks J, Butte N, Caballero B, Flatt J, Fried S: **Dietary reference intakes for energy, carbohydrate, fiber, fat, fatty acids, cholesterol, protein, and amino acids**. *National Academy Press: Washington, DC, USA* 2002, **5**:589-768.

9. Landry MJ, Crimarco A, Gardner CD: **Benefits of Low Carbohydrate Diets: a Settled Question or Still Controversial?** *Curr Obes Rep* 2021, **10**(3):409-422.

10. Goldenberg JZ, Day A, Brinkworth GD, Sato J, Yamada S, Jonsson T, Beardsley J, Johnson JA, Thabane L, Johnston BC: **Efficacy and safety of low and very low carbohydrate diets for type 2 diabetes remission: systematic review and meta-analysis of published and unpublished randomized trial data**. *BMJ* 2021, **372**:m4743.

11. Lennerz BS, Mey JT, Henn OH, Ludwig DS: **Behavioral Characteristics and Self-Reported Health Status among 2029 Adults Consuming a "Carnivore Diet"**. *Curr Dev Nutr* 2021, **5**(12):nzab133.

12. Ben-Dor M, Sirtoli R, Barkai R: **The evolution of the human trophic level during the Pleistocene**. *American journal of physical anthropology* 2021, **175 Suppl 72**:27-56.

13. O’Hearn A: **Can a carnivore diet provide all essential nutrients?** *Current Opinion in Endocrinology, Diabetes Obesity* 2020, **27**(5):312-316.

14. Klement RJ: **Was there a need for high carbohydrate content in Neanderthal diets?** *American Journal of Biological Anthropology* 2022, **179**(4):668-677.

15. Westman EC: **Is dietary carbohydrate essential for human nutrition?** *Am J Clin Nutr* 2002, **75**(5):951-953.

16. O’Hearn A, Westman EC, Yancy WS, Wellington N: **Nutritional aspects**. In: *Ketogenic.* edn. Edited by Noakes TD, Murphy T, Wellington N, Kajee H, Rice SM: Academic Press; 2023: 71-104.

17. Martin-McGill KJ, Bresnahan R, Levy RG, Cooper PN: **Ketogenic diets for drug-resistant epilepsy**. *Cochrane Database Syst Rev* 2020, **6**(6):CD001903.

18. Hagstrom H, Hagfors LN, Tellstrom A, Hedelin R, Lindmark K: **Low carbohydrate high fat-diet in real life assessed by diet history interviews**. *Nutr J* 2023, **22**(1):14.

19. Chourpiliadis C, Mohiuddin SS: **Biochemistry, gluconeogenesis**. In: *StatPearls [Internet].* edn.: StatPearls Publishing; 2021.

20. Taherizadeh M, Khoshnia M, Shams S, Hesari Z, Joshaghani H: **Clinical Significance of Plasma Levels of Gluconeogenic Amino Acids in Esophageal Cancer Patients**. *Asian Pac J Cancer Prev* 2020, **21**(8):2463-2468.

21. Rothman DL, Magnusson I, Katz LD, Shulman RG, Shulman GI: **Quantitation of hepatic glycogenolysis and gluconeogenesis in fasting humans with 13C NMR**. *Science* 1991, **254**(5031):573-576.

22. Stewart WK, Fleming LW: **Features of a successful therapeutic fast of 382 days' duration**. *Postgraduate medical journal* 1973, **49**(569):203-209.

23. Thomson TJ, Runcie J, Miller V: **Treatment of obesity by total fasting for up to 249 days**. *Lancet* 1966, **2**(7471):992-996.

24. Cahill GF, Jr.: **Fuel metabolism in starvation**. *Annual review of nutrition* 2006, **26**:1-22.

25. Drenick EJ, Alvarez LC, Tamasi GC, Brickman ASJTJoci: **Resistance to symptomatic insulin reactions after fasting**. 1972, **51**(10):2757-2762.

26. Longo R, Peri C, Cricri D, Coppi L, Caruso D, Mitro N, De Fabiani E, Crestani M: **Ketogenic Diet: A New Light Shining on Old but Gold Biochemistry**. *Nutrients* 2019, **11**(10):2497.

27. Zhang Y, Kuang Y, Xu K, Harris D, Lee Z, LaManna J, Puchowicz MA: **Ketosis proportionately spares glucose utilization in brain**. *J Cereb Blood Flow Metab* 2013, **33**(8):1307-1311.

28. Burke LM, Whitfield J, Heikura IA, Ross ML, Tee N, Forbes SF, Hall R, McKay AK, Wallett AM, Sharma AP: **Adaptation to a low carbohydrate high fat diet is rapid but impairs endurance exercise metabolism and performance despite enhanced glycogen availability**. *The Journal of Physiology* 2021, **599**(3):771-790.

29. Phinney SD, Bistrian BR, Wolfe RR, Blackburn GL: **The human metabolic response to chronic ketosis without caloric restriction: physical and biochemical adaptation**. *Metabolism* 1983, **32**(8):757-768.

30. Kackley ML, Brownlow ML, Buga A, Crabtree CD, Sapper TN, O'Connor A, Volek JS: **The effects of a 6-week controlled, hypocaloric ketogenic diet, with and without exogenous ketone salts, on cognitive performance and mood states in overweight and obese adults**. *Front Neurosci* 2022, **16**:971144.

31. Pathak RD, Schroeder EB, Seaquist ER, Zeng C, Lafata JE, Thomas A, Desai J, Waitzfelder B, Nichols GA, Lawrence JM *et al*: **Severe Hypoglycemia Requiring Medical Intervention in a Large Cohort of Adults With Diabetes Receiving Care in U.S. Integrated Health Care Delivery Systems: 2005-2011**. *Diabetes Care* 2016, **39**(3):363-370.

32. Chantzaras A, Yfantopoulos J: **Evaluating the Incidence and Risk Factors Associated With Mild and Severe Hypoglycemia in Insulin-Treated Type 2 Diabetes**. *Value Health Reg Issues* 2022, **30**:9-17.

33. Ben-Ami H, Nagachandran P, Mendelson A, Edoute Y: **Drug-induced hypoglycemic coma in 102 diabetic patients**. *Archives of internal medicine* 1999, **159**(3):281-284.

34. Dhatariya KK, Glaser NS, Codner E, Umpierrez GE: **Diabetic ketoacidosis**. *Nat Rev Dis Primers* 2020, **6**(1):40.

35. Cooper ID, Brookler KH, Kyriakidou Y, Elliott BT, Crofts CAJB: **Metabolic phenotypes and step by step evolution of type 2 diabetes: A New paradigm**. 2021, **9**(7):800.

36. Yuan X, Wang J, Yang S, Gao M, Cao L, Li X, Hong D, Tian S, Sun C: **Effect of the ketogenic diet on glycemic control, insulin resistance, and lipid metabolism in patients with T2DM: a systematic review and meta-analysis**. *Nutr Diabetes* 2020, **10**(1):38.

37. McCall AL, Fixman LB, Fleming N, Tornheim K, Chick W, Ruderman NB: **Chronic hypoglycemia increases brain glucose transport**. *Am J Physiol* 1986, **251**(4 Pt 1):E442-447.

38. Mukherjee P, El-Abbadi MM, Kasperzyk JL, Ranes MK, Seyfried TN: **Dietary restriction reduces angiogenesis and growth in an orthotopic mouse brain tumour model**. *Br J Cancer* 2002, **86**(10):1615-1621.

39. Mukherjee P, Mulrooney TJ, Marsh J, Blair D, Chiles TC, Seyfried TN: **Differential effects of energy stress on AMPK phosphorylation and apoptosis in experimental brain tumor and normal brain**. *Mol Cancer* 2008, **7**:37.

40. Mulrooney TJ, Marsh J, Urits I, Seyfried TN, Mukherjee P: **Influence of caloric restriction on constitutive expression of NF-kappaB in an experimental mouse astrocytoma**. *PLoS ONE* 2011, **6**(3):e18085.

41. Shelton LM, Huysentruyt LC, Mukherjee P, Seyfried TN: **Calorie restriction as an anti-invasive therapy for malignant brain cancer in the VM mouse**. *ASN Neuro* 2010, **2**(3):e00038.

42. Zhou W, Mukherjee P, Kiebish MA, Markis WT, Mantis JG, Seyfried TN: **The calorically restricted ketogenic diet, an effective alternative therapy for malignant brain cancer**. *Nutr Metab (Lond)* 2007, **4**:5.

43. Wong W: **Mitochondrial fission fueled by fasting**. *Sci Signal* 2023, **16**(797):eadk1008.

44. Duraj T, Carrion-Navarro J, Seyfried TN, Garcia-Romero N, Ayuso-Sacido A: **Metabolic therapy and bioenergetic analysis: The missing piece of the puzzle**. *Mol Metab* 2021, **54**:101389.

45. Desousa BR, Kim KK, Jones AE, Ball AB, Hsieh WY, Swain P, Morrow DH, Brownstein AJ, Ferrick DA, Shirihai OS *et al*: **Calculation of ATP production rates using the Seahorse XF Analyzer**. *EMBO Rep* 2023, **24**(10):e56380.

46. Altea-Manzano P, Cuadros AM, Broadfield LA, Fendt SM: **Nutrient metabolism and cancer in the in vivo context: a metabolic game of give and take**. *EMBO Rep* 2020, **21**(10):e50635.

47. Bartmann C, Janaki Raman SR, Floter J, Schulze A, Bahlke K, Willingstorfer J, Strunz M, Wockel A, Klement RJ, Kapp M *et al*: **Beta-hydroxybutyrate (3-OHB) can influence the energetic phenotype of breast cancer cells, but does not impact their proliferation and the response to chemotherapy or radiation**. *Cancer Metab* 2018, **6**(1):8.

48. Sperry J, Condro MC, Guo L, Braas D, Vanderveer-Harris N, Kim KK, Pope WB, Divakaruni AS, Lai A, Christofk HJI: **Glioblastoma utilizes fatty acids and ketone bodies for growth allowing progression during ketogenic diet therapy**. 2020, **23**(9).

49. Javier R, Wang W, Drumm M, McCortney K, Sarkaria JN, Horbinski C: **The efficacy of an unrestricted cycling ketogenic diet in preclinical models of IDH wild-type and IDH mutant glioma**. *PLoS ONE* 2022, **17**(2):e0257725.

50. Hui S, Ghergurovich JM, Morscher RJ, Jang C, Teng X, Lu W, Esparza LA, Reya T, Le Z, Yanxiang Guo J *et al*: **Glucose feeds the TCA cycle via circulating lactate**. *Nature* 2017, **551**(7678):115-118.

51. De Feyter HM, Behar KL, Rao JU, Madden-Hennessey K, Ip KL, Hyder F, Drewes LR, Geschwind J-F, De Graaf RA, Rothman DL: **A ketogenic diet increases transport and oxidation of ketone bodies in RG2 and 9L gliomas without affecting tumor growth**. *Neuro Oncol* 2016, **18**(8):1079-1087.

52. Pachnis P, Wu Z, Faubert B, Tasdogan A, Gu W, Shelton S, Solmonson A, Rao AD, Kaushik AK, Rogers TJ *et al*: **In vivo isotope tracing reveals a requirement for the electron transport chain in glucose and glutamine metabolism by tumors**. *Sci Adv* 2022, **8**(35):eabn9550.

53. Bartman CR, Faubert B, Rabinowitz JD, DeBerardinis RJ: **Metabolic pathway analysis using stable isotopes in patients with cancer**. *Nat Rev Cancer* 2023, **23**(12):863-878.

54. Berrington A, Schreck KC, Barron BJ, Blair L, Lin DDM, Hartman AL, Kossoff E, Easter L, Whitlow CT, Jung Y *et al*: **Cerebral Ketones Detected by 3T MR Spectroscopy in Patients with High-Grade Glioma on an Atkins-Based Diet**. *AJNR Am J Neuroradiol* 2019, **40**(11):1908-1915.

55. Wenger KJ, Wagner M, Harter PN, Franz K, Bojunga J, Fokas E, Imhoff D, Rodel C, Rieger J, Hattingen E *et al*: **Maintenance of Energy Homeostasis during Calorically Restricted Ketogenic Diet and Fasting-MR-Spectroscopic Insights from the ERGO2 Trial**. *Cancers (Basel)* 2020, **12**(12):3549.

56. Warburg O: **On respiratory impairment in cancer cells**. *Science* 1956, **124**(3215):269-270.

57. Seyfried TN, Chinopoulos C: **Can the mitochondrial metabolic theory explain better the origin and management of cancer than can the somatic mutation theory?** *Metabolites* 2021, **11**(9):572.

58. Ocana MC, Martinez-Poveda B, Quesada AR, Medina MA: **Highly Glycolytic Immortalized Human Dermal Microvascular Endothelial Cells are Able to Grow in Glucose-Starved Conditions**. *Biomolecules* 2019, **9**(8):332.

59. Shajahan-Haq AN, Cook KL, Schwartz-Roberts JL, Eltayeb AE, Demas DM, Warri AM, Facey CO, Hilakivi-Clarke LA, Clarke R: **MYC regulates the unfolded protein response and glucose and glutamine uptake in endocrine resistant breast cancer**. *Mol Cancer* 2014, **13**(1):239.

60. Ocana MC, Martinez-Poveda B, Quesada AR, Medina MA: **Glucose Favors Lipid Anabolic Metabolism in the Invasive Breast Cancer Cell Line MDA-MB-231**. *Biology (Basel)* 2020, **9**(1):16.

61. Kaadige MR, Looper RE, Kamalanaadhan S, Ayer DE: **Glutamine-dependent anapleurosis dictates glucose uptake and cell growth by regulating MondoA transcriptional activity**. *Proc Natl Acad Sci U S A* 2009, **106**(35):14878-14883.

62. Song Z, Wei B, Lu C, Li P, Chen L: **Glutaminase sustains cell survival via the regulation of glycolysis and glutaminolysis in colorectal cancer**. *Oncol Lett* 2017, **14**(3):3117-3123.

63. Ahmadiankia N: **In vitro and in vivo studies of cancer cell behavior under nutrient deprivation**. *Cell Biol Int* 2020, **44**(8):1588-1597.

64. Scott AJ, Mittal A, Meghdadi B, Palavalasa S, Achreja A, O'Brien A, Kothari AU, Zhou W, Xu J, Lin A *et al*: **Rewiring of cortical glucose metabolism fuels human brain cancer growth**. *medRxiv* 2023:2023.2010. 2024.23297489.

65. Vaupel P, Multhoff G: **Revisiting the Warburg effect: historical dogma versus current understanding**. *J Physiol* 2021, **599**(6):1745-1757.

66. Wu H, Ding Z, Hu D, Sun F, Dai C, Xie J, Hu X: **Central role of lactic acidosis in cancer cell resistance to glucose deprivation-induced cell death**. *J Pathol* 2012, **227**(2):189-199.

67. Skinner OS, Blanco-Fernandez J, Goodman RP, Kawakami A, Shen H, Kemeny LV, Joesch-Cohen L, Rees MG, Roth JA, Fisher DE *et al*: **Salvage of ribose from uridine or RNA supports glycolysis in nutrient-limited conditions**. *Nat Metab* 2023, **5**(5):765-776.

68. Tsai PY, Lee MS, Jadhav U, Naqvi I, Madha S, Adler A, Mistry M, Naumenko S, Lewis CA, Hitchcock DS *et al*: **Adaptation of pancreatic cancer cells to nutrient deprivation is reversible and requires glutamine synthetase stabilization by mTORC1**. *Proc Natl Acad Sci U S A* 2021, **118**(10):e2003014118.

69. Luengo A, Li Z, Gui DY, Sullivan LB, Zagorulya M, Do BT, Ferreira R, Naamati A, Ali A, Lewis CA *et al*: **Increased demand for NAD(+) relative to ATP drives aerobic glycolysis**. *Mol Cell* 2021, **81**(4):691-707 e696.

70. Klement RJ, Koebrunner PS: **Comments on "Inhibition of the ketolytic acetyl CoA supply to tumors could be their 'Achilles heel'"**. *Int J Cancer* 2020, **147**(11):3262-3263.

71. Singh B, Shamsnia A, Raythatha MR, Milligan RD, Cady AM, Madan S, Lucci A: **Highly adaptable triple-negative breast cancer cells as a functional model for testing anticancer agents**. *PLoS ONE* 2014, **9**(10):e109487.

72. Hosios AM, Hecht VC, Danai LV, Johnson MO, Rathmell JC, Steinhauser ML, Manalis SR, Vander Heiden MG: **Amino Acids Rather than Glucose Account for the Majority of Cell Mass in Proliferating Mammalian Cells**. *Developmental cell* 2016, **36**(5):540-549.

73. Wang Y, Stancliffe E, Fowle-Grider R, Wang R, Wang C, Schwaiger-Haber M, Shriver LP, Patti GJ: **Saturation of the mitochondrial NADH shuttles drives aerobic glycolysis in proliferating cells**. *Mol Cell* 2022, **82**(17):3270-3283 e3279.

74. Miska J, Chandel NS: **Targeting fatty acid metabolism in glioblastoma**. *J Clin Invest* 2023, **133**(1).

75. Ma Y, Temkin SM, Hawkridge AM, Guo C, Wang W, Wang XY, Fang X: **Fatty acid oxidation: An emerging facet of metabolic transformation in cancer**. *Cancer Lett* 2018, **435**:92-100.

76. Torrini C, Nguyen TTT, Shu C, Mela A, Humala N, Mahajan A, Seeley EH, Zhang G, Westhoff MA, Karpel-Massler G *et al*: **Lactate is an epigenetic metabolite that drives survival in model systems of glioblastoma**. *Mol Cell* 2022, **82**(16):3061-3076 e3066.

77. Rodrigues LM, Uribe-Lewis S, Madhu B, Honess DJ, Stubbs M, Griffiths JR: **The action of beta-hydroxybutyrate on the growth, metabolism and global histone H3 acetylation of spontaneous mouse mammary tumours: evidence of a beta-hydroxybutyrate paradox**. *Cancer Metab* 2017, **5**:4.

78. Reitzer LJ, BM W: **Evidence that glutamine, not sugar, is the major energy source for cultured HeLa cells**. 1979.

79. Linker W, Loffler M, Schneider F: **Uridine, but Not Cytidine Can Sustain Growth of Ehrlich Ascites Tumor-Cells in Glucose-Deprived Medium with Altered Proliferation Kinetics**. *Eur J Cell Biol* 1985, **36**(2):176-181.

80. Wice BM, Reitzer LJ, Kennell D: **The continuous growth of vertebrate cells in the absence of sugar**. *J Biol Chem* 1981, **256**(15):7812-7819.

81. Maher EA, Marin‐Valencia I, Bachoo RM, Mashimo T, Raisanen J, Hatanpaa KJ, Jindal A, Jeffrey FM, Choi C, Madden C: **Metabolism of [U‐13C] glucose in human brain tumors in vivo**. *NMR in biomedicine* 2012, **25**(11):1234-1244.

82. Fan TW, Lane AN, Higashi RM, Farag MA, Gao H, Bousamra M, Miller DM: **Altered regulation of metabolic pathways in human lung cancer discerned by (13)C stable isotope-resolved metabolomics (SIRM)**. *Mol Cancer* 2009, **8**(1):41.

83. Tan AS, Baty JW, Dong LF, Bezawork-Geleta A, Endaya B, Goodwin J, Bajzikova M, Kovarova J, Peterka M, Yan B *et al*: **Mitochondrial genome acquisition restores respiratory function and tumorigenic potential of cancer cells without mitochondrial DNA**. *Cell Metab* 2015, **21**(1):81-94.

84. Martinez-Reyes I, Cardona LR, Kong H, Vasan K, McElroy GS, Werner M, Kihshen H, Reczek CR, Weinberg SE, Gao P *et al*: **Mitochondrial ubiquinol oxidation is necessary for tumour growth**. *Nature* 2020, **585**(7824):288-292.

85. Caldez MJ, Van Hul N, Koh HWL, Teo XQ, Fan JJ, Tan PY, Dewhurst MR, Too PG, Talib SZA, Chiang BE *et al*: **Metabolic Remodeling during Liver Regeneration**. *Developmental cell* 2018, **47**(4):425-438 e425.

86. DeBerardinis RJ, Chandel NS: **We need to talk about the Warburg effect**. *Nat Metab* 2020, **2**(2):127-129.

87. Cassim S, Vucetic M, Zdralevic M, Pouyssegur J: **Warburg and Beyond: The Power of Mitochondrial Metabolism to Collaborate or Replace Fermentative Glycolysis in Cancer**. *Cancers (Basel)* 2020, **12**(5):1119.

88. Hefzi H, Martinez-Monge I, Marin de Mas I, Cowie NL, Gomez Toledo A, Noh SM, Karottki KJlC, Decker M, Arnsdorf J, Camacho-Zaragoza JM: **Multiplex genome editing eliminates the Warburg Effect without impacting growth rate in mammalian cells**. *bioRxiv* 2024:2024.2008. 2002.606284.

89. Zdralevic M, Brand A, Di Ianni L, Dettmer K, Reinders J, Singer K, Peter K, Schnell A, Bruss C, Decking SM *et al*: **Double genetic disruption of lactate dehydrogenases A and B is required to ablate the "Warburg effect" restricting tumor growth to oxidative metabolism**. *J Biol Chem* 2018, **293**(41):15947-15961.

90. Fantin VR, St-Pierre J, Leder P: **Attenuation of LDH-A expression uncovers a link between glycolysis, mitochondrial physiology, and tumor maintenance**. *Cancer Cell* 2006, **9**(6):425-434.

91. Warburg OJS: **On the origin of cancer cells**. 1956, **123**(3191):309-314.

92. Garofano L, Migliozzi S, Oh YT, D'Angelo F, Najac RD, Ko A, Frangaj B, Caruso FP, Yu K, Yuan J *et al*: **Pathway-based classification of glioblastoma uncovers a mitochondrial subtype with therapeutic vulnerabilities**. *Nat Cancer* 2021, **2**(2):141-156.

93. Alzial G, Renoult O, Paris F, Gratas C, Clavreul A, Pecqueur C: **Wild-type isocitrate dehydrogenase under the spotlight in glioblastoma**. *Oncogene* 2022, **41**(5):613-621.

94. Schmidt CA, Fisher-Wellman KH, Neufer PD: **From OCR and ECAR to energy: Perspectives on the design and interpretation of bioenergetics studies**. *J Biol Chem* 2021, **297**(4):101140.

95. Seyfried TN, Arismendi-Morillo G, Mukherjee P, Chinopoulos C: **On the Origin of ATP Synthesis in Cancer**. *iScience* 2020, **23**(11):101761.

96. Pedersen PL: **Tumor mitochondria and the bioenergetics of cancer cells**. *Prog Exp Tumor Res* 1978, **22**:190-274.

97. Gallia GL, Holdhoff M, Brem H, Joshi AD, Hann CL, Bai RY, Staedtke V, Blakeley JO, Sengupta S, Jarrell TC *et al*: **Mebendazole and temozolomide in patients with newly diagnosed high-grade gliomas: results of a phase 1 clinical trial**. *Neurooncol Adv* 2021, **3**(1):vdaa154.

98. Meco D, Attinà G, Mastrangelo S, Navarra P, Ruggiero AJIJoMS: **Emerging Perspectives on the Antiparasitic Mebendazole as a Repurposed Drug for the Treatment of Brain Cancers**. 2023, **24**(2):1334.

99. Mansoori S, Fryknas M, Alvfors C, Loskog A, Larsson R, Nygren P: **A phase 2a clinical study on the safety and efficacy of individualized dosed mebendazole in patients with advanced gastrointestinal cancer**. *Sci Rep* 2021, **11**(1):8981.

100. Chai JY, Jung BK, Hong SJ: **Albendazole and Mebendazole as Anti-Parasitic and Anti-Cancer Agents: an Update**. *Korean J Parasitol* 2021, **59**(3):189-225.

101. Meco D, Attina G, Mastrangelo S, Navarra P, Ruggiero A: **Emerging Perspectives on the Antiparasitic Mebendazole as a Repurposed Drug for the Treatment of Brain Cancers**. *Int J Mol Sci* 2023, **24**(2):1334.

102. Agrawal S, Vamadevan P, Mazibuko N, Bannister R, Swery R, Wilson S, Edwards S: **A New Method for Ethical and Efficient Evidence Generation for Off-Label Medication Use in Oncology (A Case Study in Glioblastoma)**. *Front Pharmacol* 2019, **10**:681.

103. Patil VM, Bhelekar A, Menon N, Bhattacharjee A, Simha V, Abhinav R, Abhyankar A, Sridhar E, Mahajan A, Puranik AD *et al*: **Reverse swing-M, phase 1 study of repurposing mebendazole in recurrent high-grade glioma**. *Cancer Med* 2020, **9**(13):4676-4685.

104. Group WIW: **Guidelines for the treatment of cystic and alveolar echinococcosis in humans**. *WHO* 1996, **74**:231-242.

105. Münst G, Karlaganis G, Bircher J: **Plasma concentrations of mebendazole during treatment of echinococcosis: preliminary results**. *European journal of clinical pharmacology* 1980, **17**:375-378.

106. Zimmermann SC, Tichy T, Vavra J, Dash RP, Slusher CE, Gadiano AJ, Wu Y, Jancarik A, Tenora L, Monincova L *et al*: **N-Substituted Prodrugs of Mebendazole Provide Improved Aqueous Solubility and Oral Bioavailability in Mice and Dogs**. *J Med Chem* 2018, **61**(9):3918-3929.

107. Patil VM, Menon N, Chatterjee A, Tonse R, Choudhari A, Mahajan A, Puranik AD, Epari S, Jadhav M, Pathak S *et al*: **Mebendazole plus lomustine or temozolomide in patients with recurrent glioblastoma: A randomised open-label phase II trial**. *EClinicalMedicine* 2022, **49**:101449.

108. Michaelis M, Agha B, Rothweiler F, Loschmann N, Voges Y, Mittelbronn M, Starzetz T, Harter PN, Abhari BA, Fulda S *et al*: **Identification of flubendazole as potential anti-neuroblastoma compound in a large cell line screen**. *Sci Rep* 2015, **5**(1):8202.

109. Bai RY, Staedtke V, Wanjiku T, Rudek MA, Joshi A, Gallia GL, Riggins GJ: **Brain Penetration and Efficacy of Different Mebendazole Polymorphs in a Mouse Brain Tumor Model**. *Clin Cancer Res* 2015, **21**(15):3462-3470.

110. Ren LW, Li W, Zheng XJ, Liu JY, Yang YH, Li S, Zhang S, Fu WQ, Xiao B, Wang JH *et al*: **Benzimidazoles induce concurrent apoptosis and pyroptosis of human glioblastoma cells via arresting cell cycle**. *Acta Pharmacol Sin* 2022, **43**(1):194-208.

111. Dogra N, Kumar A, Mukhopadhyay TJSR: **Fenbendazole acts as a moderate microtubule destabilizing agent and causes cancer cell death by modulating multiple cellular pathways**. 2018, **8**(1):1-15.

112. (WHO) WHO: **WHO food additive series, no. 29. Toxicological evaluation of certain veterinary drug residues.** . In*.*; 1991.

113. Bruch K, Haas J: **Effectiveness of single doses of Fenbendazole Hoe 88I against Ascaris, hookworm and Trichuris in man**. *Ann Trop Med Parasitol* 1976, **70**(2):205-211.

114. Bhandari B, Singhi A: **Fenbendazole (Hoe 881) in enterobiasis**. *Trans R Soc Trop Med Hyg* 1980, **74**(5):691.

115. Vlachou I, Parsonidis P, Mamagkaki A, Bouris I, Papasotiriou I: **Teaching an old dog new tricks: The case of Fenbendazole**. *Cancer Treat Res Commun* 2022, **32**:100601.

116. Gao P, Dang CV, Watson J: **Unexpected Antitumorigenic Effect of Fenbendazole when Combined with Supplementary Vitamins**. *Journal of the American Association for Laboratory Animal Science* 2008, **47**(6):37-40.

117. Park D, Lee JH, Yoon SP: **Anti-cancer effects of fenbendazole on 5-fluorouracil-resistant colorectal cancer cells**. *Korean J Physiol Pharmacol* 2022, **26**(5):377-387.

118. Sultana T, Jan U, Lee H, Lee H, Lee JI: **Exceptional Repositioning of Dog Dewormer: Fenbendazole Fever**. *Curr Issues Mol Biol* 2022, **44**(10):4977-4986.

119. Bai RY, Staedtke V, Aprhys CM, Gallia GL, Riggins GJ: **Antiparasitic mebendazole shows survival benefit in 2 preclinical models of glioblastoma multiforme**. *Neuro Oncol* 2011, **13**(9):974-982.

120. Yamaguchi T, Shimizu J, Oya Y, Horio Y, Hida T: **Drug-Induced Liver Injury in a Patient with Nonsmall Cell Lung Cancer after the Self-Administration of Fenbendazole Based on Social Media Information**. *Case Rep Oncol* 2021, **14**(2):886-891.

121. Alavi SE, Ebrahimi Shahmabadi H: **Anthelmintics for drug repurposing: Opportunities and challenges**. *Saudi Pharm J* 2021, **29**(5):434-445.

122. Weng N, Zhang Z, Tan Y, Zhang X, Wei X, Zhu Q: **Repurposing antifungal drugs for cancer therapy**. *J Adv Res* 2023, **48**:259-273.

123. Pfab C, Schnobrich L, Eldnasoury S, Gessner A, El-Najjar N: **Repurposing of Antimicrobial Agents for Cancer Therapy: What Do We Know?** *Cancers (Basel)* 2021, **13**(13):3193.

124. Verbaanderd C, Maes H, Schaaf MB, Sukhatme VP, Pantziarka P, Sukhatme V, Agostinis P, Bouche G: **Repurposing Drugs in Oncology (ReDO)-chloroquine and hydroxychloroquine as anti-cancer agents**. *Ecancermedicalscience* 2017, **11**:781.

125. Wang X, Shen C, Liu Z, Peng F, Chen X, Yang G, Zhang D, Yin Z, Ma J, Zheng Z *et al*: **Nitazoxanide, an antiprotozoal drug, inhibits late-stage autophagy and promotes ING1-induced cell cycle arrest in glioblastoma**. *Cell Death Dis* 2018, **9**(10):1032.

126. Moertel CG, Fleming TR, Macdonald JS, Haller DG, Laurie JA, Goodman PJ, Ungerleider JS, Emerson WA, Tormey DC, Glick JH *et al*: **Levamisole and fluorouracil for adjuvant therapy of resected colon carcinoma**. *N Engl J Med* 1990, **322**(6):352-358.

127. Schultz CW, Nevler A: **Pyrvinium Pamoate: Past, Present, and Future as an Anti-Cancer Drug**. *Biomedicines* 2022, **10**(12):3249.

128. Laudisi F, Maronek M, Di Grazia A, Monteleone G, Stolfi C: **Repositioning of Anthelmintic Drugs for the Treatment of Cancers of the Digestive System**. *Int J Mol Sci* 2020, **21**(14):4957.

129. Pantziarka P, Sukhatme V, Bouche G, Meheus L, Sukhatme VP: **Repurposing Drugs in Oncology (ReDO)—itraconazole as an anti-cancer agent**. *Ecancermedicalscience* 2015, **9**.

130. Zhang L, Xu L, Zhang F, Vlashi E: **Doxycycline inhibits the cancer stem cell phenotype and epithelial-to-mesenchymal transition in breast cancer**. *Cell Cycle* 2017, **16**(8):737-745.

131. Mauthe M, Orhon I, Rocchi C, Zhou X, Luhr M, Hijlkema KJ, Coppes RP, Engedal N, Mari M, Reggiori F: **Chloroquine inhibits autophagic flux by decreasing autophagosome-lysosome fusion**. *Autophagy* 2018, **14**(8):1435-1455.

132. Chong CR, Xu J, Lu J, Bhat S, Sullivan DJ, Jr., Liu JO: **Inhibition of angiogenesis by the antifungal drug itraconazole**. *ACS Chem Biol* 2007, **2**(4):263-270.

133. De Francesco EM, Bonuccelli G, Maggiolini M, Sotgia F, Lisanti MP: **Vitamin C and Doxycycline: A synthetic lethal combination therapy targeting metabolic flexibility in cancer stem cells (CSCs)**. *Oncotarget* 2017, **8**(40):67269-67286.

134. Nau R, Sorgel F, Eiffert H: **Penetration of drugs through the blood-cerebrospinal fluid/blood-brain barrier for treatment of central nervous system infections**. *Clin Microbiol Rev* 2010, **23**(4):858-883.

135. Miceli MH: **Central Nervous System Infections Due to Aspergillus and Other Hyaline Molds**. *J Fungi (Basel)* 2019, **5**(3):79.

136. Halatsch ME, Kast RE, Karpel-Massler G, Mayer B, Zolk O, Schmitz B, Scheuerle A, Maier L, Bullinger L, Mayer-Steinacker R *et al*: **A phase Ib/IIa trial of 9 repurposed drugs combined with temozolomide for the treatment of recurrent glioblastoma: CUSP9v3**. *Neurooncol Adv* 2021, **3**(1):vdab075.

137. Nencioni A, Caffa I, Cortellino S, Longo VD: **Fasting and cancer: molecular mechanisms and clinical application**. *Nat Rev Cancer* 2018, **18**(11):707-719.

138. Takagi A, Kume S, Maegawa H, Uzu T: **Emerging role of mammalian autophagy in ketogenesis to overcome starvation**. *Autophagy* 2016, **12**(4):709-710.

139. Stepien K, Ostrowski RP, Matyja E: **Hyperbaric oxygen as an adjunctive therapy in treatment of malignancies, including brain tumours**. *Med Oncol* 2016, **33**(9):101.

140. Ortega MA, Fraile-Martinez O, Garcia-Montero C, Callejon-Pelaez E, Saez MA, Alvarez-Mon MA, Garcia-Honduvilla N, Monserrat J, Alvarez-Mon M, Bujan J *et al*: **A General Overview on the Hyperbaric Oxygen Therapy: Applications, Mechanisms and Translational Opportunities**. *Medicina (Kaunas)* 2021, **57**(9):864.

141. Kohshi K, Kinoshita Y, Imada H, Kunugita N, Abe H, Terashima H, Tokui N, Uemura SJBjoc: **Effects of radiotherapy after hyperbaric oxygenation on malignant gliomas**. 1999, **80**(1):236-241.

142. Poff AM, Ari C, Seyfried TN, D’Agostino DPJPo: **The ketogenic diet and hyperbaric oxygen therapy prolong survival in mice with systemic metastatic cancer**. 2013, **8**(6):e65522.

143. Alpuim Costa D, Sampaio-Alves M, Netto E, Fernandez G, Oliveira E, Teixeira A, Daniel PM, Bernardo GS, Amaro C: **Hyperbaric Oxygen Therapy as a Complementary Treatment in Glioblastoma-A Scoping Review**. *Front Neurol* 2022, **13**:886603.

144. Elsakka AMA, Bary MA, Abdelzaher E, Elnaggar M, Kalamian M, Mukherjee P, Seyfried TN: **Management of Glioblastoma Multiforme in a Patient Treated With Ketogenic Metabolic Therapy and Modified Standard of Care: A 24-Month Follow-Up**. *Front Nutr* 2018, **5**:20.

145. Suzuki Y, Tanaka K, Negishi D, Shimizu M, Yoshida Y, Hashimoto T, Yamazaki H: **Pharmacokinetic investigation of increased efficacy against malignant gliomas of carboplatin combined with hyperbaric oxygenation**. *Neurol Med Chir (Tokyo)* 2009, **49**(5):193-197; discussion 197.

146. Ishihara A: **Mild hyperbaric oxygen: mechanisms and effects**. *J Physiol Sci* 2019, **69**(4):573-580.

147. Gawdi R, Cooper JS: **Hyperbaric contraindications**. In: *StatPearls [Internet].* edn.: StatPearls Publishing; 2022.

148. Edwards ML: **Hyperbaric oxygen therapy. Part 1: history and principles**. *J Vet Emerg Crit Care (San Antonio)* 2010, **20**(3):284-288.

149. Costa DA, Ganilha JS, Barata PC, Guerreiro FG: **Seizure frequency in more than 180,000 treatment sessions with hyperbaric oxygen therapy - a single centre 20-year analysis**. *Diving Hyperb Med* 2019, **49**(3):167-174.

150. D'Agostino DP, Pilla R, Held HE, Landon CS, Puchowicz M, Brunengraber H, Ari C, Arnold P, Dean JB: **Therapeutic ketosis with ketone ester delays central nervous system oxygen toxicity seizures in rats**. *American journal of physiology* 2013, **304**(10):R829-836.

151. Ari C, Koutnik AP, DeBlasi J, Landon C, Rogers CQ, Vallas J, Bharwani S, Puchowicz M, Bederman I, Diamond DM *et al*: **Delaying latency to hyperbaric oxygen-induced CNS oxygen toxicity seizures by combinations of exogenous ketone supplements**. *Physiol Rep* 2019, **7**(1):e13961.

152. Mahmoudi K, Bouras A, Bozec D, Ivkov R, Hadjipanayis C: **Magnetic hyperthermia therapy for the treatment of glioblastoma: a review of the therapy's history, efficacy and application in humans**. *Int J Hyperthermia* 2018, **34**(8):1316-1328.

153. Clavo B, Santana-Rodríguez N, Llontop P, Gutiérrez D, Suárez G, López L, Rovira G, Martinez-Sanchez G, Gonzalez E, Jorge IJ: **Ozone therapy as adjuvant for cancer treatment: is further research warranted?** *Evidence-Based Complementary Alternative Medicine* 2018, **2018**.

154. Roberts JW, Powlovich L, Sheybani N, LeBlang S: **Focused ultrasound for the treatment of glioblastoma**. *J Neurooncol* 2022, **157**(2):237-247.

155. Skandalakis GP, Rivera DR, Rizea CD, Bouras A, Jesu Raj JG, Bozec D, Hadjipanayis CG: **Hyperthermia treatment advances for brain tumors**. *Int J Hyperthermia* 2020, **37**(2):3-19.

156. Fiorentini G, Sarti D, Milandri C, Dentico P, Mambrini A, Fiorentini C, Mattioli G, Casadei V, Guadagni S: **Modulated Electrohyperthermia in Integrative Cancer Treatment for Relapsed Malignant Glioblastoma and Astrocytoma: Retrospective Multicenter Controlled Study**. *Integr Cancer Ther* 2019, **18**:1534735418812691.

157. Megele R, Riemenschneider MJ, Dodoo-Schittko F, Feyrer M, Kleindienst A: **Intra-tumoral treatment with oxygen-ozone in glioblastoma: A systematic literature search and results of a case series**. *Oncol Lett* 2018, **16**(5):5813-5822.

158. Michelakis ED, Webster L, Mackey JR: **Dichloroacetate (DCA) as a potential metabolic-targeting therapy for cancer**. *Br J Cancer* 2008, **99**(7):989-994.

159. Tataranni T, Piccoli C: **Dichloroacetate (DCA) and Cancer: An Overview towards Clinical Applications**. *Oxid Med Cell Longev* 2019, **2019**:8201079.

160. Powell SF, Mazurczak M, Dib EG, Bleeker JS, Geeraerts LH, Tinguely M, Lohr MM, McGraw SC, Jensen AW, Ellison CA *et al*: **Phase II study of dichloroacetate, an inhibitor of pyruvate dehydrogenase, in combination with chemoradiotherapy for unresected, locally advanced head and neck squamous cell carcinoma**. *Invest New Drugs* 2022, **40**(3):622-633.

161. Dunbar EM, Coats BS, Shroads AL, Langaee T, Lew A, Forder JR, Shuster JJ, Wagner DA, Stacpoole PW: **Phase 1 trial of dichloroacetate (DCA) in adults with recurrent malignant brain tumors**. *Invest New Drugs* 2014, **32**(3):452-464.

162. Chu QS, Sangha R, Spratlin J, Vos LJ, Mackey JR, McEwan AJ, Venner P, Michelakis ED: **A phase I open-labeled, single-arm, dose-escalation, study of dichloroacetate (DCA) in patients with advanced solid tumors**. *Invest New Drugs* 2015, **33**(3):603-610.

163. Garon EB, Christofk HR, Hosmer W, Britten CD, Bahng A, Crabtree MJ, Hong CS, Kamranpour N, Pitts S, Kabbinavar F *et al*: **Dichloroacetate should be considered with platinum-based chemotherapy in hypoxic tumors rather than as a single agent in advanced non-small cell lung cancer**. *J Cancer Res Clin Oncol* 2014, **140**(3):443-452.

164. Abdelmalak M, Lew A, Ramezani R, Shroads AL, Coats BS, Langaee T, Shankar MN, Neiberger RE, Subramony SH, Stacpoole PW: **Long-term safety of dichloroacetate in congenital lactic acidosis**. *Mol Genet Metab* 2013, **109**(2):139-143.

165. Ishiguro T, Ishiguro R, Ishiguro M, Iwai S: **Co-treatment of dichloroacetate, omeprazole and tamoxifen exhibited synergistically antiproliferative effect on malignant tumors: in vivo experiments and a case report**. *Hepatogastroenterology* 2012, **59**(116):994-996.

166. Flavin D: **Medullary thyroid carcinoma relapse reversed with dichloroacetate: A case report**. *Oncol Lett* 2010, **1**(5):889-891.

167. Strum SB, Adalsteinsson Ö, Black RR, Segal D, Peress NL, Waldenfels J: **Case report: Sodium dichloroacetate (DCA) inhibition of the “Warburg Effect” in a human cancer patient: complete response in non-Hodgkin’s lymphoma after disease progression with rituximab-CHOP**. *Journal of bioenergetics biomembranes* 2013, **45**:307-315.

168. Khan A, Marier D, Marsden E, Andrews D, Eliaz I: **A novel form of dichloroacetate therapy for patients with advanced cancer: a report of 3 cases**. *Altern Ther Health Med* 2014, **20 Suppl 2**(Suppl 2):21-28.

169. Khan A, Andrews D, Shainhouse J, Blackburn AC: **Long-term stabilization of metastatic melanoma with sodium dichloroacetate**. *World J Clin Oncol* 2017, **8**(4):371-377.

170. Khan A, Andrews D, Blackburn AC: **Long-term stabilization of stage 4 colon cancer using sodium dichloroacetate therapy**. *World J Clin Cases* 2016, **4**(10):336-343.

171. Stacpoole PW: **The pharmacology of dichloroacetate**. *Metabolism* 1989, **38**(11):1124-1144.

172. Shroads AL, Guo X, Dixit V, Liu HP, James MO, Stacpoole PW: **Age-dependent kinetics and metabolism of dichloroacetate: possible relevance to toxicity**. *J Pharmacol Exp Ther* 2008, **324**(3):1163-1171.

173. Hoffer L, Levine M, Assouline S, Melnychuk D, Padayatty S, Rosadiuk K, Rousseau C, Robitaille L, Miller Jr W: **Phase I clinical trial of iv ascorbic acid in advanced malignancy**. *Annals of Oncology* 2008, **19**(11):1969-1974.

174. Creagan ET, Moertel CG, O'Fallon JR, Schutt AJ, O'Connell MJ, Rubin J, Frytak S: **Failure of high-dose vitamin C (ascorbic acid) therapy to benefit patients with advanced cancer. A controlled trial**. *N Engl J Med* 1979, **301**(13):687-690.

175. Ngo B, Van Riper JM, Cantley LC, Yun J: **Targeting cancer vulnerabilities with high-dose vitamin C**. *Nat Rev Cancer* 2019, **19**(5):271-282.

176. Padayatty SJ, Sun H, Wang Y, Riordan HD, Hewitt SM, Katz A, Wesley RA, Levine M: **Vitamin C pharmacokinetics: implications for oral and intravenous use**. *Ann Intern Med* 2004, **140**(7):533-537.

177. Luthringer M, Marziale J: **Intravenous Vitamin C (PDQ®): Integrative, alternative, and complementary therapies-Health Professional Information [NCI]**.

178. Integrative P: **High-Dose Vitamin C (PDQ®)**. In: *PDQ Cancer Information Summaries [Internet].* edn.: National Cancer Institute (US); 2013.

179. Heaney ML, Gardner JR, Karasavvas N, Golde DW, Scheinberg DA, Smith EA, O'Connor OA: **Vitamin C antagonizes the cytotoxic effects of antineoplastic drugs**. *Cancer Res* 2008, **68**(19):8031-8038.

180. Yanase F, Fujii T, Naorungroj T, Belletti A, Luethi N, Carr AC, Young PJ, Bellomo R: **Harm of IV High-Dose Vitamin C Therapy in Adult Patients: A Scoping Review**. *Critical care medicine* 2020, **48**(7):e620-e628.

181. Reang J, Sharma PC, Thakur VK, Majeed J: **Understanding the Therapeutic Potential of Ascorbic Acid in the Battle to Overcome Cancer**. *Biomolecules* 2021, **11**(8):1130.

182. Katzman BM, Kelley BR, Deobald GR, Myhre NK, Agger SA, Karon BS: **Unintended Consequence of High-Dose Vitamin C Therapy for an Oncology Patient: Evaluation of Ascorbic Acid Interference With Three Hospital-Use Glucose Meters**. *J Diabetes Sci Technol* 2021, **15**(4):897-900.

183. Heinemann L: **Interferences With CGM Systems: Practical Relevance?** *J Diabetes Sci Technol* 2022, **16**(2):271-274.

184. Wang W, Darling JL: **How could a drug used to treat alcoholism also be effective against glioblastoma?** *Expert review of anticancer therapy* 2013, **13**(3):239-241.

185. Ekinci E, Rohondia S, Khan R, Dou QP: **Repurposing Disulfiram as An Anti-Cancer Agent: Updated Review on Literature and Patents**. *Recent Pat Anticancer Drug Discov* 2019, **14**(2):113-132.

186. Kelley KC, Grossman KF, Brittain-Blankenship M, Thorne KM, Akerley WL, Terrazas MC, Kosak KM, Boucher KM, Buys SS, McGregor KA *et al*: **A Phase 1 dose-escalation study of disulfiram and copper gluconate in patients with advanced solid tumors involving the liver using S-glutathionylation as a biomarker**. *BMC Cancer* 2021, **21**(1):510.

187. **Trial of Disulfiram With Copper in Metastatic Breast Cancer (DISC)**. *The Institute of Molecular Translational Medicine, Czech Republic Clinicaltrials gov Identifier: NCT03323346*.

188. Hou J, Karin M, Sun B: **Targeting cancer-promoting inflammation—have anti-inflammatory therapies come of age?** *Nature reviews Clinical oncology* 2021, **18**(5):261-279.

189. Pantziarka P, Sukhatme V, Bouche G, Meheus L, Sukhatme VP: **Repurposing Drugs in Oncology (ReDO)-diclofenac as an anti-cancer agent**. *Ecancermedicalscience* 2016, **10**:610.

190. Dembo G, Park SB, Kharasch ED: **Central nervous system concentrations of cyclooxygenase-2 inhibitors in humans**. *Anesthesiology* 2005, **102**(2):409-415.

191. Rice AS, Lloyd J, Bullingham RE, O'Sullivan G: **Ketorolac penetration into the cerebrospinal fluid of humans**. *J Clin Anesth* 1993, **5**(6):459-462.

192. Netter P, Lapicque F, Bannwarth B, Tamisier JN, Thomas P, Royer RJ: **Diffusion of intramuscular ketoprofen into the cerebrospinal fluid**. *Eur J Clin Pharmacol* 1985, **29**(3):319-321.

193. Bannwarth B, Netter P, Lapicque F, Pere P, Thomas P, Gaucher A: **Plasma and cerebrospinal fluid concentrations of indomethacin in humans. Relationship to analgesic activity**. *Eur J Clin Pharmacol* 1990, **38**(4):343-346.

194. Ajmone-Cat MA, Bernardo A, Greco A, Minghetti L: **Non-Steroidal Anti-Inflammatory Drugs and Brain Inflammation: Effects on Microglial Functions**. *Pharmaceuticals (Basel)* 2010, **3**(6):1949-1965.

195. Stokum JA, Gerzanich V, Sheth KN, Kimberly WT, Simard JM: **Emerging Pharmacological Treatments for Cerebral Edema: Evidence from Clinical Studies**. *Annu Rev Pharmacol Toxicol* 2020, **60**:291-309.

196. Koh S, Dupuis N, Auvin S: **Ketogenic diet and Neuroinflammation**. *Epilepsy Res* 2020, **167**:106454.

197. Youm Y-H, Nguyen KY, Grant RW, Goldberg EL, Bodogai M, Kim D, D'agostino D, Planavsky N, Lupfer C, Kanneganti TD: **The ketone metabolite β-hydroxybutyrate blocks NLRP3 inflammasome–mediated inflammatory disease**. *Nat Med* 2015, **21**(3):263-269.

198. Polito R, La Torre ME, Moscatelli F, Cibelli G, Valenzano A, Panaro MA, Monda M, Messina A, Monda V, Pisanelli D *et al*: **The Ketogenic Diet and Neuroinflammation: The Action of Beta-Hydroxybutyrate in a Microglial Cell Line**. *Int J Mol Sci* 2023, **24**(4):3102.

199. Hossein Pourgholami M, Yan Cai Z, Lu Y, Wang L, Lawson Morris D: **Albendazole: a potent inhibitor of vascular endothelial growth factor and malignant ascites formation in OVCAR-3 tumor-bearing nude mice**. *Clinical Cancer Research* 2006, **12**(6):1928-1935.

200. Mercurio A, Adriani G, Catalano A, Carocci A, Rao L, Lentini G, Cavalluzzi MM, Franchini C, Vacca A, Corbo F: **A Mini-Review on Thalidomide: Chemistry, Mechanisms of Action, Therapeutic Potential and Anti-Angiogenic Properties in Multiple Myeloma**. *Curr Med Chem* 2017, **24**(25):2736-2744.

201. Chu M, Zhang C: **Inhibition of angiogenesis by leflunomide via targeting the soluble ephrin-A1/EphA2 system in bladder cancer**. *Sci Rep* 2018, **8**(1):1539.

202. Pore N, Gupta AK, Cerniglia GJ, Maity A: **HIV protease inhibitors decrease VEGF/HIF-1α expression and angiogenesis in glioblastoma cells**. *Neoplasia* 2006, **8**(11):889-895.

203. Gilbertson-Beadling S, Powers EA, Stamp-Cole M, Scott PS, Wallace TL, Copeland J, Petzold G, Mitchell M, Ledbetter S, Poorman R: **The tetracycline analogs minocycline and doxycycline inhibit angiogenesis in vitro by a non-metalloproteinase-dependent mechanism**. *Cancer Chemother Pharmacol* 1995, **36**(5):418-424.

204. Ripoll GV, Garona J, Pifano M, Farina HG, Gomez DE, Alonso DF: **Reduction of tumor angiogenesis induced by desmopressin in a breast cancer model**. *Breast Cancer Res Treat* 2013, **142**(1):9-18.

205. Nabors LB, Mikkelsen T, Hegi ME, Ye X, Batchelor T, Lesser G, Peereboom D, Rosenfeld MR, Olsen J, Brem S *et al*: **A safety run-in and randomized phase 2 study of cilengitide combined with chemoradiation for newly diagnosed glioblastoma (NABTT 0306)**. *Cancer* 2012, **118**(22):5601-5607.

206. Alalami H, Bannykh S, Fan X, Hu J: **Very long-term survival of an older glioblastoma patient after treatment with cilengitide: a case report**. *CNS Oncol* 2023, **12**(2):CNS96.

207. Mendez-Valdes G, Gomez-Hevia F, Lillo-Moya J, Gonzalez-Fernandez T, Abelli J, Cereceda-Cornejo A, Bragato MC, Saso L, Rodrigo R: **Endostatin and Cancer Therapy: A Novel Potential Alternative to Anti-VEGF Monoclonal Antibodies**. *Biomedicines* 2023, **11**(3):718.

208. Lugano R, Ramachandran M, Dimberg A: **Tumor angiogenesis: causes, consequences, challenges and opportunities**. *Cell Mol Life Sci* 2020, **77**(9):1745-1770.

209. Fu M, Zhou Z, Huang X, Chen Z, Zhang L, Zhang J, Hua W, Mao Y: **Use of Bevacizumab in recurrent glioblastoma: a scoping review and evidence map**. *BMC Cancer* 2023, **23**(1):544.

210. Gilbert MR, Dignam JJ, Armstrong TS, Wefel JS, Blumenthal DT, Vogelbaum MA, Colman H, Chakravarti A, Pugh S, Won M *et al*: **A randomized trial of bevacizumab for newly diagnosed glioblastoma**. *N Engl J Med* 2014, **370**(8):699-708.

211. Paez-Ribes M, Allen E, Hudock J, Takeda T, Okuyama H, Vinals F, Inoue M, Bergers G, Hanahan D, Casanovas O: **Antiangiogenic therapy elicits malignant progression of tumors to increased local invasion and distant metastasis**. *Cancer Cell* 2009, **15**(3):220-231.

212. de Groot JF, Fuller G, Kumar AJ, Piao Y, Eterovic K, Ji Y, Conrad CA: **Tumor invasion after treatment of glioblastoma with bevacizumab: radiographic and pathologic correlation in humans and mice**. *Neuro Oncol* 2010, **12**(3):233-242.

213. Thompson EM, Frenkel EP, Neuwelt EA: **The paradoxical effect of bevacizumab in the therapy of malignant gliomas**. *Neurology* 2011, **76**(1):87-93.

214. Maeyama M, Tanaka K, Nishihara M, Irino Y, Shinohara M, Nagashima H, Tanaka H, Nakamizo S, Hashiguchi M, Fujita YJSR: **Metabolic changes and anti-tumor effects of a ketogenic diet combined with anti-angiogenic therapy in a glioblastoma mouse model**. 2021, **11**(1):79.

215. Rieger J, Bähr O, Maurer GD, Hattingen E, Franz K, Brucker D, Walenta S, Kämmerer U, Coy JF, Weller MJIjoo: **ERGO: A pilot study of ketogenic diet in recurrent glioblastoma Erratum in/ijo/45/6/2605**. 2014, **44**(6):1843-1852.

216. Melhem JM, Tahir A, Calabrese E, Granovskaya I, Atenafu EG, Sahgal A, Lim-Fat MJ, Perry JR: **Dose-dependent efficacy of bevacizumab in recurrent glioblastoma**. *J Neurooncol* 2023, **161**(3):633-641.

217. Woolf EC, Curley KL, Liu Q, Turner GH, Charlton JA, Preul MC, Scheck ACJPO: **The ketogenic diet alters the hypoxic response and affects expression of proteins associated with angiogenesis, invasive potential and vascular permeability in a mouse glioma model**. 2015, **10**(6):e0130357.

218. Brewer GJ, Dick RD, Grover DK, LeClaire V, Tseng M, Wicha M, Pienta K, Redman BG, Jahan T, Sondak VK *et al*: **Treatment of metastatic cancer with tetrathiomolybdate, an anticopper, antiangiogenic agent: Phase I study**. *Clin Cancer Res* 2000, **6**(1):1-10.

219. Navratilova J, Hankeova T, Benes P, Smarda J: **Low-glucose conditions of tumor microenvironment enhance cytotoxicity of tetrathiomolybdate to neuroblastoma cells**. *Nutr Cancer* 2013, **65**(5):702-710.

220. Liu YL, Bager CL, Willumsen N, Ramchandani D, Kornhauser N, Ling L, Cobham M, Andreopoulou E, Cigler T, Moore A *et al*: **Tetrathiomolybdate (TM)-associated copper depletion influences collagen remodeling and immune response in the pre-metastatic niche of breast cancer**. *NPJ Breast Cancer* 2021, **7**(1):108.

221. Oien DB, Pathoulas CL, Ray U, Thirusangu P, Kalogera E, Shridhar V: **Repurposing quinacrine for treatment-refractory cancer**. In: *Seminars in Cancer Biology: 2021*: Elsevier; 2021: 21-30.

222. Kast RE: **Glioblastoma chemotherapy adjunct via potent serotonin receptor-7 inhibition using currently marketed high-affinity antipsychotic medicines**. *British journal of pharmacology* 2010, **161**(3):481-487.

223. Berges R, Denicolai E, Tchoghandjian A, Baeza-Kallee N, Honore S, Figarella-Branger D, Braguer D: **Proscillaridin A exerts anti-tumor effects through GSK3β activation and alteration of microtubule dynamics in glioblastoma**. *Cell Death Dis* 2018, **9**(10):1-14.

224. Jonsson KO, Andersson A, Jacobsson SO, Vandevoorde S, Lambert DM, Fowler CJ: **AM404 and VDM 11 non-specifically inhibit C6 glioma cell proliferation at concentrations used to block the cellular accumulation of the endocannabinoid anandamide**. *Archives of toxicology* 2003, **77**(4):201-207.

225. Cheng HW, Liang YH, Kuo YL, Chuu CP, Lin CY, Lee MH, Wu AT, Yeh CT, Chen EI, Whang-Peng J *et al*: **Identification of thioridazine, an antipsychotic drug, as an antiglioblastoma and anticancer stem cell agent using public gene expression data**. *Cell Death Dis* 2015, **6**(5):e1753.

226. Lee JK, Nam DH, Lee J: **Repurposing antipsychotics as glioblastoma therapeutics: Potentials and challenges**. *Oncol Lett* 2016, **11**(2):1281-1286.

227. Shafizadeh M, Farzaneh F, Kankam SB, Jangholi E, Shafizadeh Y, Khoshnevisan A: **Effects of postoperative intravenous Cyclosporine treatement on the survival and functional performance status of patients with glioblastoma: A randomized, triple-blinded, placebo-controlled clinical trial**. *World Neurosurgery* 2023.

228. Friesen C, Hormann I, Roscher M, Fichtner I, Alt A, Hilger R, Debatin KM, Miltner E: **Opioid receptor activation triggering downregulation of cAMP improves effectiveness of anti-cancer drugs in treatment of glioblastoma**. *Cell Cycle* 2014, **13**(10):1560-1570.

229. Shaw EG, Rosdhal R, D'Agostino RB, Jr., Lovato J, Naughton MJ, Robbins ME, Rapp SR: **Phase II study of donepezil in irradiated brain tumor patients: effect on cognitive function, mood, and quality of life**. *J Clin Oncol* 2006, **24**(9):1415-1420.

230. Huang C-x, Hu S, Chen B, Palma L, Schiffer D: **Growth inhibition of epidermal growth factor-stimulated human glioblastoma cells by nicardipine in vitro/Comments**. *Journal of neurosurgical sciences* 2001, **45**(3):151.

231. Zhang Y, Cruickshanks N, Yuan F, Wang B, Pahuski M, Wulfkuhle J, Gallagher I, Koeppel AF, Hatef S, Papanicolas C: **Targetable T-type Calcium Channels Drive GlioblastomaRole and Targeting of Calcium Channels in Glioblastoma**. *Cancer research* 2017, **77**(13):3479-3490.

232. Assad Kahn S, Costa SL, Gholamin S, Nitta RT, Dubois LG, Fève M, Zeniou M, Coelho PLC, El‐Habr E, Cadusseau J: **The anti‐hypertensive drug prazosin inhibits glioblastoma growth via the PKC δ‐dependent inhibition of the AKT pathway**. *EMBO molecular medicine* 2016, **8**(5):511-526.

233. El-Bacha RS, Netter P, Minn A: **Mechanisms of apomorphine cytoxicity towards rat glioma C6 cells: protection by bovine serum albumin and formation of apomorphine-protein conjugates**. *Neurosci Lett* 1999, **263**(1):25-28.

234. Durmaz R, Deliorman S, Uyar R, Isiksoy S, Erol K, Tel E: **The effects of anticancer drugs in combination with nimodipine and verapamil on cultured cells of glioblastoma multiforme**. *Clin Neurol Neurosurg* 1999, **101**(4):238-244.

235. Kast RE, Belda-Iniesta C: **Suppressing glioblastoma stem cell function by aldehyde dehydrogenase inhibition with chloramphenicol or disulfiram as a new treatment adjunct: an hypothesis**. *Curr Stem Cell Res Ther* 2009, **4**(4):314-317.

236. Damiani E, Yuecel R, Wallace HM: **Repurposing of idebenone as a potential anti-cancer agent**. *Biochem J* 2019, **476**(2):245-259.

237. Fu L, Jin W, Zhang J, Zhu L, Lu J, Zhen Y, Zhang L, Ouyang L, Liu B, Yu H: **Repurposing non-oncology small-molecule drugs to improve cancer therapy: Current situation and future directions**. *Acta Pharm Sin B* 2022, **12**(2):532-557.

238. PDQ Integrative A, Complementary Therapies Editorial Board: **Mistletoe Extracts (PDQ®): Health Professional Version**. *PDQ Cancer Information Summaries* 2002.

239. Grabacka MM, Wilk A, Antonczyk A, Banks P, Walczyk-Tytko E, Dean M, Pierzchalska M, Reiss KJFie: **Fenofibrate induces ketone body production in melanoma and glioblastoma cells**. 2016, **7**:5.

240. Natale G, Fini E, Calabro PF, Carli M, Scarselli M, Bocci G: **Valproate and lithium: Old drugs for new pharmacological approaches in brain tumors?** *Cancer Lett* 2023, **560**:216125.

241. Serafin MB, Bottega A, da Rosa TF, Machado CS, Foletto VS, Coelho SS, da Mota AD, Horner R: **Drug Repositioning in Oncology**. *Am J Ther* 2021, **28**(1):e111-e117.

242. Halma MTJ, Tuszynski JA, Marik PE: **Cancer Metabolism as a Therapeutic Target and Review of Interventions**. *Nutrients* 2023, **15**(19).

243. Galicia-Garcia U, Jebari S, Larrea-Sebal A, Uribe KB, Siddiqi H, Ostolaza H, Benito-Vicente A, Martin C: **Statin Treatment-Induced Development of Type 2 Diabetes: From Clinical Evidence to Mechanistic Insights**. *Int J Mol Sci* 2020, **21**(13):4725.

244. Tricarico PM, Crovella S, Celsi F: **Mevalonate Pathway Blockade, Mitochondrial Dysfunction and Autophagy: A Possible Link**. *Int J Mol Sci* 2015, **16**(7):16067-16084.

245. Afshari AR, Mollazadeh H, Henney NC, Jamialahmad T, Sahebkar A: **Effects of statins on brain tumors: a review**. In: *Seminars in Cancer Biology: 2021*: Elsevier; 2021: 116-133.
