# Supplementary material for: Clinical research framework proposal for ketogenic metabolic therapy in glioblastoma
Source: BMC Med. 2024 Dec 5;22:578. doi: 10.1186/s12916-024-03775-4 (PMC11622503; doi:10.1186/s12916-024-03775-4)
Supplement: Supplementary file 3 — Supplementary Material 3. [file 12916_2024_3775_MOESM3_ESM.docx]

**Supplementary Table 1.** Clinical studies evaluating KDs for high-grade glioma. Patient-specific data should be presented in a manner that allows for statistical evaluation of individualized responses over time, avoiding group averages. All units have been transformed to millimolar (mM) for clarity.

| **Intervention and study design** | **Glucose data (average ± SD, or min/max)** | **βHB data (average ± SD, or min/max)** | **Reported or estimated GKI (average ± SD, if available)** | **Comments and study outcomes** | **References** |
| --- | --- | --- | --- | --- | --- |
| **Studies with raw data, allowing for cumulative GKI stratification** | | | | | |
| KD-R. Case report, grade IV IDH1-R132H mutant glioma, 80 month follow-up. | 4.66 ± 0.64 mM (calculated from raw data for 56 months). | 3.62 ± 1.47 mM (calculated from raw data for 56 months). | 1.68 ± 1.25 (calculated from raw data for 56 months). | Daily glucose and ketone values provided as raw data, allowing for GKI calculation and statistical analysis. Cumulative GKI largely in therapeutic zone. Low absolute glucose levels and low glycemic variability. Tumor response determines study duration: lowest GKI should be maintained as long as there is evidence of persistent disease. Diet flexibility should not compromise GKI targets. Methodology from this case report could be adapted for larger clinical trials. | [1] |
| **Studies that would allow for individual GKI stratification if raw data was provided** | | | | | |
| 3:1 KD. Clinical study with 9 evaluable GBM patients, KD for 6 weeks. | Reported as average ± SD for each patient at the end of the study and daily aggregate averages for all patients (numerical values not provided). | Reported as average ± SD for each patient at the end of the study and daily aggregate averages for all patients (numerical values not provided). | Aggregate GKI at the end of the study: 2.76. Cannot be calculated for daily measurements in individual patients. | Absolute glucose levels out of therapeutic zone, although some patients experienced lower glucose variability. GKI < 2 likely achieved by several patients, but data reporting prevents from correlating outcomes with time spent in specific ranges. Long-term survivors were younger and IDH1-R132H positive (80, 62, and 64 months PFS since diagnosis). Despite being isocaloric in design, reported weight loss indicates that this was effectively a KD-R study. | [2] |
| KD (unrestricted). Prospective case series, 10 GBM patients, 161 ± 74 days follow-up. | Measured 3 times a week. Reported average: 5.66 ± 0.46 mM. | Measured 3 times a week. Reported average: 1.11 ± 0.52 mM. | 5.10 (calculated from reported averages). | GKI mostly outside therapeutic zone. Absolute glucose levels too high. Raw data for each patient would be necessary to evaluate sources of glycemic variability. Given the combined fasting intervention, it is not possible to assign weight loss causality, although GKI values suggests that the KD was likely not restricted. | [3] |
| 5-7 day water-only fasting every 1-2 months in the same cohort as above. 10 GBM patients, 34 ± 18 days. | Measured daily during fasting. Reported average: 4.50 ± 0.68 mM. | Measured daily during fasting. Reported average: 3.52 ± 0.99 mM. | 1. 28 (calculated from reported averages). | GKI in therapeutic range during fasting. KD outside fasting intervals would require adjustments to reach GKI targets. Reported mOS 13 months (range 5.2-33.0). Short periods of fasting do not appear to compensate for high cumulative GKI. | [3] |
| Isocaloric 3:1 KD (<30g daily carbohydrates). Single-arm prospective study with 6 eligible high-grade glioma patients (5 GBM), 1 month follow-up. | Daily measurements not reported as numerical values. Baseline glycemia: 5.2 ± 0.5 mM.  1 month follow-up: 4.9 ± 0.6 mM. | Daily measurements not reported as numerical values. High inter-patient variability. | Cannot be calculated without numerical values. | Heterogeneous responses and diet adherence. Absolute glucose levels too high. Changes in average fat mass indicate that the intervention was effectively KD-R for some patients. Survival was not an endpoint. Further evaluation could be performed if individual raw data for each patient was provided. | [4] |
| KD-R (meal replacement). Prospective pilot study, 8 GBM patients, 5 of which completed 6 months of KD. | Blood glucose reported as average ± min/max for each patient. | Blood ketone levels reported as average ± min/max for each patient. | Average GKI could be calculated for each patient, but not cumulative daily GKI. | Calorie restriction was only sustained during the first month and then reversed due to weight loss concerns. High glycemic variability, probably related to corticosteroids. Reported mean survival: 21.8 months (range 11-29.2) for newly diagnosed GBM patients. Correlations between outcomes and GKI could be performed if daily raw data was provided. | [5] |
| 3:1 KD. Retrospective pilot study, 8 evaluable high-grade glioma patients, 120 days. | Blood glucose measured twice daily. | Blood ketones measured twice daily. | GKI values provided at day 30 and end of study for each patient; cumulative GKI could be calculated if numerical values were provided. | This study provides an example of appropriate GKI reporting; however, raw data was not included. Average ketone levels were higher in six patients who were alive at study endpoint compared to two deceased patients. Despite relatively good glycemic control, most patients found it difficult to sustain GKI < 2.0 and blood glucose levels < 5 mM towards the end of the study. Reduction in BMI for most patients suggests that the diet was effectively KD-R. | [6] |
| 2:1 and 1:1 KD. Case series of 37 evaluable patients with stage 4 malignancies, including brain cancer, 3 month follow-up. | Individual values presented for each patient at first week and each monthly visit. Average glucose levels at the end of the study: 5.0 ± 0.7 mM. Daily measurements not available. | Individual values presented for each patient at first week and each monthly visit. Average βHB levels at the end of the study: 1.7 ± 1.2 mM. Daily measurements not available. | GKI reported for each patient’s visit. Only a small portion of patients maintained GKI < 1, but most could sustain GKI < 3. | This study provides an example of appropriate GKI reporting; however, daily measurements would be preferable, as there was clear GKI variability, indicating that once per month evaluation cannot capture cumulative effects. Glucose ≤ 5 mM was associated with improved prognosis, together with CRP ≤ 0.5 mg/dl and albumin ≥ 4.0 g/dl (defined as the KD-ABC score). Five-year follow-up with long-term survival rates presented in [7]. Changes in fat mass indicates that the diet was effectively eucaloric. Initial BMI shows that the KD can be safely administered in cancer patients with normal weight. The characteristics of this population (lower basal caloric intake) enabled nutritional ketosis with a 2:1 ratio due to absolute carbohydrate intake < 30 g/day. In populations with higher caloric requirements, carbohydrate intake should be kept < 20-50 g/day, regardless of ketogenic ratios. | [8] |
| Unrestricted, liquid KD (90% fat) during chemoradiotherapy. Prospective feasibility study, 9 evaluable GBM patients, 8 week follow-up. | 4.7 ± 0.17 mM (calculated from reported averages) | 4.3 ± 1.20  mM (calculated from reported averages) | Not reported. Cannot be calculated for individual patients. | Average estimated GKI close to therapeutic zone (owing to diet uniformity by consuming a high ketogenic ratio medical food). Raw data of daily measurements for each patient not provided. Glycemic variability and adherence to daily tracking unknown. | [9] |
| Unrestricted, solid food KD with added MCTs. Prospective feasibility study, 9 evaluable GBM patients, 6 week follow-up. | 5.2 ± 0.70 mM (calculated from reported data) | 2.9 ± 1.17 mM (calculated from reported data) | Not reported. Cannot be calculated for individual patients. | Average estimated GKI outside therapeutic zone for most patients due to high diet flexibility. Reported mOS for evaluable patients: 12.8 months (range 9.8–19.0). Absolute glucose levels too high, despite relatively stable nutritional ketosis. Changes in weight not described, but caloric restriction was not intended. | [9] |
| KD-R. Case reports of 2 GBM patients, 12 week follow-up. | Blood glucose measured daily. Numerical values not provided, reported as generally < 5 mM. | Blood ketones measured daily. Numerical values not provided, reported as 2 to 4 mM. | Could be calculated if raw data was provided. | GKI could be estimated as close to 2, hindered by high glycemic variability, despite relatively stable nutritional ketosis. Personalized approaches to KD-R implementation with daily tracking could be implemented in larger clinical trials. | [10] |
| KD and KD-R. Retrospective study with 6 GBM patients following KD during SOC, 3 to 12 months. | Average non-fasting serum glucose on KD (during radiotherapy): 4.66 mM (min/max 4.2-5.1 mM). Baseline: 7.9 mM (range 4.5-10.0 mM). | Patients instructed to measure blood ketones twice a week, but values not reported. | Cannot be calculated without βHB data. | Emphasis given to blood glucose levels, managed even during steroid therapy. As inferred from weight loss, the diet was effectively a KD-R for some patients. Relatively low glycemic variability. Four patients were alive at median follow-up of 14 months. No correlations with outcomes can be drawn without sharing raw data for individual patients. | [11] |
| **Studies that do not allow for individual GKI stratification** | | | | | |
| KMT (in the form of non-standardized KDs). Retrospective case series of 16 high-grade glioma patients, 3 to 48 months. | Not reported. | Blood βHB not consistently measured. For available data, reported as 1.6 ± 1.1 mM. | Cannot be calculated. | Unfortunately, ketone levels cannot be correlated with therapeutic efficacy without continuous recording glycemic control. Reported PFS for patients following intervention: 20.0 ± 14.4 months. | [12] |
| 5 days of unrestricted Modified Atkins Diet (MAD) with 2 days of intermittent fasting at < 20% caloric requirements. 25 participants, of which 12 with WHO grade 4 astrocytoma. | Reported at baseline and end of study. No reduction in fasting blood glucose. | Urinary ketones measured twice weekly. | Cannot be calculated. | No changes in lactate were detected in tumor tissue via MRS, but there was normalization of glutamine and choline metabolism. Correlations with survival not evaluated. | [13] |
| MAD with MCT supplementation and metformin. 8 week phase I clinical trial combining KD with MCT supplementation and metformin during radiotherapy in high-grade gliomas, 8 evaluable patients. | Mean baseline glucose reported as 5.5 mM. During treatment: 5.3 mM. SD not reported. | Blood βHB during first 2 weeks, then urinary sticks every 2 days. Mean ranging from 0.17 to 1.3 mM, with maximal up to 2.5 mM. | Cannot be calculated. | Despite MCT supplementation, relatively low nutritional ketosis suggest that the diet was not sufficiently restricted in carbohydrates (ratio reported as 1:1 to 3:1). This is especially apparent from insulin, C-peptide and IGF1. Reported mOS: 21 months for newly diagnosed GBM and 8 months for recurrent disease. The authors recognize that flexibility at the request of local IRB may have compromised efficacy. Absolute glucose values outside therapeutic zone. Mean ketone levels would suggest that GKI was generally outside therapeutic zone. | [14] |
| KD-R and 3-day fasting over 9 days. 20 patients with recurrent GBM (ERGO 2 trial). | Glucose levels measured at the end of the 3-day fast: reduction of 0.6 ± 0.8 mM from baseline. | βHB levels on the last day of the 3-day fast: 1.9 ± 1.5 mM | Not reported. | Increased PFS associated with glucose levels < 4.6 mM. Unfortunately, this 9-day intervention was administered only once as a radiosensitizer. Effective KMT likely requires sustained treatment. 3-day water-only fasting would be expected to induce higher average increases in βHB, e.g., 3.15 + 0.67 mM as shown in [15], and steeper reductions in blood glucose [16], but this may have been related to concurrent SOC. | [17] |
| Modified Ketogenic Diet (MKD) and MCT-oil based KD. Prospective controlled feasibility study in newly diagnosed GBM with 4 evaluable patients, 12 months. | It is stated that blood glucose was monitored once weekly, but values not reported. | Blood ketones measured once weekly, but not reported. | Cannot be calculated without glucose and βHB data. | In one patient receiving MKD, significant weight loss was observed during the first 3 months (KD-R). In patients receiving MCT-oil KD, weight stability indicates an unrestricted KD. Unfortunately, most patients withdrew during SOC administration. Reported mOS 15.48 months. Correlations with therapeutic efficacy cannot be performed. | [18] |
| MAD. Retrospective feasibility study with 29 evaluable patients, 19 with grade IV astrocytoma, 6 weeks. | Measured only during scheduled blood draws. Data not reported. | Range of serum βHB reported for each patient (not as numerical values). | Cannot be calculated without glucose values. | The diet was KD-R for some patients due to weight loss. Feasibility defined as 2 or more readings of βHB ≥ 0.5 mM during the intervention. 2-year survival for evaluable patients reported as 26.7%. Correlations with GKI cannot be performed. | [19] |
| KMT (KD-R and unrestricted KD). GBM case report, 24 months follow-up. | During follow-up, glucose between ≈ 3 to 4 mM. | Only urinary ketones evaluated. | After ketogenic adaptation, GKI consistently below 2.0 (as estimated based on urinary ketones). | GKI seemingly in therapeutic zone even during refeeding for weight maintenance. No steroids (dexamethasone), phenytoin, or sugar-based osmatic diuretics (mannitol) were administered, improving GKI. | [20] |
| MKD. Prospective controlled feasibility NHS trial with 4 evaluable GBM patients, 12 weeks. | Baseline glucose not reported. No ongoing evaluation of glucose levels. | Only urinary ketones, estimated as at least 4 mM for 3 out of 4 patients. | Cannot be calculated without glucose and βHB data. | Stable weight indicates that the diet was effectively unrestricted. Not enough data for an objective assessment of survival and biological effects. | [21] |
| MAD. Prospective 3 month study of intranasal perillyl alcohol with 17 evaluable GBM patients, 9 followed KD. | Glucose not measured daily. Baseline mean: 4.9 ± 0.6 mM, at 3 months: 4.6 ±0.7 mM. | Urinary ketones measured 3 times a week, but not reported. | Cannot be calculated without ketone levels. | Variable evolution of weight indicates that the diet was hypercaloric for some patients, or there was poor adherence. Fasting glucose levels did not change with the intervention. Correlations with efficacy cannot be made without daily tracking. | [22] |
| MAD. 11 evaluable patients with advanced malignancies, 2 high-grade gliomas, 16 weeks. | After diet initiation, 4.9 ± 0.8 mM.  Last visit at 16 weeks, 5.4 ± 0.66 mM | After diet initiation, 1.10 ± 0.98 mM.  Last visit at 16 weeks, 0.87 ± 0.83 mM. | Reported for some patients, but not for high-grade glioma patients. | GKI out of therapeutic range or not reported, absolute glucose levels out of therapeutic range. Inconsistent diet adherence. Correlations with efficacy cannot be made for brain cancer patients. | [23] |
| MAD. 8 patients with high-grade brain tumors, 2 to 24 months. | Not measured. | Urine ketones measured biweekly for 1 month, then once a week. | Cannot be calculated. | Relative weight stability at 13 months follow-up indicates that the diet was eventually unrestricted. Survival rates comparable to expected outcomes. No further assessment of compliance or efficacy can be performed. | [24] |
| KD (unrestricted). Prospective controlled pilot study of KD for recurrent GBM (ERGO trial), 17 patients, 3 to 16 weeks. | Prior to KD, glucose reported as 5.5 ± 1.2 mM; during KD, 5.1 ± 0.5 mM. | Only urinary ketones measured. | Not reported. | As suggested by weight stability, the diet was effectively unrestricted for most patients. No change in absolute glucose levels but improved glycemic variability. Reported mOS after start of diet: 32 weeks (range 6-86+). Stable ketosis associated with improved PFS. | [25] |
| KMT (KD-R and fasting). GBM case report. | During KD-R, blood glucose ≈ 3.5 mM, but not measured daily. | Only urinary ketones measured, estimated as ≈ 2.5 mM. | During KD-R, GKI below 2.0 (as estimated based on urinary ketones). | Significant calorie restriction. Low absolute glucose levels. Stable disease after 2 months of treatment. Recurrence after 10 weeks of discontinuing KD-R. | [26] |
| MCT-oil based KD. 2 case reports, high-grade astrocytoma. | Numerical values not provided. Glucose levels reported as ≈ 3 to 6 mM. | Numerical values not provided. During dietary compliance, βHB reported as > 2 mM. | Not reported. | Glucose and ketones assessed once per week. Daily measurements would be more appropriate to capture cumulative time in therapeutic range. | [27] |

**Abbreviations:** βHB, Beta-Hydroxybutyrate; GBM, Glioblastoma Multiforme; GKI, Glucose Ketone Index; KD, Ketogenic Diet; KD-R, Restricted Ketogenic Diet; MAD, Modified Atkins Diet; MCT, Medium-Chain Triglyceride; MKD, Modified Ketogenic Diet; mOS, Median Overall Survival; PFS, Progression-Free Survival; SD, Standard Deviation.

**References**:

1. Seyfried TN, Shivane AG, Kalamian M, Maroon JC, Mukherjee P, Zuccoli G: **Ketogenic Metabolic Therapy, Without Chemo or Radiation, for the Long-Term Management of IDH1-Mutant Glioblastoma: An 80-Month Follow-Up Case Report**. *Front Nutr* 2021, **8**:682243.

2. Schwartz KA, Noel M, Nikolai M, Olson LK, Hord NG, Zakem M, Clark J, Elnabtity M, Figueroa B, Chang HTJFiN: **Long Term Survivals in Aggressive Primary Brain Malignancies Treated With an Adjuvant Ketogenic Diet**. 2022, **9**.

3. Phillips MCL, Leyden J, McManus EJ, Lowyim DG, Ziad F, Moon BG, Haji Mohd Yasin NAB, Tan A, Thotathil Z, Jameson MB: **Feasibility and Safety of a Combined Metabolic Strategy in Glioblastoma Multiforme: A Prospective Case Series**. *Journal of oncology* 2022, **2022**:4496734.

4. Foppiani A, De Amicis R, Lessa C, Leone A, Ravella S, Ciusani E, Silvani A, Zuccoli G, Battezzati A, Lamperti EJN *et al*: **Isocaloric ketogenic diet in adults with high-grade gliomas: a prospective metabolic study**. 2021, **73**(6):1004-1014.

5. Klein P, Tyrlikova I, Zuccoli G, Tyrlik A, Maroon JC: **Treatment of glioblastoma multiforme with "classic" 4:1 ketogenic diet total meal replacement**. *Cancer Metab* 2020, **8**(1):24.

6. Panhans CM, Gresham G, Amaral LJ, Hu J: **Exploring the Feasibility and Effects of a Ketogenic Diet in Patients With CNS Malignancies: A Retrospective Case Series**. *Front Neurosci* 2020, **14**:390.

7. Egashira R, Matsunaga M, Miyake A, Hotta S, Nagai N, Yamaguchi C, Takeuchi M, Moriguchi M, Tonari S, Nakano M *et al*: **Long-Term Effects of a Ketogenic Diet for Cancer**. *Nutrients* 2023, **15**(10):2334.

8. Hagihara K, Kajimoto K, Osaga S, Nagai N, Shimosegawa E, Nakata H, Saito H, Nakano M, Takeuchi M, Kanki H *et al*: **Promising Effect of a New Ketogenic Diet Regimen in Patients with Advanced Cancer**. *Nutrients* 2020, **12**(5).

9. van der Louw EJ, Olieman JF, van den Bemt PM, Bromberg JE, Oomen-de Hoop E, Neuteboom RF, Catsman-Berrevoets CE, Vincent AJ: **Ketogenic diet treatment as adjuvant to standard treatment of glioblastoma multiforme: a feasibility and safety study**. *Therapeutic advances in medical oncology* 2019, **11**:1758835919853958.

10. Schwartz K, Chang HT, Nikolai M, Pernicone J, Rhee S, Olson K, Kurniali PC, Hord NG, Noel M: **Treatment of glioma patients with ketogenic diets: report of two cases treated with an IRB-approved energy-restricted ketogenic diet protocol and review of the literature**. *Cancer Metab* 2015, **3**:3.

11. Champ CE, Palmer JD, Volek JS, Werner-Wasik M, Andrews DW, Evans JJ, Glass J, Kim L, Shi W: **Targeting metabolism with a ketogenic diet during the treatment of glioblastoma multiforme**. *J Neurooncol* 2014, **117**(1):125-131.

12. Smith KA, Hendricks BK, DiDomenico JD, Conway BN, Smith TL, Azadi A, Fonkem E: **Ketogenic Metabolic Therapy for Glioma**. *Cureus* 2022, **14**(6):e26457.

13. Schreck KC, Hsu FC, Berrington A, Henry-Barron B, Vizthum D, Blair L, Kossoff EH, Easter L, Whitlow CT, Barker PB *et al*: **Feasibility and Biological Activity of a Ketogenic/Intermittent-Fasting Diet in Patients With Glioma**. *Neurology* 2021, **97**(9):e953-e963.

14. Porper K, Shpatz Y, Plotkin L, Pechthold RG, Talianski A, Champ CE, Furman O, Shimoni-Sebag A, Symon Z, Amit U *et al*: **A Phase I clinical trial of dose-escalated metabolic therapy combined with concomitant radiation therapy in high-grade glioma**. *J Neurooncol* 2021, **153**(3):487-496.

15. Pan JW, Rothman TL, Behar KL, Stein DT, Hetherington HP: **Human brain beta-hydroxybutyrate and lactate increase in fasting-induced ketosis**. *J Cereb Blood Flow Metab* 2000, **20**(10):1502-1507.

16. Gjedsted J, Gormsen LC, Nielsen S, Schmitz O, Djurhuus CB, Keiding S, Orskov H, Tonnesen E, Moller N: **Effects of a 3-day fast on regional lipid and glucose metabolism in human skeletal muscle and adipose tissue**. *Acta Physiol (Oxf)* 2007, **191**(3):205-216.

17. Voss M, Wagner M, von Mettenheim N, Harter PN, Wenger KJ, Franz K, Bojunga J, Vetter M, Gerlach R, Glatzel M *et al*: **ERGO2: A Prospective, Randomized Trial of Calorie-Restricted Ketogenic Diet and Fasting in Addition to Reirradiation for Malignant Glioma**. *Int J Radiat Oncol Biol Phys* 2020, **108**(4):987-995.

18. Martin-McGill KJ, Marson AG, Tudur Smith C, Young B, Mills SJ, Cherry MG, Jenkinson MD: **Ketogenic diets as an adjuvant therapy for glioblastoma (KEATING): a randomized, mixed methods, feasibility study**. *J Neurooncol* 2020, **147**(1):213-227.

19. Woodhouse C, Ward T, Gaskill-Shipley M, Chaudhary R: **Feasibility of a modified Atkins diet in glioma patients during radiation and its effect on radiation sensitization**. *Curr Oncol* 2019, **26**(4):e433-e438.

20. Elsakka AMA, Bary MA, Abdelzaher E, Elnaggar M, Kalamian M, Mukherjee P, Seyfried TN: **Management of Glioblastoma Multiforme in a Patient Treated With Ketogenic Metabolic Therapy and Modified Standard of Care: A 24-Month Follow-Up**. *Front Nutr* 2018, **5**:20.

21. Martin-McGill KJ, Marson AG, Tudur Smith C, Jenkinson MD: **The Modified Ketogenic Diet in Adults with Glioblastoma: An Evaluation of Feasibility and Deliverability within the National Health Service**. *Nutr Cancer* 2018, **70**(4):643-649.

22. Santos JG, Da Cruz WMS, Schönthal AH, Salazar M, Fontes CAP, Quirico‑Santos T, Da Fonseca CO: **Efficacy of a ketogenic diet with concomitant intranasal perillyl alcohol as a novel strategy for the therapy of recurrent glioblastoma**. *Oncol Lett* 2018, **15**(1):1263-1270.

23. Tan-Shalaby JL, Carrick J, Edinger K, Genovese D, Liman AD, Passero VA, Shah RB: **Modified Atkins diet in advanced malignancies-final results of a safety and feasibility trial within the Veterans Affairs Pittsburgh Healthcare System**. *Nutrition metabolism* 2016, **13**(1):1-12.

24. Strowd RE, Cervenka MC, Henry BJ, Kossoff EH, Hartman AL, Blakeley JO: **Glycemic modulation in neuro-oncology: experience and future directions using a modified Atkins diet for high-grade brain tumors**. *Neurooncol Pract* 2015, **2**(3):127-136.

25. Rieger J, Bähr O, Maurer GD, Hattingen E, Franz K, Brucker D, Walenta S, Kämmerer U, Coy JF, Weller M: **ERGO: A pilot study of ketogenic diet in recurrent glioblastoma Erratum in/ijo/45/6/2605**. *International journal of oncology* 2014, **44**(6):1843-1852.

26. Zuccoli G, Marcello N, Pisanello A, Servadei F, Vaccaro S, Mukherjee P, Seyfried TN: **Metabolic management of glioblastoma multiforme using standard therapy together with a restricted ketogenic diet: Case Report**. *Nutr Metab (Lond)* 2010, **7**(1):33.

27. Nebeling LC, Miraldi F, Shurin SB, Lerner E: **Effects of a ketogenic diet on tumor metabolism and nutritional status in pediatric oncology patients: two case reports**. *J Am Coll Nutr* 1995, **14**(2):202-208.
