# Supplementary material for: Clinical research framework proposal for ketogenic metabolic therapy in glioblastoma
Source: BMC Med. 2024 Dec 5;22:578. doi: 10.1186/s12916-024-03775-4 (PMC11622503; doi:10.1186/s12916-024-03775-4)
Supplement: Supplementary file 4 — Supplementary Material 4. [file 12916_2024_3775_MOESM4_ESM.docx]

**

Supplementary Figure 1.** Proposed flow diagram for clinical studies testing standalone dietary and pharmacological KMT in GBM, with elective, conditional radiotherapy and/or chemotherapy delay.
