# Supplementary material for: Clinical research framework proposal for ketogenic metabolic therapy in glioblastoma
Source: BMC Med. 2024 Dec 5;22:578. doi: 10.1186/s12916-024-03775-4 (PMC11622503; doi:10.1186/s12916-024-03775-4)
Supplement: Supplementary file 5 — Supplementary Material 5. [file 12916_2024_3775_MOESM5_ESM.docx]

**Supplementary Table 2.** Drug repurposing and research-phase compounds for the simultaneous targeting of glycolysis and glutaminolysis.

| **Drug or intervention** | **Core pathways** | **Safety and biotransformation** | **Commentary and suggested use** | **References** |
| --- | --- | --- | --- | --- |
| **GLYCOLYSIS TARGETING** | | | | |
| Metformin. | Reduced liver gluconeogenesis, AMPK activation, mTOR inhibition. | Metformin is a widely used anti-diabetic medication with an established safety profile. Common side effects include mild gastrointestinal disturbances; a rare adverse effect is vitamin B12 depletion and metformin-associated lactic acidosis [1]. No hepatic metabolism. Excreted unchanged in urine. At clinical doses, metformin is not an OXPHOS inhibitor, rather primarily a liver gluconeogenesis inhibitor. | Adjust to reach ideal GKI. Suggested dosing in type 2 diabetes: starting at lowest dose, 500 mg q.d., up to maximum 1000 mg b.i.d. (immediate release formulation) or 2000 mg q.d. (extended-release formulation). In cancer therapy, metformin is usually started at 850 mg/day and increased up to 2550 mg/day. Clinical trials with KD in GBM (NCT04691960, NCT05183204, NCT04945148). Measuring GKI is recommended to evaluate outcomes. | [2-4] |
| SGLT2 inhibitors (e.g., empagliflozin, dapagliflozin, canagliflozin). | Inhibition of renal glucose reabsorption (improved GKI), reduced insulin signaling. | Adverse effects include increased risk of urinary tract infections and euglycemic diabetic ketoacidosis, particularly in patients with insulin deficiency or acute illness [5, 6]. Canagliflozin metabolized by UGT1A9 and UGT2B4. Dapagliflozin primarily metabolized via UGT1A9. | Adjust dosing to reach therapeutic GKI. No ongoing trials for adult GBM but increasing research interest in other solid tumors. Pediatric brain tumors: NCT05521984. Standard dosing:  Canagliflozin: 100 to 300 mg/day.  Dapagliflozin: 5 to 10 mg/day. | [7-9] |
| Thiazolidinediones (e.g., pioglitazone and rosiglitazone). | Increased insulin sensitivity, PPAR-γ activation, apoptosis, growth arrest, cell differentiation. | Primarily via CYP2C8/CYP2C9. Possible interactions with ketoconazole and itraconazole. Monitoring of cardiac function and liver function is recommended. Contraindications and long-term safety discussed in [10]. | For cancer therapy, administration followed antidiabetic dosing, e.g., pioglitazone: 15 to 30 mg/day, maximum dose 45 mg/day; rosiglitazone: 4 mg q.d. or b.i.d. | [11, 12] |
| DPP-4 inhibitors (gliptins, e.g., sitagliptin, saxagliptin, linagliptin, alogliptin, vildagliptin). | Antiproliferative effects but may negatively influence chemoresistance and invasion. Recommended use in cancer unresolved. | Rare cases of pancreatitis and hypersensitivity have been reported [13]. E.g., sitagliptin mostly not metabolized, minor metabolism via CYP3A4 and CYP2C8. Saxagliptin is reported as the only DPP-4 inhibitor undergoing extensive hepatic metabolism via CYP3A4. | Dosing for type 2 diabetes described in [14]. Use for cancer therapy and chemoprevention is considered in [15]. Might benefit from co-prescription with metformin to avoid undesired effects [16]. | [17] |
| GLP-1 agonists, e.g., semaglutide (oral), dulaglutide, liraglutide, exenatide, lixisenatide, as well as tirzepatide (subcutaneous). | Glucose availability (improved GKI), weight loss, chemosensitivity. | Incretin mimetics. Exendin-4 analogs (exenatide and lixisenatide) are metabolized by the kidneys, while human GLP-1RA (liraglutide, semaglutide, albiglutide, dulaglutide) are eliminated by proteolytic degradation, thus not involving CYP or transporter-mediated drug interactions. Frequent transient gastrointestinal symptoms. Rare side effects may include allergic reactions, pancreatitis, and cholecystitis [18, 19]. | Dosing discussed in [20]. The utility of GLP-1 analogs in cancer is still under evaluation [21]. Could be considered for weight loss and blood glucose control in overweight or diabetic patients. | [22, 23] |
| Bromocriptine. | Cytotoxicity, chemosensitivity, NF-κB pathway. | Primarily via CYP3A4. Rare but serious side effects may include hallucinations, valvular heart disease, fibrosis and syncope [24]. | Dosing for type 2 diabetes: 0.8 mg/day, weekly increase of 0.8 mg until desired glycemic control, maximum dose 4.8 mg/day [24]. Investigated primarily against pituitary tumors but also hormone-independent tumors. | [25] |
| Alpha-glucosidase inhibitors (e.g., acarbose, voglibose, miglitol). | Reduces carbohydrate absorption. | Not metabolized, may cause mild gastrointestinal disturbances, liver function abnormalities and allergic reactions [26]. | Potential GKI improvement by limiting the total amount of absorbed carbohydrates. E.g., acarbose, maximum daily dose 100 mg t.i.d. [27]. | [28] |
| Research-phase glucose targeting compounds. | Glycolysis inhibition at the substrate, enzyme, or transport level. | Metabolization and pharmacokinetics/dynamics for each compound would require careful evaluation and dose-escalation during trial approval, based on the available preclinical and clinical data.  Increased potency requires close monitoring. Functionally relevant keto-adaptation is a pre-requisite for the safe application of anti-glycolytic agents, such as 2-DG or 3-BP, which has not been considered in most clinical trials to date. | Clinical stage: e.g., 2DG, 3-BP, lonidamine, hydrazine compounds, TLN-232/CAP-232, imatinib, silybin, AT-101, AZD396, resveratrol. KMT is hypothesized to provide synergy with PI3K inhibitors [29].  Preclinical or tool compounds: e.g., 3PO, N4A, YZ9, PGMI-004A, MJE3, shikonin/alkannin, ML265, FX11, quinoline 3-sulfonamides, 6-AN, oxythiamine, genistein-27, benserazide, WZB117, STF-31, pachymic acid, galloflavin, FX11AR-C155858, syrosingopine, sodium oxamate, RS621, etc. | [30-35] |
| **GLUTAMINOLYSIS TARGETING** | | | | |
| Sodium or glycerol phenylbutyrate. | Glutamine depletion. | Metabolized by liver and kidney. 80–100% excreted within 24 h as conjugation product, phenylacetylglutamine. Rare but serious side effects may include increased sodium intake liver toxicity or pancreatitis, although these may be correlated with the primary indication for urea cycle disorders. Safety in cancer discussed in [36]. | Typical dosing for urea cycle disorders: 9.9 - 13.0 g/m^2^/day. Early phase clinical studies in cancer suggested 180 to 360 mg/kg/day [37], or up to 27 g/day [38, 39]. Off-label use also reported for neurodegenerative diseases, phase 2 clinical trials ongoing [40]. Other formulations such as L-ornithine phenylacetate are being explored [41]. | [40, 42] |
| L-asparaginase. | Asparagine and glutamine depletion. | Safety profile discussed in [43].  In the context of KMT, we would advise minimal effective dosing for glutamine depletion in combination with direct glutaminolysis targeting such as DON. | Approved for acute lymphoblastic leukemia and lymphoblastic lymphoma. Parenteral administration (IM or IV). Variable dosing, half-life and glutamine depletion depending on the formulation [44-46]. Clinical trials in solid tumors ongoing (e.g., NCT05631327). | [47, 48] |
| Research-phase glutamine targeting compounds. | Glutaminolysis at the substrate, enzyme, or transport level. | Variable safety and efficacy depending on the proposed mechanism and potency. Close monitoring and dose escalation in the context of KMT (not only as a single addition to SOC) will be required in future clinical research.  As a prototypic drug from this class, possible drug interactions for DON are not well characterized, but it had an acceptable safety profile in up to phase 2a clinical trials [49]. Most adverse effects such as mucositis or GI distress resolve after interrupting therapy for 1-3 days. | Clinical stage: e.g., DON and DON prodrugs such as JHU-083 and DRP-104, EGCG, CB-839, IPN60090. Tamoxifen, raloxifene, apomorphine and ebselen studied for other indications, preclinical as glutamine inhibitors. Ceftriaxone may reduce glutaminolysis via glutamate transport. CPI-613 targets glutaminolysis-derived TCA cycle flux through α-KGDH inhibition.  As a prototypic drug from this class, DON dosing has been tested in clinical trials in the range of 0.2-0.3 mg/kg/day (low dose IV) to 0.6-1.25 mg/kg/day (high dose IV), or as 50 to 200 mg/m^2^/dose. Oral administration suggested at 0.2-1.1 mg/kg/day, as a single daily dose, or q4-6h, or q2-4 days, with lower side effects at lower doses. Preclinical or tool compounds: DON prodrugs such as Azo-DON, BPTES, compound 968, azaserine, azotomycin and acivicin, V-9302, C9.22, caudatan A, physapubescin K, aspulvinone O, GPNA, chelerythrine. | [50-62] |

**Abbreviations:** α-KGDH, Alpha-Ketoglutarate Dehydrogenase; 2-DG, 2-Deoxyglucose; 3-BP, 3-Bromopyruvate; 3PO, 3-(3-Pyridinyl)-1-(4-pyridinyl)-2-propen-1-one; 6-AN, 6-Aminonicotinamide; AMPK, Adenosine Monophosphate-Activated Protein Kinase; AT-101, Gossypol; BPTES, Bis-2-(5-phenylacetamido-1,2,4-thiadiazol-2-yl)ethyl sulfide; CYP, Cytochrome P450; DON, 6-Diazo-5-oxo-L-norleucine; EGCG, Epigallocatechin gallate; FX11, CAS 213971-34-7; GKI, Glucose Ketone Index; GLP-1, Glucagon-Like Peptide-1; GPNA, L-γ-Glutamyl-p-nitroanilide; IV, Intravenous; KD, Ketogenic Diet; KMT, Ketogenic Metabolic Therapy; mTOR, Mechanistic Target of Rapamycin; NF-κB, Nuclear Factor Kappa B; OXPHOS, Oxidative Phosphorylation; PPAR-γ, Peroxisome Proliferator-Activated Receptor Gamma; SGLT2, Sodium-glucose cotransporter 2; SOC, Standard of Care; UGT, UDP-Glucuronosyltransferase.

**References:**

1. Corcoran C, Jacobs T: **Metformin. StatPearls**. In*.*: StatPearls Publishing: Treasure Island, FL, USA; 2022.

2. Gong L, Goswami S, Giacomini KM, Altman RB, Klein TE: **Metformin pathways: pharmacokinetics and pharmacodynamics**. *Pharmacogenet Genomics* 2012, **22**(11):820-827.

3. Corcoran C, Jacobs TF: **Metformin**. In: *StatPearls [Internet].* edn.: StatPearls Publishing; 2022.

4. Schmidt K, Thatcher A, Grobe A, Hicks L, Gu H, Sears DD, Ellies LG, Kalachev L, Kroll E: **The Combined Treatment with Ketogenic Diet and Metformin Slows Tumor Growth in Two Mouse Models of Triple Negative Breast Cancer**. *Res Sq* 2023.

5. Padda IS, Mahtani AU, Parmar M: **Sodium-glucose transport protein 2 (SGLT2) inhibitors**. In: *StatPearls [Internet].* edn.: StatPearls Publishing; 2022.

6. Plewa MC, Bryant M, King-Thiele R: **Euglycemic Diabetic Ketoacidosis**. In: *StatPearls [Internet].* edn.: StatPearls Publishing; 2022.

7. Sarnoski-Brocavich S, Hilas O: **Canagliflozin (invokana), a novel oral agent for type-2 diabetes**. *P T* 2013, **38**(11):656-666.

8. Kasichayanula S, Liu X, Lacreta F, Griffen SC, Boulton DW: **Clinical pharmacokinetics and pharmacodynamics of dapagliflozin, a selective inhibitor of sodium-glucose co-transporter type 2**. *Clin Pharmacokinet* 2014, **53**(1):17-27.

9. Min SH, Oh TJ, Baek SI, Lee DH, Kim KM, Moon JH, Choi SH, Park KS, Jang HC, Lim S: **Degree of ketonaemia and its association with insulin resistance after dapagliflozin treatment in type 2 diabetes**. *Diabetes & metabolism* 2018, **44**(1):73-76.

10. Eggleton JS, Jialal I: **Thiazolidinediones**. In: *StatPearls [Internet].* edn.: StatPearls Publishing; 2021.

11. Blanquicett C, Roman J, Hart CM: **Thiazolidinediones as anti-cancer agents**. *Cancer therapy* 2008, **6**(A):25.

12. Maideen NMP: **Thiazolidinediones and their Drug Interactions involving CYP enzymes**. *American Journal of Physiology, Biochemistry Pharmacology* 2018, **8**(2):1-8.

13. Kasina SVSK, Baradhi KM: **Dipeptidyl Peptidase IV (DPP IV) Inhibitors**. *StatPearls Publishing: Treasure Island, FL, USA* 2022.

14. Dungan K, DeSantis A: **Dipeptidyl peptidase-4 (DPP-4) inhibitors for the treatment of type 2 diabetes mellitus**. *U: UpToDate, Mulder JE ur UpToDate Waltham, MA: UpToDate* 2017.

15. Busek P, Duke-Cohan JS, Sedo A: **Does DPP-IV Inhibition Offer New Avenues for Therapeutic Intervention in Malignant Disease?** *Cancers (Basel)* 2022, **14**(9):2072.

16. Kawakita E, Koya D, Kanasaki K: **CD26/DPP-4: Type 2 Diabetes Drug Target with Potential Influence on Cancer Biology**. *Cancers (Basel)* 2021, **13**(9):2191.

17. Vincent SH, Reed JR, Bergman AJ, Elmore CS, Zhu B, Xu S, Ebel D, Larson P, Zeng W, Chen L *et al*: **Metabolism and excretion of the dipeptidyl peptidase 4 inhibitor [14C]sitagliptin in humans**. *Drug metabolism and disposition: the biological fate of chemicals* 2007, **35**(4):533-538.

18. He L, Wang J, Ping F, Yang N, Huang J, Li Y, Xu L, Li W, Zhang H: **Association of Glucagon-Like Peptide-1 Receptor Agonist Use With Risk of Gallbladder and Biliary Diseases: A Systematic Review and Meta-analysis of Randomized Clinical Trials**. *JAMA Intern Med* 2022, **182**(5):513-519.

19. Collins L, Costello RA: **Glucagon-like Peptide-1 Receptor Agonists**. In: *StatPearls [Internet].* edn.: StatPearls Publishing; 2022.

20. Almandoz JP, Lingvay I, Morales J, Campos C: **Switching between glucagon-like peptide-1 receptor agonists: rationale and practical guidance**. *Clinical Diabetes* 2020, **38**(4):390-402.

21. Nomiyama T, Yanase T: **GLP-1 receptor agonist as treatment for cancer as well as diabetes: beyond blood glucose control**. *Expert Rev Endocrinol Metab* 2016, **11**(4):357-364.

22. Collins L, Costello R: **Glucagon-like peptide-1 receptor agonists. In: StatPearls. Treasure Island (FL)**. *StatPearls Publishing: Treasure Island, FL, USA* 2022.

23. Jastreboff AM, Aronne LJ, Ahmad NN, Wharton S, Connery L, Alves B, Kiyosue A, Zhang S, Liu B, Bunck MC: **Tirzepatide once weekly for the treatment of obesity**. *New England Journal of Medicine* 2022, **387**(3):205-216.

24. Ozery M, Wadhwa R: **Bromocriptine**. In: *StatPearls.* edn. Treasure Island (FL): StatPearls Publishing; 2024.

25. Seo EJ, Sugimoto Y, Greten HJ, Efferth T: **Repurposing of Bromocriptine for Cancer Therapy**. *Front Pharmacol* 2018, **9**:1030.

26. Akmal M, Wadhwa R: **Alpha Glucosidase Inhibitors**. In*.*: J National Center for Biotechnology Information. StatPearls; 2022.

27. McIver L, Tripp J: **Acarbose**. *StatPearls Treasure Island : StatPearls Publishing* 2022.

28. Akmal M, Patel P, Wadhwa R: **Alpha Glucosidase Inhibitors**. In: *StatPearls.* edn. Treasure Island (FL): StatPearls Publishing; 2024.

29. Hopkins BD, Pauli C, Du X, Wang DG, Li X, Wu D, Amadiume SC, Goncalves MD, Hodakoski C, Lundquist MR: **Suppression of insulin feedback enhances the efficacy of PI3K inhibitors**. *Nature* 2018, **560**(7719):499-503.

30. Pelicano H, Martin D, Xu R, and, Huang PJO: **Glycolysis inhibition for anticancer treatment**. 2006, **25**(34):4633-4646.

31. Qian Y, Wang X, Chen X: **Inhibitors of glucose transport and glycolysis as novel anticancer therapeutics**. *World J Transl Med* 2014, **3**(2):37-57.

32. Kozal K, Jozwiak P, Krzeslak A: **Contemporary Perspectives on the Warburg Effect Inhibition in Cancer Therapy**. *Cancer Control* 2021, **28**:10732748211041243.

33. Granchi C, Minutolo F: **Anticancer agents that counteract tumor glycolysis**. *ChemMedChem* 2012, **7**(8):1318-1350.

34. Xiao C, Tian H, Zheng Y, Yang Z, Li S, Fan T, Xu J, Bai G, Liu J, Deng Z *et al*: **Glycolysis in tumor microenvironment as a target to improve cancer immunotherapy**. *Front Cell Dev Biol* 2022, **10**:1013885.

35. Integrative P: **Hydrazine Sulfate (PDQ®): Health Professional Version**. *PDQ Cancer Information Summaries* 2002.

36. Camacho LH, Olson J, Tong WP, Young CW, Spriggs DR, Malkin MG: **Phase I dose escalation clinical trial of phenylbutyrate sodium administered twice daily to patients with advanced solid tumors**. *Invest New Drugs* 2007, **25**(2):131-138.

37. Darmaun D, Welch S, Rini A, Sager BK, Altomare A, Haymond MWJAJoP-E, Metabolism: **Phenylbutyrate-induced glutamine depletion in humans: effect on leucine metabolism**. 1998, **274**(5):E801-E807.

38. Phuphanich S, Baker SD, Grossman SA, Carson KA, Gilbert MR, Fisher JD, Carducci MA: **Oral sodium phenylbutyrate in patients with recurrent malignant gliomas: a dose escalation and pharmacologic study**. *Neuro Oncol* 2005, **7**(2):177-182.

39. Gilbert J, Baker SD, Bowling MK, Grochow L, Figg WD, Zabelina Y, Donehower RC, Carducci MA: **A phase I dose escalation and bioavailability study of oral sodium phenylbutyrate in patients with refractory solid tumor malignancies**. *Clin Cancer Res* 2001, **7**(8):2292-2300.

40. Paganoni S, Macklin EA, Hendrix S, Berry JD, Elliott MA, Maiser S, Karam C, Caress JB, Owegi MA, Quick A *et al*: **Trial of Sodium Phenylbutyrate-Taurursodiol for Amyotrophic Lateral Sclerosis**. *N Engl J Med* 2020, **383**(10):919-930.

41. Safadi R, Rahimi RS, Thabut D, Bajaj JS, Ram Bhamidimarri K, Pyrsopoulos N, Potthoff A, Bukofzer S, Wang L, Jamil K *et al*: **Pharmacokinetics/pharmacodynamics of L-ornithine phenylacetate in overt hepatic encephalopathy and the effect of plasma ammonia concentration reduction on clinical outcomes**. *Clin Transl Sci* 2022, **15**(6):1449-1459.

42. Kusaczuk M, Bartoszewicz M, Cechowska-Pasko M: **Phenylbutyric Acid: simple structure - multiple effects**. *Current pharmaceutical design* 2015, **21**(16):2147-2166.

43. Hijiya N, van der Sluis IM: **Asparaginase-associated toxicity in children with acute lymphoblastic leukemia**. *Leukemia & lymphoma* 2016, **57**(4):748-757.

44. Maese L, Rau RE: **Current Use of Asparaginase in Acute Lymphoblastic Leukemia/Lymphoblastic Lymphoma**. *Front Pediatr* 2022, **10**:902117.

45. Asselin B, Rizzari C: **Asparaginase pharmacokinetics and implications of therapeutic drug monitoring**. *Leukemia & lymphoma* 2015, **56**(8):2273-2280.

46. Avramis VI, Panosyan EH: **Pharmacokinetic/pharmacodynamic relationships of asparaginase formulations: the past, the present and recommendations for the future**. *Clin Pharmacokinet* 2005, **44**(4):367-393.

47. Blachier J, Cleret A, Guerin N, Gil C, Fanjat JM, Tavernier F, Vidault L, Gallix F, Rama N, Rossignol R *et al*: **L-asparaginase anti-tumor activity in pancreatic cancer is dependent on its glutaminase activity and resistance is mediated by glutamine synthetase**. *Exp Cell Res* 2023, **426**(2):113568.

48. Chan WK, Horvath TD, Tan L, Link T, Harutyunyan KG, Pontikos MA, Anishkin A, Du D, Martin LA, Yin E *et al*: **Glutaminase Activity of L-Asparaginase Contributes to Durable Preclinical Activity against Acute Lymphoblastic Leukemia**. *Mol Cancer Ther* 2019, **18**(9):1587-1592.

49. Lemberg KM, Vornov JJ, Rais R, Slusher BS: **We're not “DON” yet: optimal dosing and prodrug delivery of 6-Diazo-5-oxo-L-norleucine**. *Mol Cancer Ther* 2018, **17**(9):1824-1832.

50. Altman BJ, Stine ZE, Dang CV: **From Krebs to clinic: glutamine metabolism to cancer therapy**. *Nature Reviews Cancer* 2016, **16**(10):619-634.

51. Chen L, Cui H: **Targeting Glutamine Induces Apoptosis: A Cancer Therapy Approach**. *Int J Mol Sci* 2015, **16**(9):22830-22855.

52. Magill G, Myers W, Reilly H, Putnam R, Magill J, Sykes M, Escher G, Karnofsky D, Burchenal J: **Pharmacological and initial therapeutic observations on 6‐Diazo‐5‐Oxo‐L‐Norleucine (Don) in human neoplastic disease**. *Cancer* 1957, **10**(6):1138-1150.

53. Xu X, Meng Y, Li L, Xu P, Wang J, Li Z, Bian J: **Overview of the Development of Glutaminase Inhibitors: Achievements and Future Directions**. *J Med Chem* 2019, **62**(3):1096-1115.

54. RK EC, Rodrigues CT, JC HC, Paradela LS, Dias MM, Novaes da Silva B, de Valega Negrao CVZ, Goncalves KA, Ascencao CFR, Adamoski D *et al*: **High-Throughput Screening Reveals New Glutaminase Inhibitor Molecules**. *ACS Pharmacol Transl Sci* 2021, **4**(6):1849-1866.

55. Todorova VK, Kaufmann Y, Luo S, Klimberg VS: **Tamoxifen and raloxifene suppress the proliferation of estrogen receptor-negative cells through inhibition of glutamine uptake**. *Cancer Chemother Pharmacol* 2011, **67**(2):285-291.

56. Thomas AG, Rojas C, Tanega C, Shen M, Simeonov A, Boxer MB, Auld DS, Ferraris DV, Tsukamoto T, Slusher BS: **Kinetic characterization of ebselen, chelerythrine and apomorphine as glutaminase inhibitors**. *Biochem Biophys Res Commun* 2013, **438**(2):243-248.

57. Catane R, Von Hoff DD, Glaubiger DL, Muggia FM: **Azaserine, DON, and azotomycin: three diazo analogs of L-glutamine with clinical antitumor activity**. *Cancer Treat Rep* 1979, **63**(6):1033-1038.

58. Soth MJ, Le K, Di Francesco ME, Hamilton MM, Liu G, Burke JP, Carroll CL, Kovacs JJ, Bardenhagen JP, Bristow CA *et al*: **Discovery of IPN60090, a Clinical Stage Selective Glutaminase-1 (GLS-1) Inhibitor with Excellent Pharmacokinetic and Physicochemical Properties**. *J Med Chem* 2020, **63**(21):12957-12977.

59. Xu H, Zheng M, Yang C, Wang K, Lv Z, Liu Z, Tang Z, Chen X: **Azo-based hypoxic-activated 6-diazo-5-oxo-L-norleucine (DON) prodrug combined with vascular disrupting agent nanoparticles for tumor-selective glutamine metabolism blockade**. *Chemical Engineering Journal* 2024, **481**:148281.

60. Lewerenz J, Albrecht P, Tien ML, Henke N, Karumbayaram S, Kornblum HI, Wiedau-Pazos M, Schubert D, Maher P, Methner A: **Induction of Nrf2 and xCT are involved in the action of the neuroprotective antibiotic ceftriaxone in vitro**. *J Neurochem* 2009, **111**(2):332-343.

61. Rothstein JD, Patel S, Regan MR, Haenggeli C, Huang YH, Bergles DE, Jin L, Dykes Hoberg M, Vidensky S, Chung DS *et al*: **Beta-lactam antibiotics offer neuroprotection by increasing glutamate transporter expression**. *Nature* 2005, **433**(7021):73-77.

62. de Groot JF, Liu TJ, Fuller G, Yung WK: **The excitatory amino acid transporter-2 induces apoptosis and decreases glioma growth in vitro and in vivo**. *Cancer Res* 2005, **65**(5):1934-1940.
