# Supplementary material for: Clinical research framework proposal for ketogenic metabolic therapy in glioblastoma
Source: BMC Med. 2024 Dec 5;22:578. doi: 10.1186/s12916-024-03775-4 (PMC11622503; doi:10.1186/s12916-024-03775-4)
Supplement: Supplementary file 6 — Supplementary Material 6. [file 12916_2024_3775_MOESM6_ESM.docx]

**Supplementary Table 3.** Drug repurposing and adjuvant therapies for the targeting of cancer-associated pathways and tumor microenvironment in GBM.

| **Drug or intervention** | **Core pathways** | **Safety and biotransformation** | **Commentary and suggested use** | **References** |
| --- | --- | --- | --- | --- |
| Mebendazole. | Disrupts microtubule formation and polymerization, angiogenesis, invasion, chemosensitization, glucose uptake, BRAF-MEK-ERK pathway. | Generally safe; rare serious effects include acute liver injury and allergic reactions [1]. Tumor growth kinetics should be monitored to elucidate differential effects across tumor subtypes [2]. Liver metabolism, enzymes unknown (possibly CYP family). Plasma levels decrease by CYP450 inducers and could be potentially increased via CYP450 inhibitors [3]. Avoid concomitant use of mebendazole and metronidazole [4]. | Proposed dosing variable, but up to 200 mg/kg/day demonstrated long-term safety, while dosing at 50 mg/kg reached acceptable steady-state plasma concentrations [5]. Additional clinical trials for GBM ongoing (NCT01729260, NCT01837862, NCT02644291). | [6] |
| Hyperbaric oxygen therapy (HBOT). | ROS production, reversal of hypoxia, angiogenesis, metastasis, radio/chemosensitization. | Safety reviewed in [7, 8]. Absolute contraindication: untreated pneumothorax. Relative contraindications: doxorubicin, bleomycin, disulfiram, cisplatin and mafenide, as well as COPD, asthma, pregnancy, epilepsy, respiratory infections and other serious preexisting conditions [9, 10]. | As an adjuvant for GBM or other cancers, sessions usually start at 1.5 ATA and escalate up to 2.5 ATA, typically for 60-90 min, 3 to 5 days/week [11]. | [12-14] |
| Vitamin C (intravenous). | ROS production (pro-oxidant), cytotoxicity, radio/chemosensitization, immune modulation. | High long-term safety profile [15]. Renal excretion, monitor fluid balance. Intravenous administration requires medical supervision and screening for preexisting contraindications [16, 17]. | Oral administration up to 2,000-6,000 mg/day is safe and well tolerated, but unlikely to achieve effective plasma concentrations [18]. Intravenous infusion for cancer therapy is generally up to 1.5 g/kg, 2 or 3 times a week, for several weeks or months, or adjusted to reach a specific concentration target [19]. Potential synergy with HBOT [20]. | [21] |
| Dichloroacetate (DCA). | ROS production, radio/chemosensitization, cytotoxicity. | Unknown; clearance decreases after multiple doses [22]. Pharmacogenetic testing (GSTZ1) can predict accumulation [23]. Investigational for cancer; reported adverse effects include neuropathy, liver toxicity and tumor lysis syndrome [24]. | Human clinical trials ranged from 10 to 50 mg/kg/day [25]. Empirically, DCA can be combined with thiamine/benfotiamine (vitamin B1), acetyl-L-carnitine and alpha lipoic acid to reduce the risk of reversible peripheral neuropathy, as well as proton pump inhibitors (e.g., omeprazole, pantoprazole) to avoid gastric irritation with oral formulations [26]. Ongoing clinical trial in recurrent GBM (NCT05120284). | [27] |
| Hyperthermia. | Cytotoxicity, apoptosis, radiosensitization, reduced adaptive flexibility. | Safety depends on maximal temperature, localization, and technique (e.g., laser interstitial therapy, magnetic, ultrasound, radiofrequency, or microwave hyperthermia). | Different modalities and previous studies in neuro-oncology discussed in [28]. Due to resurgence in popularity, more than 100 clinical trials for various tumor subtypes are ongoing, mostly as a synergistic adjunct with SOC. Active GBM trial using modulated electro-hyperthermia: NCT06140875. | [29-31] |
| Photodynamic and sonodynamic therapy. | ROS production or drug delivery by activation of sensitizing agents. | Non-invasive (before or after surgical debulking) or invasive (intraoperative application or implantable devices). Sonodynamic therapy has greater potential non-invasive tissue penetration. | A variety of competing photo/sonosensitizers are being tested in preclinical models, which could be additionally adapted for targeted drug delivery (e.g., nanoparticles or platelet carriers). Ongoing clinical trials are focusing mostly on the porphyrin precursor 5-ALA. | [32, 33] |
| Disulfiram. | ROS production, reduces inflammation, invasion, MGMT inhibition, activates JNK and P38 pathways. | Primarily via CYP2E1 (inhibitor), CYP3A4 and CYP2A6. Chronic use might modulate other enzymes. Acceptable risk profile; as per primary indication, cannot be combined with any alcohol-related metabolite (e.g., paraldehyde) and metronidazole [34]. Serious side effects are rare and dose-dependent, including hepatotoxicity, psychosis, seizures, peripheral neuropathy, and optic neuritis [35]. | Dosing in GBM, 250 to 400 mg b.i.d. [36]. Usually combined with copper gluconate (e.g., 8 mg of elemental copper). Cannot be administered simultaneously with HBOT due to increased risk of oxygen toxicity. | [37, 38] |
| COX2-specific NSAIDs (e.g., celecoxib, rofecoxib, parecoxib, etoricoxib, valdecoxib). | Inflammation, apoptosis, antiangiogenic, anti-proliferative activity, autophagy. | E.g., celecoxib, largely via CYP2C9, with some contribution from CYP3A4, CYP2C8 and CYP2D6 (inhibitor). Relative long-term cardiovascular risk; monitor high-risk patients [39]. | Increased interest for cancer therapy [40]. E.g., celecoxib, dosing is variable according to indication, typically 100 to 400 mg/day. Part of the CUSP9 trial [36]. | [41-43] |
| Diclofenac. | Inflammation, indirectly on angiogenesis, apoptosis, and radio/chemo sensitization. | Primarily via CYP2C9. Evaluate risk of bleeding, kidney and liver toxicity, and monitor cardiovascular profile [44]. | Reported doses in cancer between 50 to 200 mg/day (empirically combined with alpha-lipoic acid and hydroxycitric acid) [45]. No ongoing clinical trials in GBM. | [46-48] |
| Acetyl salicylic acid (aspirin), ibuprofen and other NSAIDs. | Inflammation, COX dependent and independent mechanisms. | E.g., aspirin primarily via UGT1A6, CYP2C9, and NAT2. Safety considerations of chronic administration discussed in [49]. | Variable dosing in cancer depending on the intended effect, discussed in [50]. | [51-53] |
| Itraconazole. | Angiogenesis, inhibition of Hedgehog, AKT-mTOR pathway, autophagy, synergy with other cytotoxic agents. | Primarily via CYP3A4 and CYP2C19 (strong inhibitor). Generally safe; monitor patients with hepatic/cardiac conditions [54]. | Clinical studies in cancer are usually dosed between 200 to 600 mg/day.  Part of the CUSP9 trial, 200 mg q.d. up to 200 mg b.i.d. [36]. | [55, 56] |
| Ketoconazole. | Drug efflux inhibitor at BBB, antiproliferative effects, mitophagy. | Primarily via CYP3A4 (strong inhibitor). High risk of drug interactions (e.g., benzodiazepines, antiarrhythmic drugs). Contraindicated in patients with acute or chronic liver disease, risk of bone fracture, adrenal insufficiency, and concomitant HMG-CoA reductase inhibitors (risk of myopathy) [57]. | Previously included in the CUSP9 protocol (200 mg b.i.d.) [58]. In prostate cancer, clinical trials dosed at 200-400 mg, t.i.d. [59]. | [60] |
| Chloroquine and hydroxychloroquine. | Autophagy, chemosensitization, cytotoxicity, cell proliferation and invasion. | E.g., hydroxychloroquine via CYP2C8, CYP3A4/5 (not an inhibitor), and CYP2D6 (inhibitor); chloroquine primarily via CYP2C8 and CYP3A4 (not an inhibitor). Known safety profile; long-term use associated with potential cardiac toxicity and retinopathy (requires screening and monitoring) [61, 62]. | Hydroxychloroquine, long-term dosing: 200 and 400 mg/day. Chloroquine-phosphate, long-term dosing: 250 mg/day. Ongoing clinical trials discussed in [63]. A combinatory trial with metformin, sirolimus and nelfinavir can be found in NCT05036226. | [64-66] |
| Doxycycline (tetracycline). | Cytotoxicity, ATP depletion, targets cancer cells with stem-like characteristics. | Limited evidence, possibly via CYP3A4. Generally safe; avoid in pregnancy, concomitant use of penicillin or isotretinoin; rare severe hypersensitivity reactions, intracranial hypertension and hepatotoxicity [67]. | Dosed in a GBM study at 100 mg/day [68]. Several completed and ongoing clinical trials for different malignancies. | [69] |
| Minocycline (tetracycline). | Immune regulation, invasion, cytotoxicity through apoptosis and autophagy, chemoprotection. | CYP450 mediated metabolism, including CYP3A4. Higher CNS permeability. As with other tetracyclines, rare serious effects include hypersensitivity, autoimmune syndromes, intracranial hypertension, hepatotoxicity [70]. | Typical dose in clinical trials: 200 mg/day [70]. | [71] |
| Albendazole. | ROS production, apoptosis, microtubule polymerization, inhibits HIF-1α-dependent glycolysis and VEGF. | Flavin-containing monooxygenases (FMO), CYP4A3 and CYP1A2. Could inhibit or induce other CYP activities. Generally safe; monitor liver function, myelosuppression and drug interactions [72]. | Dose escalation in cancer examined from 400 mg b.i.d. up to 1200 mg b.i.d. [73]. No ongoing clinical trials for GBM. | [74] |
| Niclosamide. | mTOR inhibition, apoptosis, invasion, radiosensitization, ROS production, STAT3 pathway. | CYP1A2 and UGT1A1. Generally safe; mild gastrointestinal symptoms; close monitoring and appropriate use in clinical research [75, 76]. | Oral dosing in cestocidal treatment is usually 2 g as a single dose [77]. Clinical trials in cancer ongoing (NCT02687009, NCT03123978 and NCT02807805), but currently not for GBM therapy. | [78, 79] |
| Low-dose naltrexone (LDN). | Intended primarily for quality of life but also regulates cell signaling and immune function. | Liver metabolism, generally considered CYP-independent, but *in vitro* inhibition of CYP2C9 and CYP2D6 was reported. Generally safe off-label use; common mild gastrointestinal symptoms, sleep disturbances [80]. | Low dosing is defined as equal to or less than 5 mg/day. Mechanisms of action in GBM require further study [81, 82]. | [83, 84] |
| Bis-choline tetrathiomolybdate | Angiogenesis. | Copper protein-binding agent. Limited safety profile; requires close monitoring and dose adjustment to specific threshold of copper depletion [85]. | Dosing reported as 90 to 120 mg/day in metastatic cancer, or 180 mg induction and 100 mg maintenance in breast cancer, adjusted to serum ceruloplasmin levels [86-88]. Optimal dosing for GBM unknown. | [89] |
| Cimetidine. | Proliferation, immunomodulation, adhesion, angiogenesis. | Variable inhibition of CYP1A2, CYP2D6, and CYP3A, CYP3A3/A4, CYP2C9, and CYP2C18. Generally safe; monitor drug interactions and renal/liver function [90]. | Dosing in GBM reported as 800 mg/day. Part of the CLOVA drug repurposing protocol [91]. | [92-94] |
| Sertraline. | Antiproliferative activity, apoptosis, autophagy, longer survival (epidemiological association). | Mainly via CYP3A4 (inhibitor) and CYP2B6, also CYP2C19 and CYP2D6 (weak inhibitor). Long-term safety profile and contraindications discussed in [95]. | Standard dosing: 50 to 400 mg/day [96]. Part of the CUSP9 trial, up to 100 mg b.i.d. [36]. | [97] |
| Valproic acid. | ROS production, cell cycle, apoptosis, HDAC inhibitor. | Primarily glucuronidation and β-oxidation, CYP-dependent oxidation is minor (2A6, 2B6, 2C19, 2C9, 2E1). Moderate inductor of CYP2A6. Known safety profile; possible gastrointestinal symptoms, rare liver toxicity, pancreatitis, teratogenicity; dose-escalation studies in cancer revealed mostly neurocognitive toxicity [98, 99]. | In epilepsy, adults start at 300 mg b.i.d. Appropriate dosing for GBM unknown, although valproate has been suggested at 800 mg/day. Part of the CLOVA protocol for recurrent GBM, together with cimetidine, lithium and olanzapine [91]. | [100, 101]. |
| Statins (e.g., atorvastatin, lovastatin, simvastatin, and pravastatin). | Apoptosis, proliferation, migration, and invasion, chemosensitization. | E.g., simvastatin and atorvastatin via CYP3A4. Generally safe; rare drug-induced liver injury, myopathy, rhabdomyolysis; educate on side effects, consider interactions [102]. | Lipid-lowering doses range between 5-80 mg/day, depending on the statin compound. Appropriate dosing and benefit in GBM unknown, clinical trials ongoing (NCT02115074, NCT02029573, NCT02104193). | [68, 103] |
| Valganciclovir and ganciclovir. | Human CMV has been detected in several GBM cohorts and may contribute to tumor progression. | E.g., valganciclovir: little to no liver metabolization. Serious side effects include myelosuppression, hepatic toxicity, hypersensitivity, teratogenicity; short-term treatment in GBM generally well tolerated [104]. | Dosing of valganciclovir in GBM suggested as 900 mg b.i.d. for 3 weeks followed by 900 mg daily [105, 106]. | [107, 108] |
| Levetiracetam. | Antiproliferative effects, autophagy, chemo/radiosensitization, inhibits HDAC and MGMT, apoptosis. | Major metabolism pathway likely not CYP dependent. Caution when combined with other CNS depressants (e.g., valproate). Generally well-tolerated; mild side effects include dizziness, drowsiness, behavioral changes; rare serious effects, allergic reactions, pancytopenia, suicidal ideation if risk factors [109]. | For prevention of epilepsy in GBM, doses range from initial 500 mg q12h up to 3000 mg/day. Associated with small improvements in survival [110, 111]. | [112, 113] |
| Dimethyl fumarate. | Chemo/radiosensitization, immune regulation, angiogenesis, apoptosis. | Likely not via CYP family. Common side effects include flushing, gastrointestinal disturbances, lymphopenia; serious rare effects include progressive multifocal leukoencephalopathy, hypersensitivity [114]. | Phase 1 dose-escalation study in GBM suggested up to 240 mg t.i.d. [115]. Prodrug formulations available for other indications [114]. | [116] |
| Auranofin. | Increased ROS production, inflammation, and immune regulation, ferroptosis. | Gold salt, excreted primarily via urine. Serious side effects include dermatological reactions, bone marrow suppression, hepatotoxicity [117]. | 3 mg q.d. or b.i.d. Part of the CUSP9 trial [36]. Most clinical trials to date explored combinations with sirolimus. | [118, 119] |
| Fluoroquinolones (e.g., levofloxacin, ciprofloxacin). | Cytotoxicity, apoptosis, immune regulation, invasion, ROS production, drug resistance. | E.g., ciprofloxacin primarily via CYP1A2 (inhibitor) and CYP2C9; moderate CYP3A4 inhibitor.  Levofloxacin likely via CYP2C9.  Rare serious side effects of fluoroquinolones include tendon rupture, peripheral neuropathy, central nervous system effects [120]. | Usual dosage of ciprofloxacin is 250 mg b.i.d. or 500 mg b.i.d. Recommended dosing for GBM therapy unknown. Can potentiate 5-aminolevulinic acid-induced cytotoxicity [121]. Fluoroquinolones in cancer reviewed in [122]. | [123-126] |
| Captopril. | Invasion, angiogenesis, inflammation, may improve cerebral edema. | Likely no significant CYP interactions. Rare serious effects include angioedema, renal impairment, hematological abnormalities; contraindications and monitoring reviewed in [127]. | As suggested in the CUSP9 trial, 100 mg q.d. to 100 mg b.i.d. [36]. Also explored for drug repurposing in other malignancies. | [128] |
| Losartan. | Angiogenesis, management of cerebral edema, immune regulation, cell proliferation and apoptosis. | Metabolites via CYP2C9 (seemingly not affected by inhibitors), CYP3A4 (seemingly not affected by inhibitors), CYP2C10, and glucuronidation. Rare serious side effects include angioedema, renal impairment, hematological abnormalities, which may preclude long-term use in cancer [129]. | Standard dosing: 25 to 100 mg/day. Recommended dosing for GBM under evaluation, NCT03951142 and [130]. | [131-133] |
| Carvedilol and other beta-adrenergic blocking agents. | Antiproliferative effects in glioma (empirical observations), chemoprotective agent. | Primarily via CYP2D6 and CYP2C9 (monitor drug interactions to avoid hypotension). Generally well-tolerated; contraindications include hypotension, AV block, heart failure, hepatic impairment [134]. | Two clinical trials in GBM intended to test up to 12.5 mg b.i.d. (NCT03861598 and NCT03980249). | [135] |
| Tricyclic antidepressants and selective serotonin reuptake inhibitors (SSRIs). | E.g., imipramine: reduces stemness, induces autophagy via PI3K/AKT/mTOR. | E.g., imipramine: CYP1A2 (inhibitor), CYP3A4, CYP2C19 (inhibitor) and CYP2D6 (inhibitor). Serious side effects include cardiac arrhythmia, serotonin syndrome, suicidal ideation.[136, 137] | Drug repurposing discussed in [138]. Suggested dose escalation of imipramine in GBM up to 200 mg/day (NCT04863950). Fluoxetine has been epidemiologically associated with increased survival in GBM patients [139]. | [140, 141] |
| Aprepitant. | Cytotoxicity, NK-1R inhibitor, reduces side effects of chemotherapy. | Metabolized via CYP3A4 (moderate inhibitor and inducer); also, CYP2C9 inducer. Generally well-tolerated [142]. | As suggested in the CUSP9 trial, 80 mg q.d. [36]. | [143, 144] |
| Sulfasalazine. | Antiproliferative effects, inflammation, radiosensitizer. | Metabolized by gut microbiome, the ABCG2 transporter might be a bioavailability limiting factor. Rare side effects include serious skin hypersensitivity reactions, hepatic toxicity, renal impairment [145]. | Dosing in clinical trials for GBM ranges from 1 to 4 g/day. Hypothesized to improve the efficacy of radiotherapy (NCT04205357); however, relatively high incidence of adverse effects warrants caution [146, 147]. | [148] |
| Protease inhibitors (e.g., ritonavir, nelfinavir and lopinavir). | Invasion, reduced pro-tumoral signaling, glucose transport, cell cycle, AKT/mTOR pathway. | Nelfinavir metabolized via CYP3A (strong inhibitor) and CYP2C19. Ritonavir CYP3A (strong inhibitor) and CYP2D6. Lopinavir CYP3A (strong inhibitor). Constitutional side effects; serious side effects include drug interactions, hypersensitivity, QT prolongation [149]. | As suggested in the CUSP9 trial, ritonavir 200 mg q.d. up to 200 mg b.i.d. [36]. Also explored for drug repurposing in other malignancies. | [150] |
| Rapamycin analogues (e.g., sirolimus, everolimus). | mTOR inhibition, apoptosis. | Primarily via CYP3A4 and P-glycoprotein (P-gp). Caution when combined with CYP inducers/inhibitors. Serious side effects include infection risk, delayed wound healing, interstitial lung disease. | mTOR inhibition in GBM therapy discussed in [151]. | [152, 153] |
| Leflunomide. | Cytotoxicity, angiogenesis, stemness, immune modulator. | CYP1A2, CYP2C19 and CYP3A4. Caution when combining with CYP inhibitors. Serious side effects include severe dermatological reactions, pancytopenia, interstitial lung disease, pneumonitis, hepatic injury [154]. | In standard dosing, loading dose 100 mg/day for three days, followed by a maintenance dose of 10 to 20 mg/day [154]. Optimal dosing and route of administration for GBM therapy unknown. | [155-157] |
| Ibudilast (Phosphodiesterase-4 inhibitor). | Immune regulation. | Not described as a clinically relevant inhibitor/inducer of CYP enzymes. Serious side effects include hepatotoxicity, allergic reactions, cardiovascular effects [158]. | 30 mg b.i.d. was suggested in a clinical trial for GBM, escalated to a maximal dose of 50 mg b.i.d. (NCT03782415) | [159] |
| Phosphodiesterase-5 inhibitors (e.g., sildenafil). | ROS production, apoptosis, chemosensitization, blood-brain tumor barrier permeability. | CYP3A4 (major pathway) and CYP2C9 (minor pathway). Rare side effects include cardiovascular events, visual disturbances, priapism [160]. | Clinical trial in GBM suggested up to 50 mg b.i.d. (NCT01817751). | [161, 162] |
| Nitroxoline. | Angiogenesis, apoptosis, invasion. | Metabolization route unclear. Long history of clinical use and well defined safety profile. Rare serious side effects include hepatotoxicity, hematological abnormalities, hypersensitivity [163]. | Common antimicrobial dose: 750 mg/day. Unclear BBB penetration; under active clinical trials in bladder cancer [164]. | [165-167] |
| Mefloquine. | Cytotoxicity, antiproliferative effects, autophagy. | Metabolized via CYP3A4. Rare serious side effects include arrhythmias and neuropsychiatric reactions [168]. | For malaria prevention, 250 mg, once a week. Dosing for GBM unknown, but a clinical trial suggested 30 mg q.d. on days 1-3 of week 1, and then days 2, 4, and 6 every other week (NCT01430351). | [169-171] |
| Clioquinol. | Apoptosis, proteasome inhibitor, angiogenesis, cytotoxicity. | Not described. Withdrawn from market in topical and oral form. Rare serious effects include neurotoxicity, subacute myelo-optic neuropathy [172]. | Novel prodrugs would require intravenous administration as a copper-complex [173]. No ongoing trials for original formulation [174]. | [175] |
| Ivermectin. | ROS production, EGFR and NF-κB inhibition, apoptosis, cell cycle arrest, reversal of drug resistance. | Mainly via CYP3A4 (weak inhibitor). Caution if combining with inhibitors or inducers of P4503A4 and MDR1 (P-gp), ABCG2 or MRP transporters. Rare serious side effects include neurotoxicity, ocular adverse effects, allergic reactions and hepatotoxicity [176]. | Standard dosing for parasitic infections from 150 to 200 µg/kg [177]. Optimal dosage for GBM unknown. Ongoing clinical trials in breast cancer (NCT05318469). | [177-179] |
| Acetazolamide. | Might alleviate vasogenic edema, inflammation, apoptosis. | Not metabolized. Common side effects include paresthesia, gastrointestinal disturbances, metabolic acidosis; rare serious effects include renal impairment, blood dyscrasias, hepatotoxicity, hypersensitivity [180]. | Suggested dosing for GBM begins at 250 mg b.i.d., up to 500 mg b.i.d. (NCT03011671). | [180, 181] |
| Isotretinoin (13-cis-retinoic acid). | Inhibition of EGFR activity, antiproliferative effects, migration, and invasion. | Primarily via CYP3A4. Serious side effects include teratogenicity, psychiatric disorders, hepatotoxicity; other adverse effects and contraindications reviewed in [182]. | Tested in newly diagnosed GBM after radiotherapy (NCT00112502), and in recurrent GBM at 60-100 mg/m^2^ per day [183]. | [184] |
| Thalidomide and related compounds (e.g., lenalidomide and pomalidomide). | Angiogenesis, immune regulation, antiproliferative activity, inflammation, apoptosis. | Evidence mixed; may be metabolized via CYP2C19. Common side effects include sedation, peripheral neuropathy, constipation; serious effects include teratogenicity, thromboembolic events, hematological toxicity [185]. | Dosing in phase 2 clinical trials in GBM usually starts at 100 or 200 mg/day, escalating slowly to 400, 600 or 1200 mg/day if well tolerated [186-188]. | [189] |
| Artesunate. | ROS production, cytotoxicity, radiosensitization, angiogenesis. | UGT1A9, UGT2B7 and CYP2A6. Rare serious side effects include allergic reactions, hemolysis, acute renal failure; dose-escalation trials recommended monitoring of reticulocytes, NTproBNP, as well as audiological and neurological exploration [190]. | Eventually not tested in the CUSP9 trial, but originally proposed at 50 mg b.i.d. [191]. In other cancer studies, up to 200 mg/day were generally well tolerated [192]. | [193] |
| Olanzapine. | Proliferation, apoptosis, migration, NF-κB activation, ROS production, radiosensitization. | Primarily via CYP1A2, also CYP2D6 and CYP3A4. Weight gain and insulin resistance are a common side effects, which needs to be balanced with KMT; rare serious effects include extrapyramidal symptoms and neuroleptic malignant syndrome [194]. | Dosing in GBM reported as 10 mg/day. Part of the CLOVA drug repurposing protocol [91]. | [195, 196] |
| Nitroglycerin. | Vasoactive properties, reversal of hypoxia, nitric oxide donor, apoptosis. | Bioactivated via liver ALDH2, metabolized to nitrite and then nitric oxide. Possible nitrate tolerance with long-term use. Serious effects include unwanted hypotensive effects (syncope), reflex tachycardia, methemoglobinemia. Contraindicated with PDE-5 inhibitors, increased intracranial pressure, severe anemia, right-sided myocardial infarction, or hypersensitivity [197]. | Transdermal patches for angina prophylaxis, 5 to 15 mg/day. No dosing reported for GBM, clinical trials in other cancers ongoing (NCT01210378, NCT01704274). | [198] |
| Memantine. | Invasion, proliferation, autophagy. NMDA-type glutamate receptor antagonist. | Liver metabolism, CYP2B6 inhibition. Rare side effects include neurologic effects, cardiac arrhythmias; precaution in cardiovascular disease and hepatic impairment [199]. | For protection against radiation toxicity, dose escalation usually ranges from 5 to 20 mg/day [200]. | [201-203] |
| Ribavirin. | Proliferation, migration, synergy with chemotherapeutics, elF4E signaling. | Seemingly not a substrate for CYP450, secondary metabolites excreted via urine. Rare serious side effects include hemolytic anemia, teratogenicity, pulmonary toxicity [204]. | Hepatitis C dosing ranges from 800 to 1500 mg/day. Optimal dosing for GBM unknown [205]. | [206, 207] |
| Chlorpromazine, fluphenazine and perphenazine. | Cell cycle arrest, apoptosis, chemo/radiosensitization, stem cell targeting. | Chlorpromazine CYP2D6 (major pathway), CYP1A2 and CYP3A4. Fluphenazine mainly via CYP2D6, minor inhibition of CYP2C9 and CYP2E1. Perphenazine via CYP2D6, CYP1A2, CYP2C19, CYP2C9, CYP3A4, minor inhibition of CYP1A2, CYP2D6. Common side effects include sedation, extrapyramidal symptoms, anticholinergic effects; serious effects include neuroleptic malignant syndrome, tardive dyskinesia, cardiovascular effects; contraindications discussed in [208]. | Clinical trials for chlorpromazine suggested 25 mg to 50 mg/day (NCT05190315 and NCT04224441). Optimal dosing for GBM unknown. Standard dosing discussed in [209]. | [210-213] |
| Amlexanox. | Inflammation, proliferation, chemosensitization. | Metabolism mediated via several CYP enzymes. Rare serious side effects include mucosal ulceration, hypersensitivity [214]. | In type 2 diabetes, 25 mg t.i.d. for 2 weeks, and then increased to 50 mg t.i.d. for an additional 10 weeks [215]. Appropriate dosing for GBM unknown. | [216] |

**Abbreviations:** 5-ALA, 5-Aminolevulinic Acid; ALDH2, Aldehyde Dehydrogenase 2; ATA, Atmospheres Absolute; BBB, Blood-Brain Barrier; CNS, Central Nervous System; COPD, Chronic Obstructive Pulmonary Disease; COX2, Cyclooxygenase-2; CYP, Cytochrome P450; EGFR, Epidermal Growth Factor Receptor; GBM, Glioblastoma Multiforme; GSTZ1, Glutathione S-transferase zeta 1; HBOT, Hyperbaric Oxygen Therapy; HDAC, Histone Deacetylase; HIF-1α, Hypoxia-Inducible Factor 1-alpha; HMG-CoA, 3-Hydroxy-3-Methylglutaryl-Coenzyme A; JNK, c-Jun N-terminal Kinase; KD, Ketogenic Diet; KMT, Ketogenic Metabolic Therapy; MGMT, O^6^-methylguanine-DNA methyltransferase; mTOR, Mechanistic Target of Rapamycin; NK-1R, Neurokinin 1 Receptor; NSAID, Nonsteroidal Anti-Inflammatory Drug; P-gp, P-Glycoprotein; P38, P38 Mitogen-Activated Protein Kinases; ROS, Reactive Oxygen Species; SOC, Standard of Care; STAT3, Signal Transducer and Activator of Transcription 3; UGT, UDP Glucuronosyltransferase; VEGF, Vascular Endothelial Growth Factor.

**References:**

1. Thakur RK, Patel SP: **Mebendazole**. In: *StatPearls.* edn. Treasure Island (FL): StatPearls Publishing; 2024.

2. Mansoori S, Fryknas M, Alvfors C, Loskog A, Larsson R, Nygren P: **A phase 2a clinical study on the safety and efficacy of individualized dosed mebendazole in patients with advanced gastrointestinal cancer**. *Sci Rep* 2021, **11**(1):8981.

3. Bekhti A, Pirotte J: **Cimetidine increases serum mebendazole concentrations. Implications for treatment of hepatic hydatid cysts**. *Br J Clin Pharmacol* 1987, **24**(3):390-392.

4. Chen KT, Twu SJ, Chang HJ, Lin RS: **Outbreak of Stevens-Johnson syndrome/toxic epidermal necrolysis associated with mebendazole and metronidazole use among Filipino laborers in Taiwan**. *Am J Public Health* 2003, **93**(3):489-492.

5. Gallia GL, Holdhoff M, Brem H, Joshi AD, Hann CL, Bai RY, Staedtke V, Blakeley JO, Sengupta S, Jarrell TC *et al*: **Mebendazole and temozolomide in patients with newly diagnosed high-grade gliomas: results of a phase 1 clinical trial**. *Neurooncol Adv* 2021, **3**(1):vdaa154.

6. Schalkwijk S, Buaben AO, Freriksen JJM, Colbers AP, Burger DM, Greupink R, Russel FGM: **Prediction of Fetal Darunavir Exposure by Integrating Human Ex-Vivo Placental Transfer and Physiologically Based Pharmacokinetic Modeling**. *Clin Pharmacokinet* 2018, **57**(6):705-716.

7. Montalbano C, Kiorpes C, Elam L, Miscioscia E, Shmalberg J: **Common Uses and Adverse Effects of Hyperbaric Oxygen Therapy in a Cohort of Small Animal Patients: A Retrospective Analysis of 2,792 Treatment Sessions**. *Front Vet Sci* 2021, **8**:764002.

8. Heyboer III M, Sharma D, Santiago W, McCulloch N: **Hyperbaric oxygen therapy: side effects defined and quantified**. *Advances in wound care* 2017, **6**(6):210-224.

9. Gawdi R, Cooper JS: **Hyperbaric contraindications**. In: *StatPearls [Internet].* edn.: StatPearls Publishing; 2022.

10. Baude J, Cooper JS: **Hyperbaric contraindicated chemotherapeutic agents**. In: *StatPearls [Internet].* edn.: StatPearls Publishing; 2021.

11. Alpuim Costa D, Sampaio-Alves M, Netto E, Fernandez G, Oliveira E, Teixeira A, Daniel PM, Bernardo GS, Amaro C: **Hyperbaric Oxygen Therapy as a Complementary Treatment in Glioblastoma-A Scoping Review**. *Front Neurol* 2022, **13**:886603.

12. Iyikesici MSJIJoH: **Feasibility study of metabolically supported chemotherapy with weekly carboplatin/paclitaxel combined with ketogenic diet, hyperthermia and hyperbaric oxygen therapy in metastatic non-small cell lung cancer**. 2019, **36**(1):445-454.

13. Ohguri T, Imada H, Narisada H, Yahara K, Morioka T, Nakano K, Miyaguni Y, Korogi Y: **Systemic chemotherapy using paclitaxel and carboplatin plus regional hyperthermia and hyperbaric oxygen treatment for non-small cell lung cancer with multiple pulmonary metastases: preliminary results**. *Int J Hyperthermia* 2009, **25**(2):160-167.

14. Moen I, Stuhr LE: **Hyperbaric oxygen therapy and cancer--a review**. *Target Oncol* 2012, **7**(4):233-242.

15. Nauman G, Gray JC, Parkinson R, Levine M, Paller CJ: **Systematic Review of Intravenous Ascorbate in Cancer Clinical Trials**. *Antioxidants (Basel)* 2018, **7**(7):89.

16. Alkhunaizi AM, Chan L: **Secondary oxalosis: a cause of delayed recovery of renal function in the setting of acute renal failure**. *J Am Soc Nephrol* 1996, **7**(11):2320-2326.

17. Wong K, Thomson C, Bailey RR, McDiarmid S, Gardner J: **Acute oxalate nephropathy after a massive intravenous dose of vitamin C**. *Aust N Z J Med* 1994, **24**(4):410-411.

18. Johnston CS: **Biomarkers for establishing a tolerable upper intake level for vitamin C**. *Nutr Rev* 1999, **57**(3):71-77.

19. Board AME: **High-Dose Vitamin C (PDQ®)**. In: *PDQ Cancer Information Summaries [Internet].* edn.: National Cancer Institute (US); 2013.

20. DeBlasi JM, Ward NP, Poff AM, Koutnik AP, Rogers CQ, D'Agostino DP: **Anti‐Cancer Effects of Ascorbic Acid and Hyperbaric Oxygen Therapy in vitro**. *The FASEB Journal* 2017, **31**:879.874-879.874.

21. Bottger F, Valles-Marti A, Cahn L, Jimenez CR: **High-dose intravenous vitamin C, a promising multi-targeting agent in the treatment of cancer**. *J Exp Clin Cancer Res* 2021, **40**(1):343.

22. Stacpoole PW, Nagaraja NV, Hutson AD: **Efficacy of dichloroacetate as a lactate‐lowering drug**. *The Journal of Clinical Pharmacology* 2003, **43**(7):683-691.

23. James MO, Stacpoole PW: **Pharmacogenetic considerations with dichloroacetate dosing**. *Pharmacogenomics* 2016, **17**(7):743-753.

24. Stacpoole PW: **The pharmacology of dichloroacetate**. *Metabolism* 1989, **38**(11):1124-1144.

25. Michelakis E, Sutendra G, Dromparis P, Webster L, Haromy A, Niven E, Maguire C, Gammer T-L, Mackey J, Fulton DJStm: **Metabolic modulation of glioblastoma with dichloroacetate**. 2010, **2**(31):31ra34-31ra34.

26. Khan A, Marier D, Marsden E, Andrews D, Eliaz I: **A novel form of dichloroacetate therapy for patients with advanced cancer: a report of 3 cases**. *Altern Ther Health Med* 2014, **20 Suppl 2**(Suppl 2):21-28.

27. Tataranni T, Piccoli C: **Dichloroacetate (DCA) and Cancer: An Overview towards Clinical Applications**. *Oxid Med Cell Longev* 2019, **2019**:8201079.

28. Skandalakis GP, Rivera DR, Rizea CD, Bouras A, Jesu Raj JG, Bozec D, Hadjipanayis CG: **Hyperthermia treatment advances for brain tumors**. *Int J Hyperthermia* 2020, **37**(2):3-19.

29. Schneider CS, Woodworth GF, Vujaskovic Z, Mishra MV: **Radiosensitization of high-grade gliomas through induced hyperthermia: Review of clinical experience and the potential role of MR-guided focused ultrasound**. *Radiother Oncol* 2020, **142**:43-51.

30. Mahmoudi K, Bouras A, Bozec D, Ivkov R, Hadjipanayis C: **Magnetic hyperthermia therapy for the treatment of glioblastoma: a review of the therapy's history, efficacy and application in humans**. *Int J Hyperthermia* 2018, **34**(8):1316-1328.

31. Szasz AM, Arrojo Alvarez EE, Fiorentini G, Herold M, Herold Z, Sarti D, Dank M: **Meta-Analysis of Modulated Electro-Hyperthermia and Tumor Treating Fields in the Treatment of Glioblastomas**. *Cancers (Basel)* 2023, **15**(3):880.

32. Bhanja D, Wilding H, Baroz A, Trifoi M, Shenoy G, Slagle-Webb B, Hayes D, Soudagar Y, Connor J, Mansouri A: **Photodynamic Therapy for Glioblastoma: Illuminating the Path toward Clinical Applicability**. *Cancers (Basel)* 2023, **15**(13):3427.

33. Mehta NH, Shah HA, D'Amico RS: **Sonodynamic Therapy and Sonosensitizers for Glioma Treatment: A Systematic Qualitative Review**. *World Neurosurg* 2023, **178**:60-68.

34. Weathermon R, Crabb DW: **Alcohol and medication interactions**. *Alcohol Res Health* 1999, **23**(1):40-54.

35. Chick J: **Safety issues concerning the use of disulfiram in treating alcohol dependence**. *Drug Saf* 1999, **20**(5):427-435.

36. Halatsch ME, Kast RE, Karpel-Massler G, Mayer B, Zolk O, Schmitz B, Scheuerle A, Maier L, Bullinger L, Mayer-Steinacker R *et al*: **A phase Ib/IIa trial of 9 repurposed drugs combined with temozolomide for the treatment of recurrent glioblastoma: CUSP9v3**. *Neurooncol Adv* 2021, **3**(1):vdab075.

37. Madan A, Parkinson A, Faiman MD: **Identification of the human P-450 enzymes responsible for the sulfoxidation and thiono-oxidation of diethyldithiocarbamate methyl ester: role of P-450 enzymes in disulfiram bioactivation**. *Alcohol Clin Exp Res* 1998, **22**(6):1212-1219.

38. Frye RF, Branch RA: **Effect of chronic disulfiram administration on the activities of CYP1A2, CYP2C19, CYP2D6, CYP2E1, and N-acetyltransferase in healthy human subjects**. *Br J Clin Pharmacol* 2002, **53**(2):155-162.

39. De Vecchis R, Baldi C, Di Biase G, Ariano C, Cioppa C, Giasi A, Valente L, Cantatrione S: **Cardiovascular risk associated with celecoxib or etoricoxib: a meta-analysis of randomized controlled trials which adopted comparison with placebo or naproxen**. *Minerva Cardioangiol* 2014, **62**(6):437-448.

40. Ye SY, Li JY, Li TH, Song YX, Sun JX, Chen XW, Zhao JH, Li Y, Wu ZH, Gao P *et al*: **The Efficacy and Safety of Celecoxib in Addition to Standard Cancer Therapy: A Systematic Review and Meta-Analysis of Randomized Controlled Trials**. *Curr Oncol* 2022, **29**(9):6137-6153.

41. Cohen B, Preuss CV: **Celecoxib**. 2018.

42. Khafaga AF, Shamma RN, Abdeen A, Barakat AM, Noreldin AE, Elzoghby AO, Sallam MA: **Celecoxib repurposing in cancer therapy: molecular mechanisms and nanomedicine-based delivery technologies**. *Nanomedicine (Lond)* 2021, **16**(19):1691-1712.

43. Toloczko-Iwaniuk N, Dziemianczyk-Pakiela D, Nowaszewska BK, Celinska-Janowicz K, Miltyk W: **Celecoxib in Cancer Therapy and Prevention - Review**. *Current drug targets* 2019, **20**(3):302-315.

44. Zapata-Morales AL, Alfaro-De la Torre MC, Hernández-Morales A, García-De la Cruz RF: **Isolation of Cultivable Bacteria Associated with the Root of Typha latifolia in a Constructed Wetland for the Removal of Diclofenac or Naproxen**. *Water, Air, & Soil Pollution* 2020, **231**(8).

45. da Veiga Moreira J, Hamraz M, Abolhassani M, Schwartz L, Jolicoeur M, Peres S: **Metabolic therapies inhibit tumor growth in vivo and in silico**. *Sci Rep* 2019, **9**(1):3153.

46. Davies NM, Anderson KE: **Clinical pharmacokinetics of diclofenac. Therapeutic insights and pitfalls**. *Clin Pharmacokinet* 1997, **33**(3):184-213.

47. Alfaro R, Davis D: **Diclofenac. In xPharm: The Comprehensive Pharmacology Reference**. In*.*: StatPearls Publishing: Treasure Island, FL, USA; 2022.

48. Pantziarka P, Sukhatme V, Bouche G, Meheus L, Sukhatme VP: **Repurposing Drugs in Oncology (ReDO)-diclofenac as an anti-cancer agent**. *Ecancermedicalscience* 2016, **10**:610.

49. de Labry Lima AO, Salamanca-Fernández E, Del Rey EA, Hoces AM, Vera MG, Tamayo CB: **Safety considerations during prescription of non-steroidal anti-inflammatory drugs (NSAIDs), through a review of systematic reviews**. In: *Anales del sistema sanitario de Navarra: 2021*; 2021: 261-273.

50. Ozleyen A, Yilmaz YB, Donmez S, Atalay HN, Antika G, Tumer TB: **Looking at NSAIDs from a historical perspective and their current status in drug repurposing for cancer treatment and prevention**. *J Cancer Res Clin Oncol* 2023, **149**(5):2095-2113.

51. Palikhe NS, Kim SH, Nam YH, Ye YM, Park HS: **Polymorphisms of Aspirin-Metabolizing Enzymes CYP2C9, NAT2 and UGT1A6 in Aspirin-Intolerant Urticaria**. *Allergy Asthma Immunol Res* 2011, **3**(4):273-276.

52. Chirasani SR, Leukel P, Gottfried E, Hochrein J, Stadler K, Neumann B, Oefner PJ, Gronwald W, Bogdahn U, Hau P *et al*: **Diclofenac inhibits lactate formation and efficiently counteracts local immune suppression in a murine glioma model**. *Int J Cancer* 2013, **132**(4):843-853.

53. Langley R, Burdett S, Tierney J, Cafferty F, Parmar M, Venning G: **Aspirin and cancer: has aspirin been overlooked as an adjuvant therapy?** *Br J Cancer* 2011, **105**(8):1107-1113.

54. Kurn H, Wadhwa R: **Itraconazole. NIH National Library of Medicine**. *National Center for Biotechnology Information StatPearls* 2022.

55. Isoherranen N, Kunze KL, Allen KE, Nelson WL, Thummel KE: **Role of itraconazole metabolites in CYP3A4 inhibition**. *Drug metabolism and disposition: the biological fate of chemicals* 2004, **32**(10):1121-1131.

56. Pounds R, Leonard S, Dawson C, Kehoe S: **Repurposing itraconazole for the treatment of cancer**. *Oncol Lett* 2017, **14**(3):2587-2597.

57. Daneshmend TK, Warnock DW: **Clinical pharmacokinetics of ketoconazole**. *Clin Pharmacokinet* 1988, **14**(1):13-34.

58. Kast RE, Boockvar JA, Bruning A, Cappello F, Chang WW, Cvek B, Dou QP, Duenas-Gonzalez A, Efferth T, Focosi D *et al*: **A conceptually new treatment approach for relapsed glioblastoma: coordinated undermining of survival paths with nine repurposed drugs (CUSP9) by the International Initiative for Accelerated Improvement of Glioblastoma Care**. *Oncotarget* 2013, **4**(4):502-530.

59. Tresnanda RI, Pramod SV, Safriadi F: **Ketoconazole for the Treatment of Docetaxel-Naïve Metastatic Castration-Resistant Prostate Cancer (mCRPC): A Systematic Review**. *Asian Pacific Journal of Cancer Prevention: APJCP* 2021, **22**(10):3101.

60. Martin P, Gillen M, Millson D, Oliver S, Brealey C, Grossbard EB, Baluom M, Lau D, Sweeny D, Mant T *et al*: **Effects of CYP3A4 Inhibitors Ketoconazole and Verapamil and the CYP3A4 Inducer Rifampicin on the Pharmacokinetic Parameters of Fostamatinib: Results from In Vitro and Phase I Clinical Studies**. *Drugs R D* 2016, **16**(1):81-92.

61. Abdel-Aziz AK, Saadeldin MK, Salem AH, Ibrahim SA, Shouman S, Abdel-Naim AB, Orecchia R: **A Critical Review of Chloroquine and Hydroxychloroquine as Potential Adjuvant Agents for Treating People with Cancer**. *Future Pharmacology* 2022, **2**(4):431-443.

62. Stokkermans TJ, Goyal A, Trichonas G: **Chloroquine and hydroxychloroquine toxicity**. 2019.

63. Verbaanderd C, Maes H, Schaaf MB, Sukhatme VP, Pantziarka P, Sukhatme V, Agostinis P, Bouche G: **Repurposing Drugs in Oncology (ReDO)-chloroquine and hydroxychloroquine as anti-cancer agents**. *Ecancermedicalscience* 2017, **11**:781.

64. Furst DE: **Pharmacokinetics of hydroxychloroquine and chloroquine during treatment of rheumatic diseases**. *Lupus* 1996, **5 Suppl 1**(1_suppl):S11-15.

65. Projean D, Baune B, Farinotti R, Flinois JP, Beaune P, Taburet AM, Ducharme J: **In vitro metabolism of chloroquine: identification of CYP2C8, CYP3A4, and CYP2D6 as the main isoforms catalyzing N-desethylchloroquine formation**. *Drug metabolism and disposition: the biological fate of chemicals* 2003, **31**(6):748-754.

66. Li X, Höhl R, Sörgel F, Fuhr U: **The parent drugs chloroquine and hydroxychloroquine do not inhibit human CYP3A activity in vitro**. *European Journal of Clinical Pharmacology* 2020, **76**(10):1481-1482.

67. Shutter M, Akhondi H: **Tetracycline**. *StatPearls* 2022.

68. Agrawal S, Vamadevan P, Mazibuko N, Bannister R, Swery R, Wilson S, Edwards S: **A New Method for Ethical and Efficient Evidence Generation for Off-Label Medication Use in Oncology (A Case Study in Glioblastoma)**. *Front Pharmacol* 2019, **10**:681.

69. Holmes NE, Charles PGP: **Safety and Efficacy Review of Doxycycline**. *Clinical Medicine Insights-Therapeutics* 2009, **1**:471-482.

70. Nazarian S, Akhondi H: **Minocycline**. 2020.

71. Nelis HJ, De Leenheer AP: **Metabolism of minocycline in humans**. *Drug metabolism and disposition: the biological fate of chemicals* 1982, **10**(2):142-146.

72. Malik K, Dua A: **Albendazole**. In: *StatPearls [Internet].* edn.: StatPearls Publishing; 2022.

73. Pourgholami MH, Szwajcer M, Chin M, Liauw W, Seef J, Galettis P, Morris DL, Links M: **Phase I clinical trial to determine maximum tolerated dose of oral albendazole in patients with advanced cancer**. *Cancer Chemother Pharmacol* 2010, **65**(3):597-605.

74. Rawden HC, Kokwaro GO, Ward SA, Edwards G: **Relative contribution of cytochromes P-450 and flavin-containing monoxygenases to the metabolism of albendazole by human liver microsomes**. *Br J Clin Pharmacol* 2000, **49**(4):313-322.

75. Burock S, Daum S, Keilholz U, Neumann K, Walther W, Stein U: **Phase II trial to investigate the safety and efficacy of orally applied niclosamide in patients with metachronous or sychronous metastases of a colorectal cancer progressing after therapy: the NIKOLO trial**. *BMC Cancer* 2018, **18**(1):297.

76. Schweizer MT, Haugk K, McKiernan JS, Gulati R, Cheng HH, Maes JL, Dumpit RF, Nelson PS, Montgomery B, McCune JS: **A phase I study of niclosamide in combination with enzalutamide in men with castration-resistant prostate cancer**. *PLoS ONE* 2018, **13**(6):e0198389.

77. Li Y, Li PK, Roberts MJ, Arend RC, Samant RS, Buchsbaum DJ: **Multi-targeted therapy of cancer by niclosamide: A new application for an old drug**. *Cancer Lett* 2014, **349**(1):8-14.

78. Lu D, Ma Z, Zhang T, Zhang X, Wu B: **Metabolism of the anthelmintic drug niclosamide by cytochrome P450 enzymes and UDP-glucuronosyltransferases: metabolite elucidation and main contributions from CYP1A2 and UGT1A1**. *Xenobiotica* 2016, **46**(1):1-13.

79. Fan X, Li H, Ding X, Zhang QY: **Contributions of Hepatic and Intestinal Metabolism to the Disposition of Niclosamide, a Repurposed Drug with Poor Bioavailability**. *Drug metabolism and disposition: the biological fate of chemicals* 2019, **47**(7):756-763.

80. Toljan K, Vrooman B: **Low-dose naltrexone (LDN)—review of therapeutic utilization**. *Medical Sciences* 2018, **6**(4):82.

81. Liubchenko K, Kordbacheh K, Khajehdehi N, Visnjevac T, Ma F, Khan JS, Storey M, Abd-Elsayed A, Visnjevac O: **Naltrexone’s impact on cancer progression and mortality: a systematic review of studies in humans, animal models, and cell cultures**. *Advances in Therapy* 2021, **38**(2):904-924.

82. Peters KB, Affronti ML, Woodring S, Lipp E, Healy P, Herndon JE, 2nd, Miller ES, Freeman MW, Randazzo DM, Desjardins A *et al*: **Effects of low-dose naltrexone on quality of life in high-grade glioma patients: a placebo-controlled, double-blind randomized trial**. *Support Care Cancer* 2022, **30**(4):3463-3471.

83. AlRabiah H, Ahad A, Mostafa GAE, Al-Jenoobi FI: **Effect of Naltrexone Hydrochloride on Cytochrome P450 1A2, 2C9, 2D6, and 3A4 Activity in Human Liver Microsomes**. *Eur J Drug Metab Pharmacokinet* 2018, **43**(6):707-713.

84. Sevarino KA, Kosten TR: **Naltrexone for initiation and maintenance of opiate abstinence**. *Opiate Receptors Antagonists* 2009:227-245.

85. Brewer GJ: **Copper lowering therapy with tetrathiomolybdate as an antiangiogenic strategy in cancer**. *Curr Cancer Drug Targets* 2005, **5**(3):195-202.

86. Brewer GJ, Dick RD, Grover DK, LeClaire V, Tseng M, Wicha M, Pienta K, Redman BG, Jahan T, Sondak VK *et al*: **Treatment of metastatic cancer with tetrathiomolybdate, an anticopper, antiangiogenic agent: Phase I study**. *Clin Cancer Res* 2000, **6**(1):1-10.

87. Liu YL, Bager CL, Willumsen N, Ramchandani D, Kornhauser N, Ling L, Cobham M, Andreopoulou E, Cigler T, Moore A *et al*: **Tetrathiomolybdate (TM)-associated copper depletion influences collagen remodeling and immune response in the pre-metastatic niche of breast cancer**. *NPJ Breast Cancer* 2021, **7**(1):108.

88. Chan N, Willis A, Kornhauser N, Ward MM, Lee SB, Nackos E, Seo BR, Chuang E, Cigler T, Moore A *et al*: **Influencing the Tumor Microenvironment: A Phase II Study of Copper Depletion Using Tetrathiomolybdate in Patients with Breast Cancer at High Risk for Recurrence and in Preclinical Models of Lung Metastases**. *Clin Cancer Res* 2017, **23**(3):666-676.

89. Weiss KH, Czlonkowska A, Hedera P, Ferenci P: **WTX101 - an investigational drug for the treatment of Wilson disease**. *Expert opinion on investigational drugs* 2018, **27**(6):561-567.

90. Pino MA, Azer SA: **Cimetidine**. In*.*: StatPearls; 2022.

91. Furuta T, Sabit H, Dong Y, Miyashita K, Kinoshita M, Uchiyama N, Hayashi Y, Hayashi Y, Minamoto T, Nakada M: **Biological basis and clinical study of glycogen synthase kinase- 3beta-targeted therapy by drug repositioning for glioblastoma**. *Oncotarget* 2017, **8**(14):22811-22824.

92. Levine M, Bellward GD: **Effect of cimetidine on hepatic cytochrome P450: evidence for formation of a metabolite-intermediate complex**. *Drug metabolism and disposition: the biological fate of chemicals* 1995, **23**(12):1407-1411.

93. Lefranc F, Yeaton P, Brotchi J, Kiss R: **Cimetidine, an unexpected anti-tumor agent, and its potential for the treatment of glioblastoma (review)**. *Int J Oncol* 2006, **28**(5):1021-1030.

94. Pantziarka P, Bouche G, Meheus L, Sukhatme V, Sukhatme VP: **Repurposing drugs in oncology (ReDO)-cimetidine as an anti-cancer agent**. *Ecancermedicalscience* 2014, **8**:485.

95. Singh HK, Saadabadi A: **Sertraline**. In: *StatPearls [Internet].* edn.: StatPearls Publishing; 2022.

96. Bau-Carneiro JL, Akemi Guirao Sumida I, Gallon M, Zaleski T, Boia-Ferreira M, Bridi Cavassin F: **Sertraline repositioning: an overview of its potential use as a chemotherapeutic agent after four decades of tumor reversal studies**. *Translational oncology* 2022, **16**:101303.

97. Murdoch D, McTavish D: **Sertraline. A review of its pharmacodynamic and pharmacokinetic properties, and therapeutic potential in depression and obsessive-compulsive disorder**. *Drugs* 1992, **44**(4):604-624.

98. Atmaca A, Al-Batran SE, Maurer A, Neumann A, Heinzel T, Hentsch B, Schwarz SE, Hovelmann S, Gottlicher M, Knuth A *et al*: **Valproic acid (VPA) in patients with refractory advanced cancer: a dose escalating phase I clinical trial**. *Br J Cancer* 2007, **97**(2):177-182.

99. Rahman M, Nguyen H: **Valproic Acid**. In: *StatPearls [Internet].* edn.: StatPearls Publishing; 2021.

100. Rettie AE, Sheffels PR, Korzekwa KR, Gonzalez FJ, Philpot RM, Baillie TA: **CYP4 isoenzyme specificity and the relationship between. Omega.-hydroxylation and terminal desaturation of valproic acid**. *Biochemistry* 1995, **34**(24):7889-7895.

101. Han W, Guan W: **Valproic Acid: A Promising Therapeutic Agent in Glioma Treatment**. *Front Oncol* 2021, **11**:687362.

102. Sizar O, Khare S, Jamil RT, Talati R: **Statin Medications**. In*.*: StatPearls 2022.

103. Pisanti S, Picardi P, Ciaglia E, D'Alessandro A, Bifulco M: **Novel prospects of statins as therapeutic agents in cancer**. *Pharmacol Res* 2014, **88**:84-98.

104. Stragliotto G, Rahbar A, Solberg NW, Lilja A, Taher C, Orrego A, Bjurman B, Tammik C, Skarman P, Peredo I: **Effects of valganciclovir as an add‐on therapy in patients with cytomegalovirus‐positive glioblastoma: a randomized, double‐blind, hypothesis‐generating study**. *International journal of cancer* 2013, **133**(5):1204-1213.

105. Peng C, Wang J, Tanksley JP, Mobley BC, Ayers GD, Moots PL, Clark SW: **Valganciclovir and bevacizumab for recurrent glioblastoma: A single-institution experience**. *Mol Clin Oncol* 2016, **4**(2):154-158.

106. Stragliotto G, Pantalone MR, Rahbar A, Soderberg-Naucler C: **Valganciclovir as Add-On to Standard Therapy in Secondary Glioblastoma**. *Microorganisms* 2020, **8**(10):1471.

107. Al-Badr AA, Ajarim TDS: **Ganciclovir**. *Profiles Drug Subst Excip Relat Methodol* 2018, **43**:1-208.

108. Pantalone MR, Rahbar A, Soderberg-Naucler C, Stragliotto G: **Valganciclovir as Add-on to Second-Line Therapy in Patients with Recurrent Glioblastoma**. *Cancers (Basel)* 2022, **14**(8):1958.

109. Kumar A, Maini K, Kadian R: **Levetiracetam**. In: *StatPearls [Internet].* edn.: StatPearls Publishing; 2022.

110. Jabbarli R, Ahmadipour Y, Rauschenbach L, Santos AN, Darkwah Oppong M, Pierscianek D, Quesada CM, Kebir S, Dammann P, Guberina N: **How about levetiracetam in glioblastoma? An institutional experience and meta-analysis**. *Cancers (Basel)* 2021, **13**(15):3770.

111. King-Stephens D: **Effect of Levetiracetam Use Duration on Overall Survival of Isocitrate Dehydrogenase Wild-Type Glioblastoma in Adults**. *Epilepsy currents / American Epilepsy Society* 2022, **22**(5):288-290.

112. Patsalos PN: **Clinical pharmacokinetics of levetiracetam**. *Clin Pharmacokinet* 2004, **43**(11):707-724.

113. Kim YH, Kim T, Joo JD, Han JH, Kim YJ, Kim IA, Yun CH, Kim CY: **Survival benefit of levetiracetam in patients treated with concomitant chemoradiotherapy and adjuvant chemotherapy with temozolomide for glioblastoma multiforme**. *Cancer* 2015, **121**(17):2926-2932.

114. Phillips JT, Agrella S, Fox RJ: **Dimethyl Fumarate: A Review of Efficacy and Practical Management Strategies for Common Adverse Events in Patients with Multiple Sclerosis**. *Int J MS Care* 2017, **19**(2):74-83.

115. Shafer D, Tombes MB, Shrader E, Ryan A, Bandyopadhyay D, Dent P, Malkin M: **Phase I trial of dimethyl fumarate, temozolomide, and radiation therapy in glioblastoma**. *Neurooncol Adv* 2020, **2**(1):vdz052.

116. Lategan TW, Wang L, Sprague TN, Rousseau FS: **Pharmacokinetics and Bioavailability of Monomethyl Fumarate Following a Single Oral Dose of Bafiertam™(Monomethyl Fumarate) or Tecfidera®(Dimethyl Fumarate)**. *CNS drugs* 2021, **35**(5):567-574.

117. Heuer MA, Pietrusko RG, Morris RW, Scheffler BJ: **An analysis of worldwide safety experience with auranofin**. *J Rheumatol* 1985, **12**(4):695-699.

118. Madeira JM, Gibson DL, Kean WF, Klegeris A: **The biological activity of auranofin: implications for novel treatment of diseases**. *Inflammopharmacology* 2012, **20**(6):297-306.

119. Gamberi T, Chiappetta G, Fiaschi T, Modesti A, Sorbi F, Magherini F: **Upgrade of an old drug: Auranofin in innovative cancer therapies to overcome drug resistance and to increase drug effectiveness**. *Med Res Rev* 2022, **42**(3):1111-1146.

120. Freeman MZ, Cannizzaro DN, Naughton LF, Bove C: **Fluoroquinolones-Associated disability: it is not all in your head**. *NeuroSci* 2021, **2**(3):235-253.

121. Kast RE, Skuli N, Sardi I, Capanni F, Hessling M, Frosina G, Kast AP, Karpel-Massler G, Halatsch ME: **Augmentation of 5-Aminolevulinic Acid Treatment of Glioblastoma by Adding Ciprofloxacin, Deferiprone, 5-Fluorouracil and Febuxostat: The CAALA Regimen**. *Brain Sci* 2018, **8**(12):203.

122. Yadav V, Talwar P: **Repositioning of fluoroquinolones from antibiotic to anti-cancer agents: An underestimated truth**. *Biomed Pharmacother* 2019, **111**:934-946.

123. McLellan RA, Drobitch RK, Monshouwer M, Renton KW: **Fluoroquinolone antibiotics inhibit cytochrome P450-mediated microsomal drug metabolism in rat and human**. *Drug metabolism and disposition: the biological fate of chemicals* 1996, **24**(10):1134-1138.

124. Zhang L, Wei MJ, Zhao CY, Qi HM: **Determination of the inhibitory potential of 6 fluoroquinolones on CYP1A2 and CYP2C9 in human liver microsomes**. *Acta Pharmacol Sin* 2008, **29**(12):1507-1514.

125. Zandi A, Zanjani TM, Ziai SA, Poul YK, Hoseini MHM: **The Synergistic Effects of the Combination of Ciprofloxacin and Temozolomide on Human Glioblastoma A-172 Cell Line**. *Middle East Journal of Cancer* 2017, **8**(1):31-38.

126. Thai T, Salisbury BH, Zito PM: **Ciprofloxacin**. In: *StatPearls [Internet].* edn.: StatPearls Publishing; 2021.

127. Marte F, Sankar P, Patel P, Cassagnol M: **Captopril**. In: *StatPearls.* edn. Treasure Island (FL): StatPearls Publishing; 2024.

128. Flockhart DA, Tanus-Santos JE: **Implications of cytochrome P450 interactions when prescribing medication for hypertension**. *Archives of internal medicine* 2002, **162**(4):405-412.

129. Mulla S, Siddiqui WJ: **Losartan. NIH National Library of Medicine**. *National Center for Biotechnology Information, StatPearls* 2022.

130. Ursu R, Thomas L, Psimaras D, Chinot O, Le Rhun E, Ricard D, Charissoux M, Cuzzubbo S, Sejalon F, Quillien V *et al*: **Angiotensin II receptor blockers, steroids and radiotherapy in glioblastoma-a randomised multicentre trial (ASTER trial). An ANOCEF study**. *Eur J Cancer* 2019, **109**:129-136.

131. Sica DA, Gehr TW, Ghosh S: **Clinical pharmacokinetics of losartan**. *Clin Pharmacokinet* 2005, **44**(8):797-814.

132. Meadowcroft AM, Williamson KM, Patterson JH, Hinderliter AL, Pieper JA: **The effects of fluvastatin, a CYP2C9 inhibitor, on losartan pharmacokinetics in healthy volunteers**. *Journal of clinical pharmacology* 1999, **39**(4):418-424.

133. Boucher Y, Posada JM, Subudhi S, Kumar AS, Rosario SR, Gu L, Kumra H, Mino-Kenudson M, Talele NP, Duda DG *et al*: **Addition of Losartan to FOLFIRINOX and Chemoradiation Reduces Immunosuppression-Associated Genes, Tregs, and FOXP3+ Cancer Cells in Locally Advanced Pancreatic Cancer**. *Clin Cancer Res* 2023, **29**(8):1605-1619.

134. Singh S, Preuss CV: **Carvedilol**. In*.*: StatPearls; 2023.

135. Tewarie IA, Senders JT, Hulsbergen AFC, Kremer S, Broekman MLD: **Beta-blockers and glioma: a systematic review of preclinical studies and clinical results**. *Neurosurg Rev* 2021, **44**(2):669-677.

136. Sohel AJ, Shutter MC, Molla M: **Fluoxetine**. 2017.

137. Bruggeman C, O'Day CS: **Selective serotonin reuptake inhibitor toxicity**. In: *StatPearls [Internet].* edn.: StatPearls Publishing; 2022.

138. Tan SK, Jermakowicz A, Mookhtiar AK, Nemeroff CB, Schurer SC, Ayad NG: **Drug Repositioning in Glioblastoma: A Pathway Perspective**. *Front Pharmacol* 2018, **9**:218.

139. Bi J, Khan A, Tang J, Armando AM, Wu S, Zhang W, Gimple RC, Reed A, Jing H, Koga T *et al*: **Targeting glioblastoma signaling and metabolism with a re-purposed brain-penetrant drug**. *Cell Rep* 2021, **37**(5):109957.

140. Sallee FR, Pollock BG: **Clinical pharmacokinetics of imipramine and desipramine**. *Clin Pharmacokinet* 1990, **18**(5):346-364.

141. Masubuchi Y, Takahashii C, Fujio N, Horie T, Suzuki T, Imaoka S, Funae Y, Narimatsu S: **Inhibition and induction of cytochrome P450 isozymes after repetitive administration of imipramine in rats**. *Drug metabolism and disposition: the biological fate of chemicals* 1995, **23**(9):999-1003.

142. Ritchie MK, Kohli A: **Aprepitant**. 2019.

143. Sanchez RI, Wang RW, Newton DJ, Bakhtiar R, Lu P, Chiu SH, Evans DC, Huskey SE: **Cytochrome P450 3A4 is the major enzyme involved in the metabolism of the substance P receptor antagonist aprepitant**. *Drug metabolism and disposition: the biological fate of chemicals* 2004, **32**(11):1287-1292.

144. Rezaei S, Assaran Darban R, Javid H, Hashemy SI: **The Therapeutic Potential of Aprepitant in Glioblastoma Cancer Cells through Redox Modification**. *Biomed Res Int* 2022, **2022**:8540403.

145. Choi J, Fenando A: **Sulfasalazine**. 2020.

146. Robe PA, Martin DH, Nguyen-Khac MT, Artesi M, Deprez M, Albert A, Vanbelle S, Califice S, Bredel M, Bours V: **Early termination of ISRCTN45828668, a phase 1/2 prospective, randomized study of sulfasalazine for the treatment of progressing malignant gliomas in adults**. *BMC Cancer* 2009, **9**(1):372.

147. Takeuchi S, Wada K, Nagatani K, Otani N, Osada H, Nawashiro H: **Sulfasalazine and temozolomide with radiation therapy for newly diagnosed glioblastoma**. *Neurol India* 2014, **62**(1):42-47.

148. Kusuhara H, Furuie H, Inano A, Sunagawa A, Yamada S, Wu C, Fukizawa S, Morimoto N, Ieiri I, Morishita M *et al*: **Pharmacokinetic interaction study of sulphasalazine in healthy subjects and the impact of curcumin as an in vivo inhibitor of BCRP**. *British journal of pharmacology* 2012, **166**(6):1793-1803.

149. Talha B, Dhamoon A: **Ritonavir**. *Stat Pearls Treasure Island: Stat Pearls Publishing* 2020.

150. Granfors MT, Wang JS, Kajosaari LI, Laitila J, Neuvonen PJ, Backman JT: **Differential inhibition of cytochrome P450 3A4, 3A5 and 3A7 by five human immunodeficiency virus (HIV) protease inhibitors in vitro**. *Basic Clin Pharmacol Toxicol* 2006, **98**(1):79-85.

151. Cruz Da Silva E, Mercier MC, Etienne-Selloum N, Dontenwill M, Choulier L: **A Systematic Review of Glioblastoma-Targeted Therapies in Phases II, III, IV Clinical Trials**. *Cancers (Basel)* 2021, **13**(8).

152. Wyeth L: **Rapamune (sirolimus) oral solution and tablets**. *Philadelphia, PA* 2011.

153. Byeon S, Kang MJ, Choi YJ, Kim YJ, Kim M, Yun J, Yi SY, Kim JY, Kim ST, Lee J: **Antitumor activity and safety of sirolimus for solid tumors with PIK3CA mutations: A multicenter, open-label, prospective single-arm study (KM 02-01, KCSG UN17-16)**. *Transl Cancer Res* 2020, **9**(5):3222-3230.

154. Padda IS, Goyal A: **Leflunomide**. In: *StatPearls [Internet].* edn.: StatPearls Publishing; 2022.

155. Rozman B: **Clinical pharmacokinetics of leflunomide**. *Clin Pharmacokinet* 2002, **41**(6):421-430.

156. Ma LL, Wu ZT, Wang L, Zhang XF, Wang J, Chen C, Ni X, Lin YF, Cao YY, Luan Y *et al*: **Inhibition of hepatic cytochrome P450 enzymes and sodium/bile acid cotransporter exacerbates leflunomide-induced hepatotoxicity**. *Acta Pharmacol Sin* 2016, **37**(3):415-424.

157. Zhang C, Chu M: **Leflunomide: A promising drug with good antitumor potential**. *Biochem Biophys Res Commun* 2018, **496**(2):726-730.

158. Padda IS, Tripp J: **Phosphodiesterase inhibitors**. 2020.

159. Rolan P, Hutchinson M, Johnson K: **Ibudilast: a review of its pharmacology, efficacy and safety in respiratory and neurological disease**. *Expert opinion on pharmacotherapy* 2009, **10**(17):2897-2904.

160. Smith BP, Babos M: **Sildenafil**. 2020.

161. Hyland R, Roe EG, Jones BC, Smith DA: **Identification of the cytochrome P450 enzymes involved in the N-demethylation of sildenafil**. *Br J Clin Pharmacol* 2001, **51**(3):239-248.

162. Haider M, Elsherbeny A, Pittala V, Fallica AN, Alghamdi MA, Greish K: **The Potential Role of Sildenafil in Cancer Management through EPR Augmentation**. *J Pers Med* 2021, **11**(6):585.

163. Naber KG, Niggemann H, Stein G, Stein G: **Review of the literature and individual patients' data meta-analysis on efficacy and tolerance of nitroxoline in the treatment of uncomplicated urinary tract infections**. *BMC Infect Dis* 2014, **14**:628.

164. Galsky MD, Sfakianos JP, Ye D-W, Song X, Hu H, Shore ND, Zhang X, Zhang M: **ANTICIPATE phase I: Oral APL-1202 in combination with tislelizumab as neoadjuvant therapy in patients with muscle-invasive bladder cancer (MIBC)**. In*.*: American Society of Clinical Oncology; 2023.

165. Zhang QI, Wang S, Yang D, Pan K, Li L, Yuan S: **Preclinical pharmacodynamic evaluation of antibiotic nitroxoline for anticancer drug repurposing**. *Oncol Lett* 2016, **11**(5):3265-3272.

166. Cho HR, Kumari N, Thakur N, Vu HT, Kim H, Choi SH: **Decreased APE-1 by Nitroxoline Enhances Therapeutic Effect in a Temozolomide-resistant Glioblastoma: Correlation with Diffusion Weighted Imaging**. *Sci Rep* 2019, **9**(1):16613.

167. Lazovic J, Guo L, Nakashima J, Mirsadraei L, Yong W, Kim HJ, Ellingson B, Wu H, Pope WB: **Nitroxoline induces apoptosis and slows glioma growth in vivo**. *Neuro Oncol* 2015, **17**(1):53-62.

168. Martins AC, Paoliello MMB, Docea AO, Santamaria A, Tinkov AA, Skalny AV, Aschner M: **Review of the mechanism underlying mefloquine-induced neurotoxicity**. *Crit Rev Toxicol* 2021, **51**(3):209-216.

169. Fontaine F, de Sousa G, Burcham PC, Duchene P, Rahmani R: **Role of cytochrome P450 3A in the metabolism of mefloquine in human and animal hepatocytes**. *Life Sci* 2000, **66**(22):2193-2212.

170. Khaliq Y, Gallicano K, Tisdale C, Carignan G, Cooper C, McCarthy A: **Pharmacokinetic interaction between mefloquine and ritonavir in healthy volunteers**. *Br J Clin Pharmacol* 2001, **51**(6):591-600.

171. Mereddy G, Ronayne C: **Repurposing antimalarial drug mefloquine for cancer Treatment**. *Transl Med* 2018, **8**(199):2161-1025.1000199.

172. Perez DR, Sklar LA, Chigaev A: **Clioquinol: To harm or heal**. *Pharmacol Ther* 2019, **199**:155-163.

173. Wehbe M, Malhotra AK, Anantha M, Lo C, Dragowska WH, Dos Santos N, Bally MB: **Development of a copper-clioquinol formulation suitable for intravenous use**. *Drug Deliv Transl Res* 2018, **8**(1):239-251.

174. Schimmer AD, Jitkova Y, Gronda M, Wang Z, Brandwein J, Chen C, Gupta V, Schuh A, Yee K, Chen J *et al*: **A phase I study of the metal ionophore clioquinol in patients with advanced hematologic malignancies**. *Clin Lymphoma Myeloma Leuk* 2012, **12**(5):330-336.

175. Khan R, Khan H, Abdullah Y, Dou QP: **Feasibility of Repurposing Clioquinol for Cancer Therapy**. *Recent Pat Anticancer Drug Discov* 2020, **15**(1):14-31.

176. Navarro M, Camprubi D, Requena-Mendez A, Buonfrate D, Giorli G, Kamgno J, Gardon J, Boussinesq M, Munoz J, Krolewiecki A: **Safety of high-dose ivermectin: a systematic review and meta-analysis**. *J Antimicrob Chemother* 2020, **75**(4):827-834.

177. Juarez M, Schcolnik-Cabrera A, Duenas-Gonzalez A: **The multitargeted drug ivermectin: from an antiparasitic agent to a repositioned cancer drug**. *Am J Cancer Res* 2018, **8**(2):317-331.

178. Rendic SP: **Metabolism and interactions of Ivermectin with human cytochrome P450 enzymes and drug transporters, possible adverse and toxic effects**. *Archives of toxicology* 2021, **95**(5):1535-1546.

179. Tang M, Hu X, Wang Y, Yao X, Zhang W, Yu C, Cheng F, Li J, Fang Q: **Ivermectin, a potential anticancer drug derived from an antiparasitic drug**. *Pharmacol Res* 2021, **163**:105207.

180. Farzam K, Abdullah M: **Acetazolamide**. In: *StatPearls [Internet].* edn.: StatPearls Publishing; 2022.

181. Amiri A, Le PU, Moquin A, Machkalyan G, Petrecca K, Gillard JW, Yoganathan N, Maysinger D: **Inhibition of carbonic anhydrase IX in glioblastoma multiforme**. *Eur J Pharm Biopharm* 2016, **109**:81-92.

182. Pile HD, Sadiq NM: **Isotretinoin**. In*.*: StatPearls; 2022.

183. Yung WK, Kyritsis AP, Gleason MJ, Levin VA: **Treatment of recurrent malignant gliomas with high-dose 13-cis-retinoic acid**. *Clin Cancer Res* 1996, **2**(12):1931-1935.

184. Sonawane P, Cho HE, Tagde A, Verlekar D, Yu AL, Reynolds CP, Kang MH: **Metabolic characteristics of 13-cis-retinoic acid (isotretinoin) and anti-tumour activity of the 13-cis-retinoic acid metabolite 4-oxo-13-cis-retinoic acid in neuroblastoma**. *British journal of pharmacology* 2014, **171**(23):5330-5344.

185. Grogan DP, Winston NR: **Thalidomide**. *StatPearls* 2023.

186. Kesari S, Schiff D, Henson JW, Muzikansky A, Gigas DC, Doherty L, Batchelor TT, Longtine JA, Ligon KL, Weaver SJN-o: **Phase II study of temozolomide, thalidomide, and celecoxib for newly diagnosed glioblastoma in adults**. 2008, **10**(3):300-308.

187. Puduvalli VK, Giglio P, Groves MD, Hess KR, Gilbert MR, Mahankali S, Jackson EF, Levin VA, Conrad CA, Hsu SH *et al*: **Phase II trial of irinotecan and thalidomide in adults with recurrent glioblastoma multiforme**. *Neuro Oncol* 2008, **10**(2):216-222.

188. Baumann F, Bjeljac M, Kollias SS, Baumert BG, Brandner S, Rousson V, Yonekawa Y, Bernays RL: **Combined thalidomide and temozolomide treatment in patients with glioblastoma multiforme**. *J Neurooncol* 2004, **67**(1-2):191-200.

189. Lepper ER, Smith NF, Cox MC, Scripture CD, Figg WD: **Thalidomide metabolism and hydrolysis: mechanisms and implications**. *Curr Drug Metab* 2006, **7**(6):677-685.

190. von Hagens C, Walter-Sack I, Goeckenjan M, Osburg J, Storch-Hagenlocher B, Sertel S, Elsässer M, Remppis BA, Edler L, Munzinger J: **Prospective open uncontrolled phase I study to define a well-tolerated dose of oral artesunate as add-on therapy in patients with metastatic breast cancer (ARTIC M33/2)**. *Breast cancer research treatment* 2017, **164**:359-369.

191. Kast RE, Karpel-Massler G, Halatsch M-EJO: **CUSP9* treatment protocol for recurrent glioblastoma: aprepitant, artesunate, auranofin, captopril, celecoxib, disulfiram, itraconazole, ritonavir, sertraline augmenting continuous low dose temozolomide**. 2014, **5**(18):8052.

192. von Hagens C, Walter-Sack I, Goeckenjan M, Storch-Hagenlocher B, Sertel S, Elsasser M, Remppis BA, Munzinger J, Edler L, Efferth T *et al*: **Long-term add-on therapy (compassionate use) with oral artesunate in patients with metastatic breast cancer after participating in a phase I study (ARTIC M33/2)**. *Phytomedicine* 2019, **54**:140-148.

193. Ilett KF, Ethell BT, Maggs JL, Davis TM, Batty KT, Burchell B, Binh TQ, Thu le TA, Hung NC, Pirmohamed M *et al*: **Glucuronidation of dihydroartemisinin in vivo and by human liver microsomes and expressed UDP-glucuronosyltransferases**. *Drug metabolism and disposition: the biological fate of chemicals* 2002, **30**(9):1005-1012.

194. Thomas K, Saadabadi A: **Olanzapine**. In*.*: StatPearls; 2023.

195. Urichuk L, Prior TI, Dursun S, Baker G: **Metabolism of atypical antipsychotics: involvement of cytochrome p450 enzymes and relevance for drug-drug interactions**. *Curr Drug Metab* 2008, **9**(5):410-418.

196. You F, Zhang C, Liu X, Ji D, Zhang T, Yu R, Gao S: **Drug repositioning: Using psychotropic drugs for the treatment of glioma**. *Cancer Lett* 2022, **527**:140-149.

197. Kim KH, Kerndt CC, Adnan G, Schaller DJ: **Nitroglycerin**. In: *StatPearls [Internet].* edn.: StatPearls Publishing; 2022.

198. Sukhatme V, Bouche G, Meheus L, Sukhatme VP, Pantziarka P: **Repurposing Drugs in Oncology (ReDO)-nitroglycerin as an anti-cancer agent**. *Ecancermedicalscience* 2015, **9**:568.

199. Kuns B, Rosani A, Patel P, Varghese D: **Memantine**. In: *StatPearls.* edn. Treasure Island (FL): StatPearls Publishing; 2024.

200. Chilukuri S, Burela N: **Memantine for Prevention of Brain Irradiation-Induced Cognitive Toxicity: A Tale of an Underappreciated and Underused Intervention**. *JCO Glob Oncol* 2020, **6**:1384-1388.

201. Micuda S, Mundlova L, Anzenbacherova E, Anzenbacher P, Chladek J, Fuksa L, Martinkova J: **Inhibitory effects of memantine on human cytochrome P450 activities: prediction of in vivo drug interactions**. *Eur J Clin Pharmacol* 2004, **60**(8):583-589.

202. Nevin RL: **Unexpectedly low rates of neuropsychiatric adverse effects associated with mefloquine repurposed for the treatment of glioblastoma**. *Cancer* 2019, **125**(8):1384-1385.

203. Maraka S, Groves MD, Mammoser AG, Melguizo‐Gavilanes I, Conrad CA, Tremont‐Lukats IW, Loghin ME, O’Brien BJ, Puduvalli VK, Sulman EP: **Phase 1 lead‐in to a phase 2 factorial study of temozolomide plus memantine, mefloquine, and metformin as postradiation adjuvant therapy for newly diagnosed glioblastoma**. *Cancer* 2019, **125**(3):424-433.

204. Krilov LR: **Safety issues related to the administration of ribavirin**. *Pediatr Infect Dis J* 2002, **21**(5):479-481.

205. Volpin F, Casaos J, Sesen J, Mangraviti A, Choi J, Gorelick N, Frikeche J, Lott T, Felder R, Scotland SJ *et al*: **Use of an anti-viral drug, Ribavirin, as an anti-glioblastoma therapeutic**. *Oncogene* 2017, **36**(21):3037-3047.

206. Glue P: **The clinical pharmacology of ribavirin**. In: *Seminars in liver disease: 1999*; 1999: 17-24.

207. Ochiai Y, Sumi K, Sano E, Yoshimura S, Yamamuro S, Ogino A, Ueda T, Suzuki Y, Nakayama T, Hara H *et al*: **Antitumor effects of ribavirin in combination with TMZ and IFN-beta in malignant glioma cells**. *Oncol Lett* 2020, **20**(5):178.

208. Chokhawala K, Stevens L: **Antipsychotic medications**. In: *StatPearls [Internet].* edn.: StatPearls Publishing; 2022.

209. Kidron A, Nguyen H: **Phenothiazine**. In: *StatPearls [Internet].* edn.: StatPearls Publishing; 2022.

210. Wojcikowski J, Boksa J, Daniel WA: **Main contribution of the cytochrome P450 isoenzyme 1A2 (CYP1A2) to N-demethylation and 5-sulfoxidation of the phenothiazine neuroleptic chlorpromazine in human liver--A comparison with other phenothiazines**. *Biochem Pharmacol* 2010, **80**(8):1252-1259.

211. Siragusa S, Bistas KG, Saadabadi A: **Fluphenazine**. 2017.

212. Tay JK, Tan CH, Chong SA, Tan EC: **Functional polymorphisms of the cytochrome P450 1A2 (CYP1A2) gene and prolonged QTc interval in schizophrenia**. *Prog Neuropsychopharmacol Biol Psychiatry* 2007, **31**(6):1297-1302.

213. Kast RE: **Adding perphenazine to increase effectiveness of standard glioblastoma chemoirradiation**. *J BUON* 2020, **25**(4):1676-1686.

214. Dosanjh A, Won CY: **Focus: Allergic Diseases and Type II Immunity: Amlexanox: A Novel Therapeutic for Atopic, Metabolic, and Inflammatory Disease**. *The Yale journal of biology medicine* 2020, **93**(5):759.

215. Oral EA, Reilly SM, Gomez AV, Meral R, Butz L, Ajluni N, Chenevert TL, Korytnaya E, Neidert AH, Hench R: **Inhibition of IKKɛ and TBK1 improves glucose control in a subset of patients with type 2 diabetes**. *Cell Metab* 2017, **26**(1):157-170. e157.

216. Gan X, Wilson MW, Beyett TS, Wen B, Sun D, Larsen SD, Tesmer JJG, Saltiel AR, Showalter HD: **Synthesis of deuterium-labelled amlexanox and its metabolic stability against mouse, rat, and human microsomes**. *J Labelled Comp Radiopharm* 2019, **62**(5):202-208.
