# Supplementary material for: Clinical research framework proposal for ketogenic metabolic therapy in glioblastoma
Source: BMC Med. 2024 Dec 5;22:578. doi: 10.1186/s12916-024-03775-4 (PMC11622503; doi:10.1186/s12916-024-03775-4)
Supplement: Supplementary file 7 — Supplementary Material 7. [file 12916_2024_3775_MOESM7_ESM.docx]

**Supplementary Table 4.** Over-the-counter nutraceuticals with emerging evidence for complementary cancer use that may be evaluated as adjuncts to KMT.

| **Compound** | **Comments and Possible Effects in Cancer** | **References** |
| --- | --- | --- |
| *Boswellia Serrata* (frankincense). | Plant from traditional Indian medicine. Contains anti-inflammatory compounds that inhibit NF-κB and may induce apoptosis and suppress proliferation in cancer cells. Preliminary evidence suggests efficacy in treating both tumor-associated cerebral edema and cerebral edema associated with radiotherapy. Representative clinical trials: NCT00243022, NCT04669119, NCT03149081, NCT04161833. | [1-5] |
| Omega-3 fatty acids (DHA/EPA). | Traditionally supplemented as anti-inflammatory and cardioprotective. May have indirect anti-tumoral effects, including suppression of tumorigenesis, angiogenesis, and metastasis. Representative clinical trials: NCT01869764, NCT01823991, NCT00815685. | [6-9] |
| Cannabidiol (CBD) and related compounds. | CBD is an anti-inflammatory agent. Shown to relieve pain and side effects of chemotherapy. CBG, a related compound, may have anti-tumoral effects, including inhibition of invasion. THC and analogues have also been studied, but CBD may be preferable due to lack of psychoactive effects. Representative clinical trials: NCT02255292, NCT00530764, NCT00674609, NCT01361607. | [10-13] |
| Resveratrol/pterostilbene. | Phytochemicals typically studied as antioxidants and chemosensitizers. May induce apoptosis and inhibit metastasis. Representative clinical trials: NCT02261844, NCT00433576, NCT00455416. | [14-16] |
| Ursolic acid. | Anti-inflammatory agent. Preliminary evidence suggests anti-tumoral activity, including pro-apoptotic effects and modulation of glutamine transport. Representative clinical trials: NCT04403568. | [17-20] |
| Nattokinase. | Enzyme extracted from Nattō, a traditional Japanese fermented food. Contrary to its name, nattokinase is a serine protease. Anti-inflammatory, antithrombotic, antiangiogenic, and antifibrinolytic effects. | [21-25] |
| Serratiopeptidase. | Protease produced by *Serratia marcescens*. Anti-inflammatory agent, hypothesized to assist in the wound-healing process. | [26, 27] |
| Polyphenols. | Large family of naturally occurring phytochemicals. Polyphenols with anti-cancer effects include honokiol and genistein, EGCG via inhibition of GDH, hesperidin via regulation of gluconeogenesis, and caffeic acid via inhibition of GLS. Anti-tumoral activity can occur through a variety of mechanisms, such as antioxidant or anti-inflammatory effects. Representative clinical trials: NCT01928485, NCT03994055, NCT01426620, NCT03751592. | [28-35] |
| Flavonoids. | Polyphenols found in plants. Several flavonoids (e.g., alpinumisoflavone, 4-O-methyl alpinumisoflavone) inhibit HIF-1α and may interact with HIF-1 target genes. Some compounds such as apigenin, kaempferol, myricetin and fisetin are being investigated for direct anti-tumoral effects and secondary cancer prevention. Representative clinical trials: NCT00609310, NCT05968677, NCT05178303, NCT05903456. | [36-38] |
| Quercetin. | One of the most abundant dietary flavonoids. May synergize with temozolomide. Preliminary evidence shows direct pro-apoptotic effects, partly by induction of autophagy. Representative clinical trials: NCT05680662, NCT05724329. | [39-41] |
| Berberine. | Antihyperglycemic, anti-inflammatory and antioxidant agent. May be pro-apoptotic in cancer cells and regulate cell cycle, autophagy, and metastasis. Representative clinical trials: NCT03486496, NCT03281096, NCT02226185. | [42-45] |
| Oral vitamin C (low plasma concentrations). | Antioxidant agent. Vitamin C supplementation has been associated with lower mortality in breast cancer patients. Chemoprotective effects. Note that oral vitamin C results in much lower intermittent plasma concentrations, possibly with antioxidant rather than prooxidant activity. High-dose oral supplementation could enhance pharmacokinetics between intravenous infusions by exceeding the baseline homeostatic saturation levels. Representative clinical trials: NCT02868151, NCT03682029. | [46-50] |
| Sodium selenite. | Selenium is a nutritionally essential element; however, anti-cancer activity has been examined primarily for sodium selenite, via prooxidative mechanisms. Evidence from pilot clinical studies suggests that sodium selenite could reduce tumor burden, potentiate chemotherapy, and reduce chemotherapy toxicity. May decrease proliferation and induce autophagy in glioma cells. Representative clinical trial: NCT02184533. | [51-54] |
| Gingerols. | Antioxidant, antiproliferative and anti-inflammatory through a variety of signaling pathways. A clinical trial noted reduction in chemotherapy-induced nausea. Representative clinical trials: NCT00040742, NCT01887314, NCT00064272. | [55-58] |
| Alpha-lipoic acid (ALA). | Endogenously produced compound that is necessary for mitochondrial reactions. Antioxidant and anti-inflammatory via inhibition of NF-κB. May inhibit invasion and metastasis. May potentiate chemotherapy. Reported to reduce chemotherapy-induced neuropathy and cardiotoxicity. Representative clinical trials: NCT01313117, NCT00477607, NCT03908528, NCT05023863. | [59-61] |
| Curcumin. | Anti-inflammatory polyphenol, primary active ingredient in turmeric. Delivery to the brain is generally low due to poor blood-brain barrier permeability but can be enhanced by nanoparticle delivery. A clinical trial found improvements in muscle mass in patients with cachexia. May reduce side effects of chemoradiotherapy. Representative clinical trials: NCT03211104, NCT04208334, NCT01917890, NCT01042938. | [62-65] |
| Sulforaphane. | Compound found in cruciferous vegetables. In preclinical studies, shown to induce cell cycle arrest and apoptosis, inhibit cancer stem cells via modulation of NF-κB, PI3K/Akt signaling and other mechanisms. Ongoing clinical research focuses on both cancer prevention and intervention. Representative clinical trials: NCT02970682, NCT00894712, NCT00982319, NCT00982319, NCT01879878. | [66-68] |
| Hydroxycitric acid (from *Garcinia cambogia*). | May potentiate chemotherapy. Anti-cancer effects likely mediated by inhibition of ATP citrate lyase. One pilot study evaluated a combination of α-lipoic acid and hydroxycitrate together with a ketogenic diet, radiation, and chemotherapy in glioblastoma. | [69-72] |
| Thymoquinone. | Bioactive compound from *Nigella sativa*. May induce apoptosis, inhibit NF-κB, and metastasis, through a variety of signaling pathways. Novel formulations with enhanced bioavailability are being developed. | [73-75] |
| *Withania Somnifera* (Ashwagandha). | Traditional use as an adaptogen. Withanone appears to be the primary active ingredient with anti-cancer effects. Note that formulations can vary widely in composition (e.g., leaf extracts vs root extracts). Representative clinical trials: NCT04092647, NCT00689195, NCT05610735. | [76-78] |
| Mushroom extracts. | Mushroom extracts include *Agaricus blazei*, *Ganoderma lucidum*, *Agaricus bisporus*, and *Lentinula edodes*. Effects may be partially mediated by immunostimulatory polysaccharides, especially β-glycans, which also act as antioxidants. Representative clinical trials: NCT02603016, NCT04519879, NCT00779168, NCT00970021. | [79-83] |
| Garlic extracts. | Beyond traditional antithrombotic, antiarthritic, hypoglycemic, and hypolipidemic uses, anti-cancer effects may be mediated by a variety of bioactive sulfur and organic compounds, as well as allicin and its metabolites. Representative clinical trials: NCT00079170, NCT00455416. | [84] |
| N-acetylcysteine (NAC). | NAC acts as an antioxidant both directly by scavenging ROS and indirectly via production of glutathione. Note that NAC supplementation is typically recommended outside prooxidative strategies such as chemoradiotherapy. Representative clinical trials: NCT02094625, NCT03492047, NCT05539053, NCT05611086. | [85-88] |
| *Echinacea.* | Contains compounds with anti-inflammatory and antioxidant properties. May be effective in managing chemoradiotherapy-induced oral mucositis. Immunomodulatory effects. Representative clinical trials: NCT01674374, NCT03959410. | [89, 90] |
| *Polypodium leucotomos.* | Anti-inflammatory and antioxidant agent that is usually used for its photoprotective effects. Preclinical studies have focused primarily on prevention of photocarcinogenesis and immunomodulation. | [91-93] |
| Vitamin D. | Vitamin D deficiency is common among cancer patients. Higher plasma vitamin D levels have been associated with improved survival in colorectal cancer. Anti-cancer benefits from vitamin D supplementation may require daily dosing, as some clinical trials did not find benefit with intermittent schedules. Representative clinical trials: NCT01516216, NCT03472833, NCT04442412, NCT04094688. | [94-97] |
| High-dose vitamin E (e.g., tocotrienols). | Antioxidant agents. Reduction of side effects from chemoradiotherapy. Possible anti-angiogenic effects. Representative clinical trials: NCT00311116, NCT00583700, NCT02397486, NCT02399592. | [98-101] |
| *Rhodiola Rosea*. | Adaptogen and anti-inflammatory from traditional Tibetan medicine. May exert anti-tumoral effects via regulation of immunocytes, inhibition of angiogenesis, and promotion of apoptosis. | [102, 103] |
| *Silybum marianum* (milk thistle). | Typically used for liver protection and prevention of chemotherapy-induced toxicity. Anti-tumoral effects may be mediated by silybin, which acts as a GLUT inhibitor. Representative clinical trials: NCT00487721, NCT01129570, NCT01829178, NCT00055718. | [104-107] |
| *Astragalus membranaceus.* | Traditional use to support renal function. Astragalus polysaccharides have shown promise in improving cancer-related fatigue and are approved for this indication in some countries. *Astragalus membranaceus* extracts are being studied for chemosensitization, chemoprotection and immunomodulation. Representative clinical trials: NCT01720550, NCT03314805, NCT01802021. | [108-112] |
| Se-methylselenocysteine. | Pre-treatment with se-methylselenocysteine has been shown to potentiate chemotherapy while reducing various organ-specific toxicities and may allow for higher dosing of chemotherapeutic agents. May induce cell cycle arrest and apoptosis, likely via conversion to methylselenol. Representative clinical trials: NCT00829205. | [113-115] |
| Inositol phosphates. | A combination of inositol and inositol hexaphosphate (IP6) has been studied for prevention of chemotherapy-related toxicity. Myo-inositol trispyrophosphate (ITPP) acts as an anti-hypoxic and anti-angiogenic agent and may potentiate chemotherapy. Representative clinical trials: NCT02528526, NCT05410314. | [116, 117] |
| Prebiotics and probiotics. | Supplementation aims to maintain normal gut microbiome function or increase biodiversity. Reduction of chemoradiotherapy-induced toxicity to the intestinal mucosal barrier. The gut microbiome may play a role in the response to immune-based therapies, e.g., PD-1 blockade was less effective in patients pretreated with broad-spectrum antibiotics. Representative clinical trials: NCT05220124, NCT01473290, NCT03705442, NCT03642548. | [118-120] |
| High-dose melatonin. | Proposed to inhibit HIF-1α stabilization in tumor cells and protect normal cells from oxidative stress. Relatively high doses have been tested in preclinical models to achieve direct anti-tumoral activity. Most clinical trials focused on sleep disturbances and quality of life though chemoprotective effects. Representative clinical trials: NCT01355523, NCT02430298, NCT01706627, NCT02506777. | [121-124] |
| Methylene blue. | Approved clinical indication as a treatment for methemoglobinemia. Studied for neuroprotection by acting as an alternative electron carrier and redox modulator. However, its impact on mitochondrial SLP needs­­­ to be considered if targeting glutamine or TCA cycle metabolism. Representative clinical trials: NCT03469284, NCT05878405. | [125-128] |
| Oxaloacetate. | TCA cycle intermediate that may modulate intracellular redox balance and bioenergetic flux. Suggested to act by stabilization of mitochondrial metabolism and glutamine scavenging. Representative clinical trials: NCT04450160, NCT04290897. | [129-131] |

**Abbreviations:** ALA, Alpha-Lipoic Acid; CBG, Cannabigerol; CBD, Cannabidiol; DHA, Docosahexaenoic Acid; EPA, Eicosapentaenoic Acid; EGCG, Epigallocatechin Gallate; GDH, Glutamate Dehydrogenase; GLS, Glutaminase; GLUT, Glucose Transporter; HIF-1α, Hypoxia-Inducible Factor 1-alpha; IP6, Inositol Hexaphosphate; ITPP, Myo-Inositol Trispyrophosphate; NAC, N-Acetylcysteine; NF-κB, Nuclear Factor Kappa B; PD-1, Programmed Cell Death Protein 1; ROS, Reactive Oxygen Species; SLP, Substrate-Level Phosphorylation; THC, Tetrahydrocannabinol.

**References:**

1. Kirste S, Treier M, Wehrle SJ, Becker G, Abdel‐Tawab M, Gerbeth K, Hug MJ, Lubrich B, Grosu AL, Momm FJC: **Boswellia serrata acts on cerebral edema in patients irradiated for brain tumors: A prospective, randomized, placebo‐controlled, double‐blind pilot trial**. 2011, **117**(16):3788-3795.

2. Ammon HP: **Modulation of the immune system by Boswellia serrata extracts and boswellic acids**. *Phytomedicine* 2010, **17**(11):862-867.

3. Bonilla IV, Garcia D, Abbott A, Spruill L, Siegel J, Forcucci J, Hanna G, Mukherjee R, Hamann M, Hilliard E: **The Anti-Proliferative Effects of a Frankincense Extract in a Window of Opportunity Phase Ia Clinical Trial for Patients with Breast Cancer**. 2022.

4. Qayum A, Magotra A, Shah SM, Nandi U, Sharma PR, Shah BA, Singh SK: **Synergistic combination of PMBA and 5-fluorouracil (5-FU) in targeting mutant KRAS in 2D and 3D colorectal cancer cells**. *Heliyon* 2022, **8**(4):e09103.

5. Streffer JR, Bitzer M, Schabet M, Dichgans J, Weller M: **Response of radiochemotherapy-associated cerebral edema to a phytotherapeutic agent, H15**. *Neurology* 2001, **56**(9):1219-1221.

6. Montecillo-Aguado M, Tirado-Rodriguez B, Tong Z, Vega OM, Morales-Martinez M, Abkenari S, Pedraza-Chaverri J, Huerta-Yepez S: **Importance of the Role of omega-3 and omega-6 Polyunsaturated Fatty Acids in the Progression of Brain Cancer**. *Brain Sci* 2020, **10**(6):381.

7. Rose DP, Connolly JM, Coleman M: **Effect of omega-3 fatty acids on the progression of metastases after the surgical excision of human breast cancer cell solid tumors growing in nude mice**. *Clin Cancer Res* 1996, **2**(10):1751-1756.

8. Noguchi M, Minami M, Yagasaki R, Kinoshita K, Earashi M, Kitagawa H, Taniya T, Miyazaki I: **Chemoprevention of DMBA-induced mammary carcinogenesis in rats by low-dose EPA and DHA**. *Br J Cancer* 1997, **75**(3):348-353.

9. Cheng M, Zhang S, Ning C, Huo QJFin: **Omega-3 fatty acids supplementation improve nutritional status and inflammatory response in patients with lung cancer: a randomized clinical trial**. 2021, **8**:686752.

10. Lah TT, Novak M, Pena Almidon MA, Marinelli O, Zvar Baskovic B, Majc B, Mlinar M, Bosnjak R, Breznik B, Zomer R *et al*: **Cannabigerol Is a Potential Therapeutic Agent in a Novel Combined Therapy for Glioblastoma**. *Cells* 2021, **10**(2):340.

11. Seltzer ES, Watters AK, MacKenzie D, Jr., Granat LM, Zhang D: **Cannabidiol (CBD) as a Promising Anti-Cancer Drug**. *Cancers (Basel)* 2020, **12**(11):3203.

12. Kogan NM, Lavi Y, Topping LM, Williams RO, McCann FE, Yekhtin Z, Feldmann M, Gallily R, Mechoulam RJM: **Novel CBG derivatives can reduce inflammation, pain and obesity**. 2021, **26**(18):5601.

13. Heider CG, Itenberg SA, Rao J, Ma H, Wu XJB: **Mechanisms of cannabidiol (CBD) in cancer treatment: A review**. 2022, **11**(6):817.

14. Patra S, Pradhan B, Nayak R, Behera C, Rout L, Jena M, Efferth T, Bhutia SK: **Chemotherapeutic efficacy of curcumin and resveratrol against cancer: Chemoprevention, chemoprotection, drug synergism and clinical pharmacokinetics**. In: *Seminars in cancer biology: 2021*: Elsevier; 2021: 310-320.

15. Jiang Z, Chen K, Cheng L, Yan B, Qian W, Cao J, Li J, Wu E, Ma Q, Yang WJAotNYAoS: **Resveratrol and cancer treatment: Updates**. 2017, **1403**(1):59-69.

16. Salehi B, Mishra AP, Nigam M, Sener B, Kilic M, Sharifi-Rad M, Fokou PVT, Martins N, Sharifi-Rad J: **Resveratrol: A Double-Edged Sword in Health Benefits**. *Biomedicines* 2018, **6**(3):91.

17. Seo DY, Lee SR, Heo J-W, No M-H, Rhee BD, Ko KS, Kwak H-B, Han JJTKjop, Society pojotKP, Pharmacology tKSo: **Ursolic acid in health and disease**. 2018, **22**(3):235.

18. Wang S, Chang X, Zhang J, Li J, Wang N, Yang B, Pan B, Zheng Y, Wang X, Ou H *et al*: **Ursolic Acid Inhibits Breast Cancer Metastasis by Suppressing Glycolytic Metabolism via Activating SP1/Caveolin-1 Signaling**. *Front Oncol* 2021, **11**:745584.

19. Lodi A, Saha A, Lu X, Wang B, Sentandreu E, Collins M, Kolonin MG, DiGiovanni J, Tiziani SJNpo: **Combinatorial treatment with natural compounds in prostate cancer inhibits prostate tumor growth and leads to key modulations of cancer cell metabolism**. 2017, **1**(1):18.

20. Feng XM, Su XL: **Anticancer effect of ursolic acid via mitochondria-dependent pathways**. *Oncol Lett* 2019, **17**(6):4761-4767.

21. Weng Y, Yao J, Sparks S, Wang KYJIjoms: **Nattokinase: an oral antithrombotic agent for the prevention of cardiovascular disease**. 2017, **18**(3):523.

22. Jamali N, Vahedi F, Soltani Fard E, Taheri-Anganeh M, Taghvimi S, Khatami SH, Ghasemi H, Movahedpour A: **Nattokinase: Structure, applications and sources**. *Biocatalysis and Agricultural Biotechnology* 2023, **47**:102564.

23. Chen H, McGowan EM, Ren N, Lal S, Nassif N, Shad-Kaneez F, Qu X, Lin Y: **Nattokinase: A Promising Alternative in Prevention and Treatment of Cardiovascular Diseases**. *Biomark Insights* 2018, **13**:1177271918785130.

24. Zhang B, Chai J, He LM, Dusanbieke M, Gong AH: **Nattokinase produced by natto fermentation with Bacillus subtilis inhibits breast cancer growth**. *International Journal of Clinical and Experimental Medicine* 2019, **12**(12):13380-13387.

25. Yan Y, Wang Y, Qian J, Wu S, Ji Y, Liu Y, Zeng J, Gong A: **Nattokinase Crude Extract Inhibits Hepatocellular Carcinoma Growth in Mice**. *J Microbiol Biotechnol* 2019, **29**(8):1281-1287.

26. Jadhav SB, Shah N, Rathi A, Rathi V, Rathi AJBR: **Serratiopeptidase: Insights into the therapeutic applications**. 2020, **28**:e00544.

27. Nair SR, C SD: **Serratiopeptidase: An integrated View of Multifaceted Therapeutic Enzyme**. *Biomolecules* 2022, **12**(10):1468.

28. Banik K, Ranaware AM, Deshpande V, Nalawade SP, Padmavathi G, Bordoloi D, Sailo BL, Shanmugam MK, Fan L, Arfuso FJPr: **Honokiol for cancer therapeutics: A traditional medicine that can modulate multiple oncogenic targets**. 2019, **144**:192-209.

29. Ferrari E, Bettuzzi S, Naponelli VJIJoMS: **The potential of epigallocatechin gallate (EGCG) in targeting autophagy for cancer treatment: A narrative review**. 2022, **23**(11):6075.

30. Li C, Li M, Chen P, Narayan S, Matschinsky FM, Bennett MJ, Stanley CA, Smith TJJJoBC: **Green tea polyphenols control dysregulated glutamate dehydrogenase in transgenic mice by hijacking the ADP activation site**. 2011, **286**(39):34164-34174.

31. Aggarwal V, Tuli HS, Thakral F, Singhal P, Aggarwal D, Srivastava S, Pandey A, Sak K, Varol M, Khan MAJEB *et al*: **Molecular mechanisms of action of hesperidin in cancer: Recent trends and advancements**. 2020, **245**(5):486-497.

32. Zareei S, Boojar MMA, Amanlou M: **Inhibition of liver alanine aminotransferase and aspartate aminotransferase by hesperidin and its aglycone hesperetin: An in vitro and in silico study**. *Life Sci* 2017, **178**:49-55.

33. Alam M, Ahmed S, Elasbali AM, Adnan M, Alam S, Hassan MI, Pasupuleti VRJFiO: **Therapeutic implications of caffeic acid in cancer and neurological diseases**. 2022, **12**:860508.

34. Tyszka-Czochara M, Bukowska-Strakova K, Kocemba-Pilarczyk KA, Majka MJN: **Caffeic acid targets AMPK signaling and regulates tricarboxylic acid cycle anaplerosis while metformin downregulates HIF-1α-induced glycolytic enzymes in human cervical squamous cell carcinoma lines**. 2018, **10**(7):841.

35. Kopustinskiene DM, Jakstas V, Savickas A, Bernatoniene JJN: **Flavonoids as anticancer agents**. 2020, **12**(2):457.

36. Gao J-L, Chen Y-GJBri: **Natural compounds regulate glycolysis in hypoxic tumor microenvironment**. 2015, **2015**.

37. Abotaleb M, Samuel SM, Varghese E, Varghese S, Kubatka P, Liskova A, Busselberg D: **Flavonoids in Cancer and Apoptosis**. *Cancers (Basel)* 2018, **11**(1):28.

38. Farhan M, Rizvi A, Aatif M, Ahmad A: **Current Understanding of Flavonoids in Cancer Therapy and Prevention**. *Metabolites* 2023, **13**(4):481.

39. Rauf A, Imran M, Khan IA, ur‐Rehman M, Gilani SA, Mehmood Z, Mubarak MSJPR: **Anticancer potential of quercetin: A comprehensive review**. 2018, **32**(11):2109-2130.

40. Okamoto T: **Safety of quercetin for clinical application (Review)**. *International journal of molecular medicine* 2005, **16**(2):275-278.

41. Shala AL, Arduino I, Salihu MB, Denora N: **Quercetin and Its Nano-Formulations for Brain Tumor Therapy-Current Developments and Future Perspectives for Paediatric Studies**. *Pharmaceutics* 2023, **15**(3):963.

42. Tillhon M, Guaman Ortiz LM, Lombardi P, Scovassi AI: **Berberine: new perspectives for old remedies**. *Biochem Pharmacol* 2012, **84**(10):1260-1267.

43. Almatroodi SA, Alsahli MA, Rahmani AH: **Berberine: An Important Emphasis on Its Anticancer Effects through Modulation of Various Cell Signaling Pathways**. *Molecules* 2022, **27**(18):5889.

44. Rauf A, Abu-Izneid T, Khalil AA, Imran M, Shah ZA, Emran TB, Mitra S, Khan Z, Alhumaydhi FA, Aljohani ASM *et al*: **Berberine as a Potential Anticancer Agent: A Comprehensive Review**. *Molecules* 2021, **26**(23):7368.

45. Chen YX, Gao QY, Zou TH, Wang BM, Liu SD, Sheng JQ, Ren JL, Zou XP, Liu ZJ, Song YY *et al*: **Berberine versus placebo for the prevention of recurrence of colorectal adenoma: a multicentre, double-blinded, randomised controlled study**. *Lancet Gastroenterol Hepatol* 2020, **5**(3):267-275.

46. Padayatty SJ, Katz A, Wang Y, Eck P, Kwon O, Lee JH, Chen S, Corpe C, Dutta A, Dutta SK *et al*: **Vitamin C as an antioxidant: evaluation of its role in disease prevention**. *J Am Coll Nutr* 2003, **22**(1):18-35.

47. Harris HR, Orsini N, Wolk A: **Vitamin C and survival among women with breast cancer: a meta-analysis**. *Eur J Cancer* 2014, **50**(7):1223-1231.

48. Chaitanya N, Muthukrishnan A, Rao KP, Reshma D, Priyanka PU, Abhijeeth H, Kovur A, Kumar AN, Research: **Oral Mucositis Severity Assessment by Supplementation of High Dose Ascorbic Acid During Chemo and/or Radiotherapy of Oro-Pharyngeal Cancers--A Pilot Project**. *Indian Journal of Pharmaceutical Education* 2018, **52**(3):532-539.

49. Padayatty SJ, Sun H, Wang Y, Riordan HD, Hewitt SM, Katz A, Wesley RA, Levine MJAoim: **Vitamin C pharmacokinetics: implications for oral and intravenous use**. 2004, **140**(7):533-537.

50. Lykkesfeldt J, Tveden-Nyborg P: **The Pharmacokinetics of Vitamin C**. *Nutrients* 2019, **11**(10):2412.

51. Lipinski B: **Sodium Selenite as an Anticancer Agent**. *Anticancer Agents Med Chem* 2017, **17**(5):658-661.

52. Jayachandran P, Knox SJ, Garcia-Cremades M, Savić RMJDiR: **Clinical pharmacokinetics of oral sodium selenite and dosing implications in the treatment of patients with metastatic cancer**. 2021, **21**:169-178.

53. Brodin O, Eksborg S, Wallenberg M, Asker-Hagelberg C, Larsen EH, Mohlkert D, Lenneby-Helleday C, Jacobsson H, Linder S, Misra S *et al*: **Pharmacokinetics and Toxicity of Sodium Selenite in the Treatment of Patients with Carcinoma in a Phase I Clinical Trial: The SECAR Study**. *Nutrients* 2015, **7**(6):4978-4994.

54. Hazane-Puch F, Arnaud J, Trocme C, Faure P, Laporte F, Champelovier P: **Sodium Selenite Decreased HDAC Activity, Cell Proliferation and Induced Apoptosis in Three Human Glioblastoma Cells**. *Anticancer Agents Med Chem* 2016, **16**(4):490-500.

55. de Lima RMT, Dos Reis AC, de Menezes AAPM, Santos JVdO, Filho JWGdO, Ferreira JRdO, de Alencar MVOB, da Mata AMOF, Khan IN, Islam AJPr: **Protective and therapeutic potential of ginger (Zingiber officinale) extract and [6]‐gingerol in cancer: A comprehensive review**. 2018, **32**(10):1885-1907.

56. Lee DH, Kim DW, Jung CH, Lee YJ, Park D: **Gingerol sensitizes TRAIL-induced apoptotic cell death of glioblastoma cells**. *Toxicol Appl Pharmacol* 2014, **279**(3):253-265.

57. Ryan JL, Heckler CE, Roscoe JA, Dakhil SR, Kirshner J, Flynn PJ, Hickok JT, Morrow GR: **Ginger (Zingiber officinale) reduces acute chemotherapy-induced nausea: a URCC CCOP study of 576 patients**. *Support Care Cancer* 2012, **20**(7):1479-1489.

58. Rastogi N, Gara RK, Trivedi R, Singh A, Dixit P, Maurya R, Duggal S, Bhatt ML, Singh S, Mishra DP: **(6)-Gingerolinduced myeloid leukemia cell death is initiated by reactive oxygen species and activation of miR-27b expression**. *Free Radic Biol Med* 2014, **68**:288-301.

59. Salehi B, Berkay Yilmaz Y, Antika G, Boyunegmez Tumer T, Fawzi Mahomoodally M, Lobine D, Akram M, Riaz M, Capanoglu E, Sharopov F *et al*: **Insights on the Use of alpha-Lipoic Acid for Therapeutic Purposes**. *Biomolecules* 2019, **9**(8):356.

60. Dörsam B, Fahrer J: **The disulfide compound α-lipoic acid and its derivatives: a novel class of anticancer agents targeting mitochondria**. *Cancer Lett* 2016, **371**(1):12-19.

61. Werida RH, Elshafiey RA, Ghoneim A, Elzawawy S, Mostafa TM: **Role of alpha-lipoic acid in counteracting paclitaxel- and doxorubicin-induced toxicities: a randomized controlled trial in breast cancer patients**. *Support Care Cancer* 2022, **30**(9):7281-7292.

62. Klinger NV, Mittal S: **Therapeutic Potential of Curcumin for the Treatment of Brain Tumors**. *Oxid Med Cell Longev* 2016, **2016**:9324085.

63. Tajbakhsh A, Hasanzadeh M, Rezaee M, Khedri M, Khazaei M, ShahidSales S, Ferns GA, Hassanian SM, Avan A: **Therapeutic potential of novel formulated forms of curcumin in the treatment of breast cancer by the targeting of cellular and physiological dysregulated pathways**. *J Cell Physiol* 2018, **233**(3):2183-2192.

64. Ryan JL, Heckler CE, Ling M, Katz A, Williams JP, Pentland AP, Morrow GR: **Curcumin for radiation dermatitis: a randomized, double-blind, placebo-controlled clinical trial of thirty breast cancer patients**. *Radiat Res* 2013, **180**(1):34-43.

65. Thambamroong T, Seetalarom K, Saichaemchan S, Pumsutas Y, Prasongsook N: **Efficacy of Curcumin on Treating Cancer Anorexia-Cachexia Syndrome in Locally or Advanced Head and Neck Cancer: A Double-Blind, Placebo-Controlled Randomised Phase IIa Trial (CurChexia)**. *J Nutr Metab* 2022, **2022**:5425619.

66. Elkashty OA, Tran SDJCMS: **Sulforaphane as a promising natural molecule for cancer prevention and treatment**. 2021, **41**:250-269.

67. Clarke JD, Dashwood RH, Ho E: **Multi-targeted prevention of cancer by sulforaphane**. *Cancer Lett* 2008, **269**(2):291-304.

68. Kaiser AE, Baniasadi M, Giansiracusa D, Giansiracusa M, Garcia M, Fryda Z, Wong TL, Bishayee A: **Sulforaphane: A Broccoli Bioactive Phytocompound with Cancer Preventive Potential**. *Cancers (Basel)* 2021, **13**(19):4796.

69. Schwartz L, Seyfried T, Alfarouk KO, Moreira JDV, Fais S: **Out of Warburg effect: An effective cancer treatment targeting the tumor specific metabolism and dysregulated pH**. In: *Seminars in cancer biology: 2017*: Elsevier; 2017: 134-138.

70. Ismail A, Doghish AS, B EME, Salama SA, Mariee AD: **Hydroxycitric acid potentiates the cytotoxic effect of tamoxifen in MCF-7 breast cancer cells through inhibition of ATP citrate lyase**. *Steroids* 2020, **160**:108656.

71. Ismail A, Mokhlis HA, Sharaky M, Sobhy MH, Hassanein SS, Doghish AS, Salama SA, Mariee AD, Attia YM: **Hydroxycitric acid reverses tamoxifen resistance through inhibition of ATP citrate lyase**. *Pathol Res Pract* 2022, **240**:154211.

72. Verrelli D, Dallera L, Stendardo M, Monzani S, Pasqualato S, Giorgio M, Pallavi R: **Hydroxycitric Acid Inhibits Chronic Myelogenous Leukemia Growth through Activation of AMPK and mTOR Pathway**. *Nutrients* 2022, **14**(13):2669.

73. Imran M, Rauf A, Khan IA, Shahbaz M, Qaisrani TB, Fatmawati S, Abu-Izneid T, Imran A, Rahman KU, Gondal TA: **Thymoquinone: A novel strategy to combat cancer: A review**. *Biomed Pharmacother* 2018, **106**:390-402.

74. Almajali B, Al-Jamal HAN, Taib WRW, Ismail I, Johan MF, Doolaanea AA, Ibrahim WNJP: **Thymoquinone, as a novel therapeutic candidate of cancers**. 2021, **14**(4):369.

75. Alhmied F, Alammar A, Alsultan B, Alshehri M, Pottoo FH: **Molecular Mechanisms of Thymoquinone as Anticancer Agent**. *Comb Chem High Throughput Screen* 2021, **24**(10):1644-1653.

76. Palliyaguru DL, Singh SV, Kensler TWJMn, research f: **Withania somnifera: from prevention to treatment of cancer**. 2016, **60**(6):1342-1353.

77. Wadhwa R, Konar A, Kaul SC: **Nootropic potential of Ashwagandha leaves: Beyond traditional root extracts**. *Neurochem Int* 2016, **95**:109-118.

78. Widodo N, Kaur K, Shrestha BG, Takagi Y, Ishii T, Wadhwa R, Kaul SC: **Selective killing of cancer cells by leaf extract of Ashwagandha: identification of a tumor-inhibitory factor and the first molecular insights to its effect**. *Clin Cancer Res* 2007, **13**(7):2298-2306.

79. Park HJ: **Current Uses of Mushrooms in Cancer Treatment and Their Anticancer Mechanisms**. *Int J Mol Sci* 2022, **23**(18):10502.

80. Nagashima Y, Yoshino S, Yamamoto S, Maeda N, Azumi T, Komoike Y, Okuno K, Iwasa T, Tsurutani J, Nakagawa K *et al*: **Lentinula edodes mycelia extract plus adjuvant chemotherapy for breast cancer patients: Results of a randomized study on host quality of life and immune function improvement**. *Mol Clin Oncol* 2017, **7**(3):359-366.

81. Ahmad MF: **Ganoderma lucidum: A rational pharmacological approach to surmount cancer**. *J Ethnopharmacol* 2020, **260**:113047.

82. Hetland G, Johnson E, Lyberg T, Kvalheim G: **The Mushroom Agaricus blazei Murill Elicits Medicinal Effects on Tumor, Infection, Allergy, and Inflammation through Its Modulation of Innate Immunity and Amelioration of Th1/Th2 Imbalance and Inflammation**. *Adv Pharmacol Sci* 2011, **2011**:157015.

83. Twardowski P, Kanaya N, Frankel P, Synold T, Ruel C, Pal SK, Junqueira M, Prajapati M, Moore T, Tryon P: **A phase I trial of mushroom powder in patients with biochemically recurrent prostate cancer: Roles of cytokines and myeloid‐derived suppressor cells for Agaricus bisporus–induced prostate‐specific antigen responses**. *Cancer* 2015, **121**(17):2942-2950.

84. Mondal A, Banerjee S, Bose S, Mazumder S, Haber RA, Farzaei MH, Bishayee AJPr: **Garlic constituents for cancer prevention and therapy: From phytochemistry to novel formulations**. 2022, **175**:105837.

85. Šalamon Š, Kramar B, Marolt TP, Poljšak B, Milisav IJA: **Medical and dietary uses of N-acetylcysteine**. 2019, **8**(5):111.

86. Piskounova E, Agathocleous M, Murphy MM, Hu Z, Huddlestun SE, Zhao Z, Leitch AM, Johnson TM, DeBerardinis RJ, Morrison SJ: **Oxidative stress inhibits distant metastasis by human melanoma cells**. *Nature* 2015, **527**(7577):186-191.

87. Schwalfenberg GK: **N-Acetylcysteine: A Review of Clinical Usefulness (an Old Drug with New Tricks)**. *J Nutr Metab* 2021, **2021**:9949453.

88. Orgel E, Knight KR, Chi YY, Malvar J, Rushing T, Mena V, Eisenberg LS, Rassekh SR, Ross CJD, Scott EN *et al*: **Intravenous N-Acetylcysteine to Prevent Cisplatin-Induced Hearing Loss in Children: A Nonrandomized Controlled Phase I Trial**. *Clin Cancer Res* 2023, **29**(13):2410-2418.

89. Tafazoli AJCMR: **Echinacea for cancer patients: To give or not to give**. 2020, **27**(2):112-116.

90. Miller SC: **Can herbs be useful in cancer therapy? A review of studies on the influence of Echinacea on cells of the immune system and on tumor amelioration**. *Biomed Res* 2012, **23**:9-16.

91. Lacerda PA, Oenning LC, Bellato GC, Lopes-Santos L, Antunes NJ, Mariz B, Teixeira G, Vasconcelos R, Simoes GF, de Souza IA *et al*: **Polypodium leucotomos targets multiple aspects of oral carcinogenesis and it is a potential antitumor phytotherapy against tongue cancer growth**. *Front Pharmacol* 2022, **13**:1098374.

92. Parrado C, Mascaraque M, Gilaberte Y, Juarranz A, Gonzalez S: **Fernblock (Polypodium leucotomos Extract): Molecular Mechanisms and Pleiotropic Effects in Light-Related Skin Conditions, Photoaging and Skin Cancers, a Review**. *Int J Mol Sci* 2016, **17**(7):1026.

93. Calzari P, Vaienti S, Nazzaro G: **Uses of Polypodium leucotomos Extract in Oncodermatology**. *J Clin Med* 2023, **12**(2):673.

94. Maalmi H, Walter V, Jansen L, Boakye D, Schottker B, Hoffmeister M, Brenner H: **Association between Blood 25-Hydroxyvitamin D Levels and Survival in Colorectal Cancer Patients: An Updated Systematic Review and Meta-Analysis**. *Nutrients* 2018, **10**(7):896.

95. Ng K, Nimeiri HS, McCleary NJ, Abrams TA, Yurgelun MB, Cleary JM, Rubinson DA, Schrag D, Miksad R, Bullock AJJJ: **Effect of high-dose vs standard-dose vitamin D3 supplementation on progression-free survival among patients with advanced or metastatic colorectal cancer: the SUNSHINE randomized clinical trial**. 2019, **321**(14):1370-1379.

96. Scragg R, Khaw KT, Toop L, Sluyter J, Lawes CMM, Waayer D, Giovannucci E, Camargo CA, Jr.: **Monthly High-Dose Vitamin D Supplementation and Cancer Risk: A Post Hoc Analysis of the Vitamin D Assessment Randomized Clinical Trial**. *JAMA Oncol* 2018, **4**(11):e182178.

97. Keum N, Chen QY, Lee DH, Manson JE, Giovannucci E: **Vitamin D supplementation and total cancer incidence and mortality by daily vs. infrequent large-bolus dosing strategies: a meta-analysis of randomised controlled trials**. *Br J Cancer* 2022, **127**(5):872-878.

98. Kirkpatrick DL, Powis G: **Clinically Evaluated Cancer Drugs Inhibiting Redox Signaling**. *Antioxid Redox Signal* 2017, **26**(6):262-273.

99. Aggarwal V, Kashyap D, Sak K, Tuli HS, Jain A, Chaudhary A, Garg VK, Sethi G, Yerer MBJIjoms: **Molecular mechanisms of action of tocotrienols in cancer: Recent trends and advancements**. 2019, **20**(3):656.

100. Thomsen CB, Andersen RF, Steffensen KD, Adimi P, Jakobsen A: **Delta tocotrienol in recurrent ovarian cancer. A phase II trial**. *Pharmacol Res* 2019, **141**:392-396.

101. Sayed R, El Wakeel L, Saad AS, Kelany M, El-Hamamsy M: **Pentoxifylline and vitamin E reduce the severity of radiotherapy-induced oral mucositis and dysphagia in head and neck cancer patients: a randomized, controlled study**. *Medical Oncology* 2020, **37**(1):8.

102. Pu WL, Zhang MY, Bai RY, Sun LK, Li WH, Yu YL, Zhang Y, Song L, Wang ZX, Peng YF *et al*: **Anti-inflammatory effects of Rhodiola rosea L.: A review**. *Biomed Pharmacother* 2020, **121**:109552.

103. Zhang X, Zhu J, Yan J, Xiao Y, Yang R, Huang R, Zhou J, Wang Z, Xiao W, Zheng C *et al*: **Systems pharmacology unravels the synergic target space and therapeutic potential of Rhodiola rosea L. for non-small cell lung cancer**. *Phytomedicine* 2020, **79**:153326.

104. Wang Y, Yuan A-J, Wu Y-J, Wu L-M, Zhang L: **Silymarin in cancer therapy: Mechanisms of action, protective roles in chemotherapy-induced toxicity, and nanoformulations**. *Journal of Functional Foods* 2023, **100**:105384.

105. Koltai T, Fliegel L: **Role of Silymarin in Cancer Treatment: Facts, Hypotheses, and Questions**. *J Evid Based Integr Med* 2022, **27**:2515690X211068826.

106. Singh M, Kadhim MM, Turki Jalil A, Oudah SK, Aminov Z, Alsaikhan F, Jawhar ZH, Ramirez-Coronel AA, Farhood B: **A systematic review of the protective effects of silymarin/silibinin against doxorubicin-induced cardiotoxicity**. *Cancer Cell Int* 2023, **23**(1):88.

107. Catanzaro D, Gabbia D, Cocetta V, Biagi M, Ragazzi E, Montopoli M, Carrara M: **Silybin counteracts doxorubicin resistance by inhibiting GLUT1 expression**. *Fitoterapia* 2018, **124**:42-48.

108. Li S, Sun Y, Huang J, Wang B, Gong Y, Fang Y, Liu Y, Wang S, Guo Y, Wang HJJoe: **Anti-tumor effects and mechanisms of Astragalus membranaceus (AM) and its specific immunopotentiation: Status and prospect**. 2020, **258**:112797.

109. Rau KM, Shen W-C, Chen S-C, Wang C-H, Hsieh RK, Hung C-M, Peng M, Liu C-T, Chang Y, Kuo W-L: **Effect of astragalus polysaccharides (PG2) treatment of adjuvant chemotherapy-induced fatigue in premenopausal patients with breast cancer**. In*.*: American Society of Clinical Oncology; 2023.

110. Wang C-H, Lai Y-L, Lin C-Y, Chen J-S, Ho C-L, Rau K-M, Tsai J-T, Chang C-S, Yeh S-P: **PG2 injection, a novel botanical drug approved for improving cancer-related fatigue among advanced cancer patients under standard palliative care: A double blind, multi-center, randomized phase IV study**. In*.*: American Society of Clinical Oncology; 2018.

111. Bamodu OA, Kuo KT, Wang CH, Huang WC, Wu ATH, Tsai JT, Lee KY, Yeh CT, Wang LS: **Astragalus polysaccharides (PG2) Enhances the M1 Polarization of Macrophages, Functional Maturation of Dendritic Cells, and T Cell-Mediated Anticancer Immune Responses in Patients with Lung Cancer**. *Nutrients* 2019, **11**(10):2264.

112. Auyeung KK, Han QB, Ko JK: **Astragalus membranaceus: A Review of its Protection Against Inflammation and Gastrointestinal Cancers**. *Am J Chin Med* 2016, **44**(1):1-22.

113. Fernandes AP, Gandin V: **Selenium compounds as therapeutic agents in cancer**. *Biochimica et biophysica acta* 2015, **1850**(8):1642-1660.

114. Cao S, Durrani F, Toth K, Rustum YJBjoc: **Se-methylselenocysteine offers selective protection against toxicity and potentiates the antitumour activity of anticancer drugs in preclinical animal models**. 2014, **110**(7):1733-1743.

115. Spallholz JE, Palace VP, Reid TW: **Methioninase and selenomethionine but not Se-methylselenocysteine generate methylselenol and superoxide in an in vitro chemiluminescent assay: implications for the nutritional carcinostatic activity of selenoamino acids**. *Biochem Pharmacol* 2004, **67**(3):547-554.

116. Schneider MA, Linecker M, Fritsch R, Muehlematter UJ, Stocker D, Pestalozzi B, Samaras P, Jetter A, Kron P, Petrowsky H *et al*: **Phase Ib dose-escalation study of the hypoxia-modifier Myo-inositol trispyrophosphate in patients with hepatopancreatobiliary tumors**. *Nat Commun* 2021, **12**(1):3807.

117. Amabile MI, De Luca A, Tripodi D, D’Alberti E, Melcarne R, Imbimbo G, Picconi O, D’Andrea V, Vergine M, Sorrenti SJJoPM: **Effects of inositol hexaphosphate and myo-inositol administration in breast cancer patients during adjuvant chemotherapy**. 2021, **11**(8):756.

118. Lu K, Dong S, Wu X, Jin R, Chen HJFiO: **Probiotics in cancer**. 2021, **11**:638148.

119. Gopalakrishnan V, Spencer CN, Nezi L, Reuben A, Andrews MC, Karpinets TV, Prieto PA, Vicente D, Hoffman K, Wei SC *et al*: **Gut microbiome modulates response to anti-PD-1 immunotherapy in melanoma patients**. *Science* 2018, **359**(6371):97-103.

120. Routy B, Le Chatelier E, Derosa L, Duong CPM, Alou MT, Daillere R, Fluckiger A, Messaoudene M, Rauber C, Roberti MP *et al*: **Gut microbiome influences efficacy of PD-1-based immunotherapy against epithelial tumors**. *Science* 2018, **359**(6371):91-97.

121. Reiter RJ, Sharma R, Rosales-Corral S, Manucha W, Chuffa LGdA, Zuccari DAPdCJIjoms: **Melatonin and pathological cell interactions: Mitochondrial glucose processing in cancer cells**. 2021, **22**(22):12494.

122. Ramos E, Egea J, Lopez-Munoz F, Gil-Martin E, Romero A: **Therapeutic Potential of Melatonin Counteracting Chemotherapy-Induced Toxicity in Breast Cancer Patients: A Systematic Review**. *Pharmaceutics* 2023, **15**(6):1616.

123. Onseng K, Johns NP, Khuayjarernpanishk T, Subongkot S, Priprem A, Hurst C, Johns J: **Beneficial Effects of Adjuvant Melatonin in Minimizing Oral Mucositis Complications in Head and Neck Cancer Patients Receiving Concurrent Chemoradiation**. *J Altern Complement Med* 2017, **23**(12):957-963.

124. Hansen MV, Madsen MT, Andersen LT, Hageman I, Rasmussen LS, Bokmand S, Rosenberg J, Gogenur I: **Effect of Melatonin on Cognitive Function and Sleep in relation to Breast Cancer Surgery: A Randomized, Double-Blind, Placebo-Controlled Trial**. *Int J Breast Cancer* 2014, **2014**:416531.

125. Roldan CJ, Huh B, Song J, Nieto Y, Osei J, Chai T, Nouri K, Koyyalagunta L, Bruera E: **Methylene blue for intractable pain from oral mucositis related to cancer treatment: a randomized phase 2 clinical trial**. *BMC Med* 2022, **20**(1):377.

126. Wen Y, Li W, Poteet EC, Xie L, Tan C, Yan L-J, Ju X, Liu R, Qian H, Marvin MAJJoBC: **Alternative mitochondrial electron transfer as a novel strategy for neuroprotection**. 2011, **286**(18):16504-16515.

127. Sikka P, Bindra VK, Kapoor S, Jain V, Saxena KK: **Blue cures blue but be cautious**. *J Pharm Bioallied Sci* 2011, **3**(4):543-545.

128. Komlódi T, Tretter LJN: **Methylene blue stimulates substrate-level phosphorylation catalysed by succinyl–CoA ligase in the citric acid cycle**. 2017, **123**:287-298.

129. Samad A, Samant R, Venkateshwara Rao K, Bhargava V, Sadique SI, Yadav R: **Oxaloacetate as a Holy Grail Adjunctive Treatment in Gliomas: A Revisit to Metabolic Pathway**. *Cureus* 2023, **15**(11):e48821.

130. Wiese EK, Hitosugi S, Loa ST, Sreedhar A, Andres-Beck LG, Kurmi K, Pang YP, Karnitz LM, Gonsalves WI, Hitosugi T: **Enzymatic activation of pyruvate kinase increases cytosolic oxaloacetate to inhibit the Warburg effect**. *Nat Metab* 2021, **3**(7):954-968.

131. Ijare O, Conway D, Cash A, Baskin D, Pichumani K: **CBMT-49. Oxaloacetate Alters Glucose Metabolism In Glioblastoma: C Isotopomer Study**. 2019.
